# Clinical similarity in cost-comparison evaluations: a systematic review of current methods in NICE appraisals and the development of a framework for the formal assessment of clinical similarity

**DOI:** 10.1136/bmjopen-2025-112164

**Published:** 2026-07-21

**Authors:** Steven J Edwards, Benjamin J Burgess, Nicole Downes, Sophie Ip, Archie Walters, Clare Dadswell

**Affiliations:** BMJ Technology Assessment Group (BMJ-TAG), BMJ Group, London, UK

**Keywords:** Systematic Review, QUALITATIVE RESEARCH, STATISTICS & RESEARCH METHODS, Network Meta-Analysis

## Abstract

**Objectives:**

To review how statistically non-significant indirect treatment comparison (ITC) results are interpreted within National Institute for Health and Care Excellence (NICE) cost-comparison evaluations (CCEs) and develop a framework to support interpretations of these results from Bayesian network meta-analyses (NMAs).

**Design:**

A systematic review of CCEs between 2017 (first introduced) and April 2025. A framework (point-and-density plots) was developed to better interpret statistically non-significant NMA results for CCEs.

**Data sources:**

CCEs were identified through NICE website searches, references of similar reviews and communications with NICE.

**Eligibility criteria:**

NICE technology appraisals (from 2017) that followed a CCE approach ab initio, had final guidance available and used non-statistically significant ITC results were included.

**Data extraction and synthesis:**

A single reviewer performed screening and data extraction with validation by a second reviewer. Narrative syntheses were performed separately for company, External Assessment Group (EAG) and committee perspectives. Point-and-density plots combine elements of forest plots and density plots alongside reporting the probability that a treatment is non-inferior relative to a comparator. These were applied to a recent CCE (TA1019) for crovalimab for patients with paroxysmal nocturnal haemoglobinuria.

**Results:**

Among 41 CCEs, EAGs raised concerns about statistically non-significant ITC results while companies relied heavily on them. Only ∼32% of CCEs applied formal methods to explore ITC result uncertainty.

For the example framework analysis, comparisons of crovalimab to eculizumab (mean difference (MD): 0.018; 95% CIs −0.22 to 0.25) and ravulizumab (MD: 0.079; 95% CIs −0.25 to 0.41) were statistically non-significant, with non-inferiority not demonstrated. However, point-and-density plots indicated a 95.9% and 86.3% probability of non-inferiority of crovalimab versus eculizumab and ravulizumab.

**Conclusions:**

Interpretations of statistically non-significant ITC results are inconsistent within individual CCEs and across appraisals. Implementation of the presented recommendations and framework would improve the consistency and robustness of CCEs.

**PROSPERO registration number:**

CRD420251034143.

**STRENGTHS AND LIMITATIONS OF THIS STUDY**

- The systematic literature review provides insight into company, External Assessment Group and committee perspectives on evidence used to support conclusions of clinical similarity within National Institute for Health and Care Excellence (NICE) cost-comparison evaluations (CCEs), with a focus on CCEs associated with the greatest amount of uncertainty.
- The inclusion of appraisals that did not use a CCE approach from the start, but introduced these later, may have provided additional insights not currently captured.
- The framework details how non-inferiority may be clearly and robustly assessed in NICE CCEs, addressing a known limitation with this pathway.
- Implementation of the framework becomes more challenging when non-inferiority margins (or other thresholds) are not available.
- A series of recommendations for NICE CCEs, where the results of indirect treatment comparisons are statistically non-significant, are provided that, if fully implemented, would substantially improve the robustness and clarity of assessments of clinical similarity within this pathway.

## Introduction

### Background

#### NICE cost-comparison evaluation process

Technology appraisals, such as those performed in England as part of the National Institute for Health and Care Excellence (NICE) technology appraisal programme, involve the assessment of the clinical and cost-effectiveness of specific drugs, technologies or other interventions. As part of the NICE technology appraisal process, independent academic groups, called External Assessment Groups (EAGs), critique company submissions on behalf of NICE. This critique is considered alongside the company submission in the NICE committees’ decision-making process. Complex health economic models are often required to assess whether the intervention of interest is likely to be a good use of National Health Service (NHS) resources, with decision-making informed by whether incremental cost-effectiveness ratios (ICERs) resulting from these models are within the range considered an acceptable and effective use of NHS resources and other considerations.^1^ However, since 2017, cost-comparison evaluations (CCEs) have been accepted within the NICE technology appraisal programme; CCEs may be considered suitable where a new intervention is ‘likely to provide similar or greater health benefits at similar or lower cost than the relevant comparator(s)’,^1–5^ with relevant comparators considered to be ‘those recommended in published NICE guidance for the same population’.^1^ CCEs involve a simpler comparison of costs and resources used between interventions, without the need for a complex health economic model such as those used in cost-utility analyses, but they require a strong assumption that the interventions being compared have similar clinical effectiveness and safety.^1 3^

CCEs were first introduced as one of three routes under the fast-track appraisal (FTA) process in 2017. In 2022, other routes via the FTA process, including where a base case ICER was <£10 000 per quality-adjusted life year (QALY) or where the most plausible ICER was <£20 000 per QALY and highly unlikely to be <£30 000, were removed and the new process focused solely on CCEs.^1–4 6 7^ CCEs are an alternative to the standard NICE single technology appraisal (STA) where a single new intervention is being assessed. They would not typically be used as an alternative to other appraisal types, such as multiple technology appraisals. The aim of CCEs is to provide an assessment of clinical and cost-effectiveness for suitable interventions that can be used for decision-making but in a shorter time frame and using fewer resources compared with the STA process, as part of NICE’s commitment to taking a proportionate approach to technology appraisals.^8^

#### Challenges within cost-comparison evaluations

While the CCE process aims to improve the efficiency of the NICE technology appraisal process for appraisals that are considered to be a lower risk in terms of cost-effectiveness and whether they are an effective and acceptable use of NHS resources, making conclusions regarding whether there is sufficient evidence to conclude that a new treatment has ‘similar’ efficacy and safety to a NICE-recommended comparator treatment can be challenging.

Decisions about clinical similarity are often more straightforward within CCEs that include direct evidence from randomised controlled trials (RCTs) for the new treatment against at least one of the NICE-recommended comparators; evidence from non-inferiority, equivalence and superiority RCTs can provide robust evidence to support conclusions of clinical similarity or that the new treatment is considerably better than a relevant comparator treatment.^9^ However, NICE CCEs often do not have any direct comparative evidence for any relevant comparators and instead rely on the results of indirect treatment comparisons (ITCs) such as network meta-analyses (NMAs), matching-adjusted indirect comparisons, simulated treatment comparisons or simple Bucher ITCs.^4 5 10^

Conclusions about the clinical similarity of treatments based on ITC results can be challenging given these analyses often generate results that are not statistically significant and there is not currently any guidance from NICE on how such results should be interpreted in order to prove clinical similarity. While non-significant differences indicate that there is insufficient evidence to conclude that there are statistically significant differences between treatments, it does not prove similarity, non-inferiority or equivalence. Therefore, a lack of statistically significant differences alone should not be used as conclusive evidence that two treatments are similar in terms of clinical outcomes.^11^ Furthermore, it is possible that a difference in efficacy between two treatments may be clinically important even where the results are statistically non-significant.^12^ Therefore, the interpretation of results from ITCs that indicate no statistically significant differences between treatments is challenging in terms of whether the treatments can be considered clinically similar.

**Figure 1:**
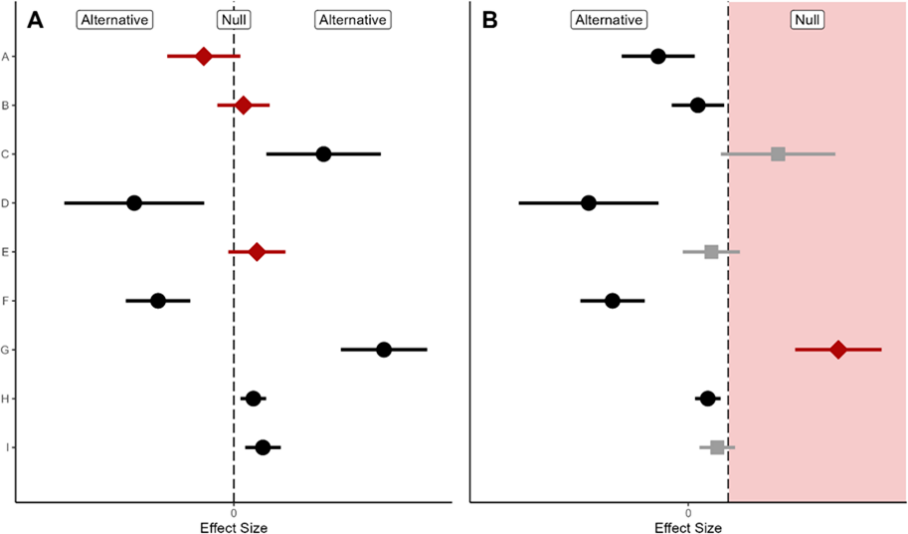
Illustration of traditional statistical significance testing (A) and non-inferiority testing (B). Points denote effect sizes, while lines denote 95% credible intervals. Dashed lines correspond to the thresholds of the null and alternative hypotheses. For traditional significance testing the dashed line corresponds to either 0 or 1 (depending on the given effect size). For non-inferiority testing the dashed line corresponds to the non-inferiority margin. Areas without shading correspond to the alternative hypothesis, areas with red shading correspond to the null hypothesis. Red diamonds denote effect sizes where the null hypothesis was accepted. Black circles denote effect sizes where the null hypothesis was rejected. Grey squares denote effect sizes where there is insufficient evidence to reject the null hypothesis.

As traditional statistical significance testing is not recommended for assessing clinical similarity, non-inferiority testing is of particular importance for CCEs, with such tests seeking to establish whether a new treatment is ‘not unacceptably’ worse than a comparator. For non-inferiority testing, an alternative threshold is specified, which is termed a non-inferiority margin (NIM) and represents the maximum reduction in clinical effectiveness that is considered acceptable while still considering the treatments to be equal.^9 13–15^ A pictorial comparison of traditional significance and non-inferiority testing is provided in figure 1.

#### Existing guidance and research

The NICE Health Technology Evaluations Manual does not provide any guidance on how clinical similarity can be concluded in situations where ITC results are not statistically significant and it only provides broad guidance on the type of evidence that can be considered or that is preferred; a preference for non-inferiority or equivalence RCTs with appropriate NIMs and including relevant comparators is expressed where possible, with meta-analysis and ITCs mentioned as alternatives if these sources of data are not available.^1^ It also mentions that sufficient certainty is required in conclusions of clinical similarity to appropriate comparators in order for committees to recommend a technology, although no further detail on what constitutes uncertainty is described.^1^

There also appears to be a lack of guidance on this elsewhere; while some discussion of clinical equivalence and non-inferiority is available in a NICE Decision Support Unit document produced by York Health Economics Consortium,^16^ this is limited to the interpretation of results from equivalence and non-inferiority trials and is not applied specifically to ITCs in this document. Furthermore, on review of all technical support documents developed by the NICE Decision Support Unit, no guidance to support with interpretation of non-significant results from ITCs was identified.^17^ This lack of guidance exacerbates the challenges associated with concluding whether treatments can be considered to have similar clinical outcomes based on statistically non-significant ITC results given there is a risk of inconsistent decision-making.

### Aims of this research

This research was commissioned by the National Institute for Health and Care Research (NIHR) Evidence Synthesis Programme (NIHR 176061). The project involved a systematic literature review (SLR) stage and a subsequent stage to develop a Bayesian framework to aid decision making.

The purpose of the SLR was to review and summarise the basis for decision-makers assuming clinical similarity based on statistically non-significant results obtained from ITCs within NICE CCEs, such as how often minimal clinically important difference (MCIDs) or other approaches are used for interpretation and decision-making. The aim was to gain insight into whether inconsistencies exist in current decisions or whether thresholds for clinical similarity have emerged organically from decision-makers.

After this, the project aimed to develop a Bayesian framework to support the interpretation of statistically non-significant results from Bayesian NMAs, with the framework able to integrate MCIDs or other thresholds where available (including any pragmatic thresholds considered appropriate by committees during their deliberations in the absence of established thresholds). Ideally, the goal was for the proposed framework to allow for consistent, robust and reproducible decision-making for future CCEs.

## Methods

### Systematic literature review

The protocol for the SLR was registered on PROSPERO (CRD420251034143; see online supplemental file 1); there were no major deviations from the protocol here.^18^ The SLR was reported in accordance with Preferred Reporting Items for Systematic Reviews and Meta-Analyses (PRISMA). For the SLR, searches of the main NICE website using “cost comparison”, “cost minimisation” and “cost minimization” terms (including quotation marks) were performed by a single reviewer in April 2025.^19^ Within the ‘Guidance programme’ filter, the ‘technology appraisal guidance’ option was selected to refine results. Results from each search were added to a Microsoft Excel spreadsheet and duplicate records were removed before screening. In addition, NICE were contacted regarding ongoing CCEs that might be published before completion of the SLR analysis and similar reviews of NICE CCEs were reviewed to identify any additional appraisals not identified through the searches.^2 3 20 21^

10.1136/bmjopen-2025-112164.supp1 Supplementary data

The SLR aimed to identify and include all NICE technology appraisals from 2017 onwards that followed the CCE approach from the outset, relied on results from ITCs that were not statistically significant for at least one comparator for the primary outcome(s) and had published final guidance before completion of the SLR. A date of 2017 onwards was applied given this is the year that CCEs were first introduced as an option by NICE.^22^ Further details regarding the inclusion and exclusion process, including pragmatic approaches that were required, are provided in online supplemental file 2. All NICE appraisals identified from the searches were screened against these criteria by a single reviewer. As validation, a second reviewer reviewed all decisions to determine whether they agreed or disagreed with the decision to include or exclude each appraisal. A third reviewer was available to resolve any disagreements. Given the time constraints of this review, the protocol outlined that the SLR would focus on the interpretation of any ITC results for primary outcomes used for decision-making within the CCE, given ITCs can be performed for numerous outcomes. Where no explicit mention of which outcomes were considered primary outcomes for decision-making in a specific CCE was made, outcomes that were defined as primary outcomes in the key clinical trials providing evidence for that appraisal or that were noted as being key components of economic models of earlier NICE technology appraisals in the same indication were considered to be the primary outcomes. Primary outcomes were not prespecified, given they were to be specific to each included appraisal, dependent on the disease area.

10.1136/bmjopen-2025-112164.supp2 Supplementary data

A single reviewer performed data extraction for each CCE using a standardised data extraction form in Microsoft Word, with a second reviewer validating a representative 30% sample of extractions. Key information extracted included project name and ID, disease area, primary outcomes used for decision-making, comparators requiring ITCs, type of ITC performed, results of committee-preferred ITCs and whether or not the treatment was recommended as a result of the CCE. Details on the methods used to support decision-making and the rationale for these were also extracted, and any comments made by either the company, EAG or committee relating to the ITC results and interpretation of clinical similarity were extracted from the documents in full for use in narrative syntheses. Further information on the data extraction process is outlined in online supplemental file 2.

As the SLR includes NICE CCEs, rather than clinical trials for which validated quality assessment tools exist, a risk of bias assessment was not performed within this SLR. A narrative synthesis of results was performed separately for company, EAG and committee perspectives, and an overall comparison between these groups was also considered. Tables were used to record new methods or themes identified from the extracted data and to collate all CCEs that mentioned each method or theme. Summary tables outlining the key findings have been presented alongside a narrative discussion of findings.

### Statistical framework

The aim of the new framework is to present the results of non-inferiority tests to allow NICE committees to make informed decisions regarding the clinical similarity of treatments, with a particular emphasis on CCEs. Importantly, the framework that has been developed can be viewed as an ‘add-on’ to existing methods for performing Bayesian NMAs. Although formal non-inferiority testing should remain the primary focus of any assessments of clinical similarity, it is anticipated that this new framework will be of particular use where there is no evidence of a significant difference between a treatment and a comparator.

**Figure 2:**
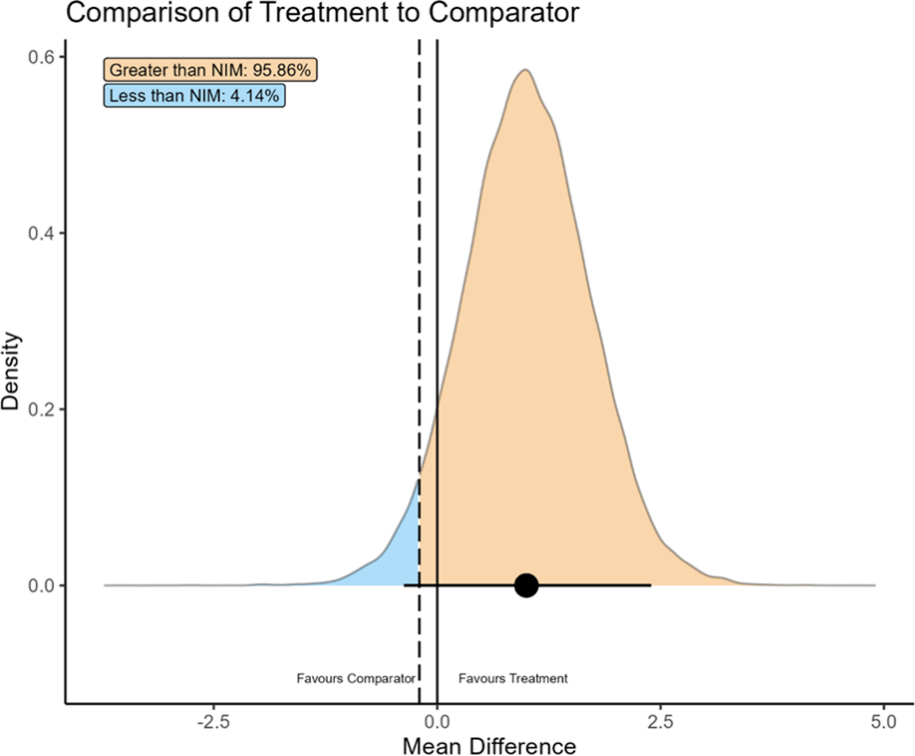
Point-and-density plot for the comparison of an example treatment and comparator. The threshold for traditional hypothesis testing is shown by the solid line, while the dashed line indicates the NIM. The area that falls under (above) the NIM is shown in blue (orange). The probability that the effect size falls under or above the NIM is shown in the upper left corner of the plot. The pooled estimate is shown by the black circle, with the error bars corresponding to 95% CrIs. CrIs, credible intervals; NIM, non-inferiority margin.

The statistical framework uses empirical cumulative density functions (ECDFs) to determine the probability that an effect size falls below a given threshold (eg, NIM). Accordingly, by combining the results of ECDFs with those of traditional density and forest plots, it is possible to convey all three separate analyses within a single readily interpretable figure, which addresses the lack of a specific visualisation for non-inferiority analyses. An example of this figure, termed a point-and-density plot, is shown in figure 2. In this example, the 95% credible intervals (CrIs) overlap both 0 and the NIM; as such, there is no evidence for a statistically significant difference between the treatment and comparator, while the non-inferiority of the treatment, relative to the comparator, is not demonstrated. However, there is a 95.86% probability that the treatment was non-inferior to the comparator. It is suggested that committees initially focus on whether the 95% CrIs overlap the NIM to determine whether a treatment is non-inferior. However, the use of probabilities of non-inferiority may provide valuable information, particularly where non-inferiority has not been demonstrated.

For some analyses, a NIM (or other threshold) may not be available. In such a situation, it is recommended that the corresponding threshold is set to either 0 or 1 (depending on the considered effect size), given that this represents a conservative threshold by which to assess non-inferiority. By using ECDFs, it is possible to determine a hypothetical, non-clinically validated threshold. Such a hypothetical threshold can be used to determine the required threshold for a given arbitrary probability that a treatment is non-inferior to a comparator. It is suggested that an arbitrary 95% probability of non-inferiority may be selected for a hypothetical threshold. In doing so, a 95% probability of non-inferiority represents a ‘high bar’ for any assessments of non-inferiority in the absence of a clinically validated NIM.

Full details of the statistical framework are provided in online supplemental file 3, with online supplemental figures 2-5 providing a visual representation of the processes applied within the framework. Furthermore, R code (and associated data) to generate point-and-density figures for the below case study are provided as online supplemental files 4,5.

10.1136/bmjopen-2025-112164.supp3 Supplementary data

10.1136/bmjopen-2025-112164.supp4 Supplementary data

10.1136/bmjopen-2025-112164.supp5 Supplementary data

#### Case study

To showcase how this framework may be used within CCEs, a case study is presented where the framework has been applied to data presented in a recent CCE. NICE TA1019 assessed crovalimab for the treatment of paroxysmal nocturnal haemoglobinuria (PNH) in people 12 years and over,^23^ with crovalimab subsequently being approved for use by NICE in line with its marketing authorisation. TA1019 compared crovalimab to the two treatments currently used to treat PNH, namely eculizumab and ravulizumab. Within the final draft guidance, NICE stated that the clinical trial evidence indicated that crovalimab works as well as eculizumab and is likely to be as effective as ravulizumab.^24^

Within TA1019, the company presented Bayesian NMAs for the primary co-endpoint from the COMMODORE 1 and 2 trials of transfusion avoidance (but not the primary co-endpoint of haemolysis control),^25 26^ which was assessed using the endpoint of mean difference (MD).^23^ Furthermore, the company stated that a NIM of 0.20 was considered based on data from a previous clinical trial.^23^ The company provided the results for random-effects (REs) Bayesian NMAs for the overall population, patients who had previously received complement inhibitors (experienced subgroup) and patients who had not previously received complement inhibitors (naive subgroup).^23^

For the case study, the Bayesian RE NMA for the overall population was reanalysed using data provided in the company’s submission for TA1019. As such, the results of the company’s submission are able to be compared with those from the reanalysis. Within the company’s submission, the company stated that meaningfully informative priors were used to estimate between-study heterogeneity; however, the company did not provide these informative priors in the submission.^23^ As such, any differences in the results of the original analysis and the equivalent analysis presented here are likely to be attributed, at least in part, to a difference in priors with uninformative priors used in the reanalysis.

### Patient and public involvement

There was no patient or public involvement in this research.

## Results

### Systematic literature review

#### Search and screening results

A total of 91 individual records of NICE technology appraisals were identified and screened for inclusion in this SLR, with 41 appraisals ultimately included. A full PRISMA diagram is presented in online supplemental figure 1. A range of disease areas were covered by the included appraisals. Multiple CCEs included in this SLR had been performed for certain conditions, such as plaque psoriasis and ulcerative colitis, while other conditions were only represented in one of the included CCEs (see online supplemental table 1). In terms of ITCs, over 60% relied solely on NMAs, with smaller percentages using other ITC methods. While all appraisals relied on ITC evidence for at least one comparator, ∼25% had direct trial evidence for another comparator relevant to the appraisal. Details of all included appraisals and a list of excluded appraisals are provided in online supplemental tables 1,2 respectively.

#### Themes identified

Narrative syntheses of company, EAG and committee approaches to the interpretation of statistically non-significant ITC results and additional evidence sources were performed. online supplemental tables 3-5 include an overview of the different methods used by companies, EAGs and committees to interpret the ITC results as well as additional methods used to support conclusions about clinical similarity, such as use of clinical expert feedback or reference to real-world data. An overall synthesis of these findings across these three groups is discussed narratively below, comparing and contrasting the approaches used across companies, EAGs and committees, with summaries relating to the interpretation of ITC results and use of supplementary evidence across these groups presented in tables 1,2 respectively.

**Table 1:** Overview of methods used to interpret statistically non-significant ITC results

Method	Company	EAG	Committee
No statistically significant differences	Some relied solely on a lack of statistically significant differences but most also discussed the direction/size of the point estimate. Companies generally did not acknowledge the uncertainty of statistically non-significant ITC results.	EAGs often considered statistically non-significant differences with or without consideration of point estimate size/direction to be insufficient. Limitations associated with statistically non-significant results from ITCs were often noted.	No clear position on whether statistically non-significant results from ITCs were sufficient for decision-making was identified.
Point estimate direction and/or size	Companies often heavily relied on point estimate direction/size. Some suggested that point estimates favouring the intervention for some analyses and the comparator for others was evidence of no consistent treatment difference. Some also suggested that clinical similarity was a conservative assumption where point estimates favoured the intervention.	Limitations of non-significant differences from ITCs were often highlighted, often noting that the inferiority of the new treatment could not be ruled out. However, many did mention them as supportive evidence and were reassured by review of other evidence or the robustness of the results to alternative analyses.	No clear position on the interpretation of point estimate direction and/or size was identified in most appraisals but point estimates close to the line of null effect in one appraisal reduced the committees’ concerns about uncertainty raised by the EAG.
Some statistically significant benefits of the new intervention	In some appraisals, statistically significant differences were identified from ITCs for some but not all analyses. Companies tended to consider this evidence that clinical similarity was conservative.	No strong EAG conclusions regarding these were noted. Some statistically significant benefits may have increased EAG confidence of clinical similarity, but concerns may have remained.	No explicit opinion from committees on this was identified within this SLR, but it may have provided some reassurance.
Probabilities calculated from NMAs	Probabilities from NMAs were mentioned in ∼15% of company submissions. However, not all used them to support their conclusions. Most involved SUCRA information from NMAs but two combined this with an NIM or clinical equivalence threshold, either from the outset or following a request from the EAG.	EAGs requested these methods from companies, suggested adaptations to company approaches or noted that they would have reduced uncertainty in ∼17% of appraisals. Simpler probability ranks and calculating probabilities of falling within a clinical equivalence range were noted as options.	No explicit opinion from committees on the application of this method for interpreting ITC results, and whether it would increase committee confidence for decision-making, was identified within this SLR.
MCIDs or other thresholds	Companies in ∼10% of appraisals incorporated MCIDs or NIMs. Thresholds were used either to assess whether point estimates represented a meaningful difference or incorporated into an NMA to calculate probabilities of clinical equivalence.	EAGs (∼17% of appraisals) used or adapted company threshold-based approaches or indicated that they would have been useful. Application of thresholds to assess whether ITC results were clinically meaningful or within NMAs to calculate probabilities were mentioned.	No explicit opinion on the application of this method for interpreting ITC results, and whether it would increase committee confidence for decision-making, was identified within this SLR.

EAG, External Assessment Group; ITC, indirect treatment comparison; MCID, minimal clinically important difference; NIM, non-inferiority margin; NMAs, network meta-analyses; SLR, systematic literature review; SUCRA, Surface Under the Cumulative RAnking curve.

**Table 2:** Overview of sources of supplementary evidence used to support conclusions of clinical similarity

Method	Company	EAG	Committee
Similar mechanisms of action	Similar mechanisms of action were mentioned in ∼15% of appraisals as supportive evidence that outcomes of the treatments were likely to be similar.	While not always explicitly used as supportive evidence for conclusions, ∼34% of EAGs commented on the similarity of mechanisms of action between the intervention and comparators.	Committee discussions often mentioned whether mechanisms of action are similar between treatments, and in ∼7% of appraisals, this may have reduced uncertainty and enabled conclusions of clinical similarity.
Clinical expert feedback	Clinical expert feedback was mentioned by companies in ∼24% of appraisals, either based on clinician experience with the treatments or anticipated differences, or clinician review of ITC results to determine whether differences were clinically meaningful or not.	EAGs mentioned clinical expert feedback in ∼34% of appraisals, including expert feedback that there are no differences between treatments based on their experience, that no differences were anticipated based on their knowledge of the treatments and/or support with interpretation of results, such as thresholds for clinically meaningful differences.	Committee discussions did not routinely include statements about clinical expert opinion. However, where EAGs raised uncertainty with ITC results, this was cited as a reason that the committee was ultimately able to conclude clinical similarity in ∼17% of all included appraisals.
Evidence of a class effect from meta-analyses	Some companies highlighted previously published meta-analyses of the intervention and comparators against a common comparator (eg, placebo) as supportive evidence of a class effect by some companies.	In two cases where the company suggested this, it was rejected by the EAG as a robust source of evidence, although it appeared to be of some use to the EAG in the third appraisal.	This was mentioned in the committee discussion of one of these appraisals, but there was no indication of whether it was considered useful to the committee.
Previous ITC results or NICE appraisals	Existing ITCs and/or NICE appraisals were cited in four appraisals, with results said to be similar to the current appraisal or decisions made based on a similar level of uncertainty to a previous appraisal.	These were mentioned by EAGs in a similar number of appraisals, including references to similar results being obtained or a similar level of uncertainty within ITC results that had not precluded a conclusion of clinical similarity.	These were generally not mentioned in documents summarising committee discussions.
Real-world data	One company had SACT data available, which was used as supportive evidence alongside ITC results.	The EAG also made use of these data when making its conclusions.	These data were not mentioned in the committee discussion of the evidence.
Other EAG sources	NA	Naive comparative evidence (for example, a visual comparison of KM curves from each study or statistical testing of differences) was used by some EAGs.	This was mentioned in some committee discussions, suggesting it was of some use, but was not mentioned in others.

EAG, External Assessment Group; ITC, indirect treatment comparison; KM, Kaplan-Meier; NA, not applicable; NICE, National Institute for Health and Care Excellence; SACT, systemic anti-cancer therapy.

The key finding was a lack of consistency in terms of methods used to interpret statistically non-significant ITC results and whether these were considered sufficient evidence of clinical similarity. While inconsistency existed across appraisals, EAGs tended to raise concerns about relying solely on these results as evidence of clinical similarity and companies usually placed a strong emphasis on the lack of statistically significant differences. EAGs sometimes concluded that there was too much uncertainty within ITCs for a CCE to be appropriate, while others considered supplementary information such as similar mechanisms of action, clinical expert feedback, previously published ITC results or NICE appraisals and/or naive comparative data to alleviate these concerns.

More formal methods of exploring uncertainty were sought by some EAGs and/or companies, including the application of MCIDs or NIMs and/or presentation of probabilities based on Surface Under the Cumulative RAnking curves from NMAs. However, one or more of these approaches were only explored in a minority (∼32.0%) of appraisals. Of note, no insight into committee preferences regarding which methods are most useful in terms of interpreting these results was identified from the appraisals, but committees frequently made use of clinical expert feedback and information on mechanisms of action where uncertainty was noted. To better illustrate these differences, the approaches used and conclusions made by companies, EAGs and committees in two of the included CCEs are summarised in table 3. These examples show how companies often relied on the lack of statistically significant differences and that EAGs often looked to expand on this by using additional information to inform their conclusions about clinical similarity. These examples also show the differences between EAGs in terms of non-statistically significant differences; one strongly dismisses their use in judging clinical similarity, while another does not dismiss them but uses other data to add to these observations. Furthermore, one EAG used clinically meaningful thresholds to facilitate interpretation of the ITC results, sought via clinical expert feedback, while another referred to other sources of data to support conclusions from ITCs. The researchers consider that these differences in approach are primarily due to the lack of guidance for companies and EAGs on how to approach the interpretation of non-statistically significant ITC results, including suitable additional methods that may improve decision-making, within NICE CCEs.

**Table 3:** Comparison of methods used across two CCE examples

Study information	Acalabrutinib for treating chronic lymphocytic leukaemia (TA689)^33^	Zanubrutinib for treating chronic lymphocytic leukaemia (TA931)^34^
Primary outcome(s)	OS. PFS.	OS. PFS.
Company approach	Focus on lack of statistically significant differences and trend towards point estimates favouring the intervention as evidence of clinical similarity. Similar mechanisms of action and clinical expert feedback also noted as supplementary evidence.	Focus on lack of statistically significant differences and some point estimates favouring the intervention as evidence of clinical similarity. Supplementary discussion of clinical expert feedback that treatments likely similar.
EAG approach	Does not reject the company’s conclusions relating to statistically significant differences outright. Also considered similarity of adjusted KM curves, clinical expert feedback, the limitations of the ITCs performed in terms of power available to detect statistically significant differences and variability across sensitivity analyses. Concluded that clinical similarity was a reasonable conclusion for one of the two included populations.	Dismisses lack of statistically significant differences and point estimates favouring the intervention as evidence of clinical similarity. Notes uncertainty and does not agree with CCE approach. Incorporates clinically meaningful differences based on clinical expert feedback into its interpretation of the results (upper CIs crossed these).
Committee considerations	Acknowledges uncertainties relating to the conclusions but concludes that clinical similarity is a reasonable conclusion. Considered clinical expert feedback alongside ITC results.	CCE approach was rejected given too much uncertainty in the ITC results.

CCE, cost-comparison evaluation; CI, confidence interval; EAG, External Assessment Group; ITC, indirect treatment comparison; KM, Kaplan-Meier; OS, overall survival; PFS, progression-free survival; TA, technology appraisal.

Two more general themes relating to CCEs were also identified, including improving the transparency of the decision-making process within committee discussions and clarifying whether only one relevant comparator outlined in the NICE final scope needs to be covered. These are summarised in online supplemental table 6; they are not addressed by the framework described in this publication but were considered to be useful findings that may be addressed through updated guidance or reporting of committee discussions by NICE.

### Statistical framework

A recent CCE, TA1019,^23^ assessing crovalimab in patients with PNH, was used as a case study to illustrate the described framework. The SLR performed by the company for TA1019 identified five trials reporting data on transfusion avoidance in people with PNH, which formed a star-shaped network (online supplemental figure 6). Two trials compared ravulizumab to eculizumab, two trials compared crovalimab to eculizumab and one trial compared eculizumab to standard of care, with data for each of the included trials provided in online supplemental table 7.

**Figure 3:**
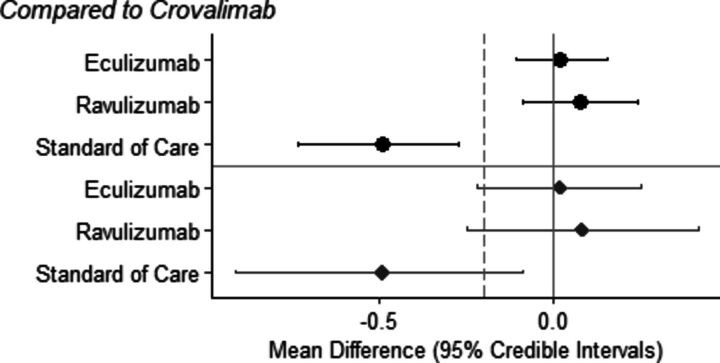
Results of the random-effects network meta-analyses, for the percentage of patients achieving transfusion avoidance in the overall population, presented by the company^23^ and as obtained from the case study reanalysis. Effect size estimates with black circles and red diamonds are from the analyses presented in TA1019 and the case study reanalysis, respectively.

The results of the RE NMAs from TA1019 and the case study re-analysis are presented in figure 3. Within the company submission,^23^ the company noted that the results of this NMA indicated that there was no statistically significant evidence for a difference between crovalimab and both eculizumab (MD: 0.017; 95% CrIs −0.11 to 0.15) and ravulizumab (MD: 0.077; 95% CrIs −0.09 to 0.24) as the 95% CrIs overlapped 0 for both comparisons. However, the 95% CrIs for the comparison of crovalimab to ravulizumab overlapped the NIM of 0.20 (ie, 20%), while the 95% CrIs for the comparison of crovalimab to eculizumab did not. Accordingly, crovalimab would be considered to be non-inferior to eculizumab but non-inferiority was not demonstrated compared with ravulizumab, although this was not explicitly stated in the company’s submission. However, the company stated in text, that there was a 99% and 93% probability of crovalimab being non-inferior to eculizumab and ravulizumab, respectively, when considering the NIM of 0.20.^23^ For the case study reanalysis, the point estimates for each of the comparisons of crovalimab to eculizumab (MD: 0.018; 95% CrIs −0.22 to 0.25) and ravulizumab (MD: 0.079; 95% CrIs −0.25 to 0.41) align with those from the analysis in the company’s submission. However, the 95% CrIs intervals associated with each point are much wider than those in the original analysis. As previously noted, this is expected to be due to the use of informative priors in the company’s original analysis.

**Figure 4:**
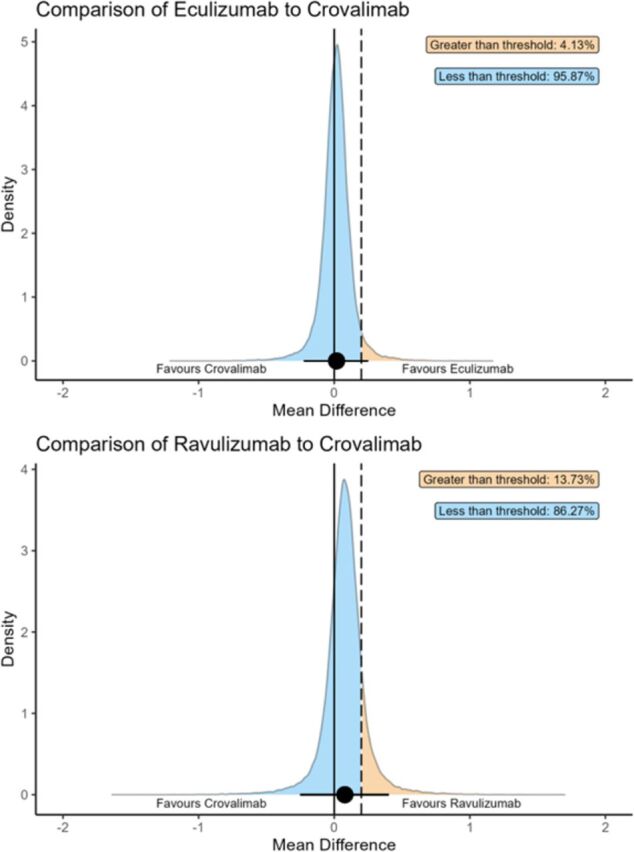
Point-and-density plots for the comparisons of eculizumab to crovalimab and ravulizumab to crovalimab for the percentage of patients achieving transfusion avoidance in the overall population.

Results of the RE NMAs from the case study reanalysis were additionally analysed using the new statistical framework with point-and-density plots shown for the comparisons of crovalimab to eculizumab and crovalimab to ravulizumab (figure 4). As shown by the point-and-density plots, there was a relatively high probability of crovalimab being non-inferior compared with both ravulizumab (86.27%) and eculizumab (95.87%). Additionally, through the use of ECDFs it was possible to determine that a hypothetical threshold of 0.31 would be required in order for there to be a 95% probability that the point estimate, for the comparison of crovalimab to ravulizumab, was below this hypothetical threshold.

Within the reanalysis, the non-inferiority of crovalimab relative to both eculizumab and ravulizumab was not statistically demonstrated. The point-and-density figures illustrated that there was a high probability that the difference in treatment effects would be considered non-inferior for comparisons of crovalimab to both eculizumab and ravulizumab. Accordingly, the results of the reanalysis for the overall population support the use of a cost-comparison for TA1019.

## Discussion

### Systematic literature review

The key finding from the SLR was inconsistency with regard to how statistically non-significant differences from ITCs were interpreted within CCEs; companies frequently interpreted these as sufficient evidence for clinical similarity, while EAGs generally seem to be aware of the importance of not misinterpreting the absence of a statistically significant difference as evidence of clinical similarity. Methods emerging from the current appraisals to further explore these ITC results include specific tests of non-inferiority using a threshold such as a clinically derived NIM or MCID. However, only a minority of CCEs in this SLR made use of such methods. Furthermore, additional detail within committee discussions would improve the transparency of decision-making in these appraisals, given it was often limited and clarity regarding how many comparators need to be covered from the NICE final scope would be beneficial for companies and EAGs.

Findings from the SLR are largely in line with a recent publication by PenTAG, an EAG working on NICE appraisals, which also raised concerns about relying on statistically non-significant ITC results in CCEs and made recommendations on methods that may improve transparency in decision-making.^21^ However, another EAG recently concluded that a lack of statistically significant differences or overlapping 95% CIs may be sufficient to demonstrate clinical similarity in CCEs.^27^ Therefore, the current lack of guidance in this area of NICE CCEs may also be contributing to differences in perspectives across EAGs, and work on methods that can support the interpretation of statistically non-significant ITC results may improve the consistency and transparency of decision-making within CCEs.

Strengths of this SLR include the focus on CCE appraisals relying on non-significant ITC results; given there is more uncertainty associated with this type of evidence, this has allowed the review to focus specifically on methods being used in situations where there is the highest uncertainty in conclusions and the greatest need to identify methods to address this. Furthermore, a thorough review of company, EAG and committee perspectives has been performed, allowing the identification of similarities and inconsistencies in approaches across appraisals and also between companies, EAGs and committees. A potential limitation of this SLR is that keywords were used to search the NICE website rather than reviewing the full list of NICE technology appraisals, but on review of appraisals included in the work by PenTAG (which used the latter approach), the researchers are reassured that this approach did not lead to many relevant appraisals being missed.^21^ Furthermore, inclusion of appraisals that did not use a CCE approach from the start but introduced them later in the process was not possible due to limited resources; it is possible that these types of appraisals may have provided useful additional information but the researchers consider it unlikely that the findings of this SLR would change considerably if these appraisals had been considered. Finally, it was not possible within the time constraints of this research to perform searching, screening and data extraction completely in duplicate using two independent reviewers, which is a further limitation of the SLR in this research; however, approaches to validate each of these stages using input from a second reviewer were implemented to minimise the risk of any errors during these stages.

### Statistical framework

There is currently uncertainty, from pharmaceutical companies, regarding how NICE assesses equivalence or non-inferiority, within NMAs and other forms of ITCs.^21^ As such, the framework presented here provides a clear outline for how non-inferiority may be assessed within CCEs that use NMAs. Accordingly, a key strength of the newly developed framework is that it addresses a known challenge that NICE committees face when evaluating CCEs, namely, how to assess clinical similarity when the results of NMAs indicate that there is no evidence for a statistically significant difference. As shown by the results of the SLR, there are no methods or frameworks that are consistently applied in CCEs, that use NMAs, to assess clinical similarity.

Within this newly presented framework, specifically through the point-and-density plots, an approach has been provided that is readily interpretable and provides information that was previously unavailable, to allow NICE committees to draw robust conclusions as to whether a new treatment is non-inferior to an existing comparator. Crucially, when non-inferiority, or inferiority, has not been demonstrated in a direct comparison within an RCT, the framework presents the probability that the new treatment is non-inferior to an existing comparator. As such, this allows NICE committees to make informed decisions on the likelihood of a new treatment being clinically similar to an existing comparator, even when non-inferiority has not been statistically demonstrated. Additionally, the proposed framework has been implemented within an EAG’s review of a recent CCE for resectable non-small-cell lung cancer.^28^

Furthermore, a recent review of CCEs and methodological approaches that are used to assess non-inferiority reported that visualisations to specifically assess non-inferiority were scarce and that where such visualisations were provided, the most common approach was to present forest plots with an overlaid NIM.^21^ As such, NIMs may be viewed as being ‘shoehorned’ into existing visualisations with little consideration of the most appropriate manner of displaying such information.^21^ Accordingly, the new framework proposed here helps to overcome this limitation through the development of point-and-density plots that specifically seek to display the results of NIMs in a dedicated, intuitive manner.

As has been previously discussed, the majority of CCEs do not provide a NIM, MCID or another threshold to allow for a statistically robust assessment of non-inferiority. As a consequence, clinical similarity has typically been erroneously assessed through inappropriate statistical approaches.^27^ However, there is disagreement between EAGs regarding what constitutes an appropriate method to assess clinical similarity within CCEs.^21 27^

Where a NIM or MCID is not available, the new framework presented here allows for a ‘hypothetical threshold’ to be calculated which refers to the required value of a NIM in order for an arbitrary probability (eg, 95%) that a treatment is non-inferior to a comparator. Such an approach is a strength of the new framework and provides further context to EAGs and committees with regards to the likelihood of a new treatment being non-inferior, relative to an existing comparator, when such information would not otherwise be available. Although it is possible to calculate a ‘hypothetical threshold’ where a NIM, MCID or other threshold has not been provided, it is strongly recommended that this approach should not be considered a substitute for the provision of a clinically validated NIM or MCID. Indeed, it is recommended that a company should provide a clinically validated NIM or MCID for all primary or coprimary outcomes in order for an appraisal to be considered for the CCE pathway. The absence of a clinically validated NIM or MCID severely limits the ability of a committee or EAG to reach definitive conclusions as to whether a new treatment is non-inferior or clinically similar to a previously approved comparator. Accordingly, when a ‘hypothetical threshold’ is implemented is it imperative that it is labelled as such, in order to prevent EAGs and committees from erroneously assuming that the selected threshold was clinically validated.

Within this framework, a 95% threshold for assessing non-inferiority has been recommended. The researchers acknowledge that this threshold represents a ‘high bar’ for assessing non-inferiority. As such, if this framework is implemented in CCEs, committees and EAGs may wish to explore alternative thresholds for assessing non-inferiority (eg, an 80% or 90% probability of being non-inferior), in doing so assessing how sensitive assessments of non-inferiority are to the choice of hypothetical threshold.

The proposed framework is only able to be implemented with Bayesian and not frequentist NMAs. For frequentist NMAs, fixed parameters represent population characteristics, therefore meaning that these analyses can only help researchers decide whether to accept or reject a hypothesis based on statistical significance.^29 30^ In contrast, Bayesian NMAs use a probability distribution of model parameters which, through Markov Chain Monte Carlo simulations, generate a posterior probability distribution.^30^ Within the framework, the posterior probability is used to determine the probability of non-inferiority. Although studies have suggested that there are no important differences in the performance of these approaches, it has been reported that Bayesian NMAs are more frequently used compared with frequentist NMAs.^30 31^ Accordingly, given the predominance of Bayesian NMAs, and the availability of software to perform Bayesian analyses,^30^ the sole applicability of the framework to Bayesian NMAs is not considered to be a limitation of the approach. Alongside the choice of Bayesian or frequentist methods, NMAs are dependent on numerous factors that should be considered a priori (eg, the choice of a fixed-effect (FE) or REs model) based on clinical or statistical rationale and not selected to allow the presented framework to be applied. However, it should be noted that the presented framework can be used for both FE and REs Bayesian NMAs. Additionally, although the presented case study centred on the effect size of MD, the framework is applicable to multiple other effect sizes (eg, odds ratios, hazard ratios or standardised MDs). Finally, we note that NMAs are the most commonly implemented approach for ITCs of fully connected networks. However, multilevel network meta-regression (ML-NMR) is a recent Bayesian ITC method that is becoming more widely adopted.^32^ Accordingly, future research may seek to allow the presented framework to be applied to both Bayesian NMAs and ML-NMRs.

The presented framework has been recently implemented within an EAG’s review of a recent CCE.^28^ Although the framework addresses issues with how NICE assesses non-inferiority in CCEs, the adoption of this method by EAGs, NICE committees and pharmaceutical companies may take some time. Conversely, alternative, statistically rigorous, frameworks may be developed and subsequently implemented. Accordingly, it is suggested that a future update to the SLR is performed, hence allowing the uptake of the proposed framework to be comprehensively assessed.

### Recommendations

A number of recommendations that cover all aspects of the approach to assess clinical similarity within CCEs, resulting from both the SLR (Recommendations 1–6) and the framework (Recommendations 7–10) have been suggested. As such, NICE CCEs would be methodologically and clinically strengthened if all of these recommendations were to be adopted.

1. Use of MCIDs or NIMs should be considered to support the interpretation of statistically non-significant results from ITCs. Where these are not available or cannot be estimated robustly, clinical expert advice could be sought to estimate pragmatic thresholds for the purpose of the appraisal.
2. The incorporation of thresholds (eg, NIMs or MCIDs) into Bayesian analyses to obtain probabilities of the new treatment being no worse than one or more established comparators with existing NICE guidance would be a robust method of using such thresholds. When multiple comparators exist, presentation as pairwise comparisons against each comparator may be useful given that committees evaluate the evidence to support a CCE against each comparator separately. Where a suitable threshold has not been identified, even with clinical expert input, probabilities of being the best treatment within a Bayesian network or being better than each comparator may be useful for decision-making purposes. When a Bayesian analysis is not possible, other methods of using MCIDs or NIMs to support the interpretation of results should be considered.
3. Guidance related to the uncertainties of statistically non-significant ITC results within the NICE methods manual or user guides for submissions may be useful to improve the case put forward in company submissions and provide support for EAGs in terms of what information or methods might be reasonable to request. This may also improve consistency across EAGs in terms of how statistically non-significant ITC results are interpreted, given the different positions noted in recent publications by EAGs in this area.^21 27^ This guidance need not be prescriptive but could simply highlight the limitations of relying on statistically non-significant differences from ITCs and that additional methods may be useful to support conclusions of clinical similarity.
4. Supportive information such as clinical expert feedback, comments on the similarity of mechanisms of action and consideration of naive comparative evidence should not be considered sufficient to resolve uncertainty associated with statistically non-significant ITC results. Statistically robust approaches should be explored to improve confidence in conclusions of clinical similarity based on ITC results, with supportive information used to supplement these conclusions.
5. Additional detail regarding committees’ interpretation of statistically non-significant ITC results within published NICE guidance for each appraisal may improve transparency within the decision-making process, particularly where there is discordance between overall conclusions made by the EAG and the committee.
6. Clarity on whether or not a CCE needs to make a case against multiple or only one relevant comparator within the NICE CCE process may be useful. Explanation of when a single comparator may be deemed appropriate within the NICE process and whether companies should still explore all or multiple comparators in their original submission might address this uncertainty.
7. CCEs should implement a consistent framework to support the interpretation of statistically non-significant results from NMAs. Through utilisation of point-and-density plots, committees and EAGs will be presented with results in a consistent format, while all stakeholders will have clarity regarding how clinical similarity is assessed.
8. When an established NIM or MCID is used, it is recommended that a probability of 95% is used to determine if a new treatment can be considered to be ‘clinically similar’ to a comparator.
9. When a NIM or MCID is unavailable, it is recommended that a value representing no difference is used to assess clinical similarity, given that this represents a conservative estimation of non-inferiority.
10. In exceptional circumstances, a committee may want to use an estimated hypothetical threshold that is required for a treatment to be considered ‘clinically similar’ to a comparator. In doing so, this approach would allow the committee to qualitatively determine how different the estimated results are from this theoretical threshold.

## Conclusions

NICE requires that CCEs demonstrate that new interventions are clinically similar to relevant comparators already approved by NICE. Here, the SLR sought to provide an overview of the current approaches that are used within CCEs with statistically non-significant ITC results to conclude clinical similarity. Furthermore, the newly developed Bayesian framework sought to provide EAGs and NICE committees with additional insights regarding clinical similarity. Overall, this research provides vital information that may result in better-informed decision-making when assessing CCEs.

The SLR determined that assessments of clinical similarity were not consistent in the identified CCEs. Companies frequently interpreted statistically non-significant differences as evidence for clinical similarity, while EAGs often correctly refuted these assertions. Importantly, an absence of a statistically significant difference between two treatments is not evidence of clinical similarity. Instead, clinical similarity should be assessed through specific tests of non-inferiority that use a clinically relevant threshold (eg, NIMs or MCIDs). The SLR indicated that 75% of CCEs did not use such approaches. Furthermore, there are some uncertainties regarding how NICE committees determine whether a new treatment is considered clinically similar to a comparator. For instance, it is unclear what methods NICE considers acceptable when relying on statistically non-significant differences from ITCs and whether clinical similarity is required to be demonstrated for an intervention relative to one or all relevant comparators.

The framework was developed to overcome several limitations of the current approach to assessing clinical similarity in the CCE pathway. Specifically, the framework represents a consistent and rigorous approach to assessing clinical similarity where the results of a Bayesian NMA are statistically non-significant. If this framework were to be widely implemented within CCEs, it would eliminate the current methodological inconsistencies in how clinical similarity is assessed, while also providing clarity to all stakeholders involved in the process. Accordingly, point-and-density plots are easily understandable through the combination of components from multiple commonly used figure types, and represent a stepwise improvement over previously used visualisations, given that these point-and-density plots have been designed specifically for CCEs. Following the completion of the SLR and development of the framework, the researchers have made a number of recommendations which, if all were to be implemented, would enhance the robustness and transparency of the NICE CCE pathway.

## Supplementary Material

Reviewer comments

Author's manuscript

## Data Availability

All data relevant to the study are included in the article or uploaded as supplementary information. Additional information on the methods and results of this research has been provided in the online supplementary files.
