# Supplementary material for: Clinical similarity in cost-comparison evaluations: a systematic review of current methods in NICE appraisals and the development of a framework for the formal assessment of clinical similarity
Source: BMJ Open. 2026 Jul 21;16(7):e112164. doi: 10.1136/bmjopen-2025-112164 (PMC13410701; doi:10.1136/bmjopen-2025-112164)
Supplement: Supplementary data [file bmjopen-16-7-s001.pdf]

Evaluating clinical similarity: a systematic review of NICE guidance and the development of a Bayesian framework for a formal assessment of clinical similarity

Protocol

April 2025

Edwards SJ, *et al. BMJ Open* 2026; 16:e112164. doi: 10.1136/bmjopen-2025-112164

1 Title of the project

Evaluating clinical similarity: a systematic review of NICE guidance and the development of a Bayesian framework for a formal assessment of clinical similarity.

2 Name of TAR team and project 'lead'

BMJ Technology Assessment Group (BMJ-TAG)

Lead: Steve Edwards

Director of Health Technology Assessment

BMJ Group

5th Floor, 90 Whitfield Street, London W1T 4EZ

3 Plain English Summary

Cost-comparison evaluations (CCEs) are one type of technology appraisal being used by the National Institute for Health and Care Excellence (NICE) to assess the clinical and cost effectiveness of new treatments. It allows new treatments to be compared against a comparator treatment in the same

Edwards SJ, *et al. BMJ Open* 2026; 16:e112164. doi: 10.1136/bmjopen-2025-112164

indication that has already been recommended by NICE and is likely to be "clinically similar", with no requirement for complex health economic modelling and a process that aims to be faster than assessments requiring more complex modelling.

Deciding whether two treatments are "clinically similar" can be difficult if there are no trials directly comparing the two treatments and an indirect treatment comparison (ITC) has been required. ITCs often report no statistically significant difference, which cannot itself be interpreted as evidence of two treatments being clinically similar. There is thought to be variation in terms of how clinical similarity is currently assessed in these cases within NICE CCEs. The availability of thresholds such as minimally clinically important differences (MCIDs) may help with the interpretation of clinical similarity, but these are not always available and it is unclear how often these or other methods are used to support decisions about clinical similarity within NICE CCEs.

This research aims to review all CCEs performed as part of the NICE technology appraisal programme since 2017 to identify and summarise any variations in the methods used to support decisions about clinical similarity when ITCs have been required. Subsequent to this, a novel Bayesian framework will be developed with the aim of supporting the interpretation of non-significant results from Bayesian network meta-analyses (NMAs). Worked examples demonstrating the use of the new tool will also be provided.

4 Research question

4.1 Purpose

Technology appraisals, such as those performed in England as part of the National Institute for Health and Care Excellence (NICE) technology appraisal programme, involve the assessment of the clinical and cost-effectiveness of specific drugs, technologies or other interventions. Most appraisals require complex health economic models, such as cost-utility analyses, to assess whether the intervention of interest is likely to be a good use of NHS resources. However, since 2017, cost comparison evaluations (CCEs) have been accepted within the NICE technology appraisal programme

under specific circumstances. CCEs involve a simpler comparison of costs and resources used with one drug compared to another, without the need for a complex model such as those used in cost-utility analyses, but they require a strong assumption that the drugs being compared are similar in terms of clinical effectiveness.

CCEs were first introduced as one option under the fast-track appraisal (FTA) process in 2017; while the FTA process itself was replaced in 2022, its replacement was a process focusing only on CCEs and methods relating to the CCE itself did not notably change. For an appraisal to be suitable for the cost comparison route, Section 4.2.13 of the 2022 NICE health technology evaluations manual states that the new interventions should be, "likely to provide similar or greater health benefits at similar or lower cost than the relevant comparator(s)"; relevant comparators are considered to be, "those recommended in published NICE guidance for the same population",[1] which is consistent with requirements described for the CCE process prior to 2022.[2] The aim of CCEs is to provide an assessment of clinical and cost-effectiveness for suitable drugs that can be used to decide whether the they should be recommended but in a shorter time frame and using fewer resources compared to technology appraisals using cost-utility methods, as part of NICE's commitment to taking a proportionate approach to technology appraisals.[3]

While the clinical similarity of two interventions can be robustly demonstrated through non-inferiority randomised controlled trials (RCTs) using prespecified non-inferiority thresholds or clinical equivalence RCTs,[4] many CCEs submitted to NICE do not involve such trials. Instead, they rely on the results of indirect treatment comparisons (ITCs) such as network meta-analyses (NMAs), matching-adjusted indirect comparisons (MAICs) or simple Bucher ITCs. These analyses often result in non-significant results based on confidence intervals or credible intervals that cross the line of null effect. A lack of a statistically significant difference should not be used to determine that two treatments are clinically similar; these analyses are often based on a small number of studies with small sample sizes, meaning there is considerable uncertainty in the effect estimate that should not be interpreted as an absence of a difference. Therefore, the interpretation of these results in terms of whether interventions can be considered clinically similar can be challenging. The use of minimally clinically important differences (MCIDs) established in the literature or estimated through other methods may aid the interpretation of results and the transparency of this process;[5] if the point estimates and uncertainty interval obtained from the ITC were less than (or mostly less than) the MCID, it may increase the confidence of decision-makers that the treatments are most likely to be clinically similar, as any difference that is present is unlikely to be clinically meaningful. Alternatively,

if the point estimate and uncertainty interval are greater than the MCID and in favour of the new intervention, there would be increased confidence that the new treatment may be better than the previously recommended intervention. However, established MCIDs are not available for all outcomes and it is unclear how often these methods are used currently to assess clinical similarity in NICE CCEs.

Through a systematic literature review (SLR), this research aims to review and summarise the basis for decision-makers assuming clinical similarity based on non-significant results obtained from ITCs as part of NICE CCEs, such as how often MCIDs or other approaches are used for interpretation and decision-making, and which thresholds have been used so far. The results of the SLR will support the development of a novel Bayesian framework that can integrate MCIDs or other thresholds to support the interpretation of non-significant results from Bayesian NMAs. Worked examples demonstrating the use of the tool, taken from the CCEs identified in the SLR, will be provided as part of this research. The SLR will highlight whether inconsistencies exist in current decisions or whether thresholds for clinical similarity have emerged organically from decision-makers, while the proposed framework should allow for consistent, robust, and reproducible decision-making for future CCEs.

4.2 Content of the systematic literature review

The SLR will aim to identify and include all NICE technology appraisals that:

- have followed the CCE approach for decision-making from the outset;
- rely on non-significant results from ITCs to assess clinical similarity for at least one comparator for the primary outcome(s);
- have published final guidance at the time of searching or where this can be obtained via NICE for appraisals due to publish final guidance imminently (i.e. before completion of the SLR).

Appraisals that only involve a non-inferiority or equivalence RCT (i.e. there are no comparators for which an ITC has been required) will not be included given these trials are considered to be robust methods of assessing clinical similarity and are not subject to the same uncertainties as results from ITCs. Similarly, appraisals that only include ITCs with statistically significant results

Edwards SJ, *et al. BMJ Open* 2026; 16:e112164. doi: 10.1136/bmjopen-2025-112164

will not be included, as there is less uncertainty associated with these results. Appraisals that include direct RCT evidence or statistically significant ITC results for one comparator but rely on the non-significant results of an ITC for another comparator will only be included if there are clear statements about the committee or external assessment group (EAG)'s conclusion of clinical similarity for the comparison where the ITC with non-significant results was performed; this is because clinical similarity only has to be demonstrated against one comparator in the NICE CCE process, meaning if multiple comparators are deemed appropriate, the decision-making is likely to have been based mostly on the one with the strongest evidence (in this case the one with direct RCT evidence or statistically significant ITC results). During the sifting stage, details about which appraisals involved decisions based on statistically significant results from ITCs or direct comparisons between treatments (and whether these were superiority, non-inferiority or equivalence trials) will be recorded, to allow brief comments to be made on this despite their exclusion from the main review.

Appraisals that have followed the CCE process from the start of the appraisal (i.e. where the company's first submission is a CCE) will be prioritised for inclusion. It is possible that some Single Technology Appraisals (STAs) start with cost-utility models but incorporate cost comparison approaches following requests from the EAG or committee. If time permits, these will be considered for inclusion but only if it is clear that decision-making was solely based on the cost comparison assessment given the exact contribution of the CCE to decision-making would otherwise be difficult to disentangle. An appraisal that has been a CCE from the start will usually be evident from the introductory pages of the NICE committee papers or the decision problem table submitted by the company, but other sections of the appraisal documents will be explored where this is unclear. Conclusions about whether decisions were based solely on cost comparison analyses for STAs may be available from committee discussion sections included in final guidance documents, but where this is unclear the appraisal will be excluded.

The review will aim to cover all CCEs meeting the above criteria from 2017 onwards; however, upon identification of relevant appraisals, prioritisation of those from 2022 onwards (given these represent the most recent examples of the NICE CCE process) may be required if the workload associated with this outweighs the resources available for this project. Due to the time constraints of this review, only the primary outcome(s) in each appraisal will be assessed with regards to how the decision of clinical similarity was made. It is anticipated that the documenting of which outcome is the primary outcome may be unclear in some appraisals; in

Edwards SJ, *et al. BMJ Open* 2026; 16:e112164. doi: 10.1136/bmjopen-2025-112164

this review, if not otherwise stated, the outcome that is discussed first, is most heavily discussed in the report or NICE committee meeting slides and/or is key in terms of any economic assessment presented will be considered to be the primary outcome. In cases where it appears there is more than one primary outcome, these will be considered separately. A clear rationale for why specific outcomes in each appraisal have been interpreted as the primary outcome will be included in the extraction tables.

4.3 Development of a Bayesian framework for a formal assessment of clinical similarity

The results of the SLR described in Section 4.2 will be used to support the development of a novel framework that utilises Bayesian NMAs to allow non-significant results to be interpreted more robustly. The framework will allow the inclusion of MCIDs or other thresholds when available to support with the interpretation of non-significant results. It is anticipated that this will involve building on existing, commonly used approaches such as cumulative density functions, point estimates, and credible intervals. Outputs will be designed such that they are clear and easily interpretable by stakeholders regardless of their statistical background. Worked examples demonstrating the use of the tool, taken from the CCEs identified in the SLR, will also be provided as part of this research. The methods for this phase of the research are not described further in this protocol but will be written up in the final report.

5 Methods for the systematic literature review

Methods described here are specifically for the systematic literature review (SLR) phase of this research, as outlined in Section 4.2. More detailed methods associated with the development of the Bayesian framework outlined in Section 4.3 are not included here but will be covered in the final report.

The SLR will be performed and reported according to the PRISMA statement. A flow diagram illustrating the number of records identified, included and excluded throughout the SLR will be presented according to the PRISMA reporting guidelines.[6]

Edwards SJ, et al. BMJ Open 2026; 16:e112164. doi: 10.1136/bmjopen-2025-112164

5.1 Search strategy

Multiple free-text searches of the National Institute for Health and Care Excellence (NICE) website will be performed by one reviewer. The terms "cost comparison", "cost minimisation" and "cost minimization" (including the quotation marks) will be searched using the search bar at the top of the main NICE webpage (https://www.nice.org.uk/). This will pick up any records including the exact terms "cost comparison" or "cost-comparison" while avoiding records that mention cost and comparison but not adjacently (or equivalent for cost minimisation and cost minimization). This is considered to be a reasonable approach given the term "cost comparison" is a formal and consistent term used by NICE in these appraisals and is likely to be mentioned somewhere in all appraisals that have followed the cost comparison evaluation (CCE) route. The terms "cost minimisation" and "cost minimization" will be included to identify any records that may have used this terminology instead, which can also be used to refer to the cost comparison approach in health economic assessment.

The results of the search will be further refined to show only technology appraisal guidance; the option to filter by guidance programme on the left-hand side of the search results page will be used to achieve this by selecting the "technology appraisal guidance" box. All records remaining after applying this filter will be screened for inclusion against the criteria outlined in Section 4.2.

For records that have not had final guidance published at the time of screening and that are due to publish before completion of the SLR, one reviewer will contact NICE to determine whether information on final guidance can be obtained to allow inclusion in this assessment. During the screening process, the contact person for appraisals that have not yet published final guidance will be captured to facilitate this. NICE will also be contacted with a full list of CCEs identified from the search of the NICE website, to ask whether it is aware of any other CCEs not captured by the search.

5.2 Review process

Given it is not possible to export records from the NICE website, records obtained from the search described in Section 5.1 will be manually added to a Microsoft Excel® spreadsheet for tracking and screening purposes. Records will then be screened for inclusion against the criteria outlined in Section 4.2 by a single reviewer. This will involve reviewing any information and documents published on the NICE website (or provided in communication with NICE) for each record in a single screening step; information within guidance documents and committee papers will be prioritised, but other documents such as final scopes may be reviewed if information remains unclear or if other

documents with key information are identified. Validation of this process will be performed by a second reviewer. Any disagreement will be resolved through discussion and involvement of a third reviewer if required to resolve outstanding conflicts.

5.3 Data extraction

Data for each included record will be extracted by a single reviewer into standardised data extraction forms using Microsoft Word®. Validation of a representative 30% sample of extractions will be performed by a second reviewer, with discussion of any discrepancies and involvement of a third reviewer if required. A draft data extraction form is included in Appendix 8.1 but is subject to change; a pilot of five extractions will be performed before validation by a second reviewer, after which the data extraction form will be finalised and used for the remaining extractions.

General information about each record such as project name, project ID and disease area will be extracted, as well as information about primary outcomes, comparators that the ITC was required for and type of ITC performed, date of final guidance publication and whether or not the treatment was recommended. Information about clinical similarity and reasons that decisions were made are likely to be reported in various ways across projects, but reviewers will be exhaustive and as a minimum will capture any information relating to:

- Any mention of ways of interpreting ITC results such as specific MCIDs or other thresholds and how these were identified;
- Results of the committee's preferred ITCs for relevant comparisons, including point estimates and measures of uncertainty (such as 95% confidence or credible intervals) of the effect estimates;
- Any committee or EAG comments on the uncertainty in the ITC, including what they thought about the uncertainty in the ITC and how it may have impacted their decision or conclusions;
- Brief comments on the availability of input data for the ITCs from NICE papers, to allow an assessment of how feasible it would be to replicate the ITCs from this information (and whether identification of data from individual publications would instead be required) for the worked Bayesian framework examples that will be provided as part of the second phase of this project;
- Narrative comments within company submissions, EAG reports or committee discussion documents describing how results of ITCs were ultimately interpreted and how clinical similarity was confirmed or refuted in the final decision-making process; clear statements

Edwards SJ, *et al. BMJ Open* 2026; 16:e112164. doi: 10.1136/bmjopen-2025-112164

from committee discussions will be most useful, but in the absence of this inferences from EAG reports about preferred analyses or interpretation may be required.

Extractions will include details on the location that each piece of information was identified to facilitate validation. Where multiple versions of an ITC are available across different documents (for example, an ITC performed within the company submission may have been adapted or amended by the EAG), methods and results relating to the committee's preferred analysis only will be extracted, which should be evident from the guidance documents. Where this information is uncertain, the EAG's preferred analysis will be preferentially extracted unless there is evidence from supporting information that the committee dismissed this analysis in favour of the company's original analysis. Details related to interpretation and decision making will be captured using quotation marks in extraction tables to ensure that detail is not lost during this process. Guidance documents and committee papers available on the NICE website will be prioritised for review during data extraction but review of other documents such as final scopes may be required if information remains unclear or if other documents with key information are identified. Any additional information provided by NICE will be considered where applicable.

5.4 Quality assessment

Given that the records to be included in this review are NICE technology appraisals rather than clinical trials such as randomised controlled trials (RCTs), non-randomised studies or qualitative studies, a quality assessment process will not be incorporated as part of this review.

5.5 Synthesis

Information extracted from included records will be summarised narratively and using summary tables, with similarities and differences across the identified CCEs noted. The narrative synthesis will focus mostly on the ways in which clinical similarity was confirmed or refuted, including:

- similarities and differences across appraisals with regards to methods used, such as specific thresholds that have been used and how they were derived (e.g. elicitation, arbitrary or other) or other methods of assessing clinical similarity;
- the rationale for the methods used;
- how common each method of assessing clinical similarity is;
- whether there are any differences in how similar methods or thresholds have been interpreted across appraisals.

Edwards SJ, *et al. BMJ Open* 2026; 16:e112164. doi: 10.1136/bmjopen-2025-112164

Any other key aspects identified throughout the review may also be covered. Specific CCEs may be highlighted as examples of different methods used to assess clinical similarity, such as use of MCIDs or other thresholds. Key strengths, limitations and uncertainties will be highlighted and summarised where appropriate.

6 Contribution of the research group:

Steve Edwards	Director of Health Technology Assessment, BMJ-TAG, London. Conception of the research project and provision of research framework. Guidance and validation of the work of the research group at all stages; provision of feedback on all versions of the protocol and the report. Guarantor of the report.
Clare Dadswell	Clinical Evidence Manager, BMJ-TAG, London. Contribution to the development of the project. Providing feedback to resolve any outstanding conflict between the first two reviewers on the systematic literature review.
Nicole Downes	Senior Clinical Evidence Analyst, BMJ-TAG, London. Lead on the development of this protocol. Lead reviewer on the systematic literature review of NICE guidance, including development of search strategy, running searches, sifting of results, data extraction and narrative synthesis of results. Drafting sections of the final report related to the systematic literature review.
Ben Burgess	Senior Clinical Evidence Analyst, BMJ-TAG, London. Leading on the development of a Bayesian framework for a formal assessment of clinical similarity. Testing the developed framework using examples identified through the systematic literature review. Drafting sections of the final report related to the Bayesian framework.
Archie Walters	Health Economist, BMJ-TAG, London. Second reviewer on the systematic literature review of NICE guidance, involved in validation

	of the screening stage and validation of a proportion of the extractions.
Sophie Ip	Senior Health Economist, BMJ-TAG, London. Supporting during the Bayesian framework development stage by validating code and analyses.

7 Timetable/Milestones

Key dates for this project are outlined in Table 1 below and are mostly related to the systematic literature review (SLR) phase of this research.

Table 1 Draft milestones table

Milestone	Start date	End date
Project start date	07/04/2025	07/04/2025
Draft protocol development	07/04/2025	11/04/2025
SLR searching, sifting, extraction and validation	14/04/2025	02/05/2025
SLR synthesis and report writing	06/05/2025	06/06/2025
Work on Bayesian framework including report writing	07/04/2025	18/06/2025
Project submission	19/06/2025	19/06/2025

8 Appendices

8.1 Draft data extraction form

Appraisal	Appraisal name (ID) {reference}
Appraisal details	**Comparator(s) included:**<br>• **ITC required for:**<br><br>**Source:**
	**Outcomes included in the ITCs:**<br><br>**Outcome(s) considered to be primary outcomes by reviewer:**<br>•<br>•<br>•<br><br>(defined as primary outcomes…)<br><br>**Outcome scales and thresholds:**<br>•

<table>
<tr><td></td><td>Source:</td></tr>
<tr><td></td><td>Final appraisal decision:

Source:</td></tr>
<tr><td>Details of committee's preferred ITCs</td><td>Preferred ITCs:

Source:</td></tr>
<tr><td rowspan="3">ITC results – committee's preferred analyses</td><td>Comparison 1 -
Outcome 1
Effect estimate from ITC: (e.g. RR and 95% CI)
Statistical significance from ITC:
Source:
Availability of ITC input data for replication:
Outcome 2
Effect estimate from ITC:
Statistical significance from ITC:
Source:
Availability of ITC input data for replication:
Outcome 3
Effect estimate from ITC:
Statistical significance from ITC:
Source:
Availability of ITC input data for replication:</td></tr>
<tr><td>Comparison 2
Outcome 1
Effect estimate from ITC:
Statistical significance from ITC:
Source:
Availability of ITC input data for replication:</td></tr>
<tr><td>Comparison 3
Outcome 1
Effect estimate from ITC:
Statistical significance from ITC:
Source:
Availability of ITC input data for replication:</td></tr>
</table>

Edwards SJ, *et al. BMJ Open* 2026; 16:e112164. doi: 10.1136/bmjopen-2025-112164

Methods used to assess clinical similarity and conclusions	***Company approach***<br>• **Method used and rationale:**<br>• **Details of thresholds used:**<br>• **Conclusions for each outcome:**<br>• **Uncertainty in the ITC results and impact on decision-making:**<br>• **Comments:**<br>• **Source:**
	EAG approach<br>• **Method used and rationale:**<br>• **Details of thresholds used:**<br>• **Conclusions for each outcome:**<br>• **Uncertainty in the ITC results and impact on decision-making:**<br>• **Comments:**<br>• **Source:**
	Committee approach<br>• **Method used and rationale:**<br>• **Details of thresholds used:**<br>• **Conclusions for each outcome:**<br>• **Uncertainty in the ITC results and impact on decision-making:**<br>• **Comments:**<br>• **Source:**
Comments	
Abbreviations:	

9 References

1. National Institute for Health and Care Excellence (NICE). NICE health technology evaluations: the manual, 2023. Available from: https://www.nice.org.uk/process/pmg36. Date accessed: Apr 25.
2. National Institute for Health and Care Excellence (NICE). Technology appraisal methods and processes used before 2022, 2022. Available from: https://www.nice.org.uk/about/what-we-do/our-programmes/nice-guidance/nice-technology-appraisal-guidance/process. Date accessed: Apr 25.
3. National Institute for Health and Care Excellence (NICE). Taking a proportionate approach to technology appraisals, 2024. Available from: https://www.nice.org.uk/about/what-we-do/proportionate-approach-to-technology-appraisals. Date accessed: Apr 25.
4. Walker E, Nowacki AS. Understanding equivalence and noninferiority testing. *J Gen Intern Med* 2011; **26**: 192-6.
5. Klukowska AM, Vandertop WP, Schröder ML, Staartjes VE. Calculation of the minimum clinically important difference (MCID) using different methodologies: case study and practical guide. *Eur Spine J* 2024; **33**: 3388-400.
6. Page MJ, McKenzie JE, Bossuyt PM, Boutron I, Hoffmann TC, Mulrow CD, et al. The PRISMA 2020 statement: an updated guideline for reporting systematic reviews. *BMJ* 2021; **372**: n71
