# Supplementary material for: Clinical similarity in cost-comparison evaluations: a systematic review of current methods in NICE appraisals and the development of a framework for the formal assessment of clinical similarity
Source: BMJ Open. 2026 Jul 21;16(7):e112164. doi: 10.1136/bmjopen-2025-112164 (PMC13410701; doi:10.1136/bmjopen-2025-112164)
Supplement: Supplementary data [file bmjopen-16-7-s002.pdf]

Online Supplementary File 2

Systematic Literature Review (SLR) methods

Inclusion and exclusion criteria

This review aimed to include National Institute for Health and Care Excellence (NICE) cost-comparison evaluations (CCEs) from 2017 onwards that followed the CCE approach from the outset, relied on results from indirect treatment comparisons (ITCs) that were not statistically significant for at least one comparator for the primary outcome(s) and had published final guidance before completion of the review. Appraisals that had direct randomised controlled trial (RCT) evidence or statistically significant ITC results for one comparator but relied on the non-significant results of an ITC for at least one other comparator were considered for inclusion providing there were clear statements from the External Assessment Group (EAG) and/or committee about where clinical similarity can be concluded for comparators relying on the latter.

Appraisals where the results from ITCs were all statistically significant were excluded given there is less uncertainty associated with these results; however, it is possible that some appraisals that were based solely on statistically significant ITC results have been included in the review, given it was sometimes difficult to decipher due to redaction of the results and discussion of statistical significance in the published NICE papers. Where it was clear that most emphasis was placed on specific outcomes for decision-making, these appraisals were excluded if the ITC results for these outcomes were statistically significant, even if there were some non-significant differences for other outcomes that were not key to decision-making. This was a pragmatic decision to try to identify appraisals where decisions were made based on the most uncertainty from ITCs, given that the presence of statistically significant differences for the key outcomes is likely to outweigh any more uncertain results for outcomes considered less important as part of the decision-making process. Only two appraisals were excluded for this reason.

In the protocol for this review, the researchers noted that appraisals that originated as a CCE, i.e. where the company's first submission was a CCE, would be prioritised for inclusion. It was also stated that appraisals that started with cost-utility models but later incorporated cost-comparison approaches would be considered for inclusion if it was clear that decision-making was solely based on the cost-comparison evidence but only if time permitted; given that 41 appraisals with a CCE approach as its first submission were

identified, there was insufficient resource available to consider inclusion of appraisals that adopted a CCE approach later in the process.

Definition of primary outcomes in this SLR

In line with the review protocol, reviewers only extracted information relating to outcomes that were considered to be primary outcomes for each appraisal. NICE papers did not often outline which outcomes were primary outcomes for decision-making purposes, but in the absence of this being explicitly stated, reviewers often based this decision on which were primary outcomes in the key trials of the intervention in each appraisal or which were noted in the submissions as being key for economic models of prior appraisals in the same area. While this may have led to some inconsistencies in terms of which outcomes were extracted as primary outcomes for different appraisals in the same or similar disease area, the researchers note that all general statements relating to how companies, EAGs and committees interpreted results and made conclusions on clinical similarity overall within the appraisal have been extracted, so key information relating to methods for concluding clinical similarity should not have been lost. Adverse events were not included as primary outcomes for any appraisals as in the researchers' experience there are usually very limited data on which to base conclusions about comparability of safety profiles between treatments within ITCs, given there are commonly few events and substantial uncertainty, and in many cases ITCs are not performed for safety outcomes for these reasons, with conclusions about these outcomes likely to be based more heavily on supplementary information such as feedback from clinical experts.

Data extraction

Identifying and summarising information about clinical similarity conclusions and the rationale or methods to make these conclusions was a key aim of this systematic literature review (SLR) and the following were extracted from included appraisals to allow approaches and patterns to be identified and discussed:

- Any mention of ways of interpreting ITC results such as specific minimal clinically important difference (MCID) thresholds or other thresholds and how these were identified;
- Results of the committee's preferred ITCs for relevant comparisons, including point estimates and measures of uncertainty (such as 95% confidence or credible intervals) of the effect estimates;

- Any committee or EAG comments on the uncertainty in the ITC results, including what they thought about the uncertainty in the ITC results and how it may have impacted their decision or conclusions;
- Narrative comments within company submissions, EAG reports or committee discussion documents describing how results of ITCs were ultimately interpreted and how clinical similarity was confirmed or refuted in the final decision-making process; clear statements from committee discussions were considered most useful, but were often not available, so often inferences from EAG reports about preferred analyses or interpretation were required.

Where multiple versions of an ITC were available across different documents (for example, an ITC performed within the company submission may have been adapted or amended by the EAG), methods and results relating to the committee’s preferred analysis only were extracted where this was clear. In some cases, results of all analyses were discussed as a whole, in which case results of each analysis were extracted separately. Where the committee’s preferred analysis was unclear, the EAG’s preferred analysis was assumed to be the committee’s preferred analysis unless there was evidence from supporting information that the committee dismissed this analysis in favour of the company’s original analysis or that it considered the results of multiple analyses when making decisions.

Details related to interpretation and decision making were captured using quotation marks in extraction tables to ensure that detail was not lost during this process. Guidance documents and committee papers available on the NICE website were prioritised for review during data extraction, with public committee slides also checked where available to identify any additional information. For appraisals where the first committee meeting included a CCE that was then rejected, only NICE papers related to the first committee meeting were reviewed, as the papers relating to the second committee meeting were not likely to provide any further information on the conclusions of the CCE stage. Review of other documents such as NICE final scopes was rarely required.

SLR results

Supplementary Figure 1. PRISMA flow diagram of records included in the SLR

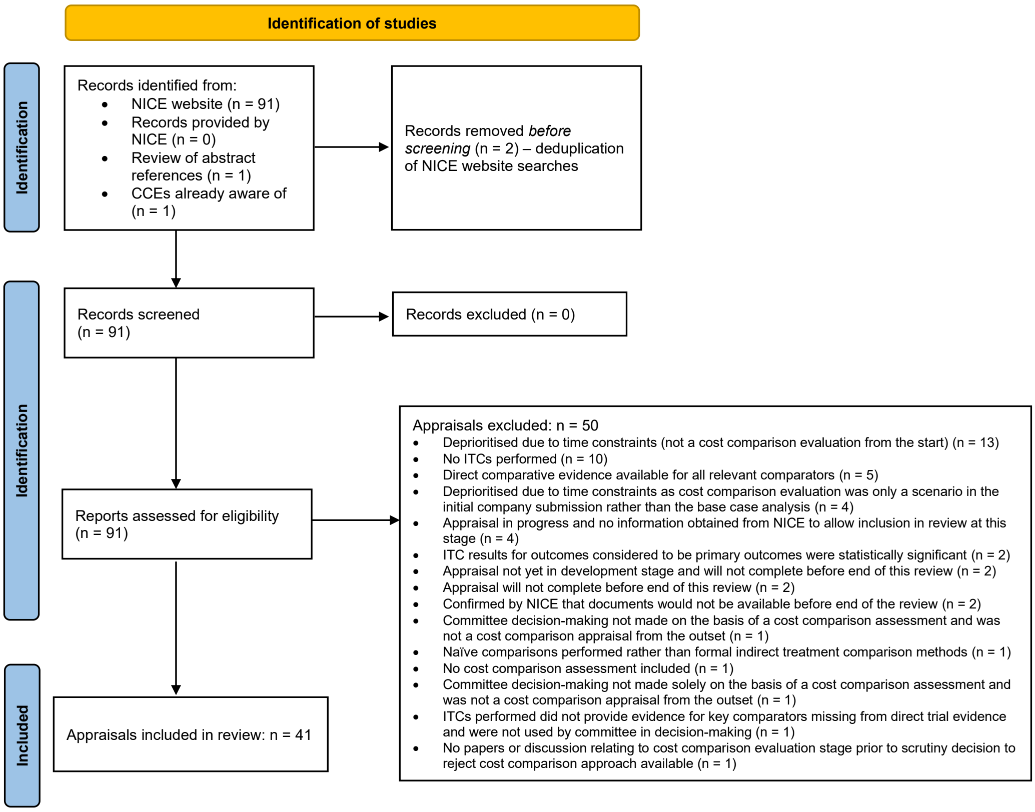

Abbreviations: CCE, cost-comparison evaluation; ITC, indirect treatment comparison; NICE, National Institute for Health and Care Excellence; PRISMA, Preferred Reporting Items for Systematic Reviews and Meta-Analyses; SLR, systematic literature review.

Supplementary Table 1. Summary of NICE technology appraisals included in the SLR

Appraisal name (ID)	Disease area	Date published	ITC outcomes considered primary in this review	Other ITC outcomes	Comparators with only ITC evidence	Statistically significant ITC results for primary outcomes?	Comparators with direct evidence (superiority or non-inferiority)	Type of ITCs
Aflibercept for treating choroidal neovascularisation (TA486)[1]	Choroidal neovascularisation	November 2017	Gain in BCVA from baseline	None	Ranibizumab	None	None	NMAs
Golimumab for treating non-radiographic axial spondyloarthritis (TA497)[2]	Axial spondyloarthritis	January 2018	ASAS20; ASAS40; BASDAI50	CFB in BASFI; CFB in BASDAI; CFB in BASMI; AEs; serious AEs; infections	Adalimumab; etanercept; certolizumab pegol	None	None	NMAs
Guselkumab for treating moderate to severe plaque psoriasis (TA521)[3]	Plaque psoriasis	June 2018	PASI 75 response	PASI 50 response; PASI 90 response; PASI 100 response; PGA/IGA; HRQoL; AEs; serious AEs; withdrawal due to AEs	Secukinumab; ixekizumab; etanercept; infliximab<br><br>(first two considered key by committee and included here)	Statistically significant for comparison against 300 mg secukinumab 300 mg but not against 80 mg ixekizumab	Adalimumab (2 superiority trials); ustekinumab (1 superiority trial)	NMAs
Ertugliflozin as monotherapy or with metformin for treating type 2 diabetes (TA572)[4]	Type 2 diabetes	March 2019	% CFB in HbA1c	% CFB in weight; % CFB in SBP; HbA1c <7.0%; AEs; UTIs	Canagliflozin; dapagliflozin; empagliflozin	Some doses of ertugliflozin reported to be superior to specific comparators, but most non-significant	None	NMAs

Ertugliflozin with metformin and a dipeptidyl peptidase-4 inhibitor for treating type 2 diabetes (TA583)[5]	Type 2 diabetes	June 2019	% CFB in HbA1c	% CFB in weight; % CFB in SBP; HbA1c <7.0%; AEs; UTIs	Canagliflozin; dapagliflozin; empagliflozin	Some significant differences noted vs dapagliflozin when one trial considered but not in other analyses	None	NMAs
Risankizumab for treating moderate to severe plaque psoriasis (TA596)[6]	Plaque psoriasis	August 2019	PASI 90 response; PASI 75 response	PASI 50 response; PASI 100 response; DLQI 0/1; AEs; serious AEs; withdrawal due to AEs	Guselkumab	Likely all non-significant but unclear	Adalimumab (1 superiority trial); ustekinumab (2 superiority trials)	NMAs
Brolucizumab for treating wet age-related macular degeneration (TA672)[7]	Age-related macular degeneration	February 2021	Mean CFB in BCVA	CFB in injection frequency; CFB in CRT; gain ≥15 ETDRS letters; loss ≥15 ETDRS letters; AEs; treatment discontinuation	Ranibizumab	None	Aflibercept (2 non-inferiority trials)	NMAs
Acalabrutinib for treating chronic lymphocytic leukaemia (TA689)[8]	Chronic lymphocytic leukaemia	April 2021	OS; PFS	AEs	Ibrutinib	None	None	MAICs
Bimekizumab for treating moderate to severe plaque psoriasis (TA723)[9]	Plaque psoriasis	September 2021	PASI 90 response; PASI 75 response	PASI 50 response; PASI 100 response; AEs; serious AEs; AEs leading to discontinuation	Brodalumab; Risankizumab; ixekizumab	Possibly some statistically significant differences but unclear	Adalimumab (1 superiority trial); secukinumab (1 superiority trial); Ustekinumab (1 superiority trial)	NMAs
Secukinumab for treating moderate to severe plaque	Plaque psoriasis	October 2021	PASI 75 response	PASI 50 response; PASI 90 response;	Ustekinumab (adalimumab could not be	None	Etanercept (2 superiority trials)	NMAs

psoriasis in children and young people (TA734)[10]				PASI 100 response; mean CFB in CDLQI	included in the ITC)			
Tofacitinib for treating juvenile idiopathic arthritis (TA735)[11]	Juvenile idiopathic arthritis	October 2021	Disease flares; ACR Pedi responses (various thresholds)	URTI	Adalimumab; tocilizumab (etanercept could not be included in the ITC)	None	None	Bucher ITCs
Empagliflozin for treating chronic heart failure with reduced ejection fraction (TA773)[12]	Heart failure	March 2022	CV death or HHF composite	First HHF; total HHF; KCCQ; worsening renal function; CV death; all-cause mortality	Dapagliflozin	None	None	Bucher ITCs
Faricimab for treating diabetic macular oedema (TA799)[13]	Diabetic macular oedema	June 2022	Mean CFB in BCVA	Mean number of administration injections; CFB in CST; gain of ≥10/15 ETDRS letters; loss of ≥10/15 ETDRS letters; all-cause discontinuation; ocular AEs	Ranibizumab	Unclear – possibly statistically significant in overall population but not in ≥400 µm subgroup relevant to assessment	Aflibercept (4 non-inferiority trials)	NMAs
Faricimab for treating wet age-related macular degeneration (TA800)[14]	Age-related macular degeneration	June 2022	Mean CFB in BCVA	Mean number of injections; mean number of administration injections; mean CFB in CST; gain of ≥10/15 BCVA letters; ETDRS	Ranibizumab	Unclear	Aflibercept (4 non-inferiority trials)	NMAs

				letters categories; AEs; discontinuation				
Risankizumab for treating active psoriatic arthritis after inadequate response to DMARDs (TA803)[15]	Psoriatic arthritis	July 2022	ACR 20; PASI 75 response	PsARC response; ACR 50; ACR 70; PASI 50 response; PASI 90 response; PASI 100 response; CFB in HAQ-DI; AEs, serious AEs; AEs leading to discontinuation	Guselkumab	None	None	NMAs
Brolucizumab for treating diabetic macular oedema (TA820)[16]	Diabetic macular oedema	August 2022	CFB in BCVA	BCVA categorical analyses; CFB in DRSS; CFB in retinal thickness; study discontinuation; serious ocular AEs; serious non-ocular AEs	Ranibizumab	None when EAG-preferred ITCs considered (statistically significant for company's preferred analysis)	Aflibercept	NMAs
Upadacitinib for treating active ankylosing spondylitis (TA829)[17]	Ankylosing spondylitis	September 2022	ASAS 40; BASDAI 50	CFB in BASDAI; CFB in BASFI; ASAS 20; ASAS PR; CFB in total back pain score	Ixekizumab; secukinumab	None	None	NMAs
Palbociclib with fulvestrant for treating hormone receptor-positive, HER2-negative advanced breast cancer after endocrine therapy (TA836)[18]	Breast cancer	October 2022	OS; PFS	None	Abemaciclib + fulvestrant; ribociclib + fulvestrant	Unclear but likely non-significant	None	MAICs and Bucher ITCs also presented

Cabozantinib for previously treated advanced hepatocellular carcinoma (TA849)[19]	Hepatocellular carcinoma	December 2022	OS; PFS	Treatment-emergent AEs with a grade 3/4 occurring in ≥5% of patients in either arm	Regorafenib	None	None	Bucher ITCs and MAICs presented
Upadacitinib for treating active non-radiographic axial spondyloarthritis (TA861)[20]	Axial spondyloarthritis	February 2023	ASAS 40	BASDAI 50; CFB in BASDAI50; CFB in BASFI; ASAS 20; ASAS PR; patient assessment of CFB in total back pain	Secukinumab; ixekizumab	None	None	NMAs
Upadacitinib for previously treated moderately to severely active Crohn’s disease (TA905)[21]	Crohn’s disease	June 2023	CDAI clinical remission; CDAI clinical response	Serious AEs; discontinuation due to AEs	Ustekinumab; vedolizumab	Possibly statistically significant results for clinical remission outcomes but not clinical response outcomes, but unclear	None	NMAs
Bimekizumab for treating axial spondyloarthritis (TA918)[22]	Axial spondyloarthritis	October 2023	ASAS 40; BASDAI 50	ASAS 20; ASAS S5/6; ASAS PR; ASDAS <2.1; ASDAS-CII; ASDAS ID; ASDAS MI; BASDAI 50; ASDAS-CRP; ASQoL; BASDAI; BASFI; BASMI; fatigue NRS; MASES; NSP; PhGADA; SF-36 MCS; SF-36 PCS; all-cause	Secukinumab; ixekizumab	None	None	NMAs

				discontinuation; discontinuation due to AEs; serious AEs				
Tofacitinib for treating active ankylosing spondylitis (TA920)[23]	Ankylosing spondylitis	October 2023	ASAS 20	ASAS 40; BASDAI 50; BASDAI; BASFI; ASDAS; ASQoL; SF-36 PCS; AE-related discontinuation	Secukinumab; ixekizumab	None	None	NMAs
Mirikizumab for treating moderately to severely active ulcerative colitis (TA925)[24]	Ulcerative colitis	October 2023	Clinical response; clinical remission	Mucosal healing; all-cause discontinuation; serious AEs	Vedolizumab; ustekinumab	Possibly some statistically significant results for certain populations but most likely non-significant, unclear	None	NMAs
Empagliflozin for treating chronic heart failure with preserved or mildly reduced ejection fraction (TA929)[25]	Heart failure	November 2023	CV mortality or HHF composite	HHF; CV mortality; all-cause mortality	Dapagliflozin	None	None	Bucher ITCs
Zanubrutinib for treating chronic lymphocytic leukaemia (TA931)[26]	Chronic lymphocytic leukaemia	November 2023	OS; PFS	None	Acalabrutinib; VenR (ibrutinib was naïve comparison)	None	None	MAIC (acalabrutinib) and NMA (VenR, performed by EAG)
Empagliflozin for treating chronic kidney disease (TA942)[27]	Chronic kidney disease	December 2023	Composite renal outcome (using various thresholds)	Progression to ESKD/ESRD; HHF; CV death; HHF or CV death	Dapagliflozin	None	None	NMAs and MAICs

				composite; 3P-MACE+; all-cause mortality; all-cause hospitalisations				
Fluocinolone acetonide intravitreal implant for treating chronic diabetic macular oedema (TA953)[28]	Chronic diabetic macular oedema	March 2024	≥15-letter BCVA improvement	Mean CFB in BCVA letter score; mean CFB in CRT; serious ocular AEs; intraocular pressure-related AEs; cataract-related AEs	Dexamethasone intravitreal implant	None	None	Bucher ITCs and MAICs
Etrasimod for treating moderately to severely active ulcerative colitis in people aged 16 and over (TA956)[29]	Ulcerative colitis	March 2024	Clinical response; clinical remission	Serious infections during induction phase of treatment	Adalimumab, infliximab; vedolizumab (others listed but not focused on)	Statistically significant for comparison against adalimumab but not infliximab or vedolizumab	None	NMAs
Linzagolix for treating moderate to severe symptoms of uterine fibroids (TA996)[30]	Uterine fibroids	August 2024	Response (MBL ≤80 ml and ≥50% reduction from baseline)	% change in MBL; pain improvement; % change in primary fibroid volume; % change in haemoglobin; CFB in HRQoL	Relugolix CT	None	None	NMAs
Risankizumab for treating moderately to severely active ulcerative colitis (TA998)[31]	Ulcerative colitis	August 2024	Clinical response; clinical remission	Endoscopic improvement; serious infections; serious AEs	Ustekinumab	None	None	NMAs
Vibegron for treating symptoms	Overactive bladder syndrome	September 2024	Average number of daily	Total incidence of incontinence	Mirabegron	One statistically significant result	None	NMAs and Bucher ITCs

of overactive bladder syndrome (TA999)[32]			micturitions; number of UUI episodes	episodes; volume of urine per micturition; any AEs; serious AEs; AEs leading to study discontinuation; specific AEs (headache, hypertension, UTI, dry mouth and constipation)		for one comparison/time-point for number of UUI episodes but all others non-significant		
Faricimab for treating visual impairment caused by macular oedema after retinal vein occlusion (TA1004)[33]	Visual impairment caused by macular oedema after retinal vein occlusion	September 2024	Mean CFB in BCVA	Mean CFB in CST; categorical vision changes; serious ocular AEs; all-cause discontinuation	Ranibizumab	None	None	NMAs
Rucaparib for maintenance treatment of relapsed platinum-sensitive ovarian, fallopian tube or peritoneal cancer (TA1007)[34]	Ovarian, fallopian tube and peritoneal cancer	September 2024	PFS; OS	PFS for second event; time to start of subsequent therapy; time to treatment discontinuation	Niraparib; olaparib	Unclear, likely non-significant	None	NMAs and MAICs
Crovalimab for treating paroxysmal nocturnal haemoglobinuria in people 12 years and over (TA1019)[35]	Paroxysmal nocturnal haemoglobinuria	November 2024	Transfusion avoidance; breakthrough haemolysis	Haemoglobin stabilisation; number of packed red blood cell transfusions; FACIT fatigue score; AEs	Ravulizumab	None	Eculizumab (1 non-inferiority, 1 safety/tolerability study)	NMAs

Eplontersen for treating hereditary transthyretin-related amyloidosis (TA1020)[36]	Hereditary transthyretin-related amyloidosis	November 2024	Serum TTR % CFB; CFB in mNIS+7; CFB in Norfolk QoL-DN	Serum TTR absolute value and absolute CFB; response analyses for mNIS+7; responder analyses for Norfolk QoL-DN; CFB in mBMI; CFB in 10-MWT; CFB in R-ODS; serious AEs; severe AEs; treatment discontinuation	Vutrisiran	None	None	MAICs
Crizotinib for treating ROS1-positive advanced non-small-cell lung cancer (TA1021)[37]	Non-small-cell lung cancer	December 2024	OS; PFS; ORR	Duration of response	Entrectinib	Unclear but likely mostly non-significant	None	STCs and MAICs
Bevacizumab gamma for treating wet age-related macular degeneration (TA1022)[38]	Age-related macular degeneration	December 2024	Mean CFB in BCVA	Gain ≥15 BCVA letters; loss ≥15 BCVA letters; ocular AEs	Aflibercept; faricimab	None	Ranibizumab (2 superiority trials)	NMAs and MAICs
Ublituximab for treating relapsing multiple sclerosis (TA1025)[39]	Multiple sclerosis	December 2024	ARR	CDP-12; CDP-24; all-cause discontinuation	Ocrelizumab; ofatumumab	None	Teriflunomide (2 superiority trials)	NMAs
Olaparib for treating BRCA mutation-positive HER2-negative advanced breast	Breast cancer	February 2025	PFS; OS	ORR; any AEs; any serious AEs; any treatment-related serious AEs;	Talazoparib	None	None	NMAs

cancer after chemotherapy (TA1040)[40]				specific types of AEs				
Durvalumab with etoposide and either carboplatin or cisplatin for untreated extensive-stage small-cell lung cancer (TA1041)[41]	Small-cell lung cancer	February 2025	OS; PFS	Any AEs; specific AE types/severities	Atezolizumab	None	None	Bucher ITCs

Abbreviations: 3P-MACE, 3-point major adverse cardiovascular outcome; 10-MWT, 10-metre walk test; ACR, American College of Rheumatology; ACR Pedi, American College of Rheumatology Pediatric response criteria; AE, adverse event; ARR, annualised relapse rate; ASAS, Assessment of SpondyloArthritis international Society; ASAS-PR, Assessment of SpondyloArthritis International Society criteria partial remission; ASDAS, Ankylosing Spondylitis Disease Activity Score; ASDAS-CII, Ankylosing Spondylitis Disease Activity Score Clinically Important Improvement; ASDAS-CRP, Ankylosing Spondylitis Disease Activity Score with C-reactive protein; ASDAS-ID, Ankylosing Spondylitis Disease Activity Score Inactive Disease; ASDAS-MI, Ankylosing Spondylitis Disease Activity Score Major Improvement; ASQoL, Ankylosing Spondylitis Quality of Life; BASDAI, Bath Ankylosing Spondylitis Disease Activity Index; BASFI, Bath Ankylosing Spondylitis Functional Index; BASMI, Bath Ankylosing Spondylitis Metrology Index; BCVA, best corrected visual acuity; CFB, change from baseline; CDAI, Clinical Disease Activity Index; CDLQI, Children's Dermatology Life Quality Index; CDP, confirmed disability progression; CRT, central retinal thickness; CST, central subfield thickness; CV, cardiovascular; DLQI, Dermatology Life Quality Index; DRSS, Diabetic Retinopathy Severity Scale; EAG, External Assessment Group; ESKD/ESRD, end-stage kidney/renal disease; ETDRS, Early Treatment Diabetic Retinopathy Study; FACIT, Functional Assessment of Chronic Illness Therapy; HAQ-DI, Health Assessment Questionnaire Disability Index; HbA1c, haemoglobin A1C; HHF, hospitalisation for heart failure; HRQoL, health-related quality of life; ITC, indirect treatment comparison; KCCQ, Kansas City Cardiomyopathy Questionnaire; MAIC, matching-adjusted indirect comparison; MASES, Maastricht Ankylosing Spondylitis Enthesitis; MBL, menstrual blood loss; mBMI, modified body mass index; mNIS+7, Modified Neuropathy Impairment Score +7; NICE, National Institute for Health and Care Excellence; NMA, network-meta-analysis; NRS, numerical rating scale; Norfolk QoL-DN, Norfolk Quality of Life-Diabetic Neuropathy; NSP, nocturnal spine pain; ORR, overall response rate; OS, overall survival; PASI, Psoriasis Area and Severity Index; PCS, physical component summary; PFS, progression-free survival; PGA/IGA, Physician's Global Assessment Scale/Investigator's Global Assessment Scale; PhGADA, Physician’s Global Assessment of Disease Activity; PsARC, Psoriatic Arthritis Response Criteria; R-ODS, Rasch-built Overall Disability Scale; SBP, systolic blood pressure; SF-36, Short Form 36-Item Health Survey; SLR, systematic literature review; STC, simulated treatment comparison; TTR, transthyretin; URTI, upper respiratory tract infection; UTI urinary tract infection; VenR, Venetoclax-Rituximab; UUI, urgency urinary incontinence.

Supplementary Table 2. Appraisals excluded from the SLR

Appraisal (ID)	Reason for exclusion
Guidance on the use of capecitabine and tegafur with uracil for metastatic colorectal cancer (TA61)[42]	No ITCs performed
Frequency of application of topical corticosteroids for atopic eczema (TA81)[43]	No ITCs performed
Topotecan for the treatment of relapsed small-cell lung cancer (TA184)[44]	No ITCs performed
Capecitabine for the treatment of advanced gastric cancer (TA191)[45]	No ITCs performed
Dexamethasone intravitreal implant for the treatment of macular oedema secondary to retinal vein occlusion (TA229)[46]	Deprioritised due to time constraints (not a cost-comparison evaluation from the start)
Colistimethate sodium and tobramycin dry powders for inhalation for treating pseudomonas lung infection in cystic fibrosis (TA276)[47]	ITCs performed did not provide evidence for key comparators missing from direct trial evidence and were not used by committee in decision-making
Ciclosporin for treating dry eye disease that has not improved despite treatment with artificial tears (TA369)[48]	No ITCs performed
Dasatinib, nilotinib and high-dose imatinib for treating imatinib-resistant or intolerant chronic myeloid leukaemia (TA425)[49]	No ITCs performed
Dasatinib, nilotinib and imatinib for untreated chronic myeloid leukaemia (TA426)[50]	Deprioritised due to time constraints (not a cost-comparison evaluation from the start)
Everolimus for advanced renal cell carcinoma after previous treatment (TA432)[51]	No ITCs performed
Ustekinumab for moderately to severely active Crohn’s disease after previous treatment (TA456)[52]	Deprioritised due to time constraints as cost-comparison evaluation was only a scenario in the initial company submission rather than the base case analysis
Atezolizumab for treating locally advanced or metastatic non-small-cell lung cancer after chemotherapy (TA520)[53]	Deprioritised due to time constraints (not a cost-comparison evaluation from the start)
Pembrolizumab for treating relapsed or refractory classical Hodgkin lymphoma (TA540)[54]	No cost-comparison assessment included
Venetoclax with rituximab for previously treated chronic lymphocytic leukaemia (TA561)[55]	Deprioritised due to time constraints (not a cost-comparison evaluation from the start)
Encorafenib with binimetinib for unresectable or metastatic BRAF V600 mutation-positive melanoma (TA562)[56]	Committee decision-making not made solely on the basis of a cost-comparison assessment and was not a cost-comparison appraisal from the outset
Abemaciclib with an aromatase inhibitor for previously untreated, hormone receptor-positive, HER2-negative, locally advanced or metastatic breast cancer (TA563)[57]	Deprioritised due to time constraints (not a cost-comparison evaluation from the start)
Benralizumab for treating severe eosinophilic asthma (TA565)[58]	Deprioritised due to time constraints (not a cost-comparison evaluation from the start)

Ustekinumab for treating moderately to severely active ulcerative colitis (TA633)[59]	Deprioritised due to time constraints (not a cost-comparison evaluation from the start)
Brigatinib for ALK-positive advanced non-small-cell lung cancer that has not been previously treated with an ALK inhibitor (TA670)[60]	Deprioritised due to time constraints as cost-comparison evaluation was only a scenario in the initial company submission rather than the base case analysis
Mepolizumab for treating severe eosinophilic asthma (TA671)[61]	ITC results for outcomes considered to be primary outcomes were statistically significant
Anakinra for treating Still's disease (TA685)[62]	Deprioritised due to time constraints (not a cost-comparison evaluation from the start)
Selective internal radiation therapies for treating hepatocellular carcinoma (TA688)[63]	No ITCs performed
Atezolizumab monotherapy for untreated advanced non-small-cell lung cancer (TA705)[64]	Deprioritised due to time constraints as cost-comparison evaluation was only a scenario in the initial company submission rather than the base case analysis
Diroximel fumarate for treating relapsing–remitting multiple sclerosis (TA794)[65]	Direct comparative evidence available for all relevant comparators
Somatrogon for treating growth disturbance in children and young people aged 3 years and over (TA863)[66]	Direct comparative evidence available for all relevant comparators
Vutrisiran for treating hereditary transthyretin-related amyloidosis (TA868)[67]	Direct comparative evidence available for all relevant comparators
Eptinezumab for preventing migraine (TA871)[68]	Deprioritised due to time constraints as cost-comparison evaluation was only a scenario in the initial company submission rather than the base case analysis
Risankizumab for previously treated moderately to severely active Crohn's disease (TA888)[69]	Deprioritised due to time constraints (not a cost-comparison evaluation from the start)
Pegunigalsidase alfa for treating Fabry disease (TA915)[70]	No ITCs performed
Bimekizumab for treating active psoriatic arthritis (TA916)[71]	ITC results for outcomes considered to be primary outcomes were statistically significant
Momelotinib for treating myelofibrosis-related splenomegaly or symptoms (TA957)[72]	Direct comparative evidence available for all relevant comparators
Atogepant for preventing migraine (TA973)[73]	No papers or discussion relating to CCE stage prior to scrutiny decision to reject CCE available
Selective internal radiation therapy with QuiremSpheres for treating unresectable advanced hepatocellular carcinoma (TA985)[74]	Naïve comparisons performed rather than formal indirect treatment comparison methods
Tenecteplase for treating acute ischaemic stroke (TA990)[75]	Direct comparative evidence available for all relevant comparators
Trastuzumab deruxtecan for treating HER2-low metastatic or unresectable breast cancer after chemotherapy (TA992)[76]	No ITCs performed
Pembrolizumab with platinum- and fluoropyrimidine-based chemotherapy for untreated advanced HER2-negative gastric or gastro-oesophageal junction adenocarcinoma (TA997)[77]	Deprioritised due to time constraints (not a cost-comparison evaluation from the start)

Evinacumab for treating homozygous familial hypercholesterolaemia in people 12 years and over (TA1002)[78]	Deprioritised due to time constraints (not a cost-comparison evaluation from the start)
Futibatinib for previously treated advanced cholangiocarcinoma with FGFR2 fusion or rearrangement (TA1005)[79]	Committee decision-making not made on the basis of a cost-comparison assessment and was not a cost-comparison appraisal from the outset
Latanoprost–netarsudil for previously treated primary open-angle glaucoma or ocular hypertension (TA1009)[80]	Deprioritised due to time constraints (not a cost-comparison evaluation from the start)
Fenfluramine for treating seizures associated with Lennox–Gastaut syndrome in people 2 years and over (TA1050)[81]	Deprioritised due to time constraints (not a cost-comparison evaluation from the start)
Tislelizumab for treating unresectable advanced oesophageal squamous cell cancer after platinum-based chemotherapy (ID4070)[82]	Appraisal in progress and no information obtained from NICE to allow inclusion in review at this stage
Somapacitan for treating growth hormone deficiency in children (ID6178)[83]	Appraisal in progress and no information obtained from NICE to allow inclusion in review at this stage
Guselkumab for treating moderately to severely active ulcerative colitis (ID6237)[84]	Appraisal will not complete before end of this review
Guselkumab for previously treated moderately to severely active Crohn's disease (ID6238)[85]	Appraisal in progress and no information obtained from NICE to allow inclusion in review at this stage
Mirikizumab for treating moderately to severely active Crohn's disease (ID6244)[86]	Appraisal in progress and no information obtained from NICE to allow inclusion in review at this stage
Nivolumab as neoadjuvant (with chemotherapy) and adjuvant (as monotherapy) treatment for resectable non-small-cell lung cancer (ID6310)[87]	Confirmed by NICE that documents would not be available before end of the review
Vanzacaftor–tezacaftor–deutivacaftor for treating cystic fibrosis with 1 or more F508del mutations in the CFTR gene in people aged 6 years and over (ID6372)[88]	Appraisal will not complete before end of this review
Abiraterone (originator and generics) for treating newly diagnosed high-risk hormone-sensitive metastatic prostate cancer (review of TA721) (ID6378)[89]	Appraisal not yet in development stage and will not complete before end of this review
Rilzabrutinib for treating persistent or chronic immune thrombocytopenia in people aged 12 and over (ID6395)[90]	Appraisal not yet in development stage and will not complete before end of this review
Darolutamide with androgen deprivation therapy for treating hormone-sensitive metastatic prostate cancer (ID6452)[91]	Confirmed by NICE that documents would not be available before end of the review

Abbreviations: CCE, cost-comparison evaluation; ITC, indirect treatment comparison; NICE, National Institute for Health and Care Excellence; SLR, systematic literature review.

Supplementary Table 3. Summary of methods used by companies to demonstrate clinical similarity

Method	Details	Appraisals
**Interpreting ITC results**		
Lack of statistically significant differences only	Relies solely on a lack of statistically significant differences only to interpret ITC results and conclude similarity.	**Three:** TA735, TA829 and TA1040.[11, 17, 40]
Lack of statistically significant differences and some discussion of point estimate direction and/or size	Relies mostly on a lack of statistically significant differences and direction and/or size of point estimates to interpret ITC results and conclude similarity.<br><br>Point estimates favouring the intervention over comparators are often noted, with those discussing point estimate size commenting on the difference being small and close to 0.0 or 1.0 (line of null effect).<br><br>Some refer to the fact that point estimates vary in direction for different outcomes, suggesting no consistent trend for a benefit of one treatment or the other as evidence of no difference.	**Fourteen:** TA689, TA773, TA849, TA861, TA929, TA931, TA942, TA953, TA998, TA1007, TA1020, TA1021, TA1025, TA1041[8, 19, 20, 25-28, 31, 34, 36, 37, 39, 41, 92]
Some statistically significant differences but none favouring the comparators	Some analyses with statistically significant differences are noted but none of these favour the comparator(s).	**Eight:** TA521, TA572, TA583, TA672, TA799, TA836, TA918, TA925[3-5, 7, 13, 18, 22, 24]
Size and direction of point estimates mentioned with no mention of lack of statistically significant differences	Size and direction of point estimates used to make conclusions of clinical similarity, with no emphasis placed on the lack of statistically significant differences.	**One:** TA803[15]
Some use of probabilities calculated from NMAs to interpret results	NMAs used to calculate probabilities of being best treatment, among best treatments, clinically equivalent or non-inferior, for example based on SUCRA values or incorporating equivalence or non-inferiority margins.<br><br>Often used alongside other methods of interpreting results such as highlighting a lack of statistically significant differences, point estimates close to 1.0 or 0.0 or point estimates favouring the intervention over the comparator(s).<br><br>In some cases they are mentioned but not focused on or are dismissed if not favouring the intervention.	**Six:** TA734, TA803, TA820, TA996, TA1019, TA1022[10, 15, 16, 30, 35, 38]

MCIDs or other thresholds used to support with interpretation of results	MCIDs or other thresholds used by the company in some way to interpret ITC results and conclude clinical similarity.<br><br>Often used alongside other methods of interpreting results such as highlighting a lack of statistically significant differences, point estimates close to 1.0 or 0.0 or point estimates favouring the intervention over the comparator(s).	**Four:** TA486, TA803, TA999, TA1019[1, 15, 32, 35]
Unclear what focus of decision-making was	Rationale for concluding similarity was unclear either because results and discussion sections were heavily redacted or because the rationale for conclusions of clinical similarity were not described.	**Ten:** TA497, TA596, TA723, TA734, TA800, TA820, TA905, TA920, TA956, TA1004[2, 6, 9, 10, 14, 16, 21, 23, 29, 33]
**Supplementary evidence**		
Similar mechanisms of action or pharmacodynamic profiles noted	Similarity in terms of mechanisms of action or pharmacodynamic profiles noted by the company as supportive evidence that the intervention is expected to be similar to the comparator(s).	**Six:** TA486, TA572, TA689, TA773, TA953, TA998[1, 4, 8, 28, 31, 92]
Clinical expert feedback	Clinical expert feedback stating that the treatments are expected to or have similar efficacy in their experience or clinical expert interpretation of ITC results as likely similar.	**Ten:** TA689, TA773, TA803, TA849, TA861, TA931, TA942, TA996, TA1040, TA1041[8, 15, 19, 20, 26, 27, 30, 40, 41, 92]
Reference to meta-analyses of intervention and comparator trials vs a common comparator as evidence of a class effect	Meta-analyses of comparator and intervention trials against a common comparator cited as evidence that there is a class effect, suggesting clinical similarity should be concluded.	**Three:** TA773, TA836, TA942[18, 27, 92]
Reference to previously published ITC results or NICE appraisals	Reference to other ITCs of the intervention and comparator(s) relevant to the appraisal within the literature or previous NICE appraisals as supportive evidence of clinical similarity given similar results were obtained.	**Four:** TA803, TA836, TA849, TA861[15, 18-20]
Reference to real-world data as supportive evidence	Reference to real-world data used as supportive evidence to ITCs evidence	**One:** TA1007[34]
Abbreviations: ITC, indirect treatment comparison; MCID, minimal clinically important difference; NICE, National Institute for Health and Care Excellence; NMA, network meta-analyses; SUCRA, Surface Under the Cumulative RAnking curve.		

Supplementary Table 4. Summary of methods used or comments made by EAGs in assessing clinical similarity

Method/comment	Details	Number of appraisals
**Interpretation of lack of statistically significant differences**		
Lack of statistically significant differences with some point estimates favouring the intervention used to make conclusions	Discussion of results and conclusions of clinical similarity focuses on the lack of statistically significant differences as well as at least some point estimates that favour the new intervention.	**Three:** TA829, TA953, TA1004[17, 28, 33]
Lack of statistically significant differences not completely dismissed but other methods of interpreting ITC results considered in making conclusions	Does not reject the use of lack of statistically significant differences as a method of making conclusions completely but incorporates other evidence in the decision-making, such as visual comparisons of adjusted KM curves, clinical expert feedback, similar mechanisms of action, decisions made in previous appraisals or additional data or analyses requested from the company or performed by the EAG itself.<br><br>While lack of statistically significant differences not rejected outright, EAGs sometimes highlight that it should be interpreted as no evidence of a difference between treatments rather than clinical similarity or equivalence conclusively.	**Twelve:** TA486, TA497, TA583, TA596, TA689, TA735, TA918, TA929, TA942, TA1007, TA1019, TA1041[1, 2, 5, 6, 8, 11, 22, 25, 27, 34, 35, 41]
Dismisses a lack of statistically significant differences as evidence for clinical similarity or equivalence	EAGs explicitly dismissed the idea that a lack of statistically significant differences is sufficient to conclude clinical similarity or equivalence, or it was otherwise suggested based on uncertainties discussed or requests made to the company.<br>In many cases this contributed to conclusions made by the EAG that there was insufficient evidence to support a CCE, but in some cases these uncertainties were addressed by consideration of other evidence and clinical similarity was concluded overall. For some appraisals, rejection of a CCE was likely not solely based on the lack of statistically significant differences and other factors such as concerns about trials included in the ITC or methodology may have contributed to this conclusion.	**Nineteen:** TA521, TA773, TA803, TA820, TA836, TA849, TA861, TA905, TA920, TA925, TA931, TA956, TA996, TA998, TA999, TA1020, TA1021, TA1025, TA1040[3, 15, 16, 18-21, 23, 24, 26, 29-32, 36, 37, 39, 40, 92]
Main rationale for conclusions of clinical similarity and position on lack of statistically significant differences unclear	EAG concludes that clinical similarity is likely to be a reasonable conclusion but the main factors for decision-making are unclear and there is no clear statement on the EAG's position regarding lack of statistically significant differences.	**Five:** TA572, TA672, TA723, TA800, TA1022[4, 7, 9, 14, 38]
**Formal methods of interpreting ITC results**		

Some use of probabilities calculated from NMAs to interpret results	NMAs used to calculate probabilities of being the best treatment, among the best treatments, clinically equivalent or non-inferior; for example, based on SUCRA values or incorporating equivalence or non-inferiority margins.<br><br>Reference to these data provided by companies was made to aid with the EAG's interpretation of results, there was a suggestion that these would have been useful if not provided or there was no criticism of the company's use of these data	**Nine:** TA497, TA734, TA803, TA820, TA996, TA1019, TA1022, TA1025, TA1040[2, 10, 15, 16, 30, 35, 38-40]
MCIDs or other thresholds used to support with interpretation of results	MCIDs or other thresholds used by the EAG in some way to interpret ITC results and conclude clinical similarity or mentioned as being potentially useful.<br><br>Based on the EAG making use of these put forward by the company or adapted versions, the EAG putting forward this approach itself or no criticism of the method having been used by the company	**Nine:** TA486, TA497, TA803, TA931, TA996, TA999, TA1019, TA1020, TA1040 [1, 2, 15, 26, 30, 32, 35, 36, 40]
**Supplementary evidence**		
Similar mechanisms of action or pharmacodynamic profiles noted	Similarity in terms of mechanisms of action or pharmacodynamic profiles noted by the EAG as supportive evidence that the intervention is expected to be similar to the comparator(s).	**Fourteen:** TA486, TA497, TA572, TA596, TA836, TA918, TA929, TA996, TA998, TA999, TA1007, TA1022, TA1025, TA1040[1, 2, 4, 6, 18, 22, 25, 30-32, 34, 38-40]
Clinical expert feedback	Clinical expert feedback stating that the treatments are expected to or have similar efficacy in their experience or clinical expert input to support with interpretation of ITC results, such as identification of MCIDs	**Fourteen:** TA497, TA689, TA723, TA836, TA929, TA931, TA956, TA998, TA999, TA1007, TA1020, TA1022, TA1025, TA1040[2, 8, 9, 18, 26, 29, 31, 32, 34, 36, 38-40, 93]
Other sources noted	Other sources of evidence used as supportive evidence of clinical similarity, including results of ITCs from previous NICE appraisals, statistical significance testing requested from companies, results from amended analyses to better align trials, visual review of adjusted KM curves from ITCs and naïve comparison of data.<br><br>Typically rejected meta-analyses of interventions and comparators to demonstrate class effect as supportive of clinical similarity when put forward by the company.	**Fourteen:** TA486, TA497, TA572, TA583, TA723, TA735, TA773, TA836, TA918, TA929, TA942, TA1007, TA1021, TA1041[1, 2, 4, 5, 9, 11, 18, 22, 25, 27, 34, 37, 41, 92]

Abbreviations: CCE, cost-comparison evaluation; EAG, External Assessment Group; ITC, indirect treatment comparison; KM, Kaplan-Meier; MCID, minimal clinically important difference; NICE, National Institute for Health and Care Excellence; NMA, network meta-analyses; SUCRA, Surface Under the Cumulative RAnking curve.

Supplementary Table 5. Summary of comments made by committees when making conclusions of clinical similarity.

Comment	Details	Number of appraisals
Discussion very limited, simply makes conclusions of clinical similarity	Only brief statement that results from ITCs were considered to indicate that intervention is likely to be similar to comparator(s), with no rationale or comments on uncertainty mentioned.	**Twenty-four:** TA572, TA583, TA596, TA672, TA723, TA734, TA735, TA800, TA836, TA905, TA918, TA925, TA942, TA953, TA998, TA999, TA1007, TA1019, TA1020, TA1021, TA1022, TA1025, TA1040, TA1041[4-7, 9-11, 14, 18, 21, 22, 24, 27, 28, 31, 32, 34-41]
Concludes similarity with uncertainty mentioned but no rationale as to how this was resolved	Notes uncertainties in ITC results but concludes clinical similarity likely overall with no discussion of how this uncertainty was addressed, why it was not considered a major issue or mention of other information that may have reduced uncertainty.	**Four:** TA521, TA799, TA920, TA929[3, 13, 23, 25]
Notes uncertainty in ITC results with clinical expert feedback considered before concluding clinical similarity	Uncertainty in ITC results noted but clinical expert feedback appears to have reduced concerns about this uncertainty to allow a conclusion of clinical similarity to be made.	**Seven:** TA486, TA689, TA820, TA829, TA849, TA861, TA905[1, 8, 16, 17, 19-21]
Notes uncertainty in ITC results with similarities in mechanisms of action considered before concluding clinical similarity	Uncertainty in ITC results noted but similarities in mechanisms of action may have reduced concerns about this uncertainty to allow a conclusion of clinical similarity to be made.	**Three:** TA773, TA803, TA956[9, 15, 29]
Notes uncertainty in ITC results but robustness of results to alternative analyses considered before concluding clinical similarity	Uncertainty in ITC results noted but the fact that results were robust to alternative analyses seems to have reduced some of the concern to allow a conclusion of clinical similarity to be made.	**One:** TA497[2]

Notes uncertainty in ITC results but point estimates being close to 1.0 or 0.0 considered in decision-making	Uncertainty in ITC results noted but the fact that point estimates were close to 1.0 or 0.0 seems to have reduced some of the concern to allow a conclusion of clinical similarity to be made.	**One:** TA803[15]
Committee concluded that results are too uncertain and cost-utility analyses required, or cost-utility analyses used despite conclusions of similarity from ITC results	Conclusion that there was too much uncertainty in the ITC results and cost-utility analyses were required for decision-making, or a conclusion of clinical similarity was made based on ITC results, but cost-utility analyses still appear to have been used for decision-making.	**Three:** TA849, TA931, TA996[19, 26, 30]
Cost-utility results available alongside cost-comparison analyses which may have contributed to decision-making	Conclusion of clinical similarity made but some cost-utility analyses were available alongside cost-comparison analyses, which may have reduced uncertainty in decision-making.	**One:** TA773[92]
Abbreviations: ITC, indirect treatment comparison.		

Supplementary Table 6. Additional findings from the SLR

Finding	Details
Additional detail regarding the committee's interpretation of statistically non-significant ITC results and consideration of supportive evidence may improve the transparency of decision-making.	In some appraisals, EAGs rejected the CCE given the uncertainty in the conclusions of clinical similarity. In some cases, the committee subsequently determined that there was sufficient evidence of clinical similarity.<br>In these cases, it was clear from most committee discussions that supportive evidence, such as clinical expert feedback, may have supported this decision, but there was rarely an explicit discussion of why these were considered to sufficiently alleviate the uncertainties raised by the EAG.
Guidance on whether or not a CCE within the NICE process needs to present a case against multiple or only one relevant comparator would be helpful, as there is clearly some uncertainty about this	EAG and committee discussions in some appraisals in this SLR suggest that demonstrating clinical similarity compared to one relevant comparator may be sufficient, including statements made by the committee in NICE TA1022 and by the EAG in NICE TA1007.[34, 38] In CCEs with more than one comparator, EAGs and committees have generally discussed the evidence for clinical similarity for all comparators separately rather than focusing on one with the strongest evidence.<br>A clear statement supporting the requirement for a case against only one comparator was not identified in the current NICE methods manual,[94] and an article by Costello Medical has recently expressed uncertainty with regards to this.[95] Therefore, clarity surrounding this requirement within NICE CCEs in the form of updated guidance may be useful for companies and EAGs.
Abbreviations: CCE, cost-comparison evaluation; EAG, External Assessment Group; ITC, indirect treatment comparison; NICE, National Institute for Health and Care Excellence; SLR, systematic literature review.	

References

1. National Institute for Health and Care Excellence (NICE). Aflibercept for treating choroidal neovascularisation - Technology appraisal guidance [TA486], 2017. Available from: https://www.nice.org.uk/guidance/ta486. Date accessed: May 25.

2. National Institute for Health and Care Excellence (NICE). Golimumab for treating non-radiographic axial spondyloarthritis - Technology appraisal guidance [TA497] 2018. Available from: https://www.nice.org.uk/guidance/ta497. Date accessed: May 25.

3. National Institute for Health and Care Excellence (NICE). Guselkumab for treating moderate to severe plaque psoriasis - Technology appraisal guidance [TA521], 2018. Available from: https://www.nice.org.uk/guidance/ta521. Date accessed: Apr 25.

4. National Institute for Health and Care Excellence (NICE). Ertugliflozin as monotherapy or with metformin for treating type 2 diabetes - Technology appraisal guidance [TA572], 2019. Available from: https://www.nice.org.uk/guidance/ta572. Date accessed: May 25.

5. National Institute for Health and Care Excellence (NICE). Ertugliflozin with metformin and a dipeptidyl peptidase-4 inhibitor for treating type 2 diabetes - Technology appraisal guidance [TA583], 2019. Available from: https://www.nice.org.uk/guidance/ta583. Date accessed: May 25.

6. National Institute for Health and Care Excellence (NICE). Risankizumab for treating moderate to severe plaque psoriasis - Technology appraisal guidance [TA596], 2019. Available from: https://www.nice.org.uk/guidance/ta596. Date accessed: Apr 25.

7. National Institute for Health and Care Excellence (NICE). Brolucizumab for treating wet age-related macular degeneration - Technology appraisal guidance [TA672], 2021. Available from: https://www.nice.org.uk/guidance/ta672. Date accessed: Apr 25.

8. National Institute for Health and Care Excellence (NICE). Acalabrutinib for treating chronic lymphocytic leukaemia - Technology appraisal guidance [TA689], 2021. Available from: https://www.nice.org.uk/guidance/ta689. Date accessed: Apr 25.

9. National Institute for Health and Care Excellence (NICE). Bimekizumab for treating moderate to severe plaque psoriasis - Technology appraisal guidance [TA723], 2021. Available from: https://www.nice.org.uk/guidance/ta723. Date accessed: Apr 25.

10. National Institute for Health and Care Excellence (NICE). Secukinumab for treating moderate to severe plaque psoriasis in children and young people - Technology appraisal guidance [TA734], 2021. Available from: https://www.nice.org.uk/guidance/ta734. Date accessed: Apr 25.

11. National Institute for Health and Care Excellence (NICE). Tofacitinib for treating juvenile idiopathic arthritis - Technology appraisal guidance [TA735], 2021. Available from: https://www.nice.org.uk/guidance/ta735. Date accessed: Apr 25.

12. National Institute for Health and Care Excellence (NICE). Empagliflozin for treating chronic heart failure with reduced ejection fraction - Technology appraisal guidance [TA773] - Committee papers, 2022. Available from: https://www.nice.org.uk/guidance/ta773/history. Date accessed: May 25.

13. National Institute for Health and Care Excellence (NICE). Faricimab for treating diabetic macular oedema - Technology appraisal guidance [TA799], 2022. Available from: https://www.nice.org.uk/guidance/ta799. Date accessed: Apr 25.

14. National Institute for Health and Care Excellence (NICE). Faricimab for treating wet age-related macular degeneration - Technology appraisal guidance [TA800], 2022. Available from: https://www.nice.org.uk/guidance/ta800. Date accessed: Apr 25.

15. National Institute for Health and Care Excellence (NICE). Risankizumab for treating active psoriatic arthritis after inadequate response to DMARDs - Technology appraisal guidance [TA803], 2022. Available from: https://www.nice.org.uk/guidance/ta803. Date accessed: Apr 25.

16. National Institute for Health and Care Excellence (NICE). Brolucizumab for treating diabetic macular oedema - Technology appraisal guidance [TA820], 2022. Available from: https://www.nice.org.uk/guidance/ta820. Date accessed: Apr 25.

17. National Institute for Health and Care Excellence (NICE). Upadacitinib for treating active ankylosing spondylitis - Technology appraisal guidance [TA829], 2022. Available from: https://www.nice.org.uk/guidance/ta829. Date accessed: Apr 25.

18. National Institute for Health and Care Excellence (NICE). Palbociclib with fulvestrant for treating hormone receptor-positive, HER2-negative advanced breast cancer after endocrine therapy - Technology appraisal guidance [TA836], 2022. Available from: https://www.nice.org.uk/guidance/ta836. Date accessed: Apr 25.

19. National Institute for Health and Care Excellence (NICE). Cabozantinib for previously treated advanced hepatocellular carcinoma - Technology appraisal giudance [TA849], 2022. Available from: https://www.nice.org.uk/guidance/ta849. Date accessed: May 25.

20. National Institute for Health and Care Excellence (NICE). Upadacitinib for treating active non-radiographic axial spondyloarthritis - Technology appraisal guidance [TA861], 2023. Available from: https://www.nice.org.uk/guidance/ta861. Date accessed: Apr 25.

21. National Institute for Health and Care Excellence (NICE). Upadacitinib for previously treated moderately to severely active Crohn's disease - Technology appraisal guidance [TA905], 2023. Available from: https://www.nice.org.uk/guidance/ta905. Date accessed: Apr 25.

22. National Institute for Health and Care Excellence (NICE). Bimekizumab for treating axial spondyloarthritis - Technology appraisal guidance [TA918], 2023. Available from: https://www.nice.org.uk/guidance/ta918. Date accessed: Apr 25.

23. National Institute for Health and Care Excellence (NICE). Tofacitinib for treating active ankylosing spondylitis - Technology appraisal guidance [TA920], 2023. Available from: https://www.nice.org.uk/guidance/ta920. Date accessed: Apr 25.

24. National Institute for Health and Care Excellence (NICE). Mirikizumab for treating moderately to severely active ulcerative colitis - Technology appraisal guidance [TA925], 2023. Available from: https://www.nice.org.uk/guidance/ta925. Date accessed: Apr 25.

25. National Institute for Health and Care Excellence (NICE). Empagliflozin for treating chronic heart failure with preserved or mildly reduced ejection fraction - Technology appraisal guidance [TA929], 2023. Available from: https://www.nice.org.uk/guidance/ta929. Date accessed: Apr 25.

26. National Institute for Health and Care Excellence (NICE). Zanubrutinib for treating chronic lymphocytic leukaemia - Technology appraisal guidance [TA931], 2023. Available from: https://www.nice.org.uk/guidance/ta931. Date accessed: May 25.

27. National Institute for Health and Care Excellence (NICE). Empagliflozin for treating chronic kidney disease - Technology appraisal guidance [TA942], 2023. Available from: https://www.nice.org.uk/guidance/ta942. Date accessed: Apr 25.

28. National Institute for Health and Care Excellence (NICE). Fluocinolone acetonide intravitreal implant for treating chronic diabetic macular oedema - Technology appraisal guidance [TA953], 2024. Available from: https://www.nice.org.uk/guidance/ta953. Date accessed: Apr 25.

29. National Institute for Health and Care Excellence (NICE). Etrasimod for treating moderately to severely active ulcerative colitis in people aged 16 and over - Technology appraisal guidance [TA956], 2024. Available from: https://www.nice.org.uk/guidance/ta956. Date accessed: Apr 25.

30. National Institute for Health and Care Excellence (NICE). Linzagolix for treating moderate to severe symptoms of uterine fibroids - Technology appraisal guidance [TA996], 2024. Available from: https://www.nice.org.uk/guidance/ta996. Date accessed: Apr 25.

31. National Institute for Health and Care Excellence (NICE). Risankizumab for treating moderately to severely active ulcerative colitis - Technology appraisal guidance [TA998], 2024. Available from: https://www.nice.org.uk/guidance/ta998. Date accessed: Apr 25.

32. National Institute for Health and Care Excellence (NICE). Vibegron for treating symptoms of overactive bladder syndrome - Technology appraisal guidance [TA999], 2024. Available from: https://www.nice.org.uk/guidance/ta999. Date accessed: Apr 25.

33. National Institute for Health and Care Excellence (NICE). Faricimab for treating visual impairment caused by macular oedema after retinal vein occlusion - Technology appraisal guidance [TA1004], 2024. Available from: https://www.nice.org.uk/guidance/ta1004. Date accessed: Apr 25.

34. National Institute for Health and Care Excellence (NICE). Rucaparib for maintenance treatment of relapsed platinum-sensitive ovarian, fallopian tube or peritoneal cancer - Technology appraisal guidance [TA1007], 2024. Available from: https://www.nice.org.uk/guidance/ta1007. Date accessed: Apr 25.

35. National Institute for Health and Care Excellence (NICE). Crovalimab for treating paroxysmal nocturnal haemoglobinuria in people 12 years and over - Technology appraisal guidance [TA1019], 2024. Available from: https://www.nice.org.uk/guidance/ta1019. Date accessed: Apr 25.

36. National Institute for Health and Care Excellence (NICE). Eplontersen for treating hereditary transthyretin-related amyloidosis - Technology appraisal guidance [TA1020], 2024. Available from: https://www.nice.org.uk/guidance/ta1020. Date accessed: Apr 25.

37. National Institute for Health and Care Excellence (NICE). Crizotinib for treating ROS1-positive advanced non-small-cell lung cancer - Technology appraisal guidance [TA1021], 2024. Available from: https://www.nice.org.uk/guidance/ta1021. Date accessed: Apr 25.

38. National Institute for Health and Care Excellence (NICE). Bevacizumab gamma for treating wet age-related macular degeneration - Technology appraisal guidance [TA1022], 2024. Available from: https://www.nice.org.uk/guidance/ta1022. Date accessed: Apr 25.

39. National Institute for Health and Care Excellence (NICE). Ublituximab for treating relapsing multiple sclerosis - Technology appraisal guidance [TA1025], 2024. Available from: https://www.nice.org.uk/guidance/ta1025. Date accessed: Apr 25.

40. National Institute for Health and Care Excellence (NICE). Olaparib for treating BRCA mutation-positive HER2-negative advanced breast cancer after chemotherapy - Technology appraisal guidance [TA1040], 2025. Available from: https://www.nice.org.uk/guidance/ta1040. Date accessed: Apr 25.

41. National Institute for Health and Care Excellence (NICE). Durvalumab with etoposide and either carboplatin or cisplatin for untreated extensive-stage small-cell lung cancer - Technology appraisal guidance [TA1041], 2025. Available from: https://www.nice.org.uk/guidance/ta1041. Date accessed: Apr 25.

42. National Institute for Health and Care Excellence (NICE). Guidance on the use of capecitabine and tegafur with uracil for metastatic colorectal cancer - Technology appraisal guidance [TA61], 2003. Available from: https://www.nice.org.uk/guidance/ta61. Date accessed: May 25.

43. National Institute for Health and Care Excellence (NICE). Frequency of application of topical corticosteroids for atopic eczema - Technology appraisal guidance [TA81], 2004. Available from: https://www.nice.org.uk/guidance/ta81. Date accessed: May 25.

44. National Institute for Health and Care Excellence (NICE). Topotecan for the treatment of relapsed small-cell lung cancer - Technology appraisal guidance [TA184], 2009. Available from: https://www.nice.org.uk/guidance/ta184. Date accessed: May 25.

45. National Institute for Health and Care Excellence (NICE). Capecitabine for the treatment of advanced gastric cancer - Technology appraisal guidance [TA191], 2010. Available from: https://www.nice.org.uk/guidance/ta191. Date accessed: May 25.

46. National Institute for Health and Care Excellence (NICE). Dexamethasone intravitreal implant for the treatment of macular oedema secondary to retinal vein occlusion - Technology appraisal guidance [TA229], 2011. Available from: https://www.nice.org.uk/guidance/ta229. Date accessed: May 25.

47. National Institute for Health and Care Excellence (NICE). Colistimethate sodium and tobramycin dry powders for inhalation for treating pseudomonas lung infection in cystic fibrosis - Technology appraisal guidance [TA276], 2013. Available from: https://www.nice.org.uk/guidance/ta276. Date accessed: May 25.

48. National Institute for Health and Care Excellence (NICE). Ciclosporin for treating dry eye disease that has not improved despite treatment with artificial tears - Technology appraisal guidance [TA369], 2015. Available from: https://www.nice.org.uk/guidance/ta369. Date accessed: May 25.

49. National Institute for Health and Care Excellence (NICE). Dasatinib, nilotinib and high-dose imatinib for treating imatinib-resistant or intolerant chronic myeloid leukaemia - Technology appraisal guidance [TA425], 2016. Available from: https://www.nice.org.uk/guidance/ta425. Date accessed: May 25.

50. National Institute for Health and Care Excellence (NICE). Dasatinib, nilotinib and imatinib for untreated chronic myeloid leukaemia - Technology appraisal guidance [TA426], 2016. Available from: https://www.nice.org.uk/guidance/ta426. Date accessed: May 25.

51. National Institute for Health and Care Excellence (NICE). Everolimus for advanced renal cell carcinoma after previous treatment - Technology appraisal guidance [TA432], 2017. Available from: https://www.nice.org.uk/guidance/ta432. Date accessed: May 25.

52. National Institute for Health and Care Excellence (NICE). Ustekinumab for moderately to severely active Crohn's disease after previous treatment - Technology appraisal guidance [TA456], 2017. Available from: https://www.nice.org.uk/guidance/ta456. Date accessed: May 25.

53. National Institute for Health and Care Excellence (NICE). Atezolizumab for treating locally advanced or metastatic non-small-cell lung cancer after chemotherapy - Technology appraisal guidance [TA520], 2018. Available from: https://www.nice.org.uk/guidance/ta520. Date accessed: May 25.

54. National Institute for Health and Care Excellence (NICE). Pembrolizumab for treating relapsed or refractory classical Hodgkin lymphoma - Technology appraisal guidance [TA540], 2018. Available from: https://www.nice.org.uk/guidance/ta540. Date accessed: May 25.

55. National Institute for Health and Care Excellence (NICE). Venetoclax with rituximab for previously treated chronic lymphocytic leukaemia - Technology appraisal guidance [TA565], 2019. Available from: https://www.nice.org.uk/guidance/ta561. Date accessed: May 25.

56. National Institute for Health and Care Excellence (NICE). Encorafenib with binimetinib for unresectable or metastatic BRAF V600 mutation-positive melanoma - Technology appraisal guidance [TA562], 2019. Available from: https://www.nice.org.uk/guidance/ta562. Date accessed: May 25.

57. National Institute for Health and Care Excellence (NICE). Abemaciclib with an aromatase inhibitor for previously untreated, hormone receptor-positive, HER2-negative, locally advanced or metastatic breast cancer - Technology appraisal guidance [TA563], 2019. Available from: https://www.nice.org.uk/guidance/ta563. Date accessed: May 25.

58. National Institute for Health and Care Excellence (NICE). Benralizumab for treating severe eosinophilic asthma - Technology appraisal guidance [TA565], 2019. Available from: https://www.nice.org.uk/guidance/ta565. Date accessed: May 25.

59. National Institute for Health and Care Excellence (NICE). Ustekinumab for treating moderately to severely active ulcerative colitis - Technology appraisal guidance [TA633], 2020. Available from: https://www.nice.org.uk/guidance/ta633. Date accessed: May 25.

60. National Institute for Health and Care Excellence (NICE). Brigatinib for ALK-positive advanced non-small-cell lung cancer that has not been previously treated with an ALK inhibitor - Technology appraisal guidance [TA670], 2021. Available from: https://www.nice.org.uk/guidance/ta670. Date accessed: May 25.

61. National Institute for Health and Care Excellence (NICE). Mepolizumab for treating severe eosinophilic asthma - Technology appraisal guidance [TA671], 2021. Available from: https://www.nice.org.uk/guidance/ta671. Date accessed: May 25.

62. National Institute for Health and Care Excellence (NICE). Anakinra for treating Still's disease - Technology appraisal guidance [TA685], 2021. Available from: https://www.nice.org.uk/guidance/ta685. Date accessed: May 25.

63. National Institute for Health and Care Excellence (NICE). Selective internal radiation therapies for treating hepatocellular carcinoma - Technology appraisal guidance [TA688], 2024. Available from: https://www.nice.org.uk/guidance/ta688. Date accessed: May 25.

64. National Institute for Health and Care Excellence (NICE). Atezolizumab monotherapy for untreated advanced non-small-cell lung cancer - Technology appraisal guidance [TA705], 2021. Available from: https://www.nice.org.uk/guidance/ta705. Date accessed: May 25.

65. National Institute for Health and Care Excellence (NICE). Diroximel fumarate for treating relapsing–remitting multiple sclerosis - Technology appraisal guidance [TA794], 2022. Available from: https://www.nice.org.uk/guidance/ta794. Date accessed: May 25.

66. National Institute for Health and Care Excellence (NICE). Somatrogon for treating growth disturbance in children and young people aged 3 years and over - Technology appraisal guidance [TA863], 2023. Available from: https://www.nice.org.uk/guidance/ta863. Date accessed: May 25.

67. National Institute For Health and Care Excellence (NICE). Vutrisiran for treating hereditary transthyretin-related amyloidosis - Technology appraisal guidance [TA868], 2023. Available from: https://www.nice.org.uk/guidance/ta868. Date accessed: Mar 24.

68. National Institute for Health and Care Excellence (NICE). Eptinezumab for preventing migraine - Technology appraisal guidance [TA871], 2023. Available from: https://www.nice.org.uk/guidance/ta871. Date accessed: May 25.

69. National Institute for Health and Care Excellence (NICE). Risankizumab for previously treated moderately to severely active Crohn's disease - Technology appraisal guidance [TA888], 2023. Available from: https://www.nice.org.uk/guidance/ta888. Date accessed: May 25.

70. National Institute for Health and Care Excellence (NICE). Pegunigalsidase alfa for treating Fabry disease - Technology appraisal guidance [TA915], 2023. Available from: https://www.nice.org.uk/guidance/ta915. Date accessed: May 25.

71. National Institute for Health and Care Excellence (NICE). Bimekizumab for treating active psoriatic arthritis - Technology appraisal guidance [TA916], 2023. Available from: https://www.nice.org.uk/guidance/ta916. Date accessed: May 25.

72. National Institute for Health and Care Excellence (NICE). Momelotinib for treating myelofibrosis-related splenomegaly or symptoms - Technology appraisal guidance [TA957], 2024. Available from: https://www.nice.org.uk/guidance/ta957. Date accessed: May 25.

73. National Institute for Health and Care Excellence (NICE). Atogepant for preventing migraine - Technology appraisal guidance [TA973], 2024. Available from: https://www.nice.org.uk/guidance/ta973. Date accessed: May 25.

74. National Institute for Health and Care Excellence (NICE). Selective internal radiation therapy with QuiremSpheres for treating unresectable advanced hepatocellular carcinoma - Technology appraisal guidance [TA985], 2024. Available from: https://www.nice.org.uk/guidance/ta985. Date accessed: May 25.

75. National Institute for Health and Care Excellence (NICE). Tenecteplase for treating acute ischaemic stroke - Technology appraisal guidance [TA990], 2024. Available from: https://www.nice.org.uk/guidance/ta990. Date accessed: May 25.

76. National Institute for Health and Care Excellence (NICE). Trastuzumab deruxtecan for treating HER2-low metastatic or unresectable breast cancer after chemotherapy - Technology appraisal guidance [TA992], 2024. Available from: https://www.nice.org.uk/guidance/ta992. Date accessed: May 25.

77. National Institute for Health and Care Excellence (NICE). Pembrolizumab with platinum- and fluoropyrimidine-based chemotherapy for untreated advanced HER2-negative gastric or gastro-oesophageal junction adenocarcinoma - Technology appraisal guidance [TA997], 2024. Available from: https://www.nice.org.uk/guidance/ta997. Date accessed: May 25.

78. National Institute for Health and Care Excellence (NICE). Evinacumab for treating homozygous familial hypercholesterolaemia in people 12 years and over - Technology appraisal guidance [TA1002], 2024. Available from: https://www.nice.org.uk/guidance/ta1002. Date accessed: May 25.

79. National Institute for Health and Care Excellence (NICE). Futibatinib for previously treated advanced cholangiocarcinoma with FGFR2 fusion or rearrangement - Technology appraisal guidance [TA1005], 2024. Available from: https://www.nice.org.uk/guidance/ta1005. Date accessed: May 25.

80. National Institute for Health and Care Excellence (NICE). Latanoprost–netarsudil for previously treated primary open-angle glaucoma or ocular hypertension Technology appraisal guidance [TA1009], 2024. Available from: https://www.nice.org.uk/guidance/ta1009. Date accessed: May 25.

81. National Institute for Health and Care Excellence (NICE). Fenfluramine for treating seizures associated with Lennox–Gastaut syndrome in people 2 years and over - Technology appraisal guidance [TA1050], 2025. Available from: https://www.nice.org.uk/guidance/ta1050. Date accessed: May 25.

82. National Institute for Health and Care Excellence (NICE). Tislelizumab for treating unresectable advanced oesophageal squamous cell cancer after platinum-based chemotherapy [ID4070], 2025. Available from: https://www.nice.org.uk/guidance/indevelopment/gid-ta11042. Date accessed: May 25.

83. National Institute for Health and Care Excellence (NICE). Somapacitan for treating growth hormone deficiency in people 3 to 17 years [ID6178], 2025. Available from: https://www.nice.org.uk/guidance/indevelopment/gid-ta11153. Date accessed: May 25.

84. National Institute for Health and Care Excellence (NICE). Guselkumab for treating moderately to severely active ulcerative colitis [ID6237], 2025. Available from: https://www.nice.org.uk/guidance/indevelopment/gid-ta11247. Date accessed: May 25.

85. National Institute for Health and Care Excellence (NICE). Guselkumab for previously treated moderately to severely active Crohn's disease [ID6238], 2025. Available from: https://www.nice.org.uk/guidance/indevelopment/gid-ta11245. Date accessed: May 25.

86. National Institute for Health and Care Excellence (NICE). Mirikizumab for treating moderately to severely active Crohn's disease [ID6244], 2025. Available from: https://www.nice.org.uk/guidance/indevelopment/gid-ta11267. Date accessed: May 25.

87. National Institute for Health and Care Excellence (NICE). Nivolumab as neoadjuvant (with chemotherapy) and adjuvant (as monotherapy) treatment for resectable non-small-cell lung cancer [ID6310], 2025. Available from: https://www.nice.org.uk/guidance/indevelopment/gid-ta11504. Date accessed: May 25.

88. National Institute for Health and Care Excellence (NICE). Vanzacaftor–tezacaftor–deutivacaftor for treating cystic fibrosis with 1 or more F508del mutations in the CFTR gene in people aged 6 years and over [ID6372], 2025. Available from: https://www.nice.org.uk/guidance/indevelopment/gid-ta11430. Date accessed: May 25.

89. National Institute for Health and Care Excellence (NICE). Abiraterone (originator and generics) for treating newly diagnosed high-risk hormone-sensitive metastatic prostate cancer (review of TA721) [ID6378], 2025. Available from: https://www.nice.org.uk/guidance/indevelopment/gid-ta11730. Date accessed: May 25.

90. National Institute for Health and Care Excellence (NICE). Rilzabrutinib for treating persistent or chronic immune thrombocytopenia in people aged 12 and over [ID6395], 2025. Available from: https://www.nice.org.uk/guidance/awaiting-development/gid-ta11491. Date accessed: May 25.

91. National Institute for Health and Care Excellence (NICE). Darolutamide with androgen deprivation therapy for treating hormone-sensitive metastatic prostate cancer ID6452, 2025. Available from: https://www.nice.org.uk/guidance/indevelopment/gid-ta11557. Date accessed: May 25.

92. National Institute for Health and Care Excellence (NICE). Empagliflozin for treating chronic heart failure with reduced ejection fraction - Technology appraisal guidance [TA773], 2022. Available from: https://www.nice.org.uk/guidance/ta773. Date accessed: May 25.

93. National Institute for Health and Care Excellence (NICE). Empagliflozin for treating chronic heart failure with preserved or mildly reduced ejection fraction - Technology appraisal guidance [TA929] - Final draft guidance. Available from: https://www.nice.org.uk/guidance/ta929/history. Date accessed: Apr 25.

94. National Institute for Health and Care Excellence (NICE). NICE health technology evaluations: the manual, 2023. Available from: https://www.nice.org.uk/process/pmg36. Date accessed: Apr 25.

95. Costello Medical. NICE's 'Proportionate Approach to Technology Appraisals (PATT)': The Story So Far, 2024. Available from: https://www.costellomedical.com/what-we-do/value-and-access/hta/nices-proportionate-approach-to-technology-appraisals/. Date accessed: May 25.
