# Supplementary material for: Clinical similarity in cost-comparison evaluations: a systematic review of current methods in NICE appraisals and the development of a framework for the formal assessment of clinical similarity
Source: BMJ Open. 2026 Jul 21;16(7):e112164. doi: 10.1136/bmjopen-2025-112164 (PMC13410701; doi:10.1136/bmjopen-2025-112164)
Supplement: Supplementary data [file bmjopen-16-7-s003.pdf]

Online Supplementary File 3

Point-and-density plot methods

Development of point-and-density plots

The framework (i.e., point-and-density plots) described in this research does not seek to replace any current methods with regards to performing Bayesian network meta-analyses (NMAs). Indeed, it is recommended that all National Institute for Care and Health Excellence (NICE) Decision Support Unit (DSU) guidelines[1] with regards to indirect treatment comparisons continue to be followed. Instead, the proposed framework should be viewed as an add-on to existing approaches for NMAs, with all relevant procedures and outputs continuing to be provided (e.g., forest plots, trace plots, and Gelman-Rubin diagrams). As such, the following detailed methodology may be considered an 'add-on' analysis to be specifically utilised for NICE Cost Comparison Evaluations (CCEs) and other Technology Appraisals (TA) where an assessment of clinical similarity, or non-inferiority, is required.

The statistics underpinning the generation of point-and-density plots are provided below.

When performing a Bayesian NMA, density plots are generated for each comparison in the network, an example of which is shown in Supplementary Figure 2, which is an example of the default output of the *gemtc* R package.[2] Such a density plot is generated using the values of iterations from across all chains used within a Bayesian analysis and can be produced for each treatment comparison.

Supplementary Figure 2. Default density plot from the *gemtc* R package for the mean difference between an example treatment and comparator.

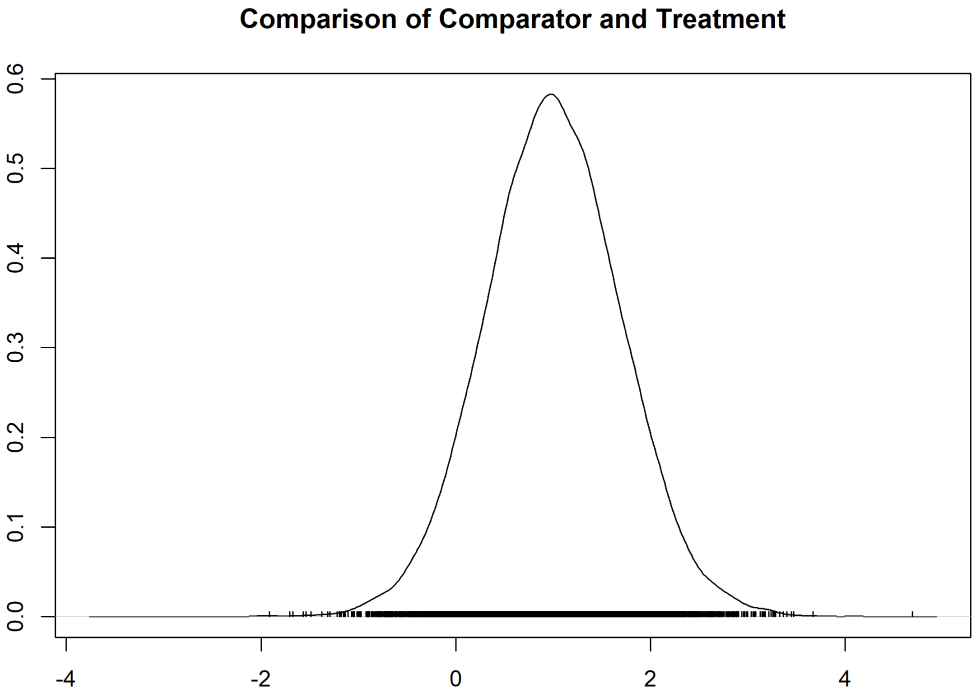

The default density plots produced by the *gemtc* R package[2] have minimal labels. Within Supplementary Figure 2, the x-axis denotes the mean difference of the comparison of the example comparator to the example treatment, while the y-axis denotes the corresponding density.

For each treatment comparison (e.g., the example comparator to the example treatment in Supplementary Figure 2) it is possible to generate an empirical cumulative density function (ECDF) from the density plot. An example of such ECDF is shown in Supplementary Figure 3. In contrast to a regular density plot, an ECDF shows the cumulative density across all values (e.g., mean difference in in Supplementary Figure 3). Within the example from in Supplementary Figure 3, the ECDF will have a cumulative density of 0 at the minimum mean difference reported for the comparison of the example comparator to the example treatment. Likewise, within the example from in Supplementary Figure 3, the ECDF will have a cumulative density of 1 at the maximum mean difference reported for the comparison of the example comparator to the example treatment. Within the example from in Supplementary Figure 3, the ECDF can be used to determine the cumulative density of any point between the minimum and maximum mean difference reported for the comparison of the example comparator to the example treatment. Accordingly, by specifying a value that corresponds to a non-inferiority margin (NIM), minimal clinically important difference (MCID), or other threshold, it is possible to determine the cumulative density of points that fall below, and above, this threshold. in Supplementary Figure 3shows an example of the application of a NIM to an ECDF, where a NIM of -0.20 is specified. As

illustrated in Supplementary Figure 4, the NIM of -0.20 would correspond to a cumulative density of 0.041.

Supplementary Figure 3. Empirical cumulative density function for the comparison of an example treatment and comparator.

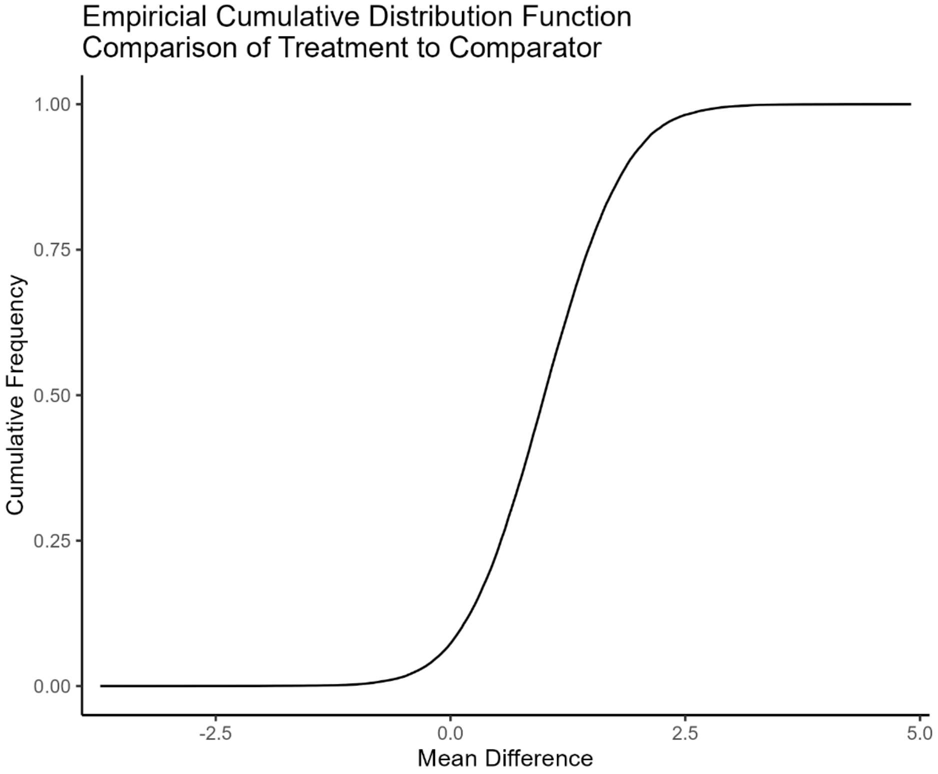

Supplementary Figure 4. Empirical cumulative density function, with a non-inferiority margin, for the comparison of an example treatment and comparator.

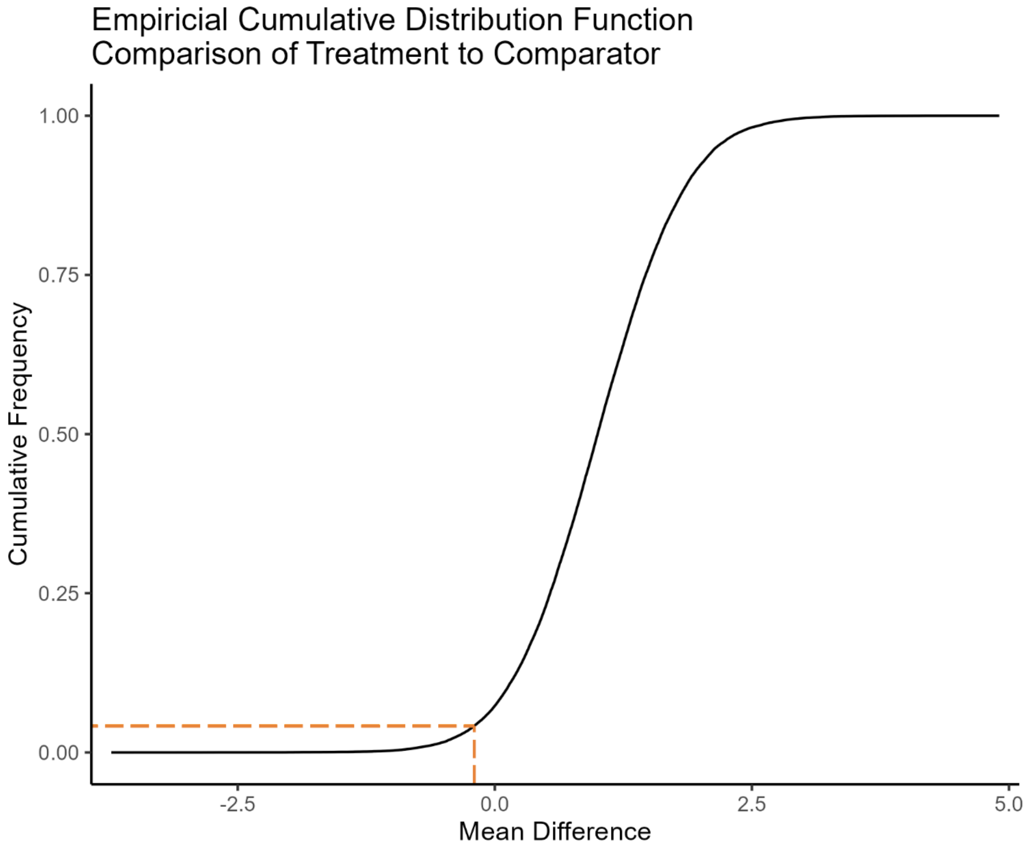

Dashed orange line corresponds to a non-inferiority margin of -0.20 and a corresponding cumulative density of 0.041.

Given the nature of Bayesian analyses, the ECDF for a given comparison can be used to determine the proportion of iterations from the Bayesian analysis that fall below a given threshold (e.g., NIM). Accordingly, within the example shown in Supplementary Figure 4, the cumulative density represents the probability that the mean difference of the comparison of the example comparator to the example treatment falls below the given threshold. As such, the probability that the mean difference of the comparison falls above the given threshold can be calculated. Therefore, by combining the results of the ECDF with those of a traditional density plot and forest plot, it is possible to convey the results of all three separate analyses within a single readily interpretable figure (i.e., a point-and-density plot). An example of a point-and-density plot is shown in Supplementary Figure 5, again using the comparison of the example comparator to the example treatment as above.

Supplementary Figure 5. Point-and-density plot for the comparison of an example treatment and comparator.

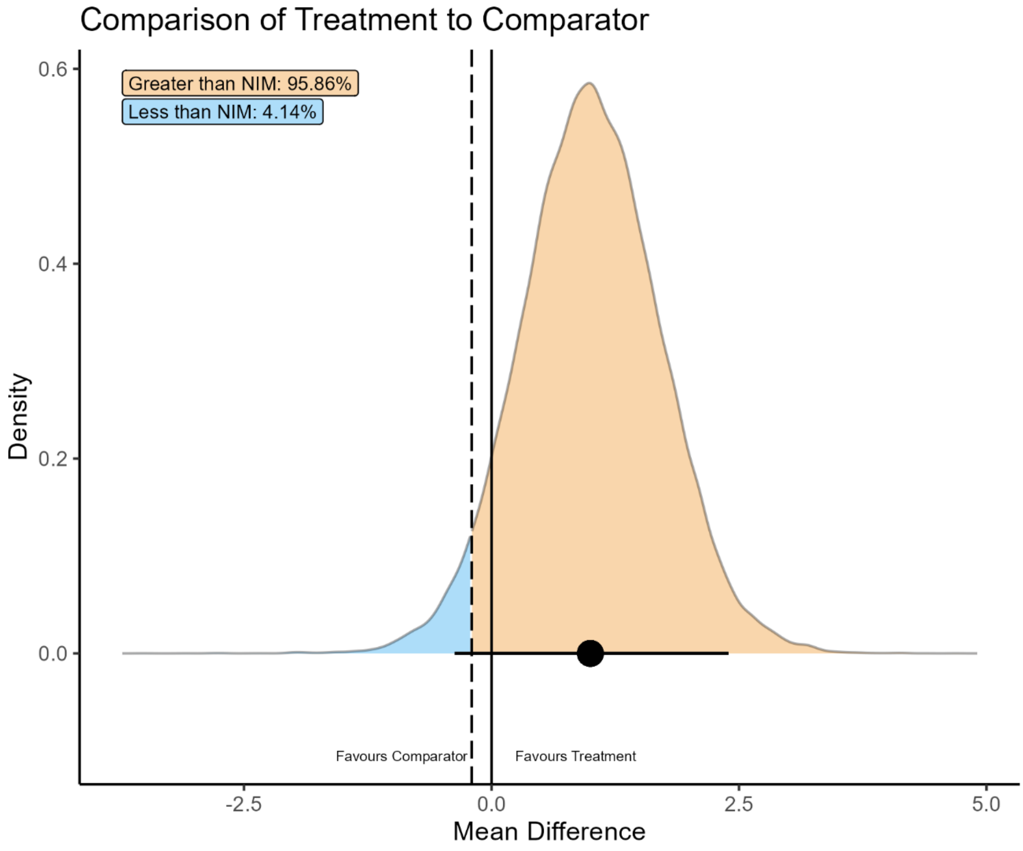

The threshold for traditional hypothesis testing is shown by the solid line, while the dashed line indicates the NIM. The area that falls under (above) the NIM is shown in blue (orange). The probability that the effect size falls under, or above, the NIM is shown in the upper left corner of the plot. The pooled estimate is shown by the black circle, with the error bars corresponding to 95% credible intervals.

Point-and-density plots have been designed to be readily interpretable and showcase data not previously reported from Bayesian NMAs, in addition to data from existing figures (e.g., forest plots). Within Supplementary Figure 5, the thick black line denotes the threshold for traditional hypothesis testing (i.e., either 0 or 1 depending on the effect estimate), while the dashed black line denotes the specified NIM, MCID, or another designated threshold. The point estimate, denoted by the black circle, indicates the pooled estimate for the comparison, with the error bars indicating 95% credible intervals. The point estimate shown within the point-and-density plot will be the same as that shown within a typical forest plot. As shown by Supplementary Figure 5, the density curve aligns with that shown from the typical output of the *gemtc* R package[2] (Supplementary Figure 2). The area under the density curve that falls above and below the NIM is shown in blue and orange, respectively. Likewise, the probability that the effect estimate for the comparison falls above or below the NIM (dashed black line) is shown in the

top-left corner, with these values being derived using the corresponding ECDF. Furthermore, within the point-and-density figure, labels have been added to denote whether a given effect size favours the treatment or the comparator.

As shown by Supplementary Figure 5, the pooled estimate for the comparison of the example comparator to the example treatment is 1.00, with 95% credible intervals of -0.34 to 2.40. Accordingly, based solely on traditional statistical testing, there was no statistically significant difference in treatment effects between the example comparator and the example treatment. In addition, the 95% credible intervals overlap the specified NIM of -0.20, indicating that non-inferiority of the example treatment compared to the example comparator is not demonstrated. However, through the use of the ECDF, the point-and-density plot (Supplementary Figure 5) depicts that there was a 95.87% probability of the example treatment being non-inferior to the example comparator. It is suggested that committees initially focus on whether the 95% credible intervals overlap the NIM to determine whether a treatment can be considered to be non-inferior. However, as in the worked example, the use of probabilities of non-inferiority may provide valuable information, particularly where non-inferiority has not been demonstrated. Additionally, the plots of ECDF functions do not necessarily need to be presented within CCE submissions; instead, it is recommended that committees primarily focus on the results of point-and-density figures.

The above example of the comparison of the example comparator to the example treatment uses a NIM of -0.20. However, for some comparisons a NIM, MCID, or other designated threshold may not be available. Accordingly, when clinically validated thresholds are not available, it is recommended that the corresponding threshold be set to either 0 (for effect estimates such as mean difference) or 1 (for effect estimates such as hazard ratios or odds ratios). By using this approach it is possible to determine the probability that the effect size for the comparison of two treatments will fall above, or below, the corresponding threshold. However, it should be noted that this approach will result in more conservative estimations of non-inferiority compared to using a clinically validated NIM, MCID, or another relevant threshold. Furthermore, in situations where a NIM, or similar threshold, is not reported, by using the corresponding ECDF it is possible to determine a hypothetical threshold. Such a hypothetical threshold can be used to determine the required threshold for a given, arbitrary, probability that a treatment is non-inferior to a comparator. For instance, using the above example, comparing the example comparator to the example treatment, the ECDF reveals that a hypothetical threshold of -0.65 would be required for there to be 99% probability that the example treatment was non-inferior to the example comparator. However, it is important to note

that such a hypothetical threshold should not be considered to be clinically appropriate or meaningful. However, in the absence of NIM, MCID, or other designated threshold, a hypothetical threshold can help inform a committee by indicating whether a relatively large, or small, hypothetical threshold would be required for there to be a certain, arbitrary, probability that a treatment is non-inferior to a comparator. Ultimately, when deriving a hypothetical threshold, the selection of the desired probability of non-inferiority may be arbitrarily selected. However, the authors suggest that a 95% probability of non-inferiority may be selected for a hypothetical threshold. Importantly, the selection of a 95% threshold represents a 'high bar' for any assessments of non-inferiority in the absence of a clinically validated NIM or MCID.

Code

The code to perform the analyses described above has been developed in R v4.2.0[3] and utilises the *gemtc*[2]*, data.table*[4]*, igraph*[5]*, tidyverse*[6]*, magrittr*[7]*, and ggplot2*[8] *packages*. The code was developed by one researcher and subsequently independently reviewed and tested by a second researcher on two separate occasions. The R code was tested using synthetic datasets to ensure that it worked as expected for a variety of link functions (e.g., *cloglog*, *identity*, *logit*, and *log*) and data types (e.g., mean and standard deviations or the overall number of patients and the number of responders).

The results of the code were also compared to the *gemtcPlus* package[9] which allows users to determine the probability of that an effect size falls above, or below, a threshold of 0 or 1. While users of the *gemtcPlus* package[9] cannot specify an alternative threshold, comparisons of the results obtained using the *gemtcPlus* package[9] and the approach detailed here aligns.

R code and NMA output to generate example point-and-density plots from the case study for crovalimab for Paroxysmal Nocturnal Haemoglobinuria (TA1019)[10] are provided as supplementary materials (Online Supplementary Files 4 and 5).

Crovalimab for Paroxysmal Nocturnal Haemoglobinuria (TA1019)

Network of evidence

Within TA1019[10], the company identified a connected evidence network (Supplementary Figure 6) that comprised crovalimab and the comparators of eculizumab, ravulizumab, and standard of care for the endpoint of transfusion avoidance.

Supplementary Figure 6. Network of evidence for studies included within network meta-analyses for the percentage of patients achieving transfusion avoidance for the overall population.

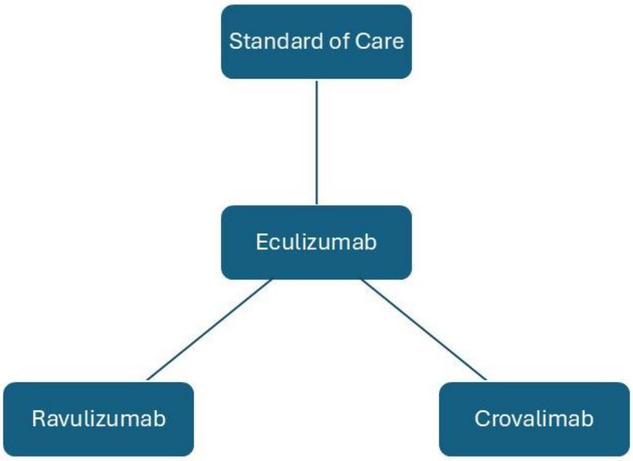

Raw data

The raw data used to perform the original analyses was reported in TA1019[10], as such these data were used to perform the re-analyses for the overall population. Data were extracted from TA1019[10] by one researcher and independently verified by a second researcher. Overall, the raw data used in the original analysis and re-analysis are shown in Supplementary Table 7.

Supplementary Table 7. Data used in the analyses and re-analyses of the network meta-analyses for the percentage of patients achieving transfusion avoidance (taken from page 62 of the company submission).

Study	Population	Treatment	Mean	Standard Error
Study 301[11]				
	Naïve	Ravulizumab	0.736	0.039
	Naïve	Eculizumab	0.661	0.043
TRIUMPH[12]				
	Naïve	Eculizumab	0.51	0.078
	Naïve	Standard of Care	0	0.025
COMMODORE 2[13]				
	Naïve	Crovalimab	0.664	0.042
	Naïve	Eculizumab	0.681	0.058
Study 302[14]				
	Experienced	Ravulizumab	0.876	0.033
	Experienced	Eculizumab	0.827	0.038
COMMODORE 1[15]				
	Experienced	Crovalimab	0.795	0.069

	Experienced	Eculizumab	0.811	0.069

Network meta-analyses

As detailed above, analyses were performed in R v4.2.0[3] and utilised the *gemtc*[2]*, data.table*[4]*, igraph*[5]*, tidyverse*[6]*, magrittr*[7]*, and ggplot2*[8] R packages. Network meta-analyses were performed using a random-effects model, with 5 chains, a burn in period of 5000 iterations, a total of 100,000 iterations, and a thinning margin of 10. As the outcome of transfusion avoidance was analysed using mean differences, an identity link function was specified.

References

1. University of Sheffield - NICE Decision Support Unit. Full list of technical support documents (TSDs), 2025. Available from: https://www.sheffield.ac.uk/nice-dsu/tsds/full-list. Date accessed: May 25.
2. van Valkenhoef G, Kuiper J. gemtc: Network Meta-Analysis Using Bayesian Methods v1.0.2. 2023.
3. Computing TRFfS. R version 4.2.0. 2022.
4. Barrett T, Dowle M, Srinivasan A, Gorecki J, Chirico M, Hocking T, et al. data.table: Extension of `data.frame` v1.17.0. 2025.
5. Csardi G, Nepusz T. The igraph software package for complex network research v2.0.3. 2006.
6. Wickham H, Averick M, Bryan J, Chang W, McGowan LDA, François R, et al. Welcome to the Tidyverse. *Journal of Open Source Software* 2019; **4**: 1686.
7. Bache S, Wickham H. magrittr: A Forward-Pipe Operator for R v2.0.3. 2022.
8. Wickham H, Sievert C. *ggplot2: Elegant Graphics for Data Analysis*: Springer New York; 2009.
9. Gsteiger S, Howlett N, Ashlee B. gemtcPlus: Provides a suite of extension functions for NMA using the `gemtc` package v1.0.0. 2025.
10. National Institute for Health and Care Excellence (NICE). Crovalimab for treating paroxysmal nocturnal haemoglobinuria in people 12 years and over - Technology appraisal guidance [TA1019], 2024. Available from: https://www.nice.org.uk/guidance/ta1019. Date accessed: Apr 25.
11. Lee JW, Sicre de Fontbrune F, Wong Lee Lee L, Pessoa V, Gualandro S, Füreder W, et al. Ravulizumab (ALXN1210) vs eculizumab in adult patients with PNH naive to complement inhibitors: the 301 study. *Blood* 2019; **133**: 530-9.
12. Hillmen P, Young NS, Schubert J, Brodsky RA, Socié G, Muus P, et al. The complement inhibitor eculizumab in paroxysmal nocturnal hemoglobinuria. *The New England journal of medicine* 2006; **355**: 1233-43.
13. Röth A, He G, Tong H, Lin Z, Wang X, Chai-Adisaksopha C, et al. Phase 3 randomized COMMODORE 2 trial: Crovalimab versus eculizumab in patients with paroxysmal nocturnal hemoglobinuria naive to complement inhibition. *Am J Hematol* 2024; **99**: 1768-77.
14. Kulasekararaj AG, Hill A, Rottinghaus ST, Langemeijer S, Wells R, Gonzalez-Fernandez FA, et al. Ravulizumab (ALXN1210) vs eculizumab in C5-inhibitor-experienced adult patients with PNH: the 302 study. *Blood* 2019; **133**: 540-9.
15. Scheinberg P, Clé DV, Kim JS, Nur E, Yenerel MN, Barcellini W, et al. Phase 3 randomized COMMODORE 1 trial: Crovalimab versus eculizumab in complement inhibitor-experienced patients with paroxysmal nocturnal hemoglobinuria. *Am J Hematol* 2024; **99**: 1757-67.
