# Supplementary material for: Clinical similarity in cost-comparison evaluations: a systematic review of current methods in NICE appraisals and the development of a framework for the formal assessment of clinical similarity
Source: BMJ Open. 2026 Jul 21;16(7):e112164. doi: 10.1136/bmjopen-2025-112164 (PMC13410701; doi:10.1136/bmjopen-2025-112164)
Supplement: Reviewer comments [file bmjopen-2025-112164.reviewer_comments.pdf]

PEER REVIEW HISTORY

BMJ Open publishes all reviews undertaken for accepted manuscripts. Reviewers are asked to complete a checklist review form and are provided with free text boxes to elaborate on their assessment. These free text comments are reproduced below.

ARTICLE DETAILS

Title (Provisional)

Clinical similarity in cost-comparison evaluations: a systematic review of current methods in NICE appraisals and the development of a framework for the formal assessment of clinical similarity

Authors

Edwards, Steve; Burgess, Benjamin Joshua; Downes, Nicole; Ip, Sophie; Walters, Archie; Dadswell, Clare

---

VERSION 1 - REVIEW

**Reviewer**	**1**
**Name**	**Dong, Olivia**
**Affiliation**	**RTI Health Solutions Research Triangle Park**
**Date**	**22-Dec-2025**
**COI**	**None**

This paper was well written, uses robust methodology, and addresses critical research questions. A few minor suggestions are provided below for the authors to consider:

Introduction:

Page 5, line 7: Define NHS at first mention

Page 5, line 18: It would be helpful to provide some examples of what specific circumstances allow CCEs

Page 5, line 29: In reference to this content: "…while this process was replaced in 2022…", what was the process replaced with? Does this mean the FTA was no longer available as an option?

Page 5, line 44: Are CCEs allowed in multiple technology appraisals (MTAs) or are they only allowed in STAs?

Methods:

Page 9, line 14: Was there a reason for selecting the year 2017 as the starting year for the search?

Page 10, line 23: Would be helpful to describe the general distinction between Bayesian NMA vs. NMA. What makes it Bayesian and in what situations would it be used vs. using a non-Bayesian NMA?

Page 88, line 38: For “R code and NMA output to generate example point-and-density plots from the case study for crovalimab for Paroxysmal Nocturnal Haemoglobinuria (TA1019)10 are provided as supplementary materials.”, it would be helpful to specify the file numbers for the R code and NMA output.

Page 11, line 29: In reference to the R code that is provided as part of online Supplementary File 4 and 5, more instructions in the R file for the user would be helpful to enable the code to run successfully. I opened the file and ran it as is, and there is a step that requires the libraries to be installed before they are loaded (the installation step would need to be added before R code line 12). Also, when I highlighted the entire code and ran it I got an error that said “Error in fwrite(df_tipping, file = paste0(output_directory_comparison, : No such file or directory: 'G:/My Drive/Cost comparison analysis/Comparison of Eculizumab to Crovalimab/tipping_points.csv'. Unable to create new file for writing (it does not exist already). Do you have permission to write here, is there space on the disk and does the path exist?” If there are spots in the code where the user is required to update the code to ensure it runs successfully, it would be helpful to include these instructions at the top of the code so the user knows what to look for throughout the code.

Results:

Page 13, line 23: Would be helpful to describe briefly the content provided in Tables 3 to 5 of the Supplementary File 1 so the reader knows what to expect in these tables. Same comment for page 13, lines 25-27 where Tables 1 and 2 are mentioned.

Page 14, line 20: Where Table 6 of the online supplementary file 1 is mentioned, the table seems to be missing the abbreviation section (see page 58, line 28)

Discussion/Conclusion sections:

A short discussion on the unanswered questions/specific directions for future research would be helpful in this section. Are there aspects of this novel framework that need to be further tested? How do we know NICE would agree with the recommendations outlined in the paper?

**Reviewer**	**2**
**Name**	**Zemplenyi, Antal**
**Affiliation**	**Syreon Research Institute**
**Date**	**23-Dec-2025**
**COI**	**None**

Thank you for the opportunity to review this manuscript titled "Clinical Similarity in Cost-Comparison Evaluations: A Systematic Review of Current Methods in NICE Appraisals and the Development of a Novel Framework for the Formal Assessment of Clinical Similarity."

This paper is well-written and addresses an important issue regarding clinical similarity assessments in cost-comparison evaluations. I have a few minor comments for the authors to consider.

Firstly, I suggest reevaluating the use of "novel framework" in the title. As I understand it, the authors do not propose to replace existing methods but rather to enhance the interpretation of results and their implications for decision-making.

The results are presented clearly and in a structured manner. However, I believe that a high-level summary table in the results section, beyond Table 2 and the tables in the appendix, could improve readability. Since one of the main reasons for developing an alternative framework is to address inconsistencies in clinical similarity assessments, I recommend that the authors focus more on illustrating how these inconsistencies arise in appraisals. For instance, a summary table detailing the methods used in various studies and examples, if any, where similar results led to differing conclusions, would help strengthen the case for the enhanced statistical framework.

Additionally, the inconsistencies across appraisals, between companies, and EAGs, could be emphasized further and substantiated with specific examples.

Regarding the statistical framework, the methodology presented in this manuscript is sound and effectively improves the communication of non-inferiority evidence by combining interval-based results with probabilistic summaries in an intuitive visualization.

To strengthen its application and prevent misinterpretation, the manuscript could more clearly state that formal non-inferiority testing remains the primary focus, with probabilities serving a supportive and contextual role. Additional guidance on probability reporting, explicit labeling of hypothetical thresholds, and a brief discussion on model dependence could further enhance clarity.

**Reviewer**	**3**
**Name**	**Dack, Kyle**
**Affiliation**	**University of Bristol, MRC Integrative Epidemiology Unit**
**Date**	**22-Feb-2026**
**COI**	**None**

Statistical review summary:

This manuscript presents a novel and potentially valuable statistical framework for appraising non-inferiority between treatments based on non-significant network meta-analysis results.

The manuscript is clear and transparent, and the authors have done well to perform a large evidence synthesis of 41 papers plus present this framework. Regarding the statistical framework, I did not identify any unmet assumptions or overreach in the interpretation. However, it is challenging to identify and verify all assumptions when a new statistical approach is presented, so I recommend cross-checking this with other reviewers.

I do not have major concerns or suggestions, my comments below are minor suggestions to improve clarity.

Abstract:

1. It may be more precise to state the review was reported in accordance with PRISMA (as opposed to "conducted"), as PRISMA is a reporting guideline rather than methodological instruction. (even if it does indirectly influence the review design).

2. "from 2017 to April 2025" if space it would be helpful to clarify that 2017 is the onset of using CCE in the NICE appraisal program, so abstract readers understand this is covering the entire period they have been used.

Methods:

3. I suggest moving the statement regarding the protocol to the beginning of the methods. This is because there is a reference to "the protocol" in supplementary material 1, but at this point in the text the protocol was not yet introduced. It would also be informative to confirm in-text that there are no major deviations (in my opinion).

4. Search & screening: It is reported that the screening of initial results was performed by a single review and validated by a second reviewer. What did this validation process involve?

5. Primary outcomes: There is information in supplementary file 1 regarding (a) how outcomes were identified within appraisals and (b) what information was extracted. The main text contains a summary of the data extraction, including a mention of primary outcomes.

-However, the main text does not directly define what the primary outcomes are, or what information will be subject to the narrative synthesis. This is essential for readers to accurately understand the design and be prepared for the results, so I suggest the authors consider adding a brief paragraph prior to the "data extraction" section which lists or defines the outcomes which will be synthesized.

6. RoB: I agree with the authors rationale for not performing risk of bias assessment – such an evaluation would not be aligned with the aims of the research.

Statistical framework:

7. The approach is statistically legitimate, I do not identify any assumptions being broken or biases being introduced, and the resulting figure (e.g. figure 2) is aligned with the aims and is user-friendly.

-My only minor comment relates to the section which reports approaches which could be used in the absence of NIM. An 95% threshold is suggested, and it is reported that this aligns with the typical threshold used for statistical significance testing. This may overstate the equivalence to the frequentist 0.05 alpha threshold, as in standard testing it is reflective of type 1 error rate, whereas in this Bayesian approach it reflects the posterior mass below the margin. It may be helpful to suggest also exploring different arbitrary thresholds to see how sensitive the results are to this decision.

8. R code: The code is clearly organised and presented, and again is user friendly – no suggestions.

Results:

9. “A range of disease areas were covered by the included appraisals, but some were more commonly represented than others.” This sentence reads a little vague because the comment is not elaborated on, and it may be useful to report which diseases area were more commonly represented, or point readers to table 1 where they can see for themselves.

10. Tables and figures: The tables, including those in the supplementary files, are informative and clear. There is a lot of information which was identified and synthesized, and it can be challenging to make this interpretable for readers, but the authors have done a very good job. [no suggestions]

Discussion:

11. I recommend acknowledging as a limitation that the search / screening / data extraction were in parts conducted by one person, which is not typically recommended. Validation by a second researcher occurred, and this is a reasonable approach to mitigating any risks of errors, but it would be useful to note this.

VERSION 1 - AUTHOR RESPONSE

Response to Reviewer 1 (Dr. Olivia Dong, RTI Health Solutions)

This paper was well written, uses robust methodology, and addresses critical research questions. A few minor suggestions are provided below for the authors to consider:

We thank Dr. Dong for their supportive comments and for the time spent reviewing our manuscript. We have incorporated their suggestions into the revised text and have provided a point-by-point response below.

Introduction:

Page 5, line 7: Define NHS at first mention

Thank you for highlighting this oversight. We have defined NHS at the first mention (lines 111 to 112).

Page 5, line 18: It would be helpful to provide some examples of what specific circumstances allow CCEs

Thank you for this suggestion; this additional detail has now been added to this sentence within the manuscript. In order to avoid duplication, similar text has been removed from a later paragraph (lines 116 to 119).

Page 5, line 29: In reference to this content: "…while this process was replaced in 2022…", what was the process replaced with? Does this mean the FTA was no longer available as an option?

Thank you for raising these questions. The original Fast-Track Appraisal process involved routes other than the cost-comparison approach (based on cost per quality-adjusted life year being likely to be below particular thresholds); these were removed while the cost-comparison approach remained an option after 2022. This has been made clearer within the relevant text of the manuscript (lines 124 to 128).

Page 5, line 44: Are CCEs allowed in multiple technology appraisals (MTAs) or are they only allowed in STAs?

Thank you for your question. CCEs are not typically used in MTAs but are an alternative to an STA when assessing a single new intervention if the intervention being assessed is "likely to provide similar or greater health benefits at similar or lower cost than the relevant comparator(s)". This has been made clearer in the manuscript (lines 132 to 135).

Methods:

Page 9, line 14: Was there a reason for selecting the year 2017 as the starting year for the search?

Thank you for your question. We chose 2017 as the start year given this is when CCEs were first introduced within the NICE appraisal process, aiming to reduce the number of irrelevant records reviewed. This rationale has been added to the manuscript (lines 231 and 232).

Page 10, line 23: Would be helpful to describe the general distinction between Bayesian NMA vs. NMA. What makes it Bayesian and in what situations would it be used vs. using a non-Bayesian NMA?

We thank Dr. Dong for raising this point. Accordingly, we have added additional text to the discussion (lines 555 to 564) that considers the application, and choice, of frequentist and Bayesian NMAs.

Page 88, line 38: For "R code and NMA output to generate example point-and-density plots from the case study for crovalimab for Paroxysmal Nocturnal Haemoglobinuria (TA1019)10 are provided as supplementary materials.", it would be helpful to specify the file numbers for the R code and NMA output.

We thank Dr. Dong for highlighting this point. We have specified the file numbers in which the R code and NMA output can be found (line 135 of Online Supplementary File 3).

Page 11, line 29: In reference to the R code that is provided as part of online Supplementary File 4 and 5, more instructions in the R file for the user would be helpful to enable the code to run successfully. I opened the file and ran it as is, and there is a step that requires the libraries to be installed before they are loaded (the installation step would need to be added before R code line 12). Also, when I highlighted the entire code and ran it I got an error that said "Error in fwrite(df_tipping, file = paste0(output_directory_comparison, : No such file or directory: 'G:/My Drive/Cost comparison analysis/Comparison of Eculizumab to Crovalimab/tipping_points.csv'. Unable to create new file for writing (it does not exist already). Do you have permission to write here, is there space on the disk and does the path exist?" If there are spots in the code where the user is required to update the code to ensure it runs successfully, it would be helpful to include these instructions at the top of the code so the user knows what to look for throughout the code.

We thank Dr. Dong for their comments on the R code. The code has been structured so that any variables that require user adjustment are found under the 'User Specified Variables' section of the code (line 300 of Online Supplementary File 4). For clarity, we have updated the description of the 'output_directory' variable to outline that this variable must be adjusted by users, to a file pathway of their choosing, for the code to be run without any issues (line 303 of Online Supplementary File 4).

Results:

Page 13, line 23: Would be helpful to describe briefly the content provided in Tables 3 to 5 of the Supplementary File 1 so the reader knows what to expect in these tables. Same comment for page 13, lines 25-27 where Tables 1 and 2 are mentioned.

Thank you for these suggestions. We have now added a brief summary of what these tables include to facilitate understanding of the content within these tables (lines 354 to 362).

Page 14, line 20: Where Table 6 of the online supplementary file 1 is mentioned, the table seems to be missing the abbreviation section (see page 58, line 28)

We thank Dr. Dong for highlighting the missing abbreviations in Table 6 of Online Supplementary File 1. We have added in these abbreviations accordingly (line 125 of Online Supplementary File 2, previously Online Supplementary File 1).

Discussion/Conclusion sections:
A short discussion on the unanswered questions/specific directions for future research would be helpful in this section. Are there aspects of this novel framework that need to be further tested?

We thank Dr. Dong for raising these points. We are not aware of any elements of this approach that require further testing at present. However, we have noted that future research may seek to apply the framework to alternative Bayesian methods (e.g., multi-level network meta-regression), which would require additional code development and testing. As such, we have stated that future research may seek to consider this within the discussion section (lines 575 to 578).

How do we know NICE would agree with the recommendations outlined in the paper?

We thank Dr. Dong for posing this question. We note that the recommendations listed within this manuscript support NICE processes and formalise an approach that is implicitly being used by NICE, and EAGs, at present. We note that EAGs may use whatever methods that they consider to be appropriate to assess clinical similarity. As such, we, as an EAG, have begun to implement this framework within our assessments of CCEs, which has been welcomed by NICE.

Response to Reviewer 2 (Dr. Antal Zemplenyi, Syreon Research Institute)

Thank you for the opportunity to review this manuscript titled "Clinical Similarity in Cost-Comparison Evaluations: A Systematic Review of Current Methods in NICE Appraisals and the Development of a Novel Framework for the Formal Assessment of Clinical Similarity."
This paper is well-written and addresses an important issue regarding clinical similarity assessments in cost-comparison evaluations. I have a few minor comments for the authors to consider.

We thank Dr. Zemplenyi for their constructive feedback on our manuscript. We have responded to each of their suggestions on a point-by-point basis below.

Firstly, I suggest reevaluating the use of "novel framework" in the title. As I understand it, the authors do not propose to replace existing methods but rather to enhance the interpretation of results and their implications for decision-making.

We thank Dr. Zemplenyi for their suggestion. We have accordingly adjusted the title of, and text within, the manuscript to omit the word 'novel' (lines 6, 18, 94, 96, 200, 208, 308, 409, 493, 589, 674 and 684).

The results are presented clearly and in a structured manner. However, I believe that a high-level summary table in the results section, beyond Table 2 and the tables in the appendix, could improve readability. Since one of the main reasons for developing an alternative framework is to address inconsistencies in clinical similarity assessments, I recommend that the authors focus more on illustrating how these inconsistencies arise in appraisals. For instance, a summary table detailing the methods used in various studies and examples, if any, where similar results led to differing conclusions, would help strengthen the case for the enhanced statistical framework.

Thank you for raising this concern. We have addressed this by making it clear earlier in the background section (lines 159 to 160) that there is not currently any guidance from NICE in terms of how non-significant results from ITCs should be interpreted in terms of proving clinical similarity, which we consider to be an important factor contributing to the current differences in approach across CCEs, as already discussed in the 'existing guidance and research' and 'recommendations' sections. Furthermore, we have added an additional table (Table 3, with discussion on lines 381 to 394) that compares methods and outcomes in two specific examples, which should hopefully help to explain the inconsistencies described as being identified more clearly.

Additionally, the inconsistencies across appraisals, between companies, and EAGs, could be emphasized further and substantiated with specific examples.

Thank you for raising this concern. In line with the response to the above comment, we have added an additional table (Table 3, with discussion on lines 381 to 394) within the manuscript that compares methods and outcomes in two specific examples, which should hopefully help to explain the inconsistencies described as being identified more clearly.

Regarding the statistical framework, the methodology presented in this manuscript is sound and effectively improves the communication of non-inferiority evidence by combining interval-based results with probabilistic summaries in an intuitive visualization.

We thank Dr. Zemplenyi for their positive feedback on the presented statistical framework. We appreciate their supportive assessment of the methodology and are very pleased that this element of the manuscript was welcomed.

To strengthen its application and prevent misinterpretation, the manuscript could more clearly state that formal non-inferiority testing remains the primary focus, with probabilities serving a supportive and contextual role.

We thank Dr. Zemplenyi for this suggestion. As such, we have stated that formal non-inferiority testing should remain the primary focus of any assessments of clinical similarity when introducing the statistical framework (lines 274 to 276).

Additional guidance on probability reporting, explicit labeling of hypothetical thresholds, and a brief discussion on model dependence could further enhance clarity.

We thank Dr. Zemplenyi for raising these points. In response to these comments, we have added additional text clearly stating that any hypothetical thresholds must be clearly labelled as being hypothetical and not based on a clinically validated NIM (lines 542 to 545). We have additionally added text to the discussion related to model dependence (lines 566 to 573). Finally, we note that throughout the manuscript we have referred to the probability of an intervention being non-inferior to a comparator, with the reported probability of non-inferiority being a key element of the point-and-density figures (e.g., Figure 4).

Response to Reviewer 3 (Dr. Kyle Dack, University of Bristol)

Statistical review summary:
This manuscript presents a novel and potentially valuable statistical framework for appraising non-inferiority between treatments based on non-significant network meta-analysis results.
The manuscript is clear and transparent, and the authors have done well to perform a large evidence synthesis of 41 papers plus present this framework. Regarding the statistical framework, I did not identify any unmet assumptions or overreach in the interpretation. However, it is challenging to identify and verify all assumptions when a new statistical approach is presented, so I recommend cross-checking this with other reviewers.
I do not have major concerns or suggestions, my comments below are minor suggestions to improve clarity.

We thank Dr. Dack for their warm comments on our manuscript and for the time taken to provide a thoughtful review. Our point-by-point responses to their comments and suggestions are provided below.

Abstract:

1. It may be more precise to state the review was reported in accordance with PRISMA (as opposed to “conducted”), as PRISMA is a reporting guideline rather than methodological instruction. (even if it does indirectly influence the review design).

Thank you for this suggestion. Given the abstract was reformatted during this revision in response to another request, we no longer mention PRISMA in the abstract. However, in the main manuscript text we have added a sentence to explain that the SLR was reported in accordance with PRISMA (lines 217 to 219).

2. “from 2017 to April 2025” if space it would be helpful to clarify that 2017 is the onset of using CCE in the NICE appraisal program, so abstract readers understand this is covering the entire period they have been used.

Thank you very much for this comment, this has been made clearer in the abstract and in the main body of text (lines 231 to 232).

Methods:

3. I suggest moving the statement regarding the protocol to the beginning of the methods. This is because there is a reference to “the protocol” in supplementary material 1, but at this point in the text the protocol was not yet introduced. It would also be informative to confirm in-text that there are no major deviations (in my opinion).

Thank you for this suggestion. The text relating to the protocol has been moved to the beginning of the methods section (lines 216 to 217) and text about there being no major deviations has been added (line 217), as suggested. This required online supplementary files 1 and 2 to be reordered, which has been implemented throughout the manuscript and supporting documents.

4. Search & screening: It is reported that the screening of initial results was performed by a single review and validated by a second reviewer. What did this validation process involve?

Thank you for your comment, further detail relating to this validation process has been added to the manuscript (lines 235 to 238).

5. Primary outcomes: There is information in supplementary file 1 regarding (a) how outcomes were identified within appraisals and (b) what information was extracted. The main text contains a summary of the data extraction, including a mention of primary outcomes.
-However, the main text does not directly define what the primary outcomes are, or what information will be subject to the narrative synthesis. This is essential for readers to accurately understand the design and be prepared for the results, so I suggest the authors consider adding a brief paragraph prior to the "data extraction" section which lists or defines the outcomes which will be synthesized.

Thank you for this suggestion, further details about the primary outcomes in this research have been added to the manuscript (lines 239 to 247) based on some of the information already included in the supplementary file. Furthermore, we have now highlighted that the information relating to methods for decision-making and interpretation of clinical similarity was the information used in narrative syntheses (line 258).

6. RoB: I agree with the authors rationale for not performing risk of bias assessment – such an evaluation would not be aligned with the aims of the research.

We appreciate the Dr. Dack's agreement with our rationale for not performing a risk of bias assessment.

Statistical framework:
7. The approach is statistically legitimate, I do not identify any assumptions being broken or biases being introduced, and the resulting figure (e.g. figure 2) is aligned with the aims and is user-friendly.
-My only minor comment relates to the section which reports approaches which could be used in the absence of NIM. An 95% threshold is suggested, and it is reported that this aligns with the typical threshold used for statistical significance testing. This may overstate the equivalence to the frequentist 0.05 alpha threshold, as in standard testing it is reflective of type 1 error rate, whereas in this Bayesian approach it reflects the posterior mass below the margin. It may be helpful to suggest also exploring different arbitrary thresholds to see how sensitive the results are to this decision.

We thank Dr. Dack for their positive feedback regarding the statistical framework. In response to their comments, we have removed any comparisons of the 95% threshold to frequentist significance testing (lines 300 to 301, 549 to 550, and 641 to 643 of manuscript and lines 117 to 118 of Online Supplementary File 3). Additionally, we have also expanded the discussion of considering alternative thresholds, within the discussion, to outline how sensitive the assessments of non-inferiority are to the choice of hypothetical threshold (lines 550 to 553).

8. R code: The code is clearly organised and presented, and again is user friendly – no suggestions.

We thank Dr. Dack for their kind words regarding the presentation of our code. We are glad to hear that the materials provided are both clearly organised and user-friendly.

Results:

9. “A range of disease areas were covered by the included appraisals, but some were more commonly represented than others.” This sentence reads a little vague because the comment is not elaborated on, and it may be useful to report which diseases area were more commonly represented, or point readers to table 1 where they can see for themselves.

Thank you for this suggestion, we have now mentioned a couple of the more common disease areas and linked to Table 1 of Supplementary File 2 (previously Supplementary File 1, on lines 342 to 344).

10. Tables and figures: The tables, including those in the supplementary files, are informative and clear. There is a lot of information which was identified and synthesized, and it can be challenging to make this interpretable for readers, but the authors have done a very good job. [no suggestions]

We thank Dr. Dack for their positive feedback on the include tables and figures. We appreciate their supportive assessment and are very glad to see this component of the study was well-received.

Discussion:

11. I recommend acknowledging as a limitation that the search / screening / data extraction were in parts conducted by one person, which is not typically recommended. Validation by a second researcher occurred, and this is a reasonable approach to mitigating any risks of errors, but it would be useful to note this.

Thank you for raising this. We have now added this as a limitation to the discussion section (lines 484 to 488).

References

1. National Institute for Health and Care Excellence. Nivolumab with chemotherapy for neoadjuvant treatment then alone for adjuvant treatment of resectable non-small-cell lung cancer [TA1127] - Committee papers, 2026. Available from: https://www.nice.org.uk/guidance/TA1127. Date accessed: May 26.

VERSION 2 - REVIEW

**Reviewer**	**2**
**Name**	**Zemplenyi, Antal**
**Affiliation**	**Syreon Research Institute**
**Date**	**28-May-2026**
**COI**	

Thank you for addressing my comments.

**Reviewer**	**3**
**Name**	**Dack, Kyle**
**Affiliation**	**University of Bristol, MRC Integrative Epidemiology Unit**
**Date**	**29-May-2026**
**COI**	

The revisions addressed all my queries and suggestions.
