# Supplementary material for: Clinical similarity in cost-comparison evaluations: a systematic review of current methods in NICE appraisals and the development of a framework for the formal assessment of clinical similarity
Source: BMJ Open. 2026 Jul 21;16(7):e112164. doi: 10.1136/bmjopen-2025-112164 (PMC13410701; doi:10.1136/bmjopen-2025-112164)
Supplement: Author's manuscript [file bmjopen-2025-112164.draft_revisions.pdf]

BMJ Open is committed to open peer review. As part of this commitment we make the peer review history of every article we publish publicly available.

When an article is published we post the peer reviewers' comments and the authors' responses online. We also post the versions of the paper that were used during peer review. These are the versions that the peer review comments apply to.

The versions of the paper that follow are the versions that were submitted during the peer review process. They are not the versions of record or the final published versions. They should not be cited or distributed as the published version of this manuscript.

BMJ Open is an open access journal and the full, final, typeset and author-corrected version of record of the manuscript is available on our site with no access controls, subscription charges or pay-per-view fees (http://bmjopen.bmj.com).

If you have any questions on BMJ Open's open peer review process please email info.bmjopen@bmj.com

BMJ Open

Clinical similarity in cost-comparison evaluations: a systematic review of current methods in NICE appraisals and the development of a novel framework for the formal assessment of clinical similarity

Journal:	*BMJ Open*
Manuscript ID	bmjopen-2025-112164
Article Type:	Original research
Date Submitted by the Author:	09-Oct-2025

Complete List of Authors:	Edwards, Steve; BMJ Technology Assessment Group, BMJ-TAG, BMJ Group<br>Burgess, Benjamin; BMJ Technology Assessment Group, BMJ-TAG, BMJ Group<br>Downes, Nicole; BMJ Technology Assessment Group, BMJ-TAG, BMJ Group<br>Ip, Sophie; BMJ Technology Assessment Group, BMJ-TAG, BMJ Group<br>Walters, Archie; BMJ Technology Assessment Group, BMJ-TAG, BMJ Group<br>Dadswell, Clare; BMJ Technology Assessment Group, BMJ-TAG, BMJ Group

Keywords:	Systematic Review, QUALITATIVE RESEARCH, STATISTICS & RESEARCH METHODS, Network Meta-Analysis

Note: The following files were submitted by the author for peer review, but cannot be converted to PDF. You must view these files (e.g. movies) online.

Online Supplementary File 4_Publication_Analysis_Code.R
Online Supplementary File 5_Publication_Analysis_Data.RData

SCHOLARONE MANUSCRIPTS

I, the Submitting Author has the right to grant and does grant on behalf of all authors of the Work (as defined in the below author licence), an exclusive licence and/or a non-exclusive licence for contributions from authors who are: i) UK Crown employees; ii) where BMJ has agreed a CC-BY licence shall apply, and/or iii) in accordance with the terms applicable for US Federal Government officers or employees acting as part of their official duties; on a worldwide, perpetual, irrevocable, royalty-free basis to BMJ Publishing Group Ltd ("BMJ") its licensees and where the relevant Journal is co-owned by BMJ to the co-owners of the Journal, to publish the Work in this journal and any other BMJ products and to exploit all rights, as set out in our licence.

The Submitting Author accepts and understands that any supply made under these terms is made by BMJ to the Submitting Author unless you are acting as an employee on behalf of your employer or a postgraduate student of an affiliated institution which is paying any applicable article publishing charge ("APC") for Open Access articles. Where the Submitting Author wishes to make the Work available on an Open Access basis (and intends to pay the relevant APC), the terms of reuse of such Open Access shall be governed by a Creative Commons licence – details of these licences and which Creative Commons licence will apply to this Work are set out in our licence referred to above.

Other than as permitted in any relevant BMJ Author's Self Archiving Policies, I confirm this Work has not been accepted for publication elsewhere, is not being considered for publication elsewhere and does not duplicate material already published. I confirm all authors consent to publication of this Work and authorise the granting of this licence.

For peer review only

Article Type

Original research

Title

Clinical similarity in cost-comparison evaluations: a systematic review of current methods in NICE appraisals and the development of a novel framework for the formal assessment of clinical similarity

Steve Edwards[1], Ben Burgess[1], Nicole Downes[1], Sophie Ip[1], Archie Walters[1], Clare Dadswell[1]

Correspondence to Dr Steve Edwards; sedwards@bmj.com

[1]BMJ Technology Assessment Group (BMJ-TAG), BMJ Group, London, UK.

Abstract

Objectives

This research aimed to review how statistically non-significant indirect treatment comparison (ITC) results are interpreted within National Institute for Health and Care Excellence (NICE) cost-comparison evaluations (CCEs) and to develop a novel framework to support the interpretation of such statistically non-significant results from Bayesian network meta-analyses (NMAs).

Methods

A systematic literature review (SLR) was conducted in accordance with Preferred Reporting Items for Systematic Reviews and Meta-Analyses criteria to review NICE CCEs. NICE technology appraisals, with published final guidance, from 2017 to April 2025, that followed the CCE approach from the outset and relied on statistically non-significant ITC results were

For peer review only

included. A narrative synthesis of the results was performed separately for company, External Assessment Group (EAG), and committee perspectives.

A new framework (point-and-density plots) was developed to better interpret statistically non-significant NMA results for NICE CCEs. Point-and-density plots combined elements of forest plots and density plots alongside reporting the probability that a treatment is non-inferior relative to a comparator. As a worked example, point-and-density plots were applied to a recent NICE CCE (TA1019) for crovalimab for patients with Paroxysmal Nocturnal Haemoglobinuria.

Results

Among the 41 included NICE CCEs, EAGs raised concerns about statistically non-significant ITC results while companies relied heavily on them. Only ~32% of CCEs applied formal methods to explore uncertainty in the ITC results (e.g., non-inferiority margins).

For the worked example (TA1019), the NMA results were statistically non-significant with the non-inferiority of crovalimab not demonstrated. However, point-and-density plots indicated there was a 95.9% and 86.3% probability that crovalimab was non-inferior to eculizumab and ravulizumab, respectively.

Conclusion

Inconsistencies in the interpretation of statistically non-significant ITC results exist within individual NICE CCEs and across appraisals. Implementation of both the presented recommendations and framework would eliminate inconsistencies and enhance the robustness and transparency of the CCE pathway.

Registration

The SLR protocol was registered on PROSPERO (CRD420251034143).

For peer review only

Article Summary

Strengths and limitations of this study

The SLR provides insight into company, EAG and committee perspectives on evidence used to support conclusions of clinical similarity within NICE CCEs, with a focus on CCEs associated with the greatest amount of uncertainty.

The inclusion of appraisals that did not use a CCE approach from the start, but introduced these later, may have provided additional insights not currently captured.

The novel framework details how non-inferiority may be clearly, and robustly, assessed in NICE CCEs, addressing a known limitation with this pathway.

Implementation of the novel framework becomes more challenging when non-inferiority margins (or other thresholds) are not available.

A series of recommendations for NICE CCEs, where the results of ITCs are statistically non-significant are provided that, if fully implemented, would substantially improve the robustness and clarity of assessments of clinical similarity within this pathway.

INTRODUCTION

Background

NICE cost-comparison evaluation process

Technology appraisals, such as those performed in England as part of the National Institute for Health and Care Excellence (NICE) technology appraisal programme, involve the assessment of the clinical and cost-effectiveness of specific drugs, technologies or other interventions. As part of the NICE technology appraisal process, independent academic groups, called External Assessment Groups (EAGs), critique company submissions on

For peer review only

behalf of NICE. This critique is considered alongside the company submission in the NICE committees' decision-making process. Complex health economic models are often required to assess whether the intervention of interest is likely to be a good use of NHS resources, with decision-making informed by whether incremental cost-effectiveness ratios (ICERs) resulting from these models are within the range considered an acceptable and effective use of NHS resources and other considerations.[1] However, since 2017, cost-comparison evaluations (CCEs) have been accepted within the NICE technology appraisal programme under specific circumstances.[1-5] CCEs involve a simpler comparison of costs and resources used between interventions, without the need for a complex health economic model such as those used in cost-utility analyses, but they require a strong assumption that the interventions being compared have similar clinical effectiveness and safety.[1, 3]

CCEs were first introduced as one option under the Fast-Track Appraisal (FTA) process in 2017; while this process was replaced in 2022, methods relating to CCEs did not notably change.[1-4, 6] For an appraisal to be suitable for a CCE, new interventions should be, "likely to provide similar or greater health benefits at similar or lower cost than the relevant comparator(s)", with relevant comparators considered to be, "those recommended in published NICE guidance for the same population".[1] The aim of CCEs is to provide an assessment of clinical and cost-effectiveness for suitable interventions that can be used for decision-making but in a shorter time frame and using fewer resources compared to technology appraisals following the standard NICE single technology appraisal (STA) process, as part of NICE's commitment to taking a proportionate approach to technology appraisals.[7]

Challenges within cost-comparison evaluations

While the CCE process aims to improve the efficiency of the NICE technology appraisal process for appraisals that are considered to be a lower risk in terms of cost-effectiveness and whether they are an effective and acceptable use of NHS resources, making

For peer review only

conclusions regarding whether there is sufficient evidence to conclude that a new treatment has “similar” efficacy and safety to a NICE-recommended comparator treatment can be challenging.

Decisions about clinical similarity are often more straightforward within CCEs that include direct evidence from randomised controlled trials (RCTs) for the new treatment against at least one of the NICE-recommended comparators; evidence from non-inferiority, equivalence and superiority RCTs can provide robust evidence to support conclusions of clinical similarity or that the new treatment is considerably better than a relevant comparator treatment.[8] However, NICE CCEs often do not have any direct comparative evidence for any relevant comparators and instead rely on the results of indirect treatment comparisons (ITCs) such as network meta-analyses (NMAs), matching-adjusted indirect comparisons (MAICs), simulated treatment comparisons (STCs) or simple Bucher ITCs.[4, 5, 9]

Conclusions about the clinical similarity of treatments based on ITC results can be challenging given these analyses often generate results that are not statistically significant. While non-significant differences indicate that there is insufficient evidence to conclude that there are statistically significant differences between treatments, it does not prove similarity, non-inferiority or equivalence. Therefore, a lack of statistically significant differences alone should not be used as conclusive evidence that two treatments are similar in terms of clinical outcomes.[10] Furthermore, it is possible that a difference in efficacy between two treatments may be clinically important even where the results are statistically non-significant.[11] Therefore, the interpretation of results from ITCs that indicate no statistically significant differences between treatments is challenging in terms of whether the treatments can be considered clinically similar.

As traditional statistical significance testing is not recommended for assessing clinical similarity, non-inferiority testing is of particular importance for CCEs, with such tests seeking to establish whether a new treatment is ‘not unacceptably’ worse than a comparator. For

For peer review only

non-inferiority testing, an alternative threshold is specified, which is termed a non-inferiority margin (NIM) and represents the maximum reduction in clinical effectiveness that is considered acceptable while still considering the treatments to be equal.[8, 12-14] A pictorial comparison of traditional significance and non-inferiority testing is provided in Figure 1.

Existing guidance and research

The NICE Health Technology Evaluations Manual does not provide any guidance on how clinical similarity can be concluded in situations where ITC results are not statistically significant and it only provides broad guidance on the type of evidence that can be considered or that is preferred; a preference for non-inferiority or equivalence RCTs with appropriate non-inferiority margins and including relevant comparators is expressed where possible, with meta-analysis and ITCs mentioned as alternatives if these sources of data are not available.[1] It also mentions that sufficient certainty is required in conclusions of clinical similarity to appropriate comparators in order for committees to recommend a technology, although no further detail on what constitutes uncertainty is described.[1]

There also appears to be a lack of guidance on this elsewhere; while some discussion of clinical equivalence and non-inferiority is available in a NICE Decision Support Unit document produced by York Health Economics Consortium (YHEC),[15] this is limited to the interpretation of results from equivalence and non-inferiority trials and is not applied specifically to ITCs in this document. Furthermore, on review of all Technical Support Documents developed by the NICE Decision Support Unit, no guidance to support with interpretation of non-significant results from ITCs was identified.[16] This lack of guidance exacerbates the challenges associated with concluding whether treatments can be considered to have similar clinical outcomes based on statistically non-significant ITC results given there is a risk of inconsistent decision-making.

For peer review only

Aims of this research

This research was commissioned by the National Institute for Health and Care Research (NIHR) Evidence Synthesis Programme (NIHR 176061). The project involved a systematic literature review (SLR) stage and a subsequent stage to develop a novel Bayesian framework to aid decision making.

The purpose of the SLR was to review and summarise the basis for decision-makers assuming clinical similarity based on statistically non-significant results obtained from ITCs within NICE CCEs, such as how often minimal clinically important difference (MCIDs) or other approaches are used for interpretation and decision-making. The aim was to gain insight into whether inconsistencies exist in current decisions or whether thresholds for clinical similarity have emerged organically from decision-makers.

After this, the project aimed to develop a novel Bayesian framework to support the interpretation of statistically non-significant results from Bayesian NMAs, with the framework able to integrate MCIDs or other thresholds where available (including any pragmatic thresholds considered appropriate by committees during their deliberations in the absence of established thresholds). Ideally, the goal was for the proposed framework to allow for consistent, robust, and reproducible decision-making for future CCEs.

METHODS

Systematic Literature Review

For the SLR, searches of the main NICE website using “cost comparison”, “cost minimisation” and “cost minimization” terms (including quotation marks) were performed by a single reviewer in April 2025.[17] Within the “Guidance programme” filter, the “technology appraisal guidance” option was selected to refine results. Results from each search were

For peer review only

added to a Microsoft® Excel spreadsheet and duplicate records were removed before screening. In addition, NICE were contacted regarding ongoing CCEs that might be published before completion of the SLR analysis and similar reviews of NICE CCEs were reviewed to identify any additional appraisals not identified through the searches.[2, 3, 18, 19]

The SLR aimed to identify and include all NICE technology appraisals from 2017 onwards that followed the CCE approach from the outset, relied on results from ITCs that were not statistically significant for at least one comparator for the primary outcome(s) and had published final guidance before completion of the SLR. Further details regarding the inclusion and exclusion process, including pragmatic approaches that were required, are provided in Online Supplementary File 1. CCEs identified from the searches were screened against these criteria by a single reviewer, with validation performed by a second reviewer. A single reviewer performed data extraction for each CCE using a standardised data extraction form in Microsoft® Word, with a second reviewer validating a representative 30% sample of extractions. Key information extracted included project name and ID, disease area, primary outcomes, comparators requiring ITCs, type of ITC performed, results of committee-preferred ITCs and whether or not the treatment was recommended as a result of the CCE. Details on the methods used to support decision-making and the rationale for these were also extracted, and any comments made by either the company, EAG or committee relating to the ITC results and interpretation of clinical similarity were extracted from the documents in full to avoid issues with misinterpretation at a later stage. Further information on the data extraction process is outlined in Online Supplementary File 1.

As the SLR includes NICE CCEs, rather than clinical trials for which validated quality assessment tools exist, a risk of bias assessment was not performed within this SLR. A narrative synthesis of results was performed separately for company, EAG and committee perspectives, and an overall comparison between these groups was also considered. Tables were used to record new methods or themes identified from the extracted data and to collate

For peer review only

all CCEs that mentioned each method or theme. Summary tables outlining the key findings have been presented alongside a narrative discussion of findings. The protocol for the SLR was registered on PROSPERO (CRD420251034143) and has been provided in Online Supplementary File 2.[20]

Statistical Framework

The aim of the new framework is to present the results of non-inferiority tests to allow NICE committees to make informed decisions regarding the clinical similarity of treatments, with a particular emphasis on CCEs. Importantly, the framework that has been developed can be viewed as an 'add-on' to existing methods for performing Bayesian NMAs. It is anticipated that this new framework will be of particular use where there is no evidence of a significant difference between a treatment and a comparator.

The statistical framework uses empirical cumulative density functions (ECDFs) to determine the probability that an effect size falls below a given threshold (e.g., NIM). Accordingly, by combining the results of ECDFs with those of traditional density and forest plots, it is possible to convey all three separate analyses within a single readily interpretable figure, which addresses the lack of a specific visualisation for non-inferiority analyses. An example of this figure, termed a point-and-density plot, is shown in Figure 2. In this example, the 95% credible intervals (CrIs) overlap both 0 and the NIM; as such, there is no evidence for a statistically significant difference between the treatment and comparator, while the non-inferiority of the treatment, relative to the comparator, is not demonstrated. However, there is a 95.86% probability that the treatment was non-inferior to the comparator. It is suggested that committees initially focus on whether the 95% CrIs overlap the NIM to determine whether a treatment is non-inferior. However, the use of probabilities of non-inferiority may provide valuable information, particularly where non-inferiority has not been demonstrated.

For peer review only

For some analyses a NIM (or other threshold) may not be available. In such a situation, it is recommended that the corresponding threshold is set to either 0 or 1 (depending on the considered effect size), given that this represents a conservative threshold by which to assess non-inferiority. By using ECDFs, it is possible to determine a hypothetical, non-clinically validated, threshold. Such a hypothetical threshold can be used to determine the required threshold for a given, arbitrary, probability that a treatment is non-inferior to a comparator. It is suggested that an arbitrary 95% probability of non-inferiority may be selected for a hypothetical threshold. In doing so, a 95% probability of non-inferiority aligns with the typical threshold used for statistical significance testing and represents a “high bar” for any assessments of non-inferiority in the absence of a clinically validated NIM.

Full details of the statistical framework are provided in Online Supplementary File 3. Furthermore, R code (and associated data) to generate point-and-density figures for the below case study are provided as supplementary files 4 and 5.

Case study

To showcase how this novel framework may be used within CCEs, a case study is presented where the framework has been applied to data presented in a recent CCE. NICE TA1019 assessed crovalimab for the treatment of paroxysmal nocturnal haemoglobinuria (PNH) in people 12 years and over,[21] with crovalimab subsequently being approved for use by NICE in line with its marketing authorisation. TA1019 compared crovalimab to the two treatments currently used to treat PNH, namely eculizumab and ravulizumab. Within the final draft guidance, NICE stated that the clinical trial evidence indicated that crovalimab works as well as eculizumab and is likely to be as effective as ravulizumab.[22]

Within TA1019, the company presented Bayesian NMAs for the primary co-endpoint from the COMMODORE 1 and 2 trials of transfusion avoidance (but not the primary co-endpoint of haemolysis control),[23, 24] which was assessed using the endpoint of mean difference.[21]

For peer review only

Furthermore, the company stated that a NIM of 0.20 was considered based on data from a previous clinical trial.[21] The company provided the results for random-effects (RE) Bayesian NMAs for the overall population, patients who had previously received complement inhibitors (experienced subgroup), and patients who had not previously received complement inhibitors (naive subgroup).[21]

For the case study, the Bayesian RE NMA for the overall population was re-analysed using data provided in the company's submission for TA1019. As such, the results of the company's submission are able to be compared with those from the re-analysis. Within the company's submission, the company stated that meaningfully informative priors were used to estimate between-study heterogeneity; however, the company did not provide these informative priors in the submission.[21] As such, any differences in the results of the original analysis and the equivalent analysis presented here are likely to be attributed, at least in part, to a difference in priors with uninformative priors used in the re-analysis.

Patient and public involvement

There was no patient or public involvement in this research.

RESULTS

Systematic Literature Review

Search and screening results

A total of 91 individual records of NICE technology appraisals were identified and screened for inclusion in this SLR, with 41 appraisals ultimately included. A full PRISMA diagram is presented in Online Supplementary File 1. A range of disease areas were covered by the included appraisals, but some were more commonly represented than others. In terms of

For peer review only

ITCs, over 60% relied solely on NMAs, with smaller percentages using other ITC methods. While all appraisals relied on ITC evidence for at least one comparator, ~25% had direct trial evidence for another comparator relevant to the appraisal. Details of all included appraisals and a list of excluded appraisals are provided in Tables 1 and 2 of Online Supplementary File 1, respectively.

Themes identified

Narrative syntheses of company, EAG and committee approaches to the interpretation of statistically non-significant ITC results and additional evidence sources considered are summarised in Tables 3 to 5 of Online Supplementary File 1. An overall synthesis of findings across these groups is discussed narratively below, with summaries presented in Tables 1 and 2.

The key finding was a lack of consistency in terms of methods used to interpret statistically non-significant ITC results and whether these were considered sufficient evidence of clinical similarity. While inconsistency existed across appraisals, EAGs tended to raise concerns about relying solely on these results as evidence of clinical similarity and companies usually placed a strong emphasis on the lack of statistically significant differences. EAGs sometimes concluded that there was too much uncertainty within ITCs for a CCE to be appropriate, while others considered supplementary information such as similar mechanisms of action, clinical expert feedback, previously published ITC results or NICE appraisals and/or naive comparative data to alleviate these concerns.

More formal methods of exploring uncertainty were sought by some EAGs and/or companies, including the application of MCIDs or NIMs and/or presentation of probabilities based on Surface Under the Cumulative RAnking curves (SUCRAs) from NMAs. However, one or more of these approaches were only explored in a minority (~32.0%) of appraisals. Of

For peer review only

note, no insight into committee preferences regarding which methods are most useful in terms of interpreting these results was identified from the appraisals, but committees frequently made use of clinical expert feedback and information on mechanisms of action where uncertainty was noted.

Two more general themes relating to CCEs were also identified, including improving the transparency of the decision-making process within committee discussions and clarifying whether only one relevant comparator outlined in the NICE final scope needs to be covered. These are summarised in Table 6 of the Online Supplementary File 1; they are not addressed by the framework described in this publication but were considered to be useful findings that may be addressed through updated guidance or reporting of committee discussions by NICE.

For peer review only

Table 1. Overview of methods used to interpret statistically non-significant ITC results

Method	Company	EAG	Committee
No statistically significant differences	Some relied solely on a lack of statistically significant differences but most also discussed the direction/size of the point estimate. Companies generally did not acknowledge the uncertainty of statistically non-significant ITC results.	EAGs often considered statistically non-significant differences with or without consideration of point estimate size/direction to be insufficient. Limitations associated with statistically non-significant results from ITCs were often noted.	No clear position on whether statistically non-significant results from ITCs were sufficient for decision-making was identified.
Point estimate direction and/or size	Companies often heavily relied on point estimate direction/size. Some suggested that point estimates favouring the intervention for some analyses and the comparator for others was evidence of no consistent treatment difference. Some also suggested that clinical similarity was a conservative assumption where point estimates favoured the intervention.	Limitations of non-significant differences from ITCs were often highlighted, often noting that the inferiority of the new treatment could not be ruled out. However, many did mention them as supportive evidence and were reassured by review of other evidence or the robustness of the results to alternative analyses.	No clear position on the interpretation of point estimate direction and/or size was identified in most appraisals but point estimates close to the line of null effect in one appraisal reduced the committees concerns about uncertainty raised by the EAG.
Some statistically significant benefits of the new intervention	In some appraisals, statistically significant differences were identified from ITCs for some but not all analyses. Companies tended to consider this evidence that clinical similarity was conservative.	No strong EAG conclusions regarding these were noted. Some statistically significant benefits may have increased EAG confidence of clinical similarity, but concerns may have remained.	No explicit opinion from committees on this was identified within this SLR, but it may have provided some reassurance.
Probabilities calculated from NMAs	Probabilities from NMAs were mentioned in ~15% of company submissions. However, not all used them to support their conclusions. Most involved SUCRA information from NMAs but two combined this with a NIM or clinical equivalence threshold, either from the outset or following a request from the EAG.	EAGs requested these methods from companies, suggested adaptations to company approaches or noted that they would have reduced uncertainty in ~17% of appraisals. Simpler probability ranks and calculating probabilities of falling within a clinical equivalence range were noted as options.	No explicit opinion from committees on the application of this method for interpreting ITC results, and whether it would increase committee confidence for decision-making, was identified within this SLR.
MCIDs or other thresholds	Companies in ~10% appraisals incorporated MCIDs or NIMs. Thresholds were used either to assess whether point estimates represented a meaningful difference, or incorporated into an NMA to calculate probabilities of clinical equivalence.	EAGs (~17% of appraisals) used or adapted company threshold-based approaches or indicated that they would have been useful. Application of thresholds to assess whether ITC results were clinically meaningful or within NMAs to calculate probabilities were mentioned.	No explicit opinion on the application of this method for interpreting ITC results, and whether it would increase committee confidence for decision-making, was identified within this SLR.

Abbreviations: EAG, External Assessment Group; ITC, indirect treatment comparison; MCID, minimal clinically important difference; NIM, non-inferiority margin; NMA, network meta-analyses; SLR, systematic literature review; SUCRA, Surface Under the Cumulative RAnking curve.

For peer review only

Table 2. Overview of sources of supplementary evidence used to support conclusions of clinical similarity

Method	Company	EAG	Committee
Similar mechanisms of action	Similar mechanisms of action were mentioned in ~15% of appraisals as supportive evidence that outcomes of the treatments were likely to be similar.	While not always explicitly used as supportive evidence for conclusions, ~34% of EAGs commented on the similarity of mechanisms of action between the intervention and comparators.	Committee discussions often mentioned whether mechanisms of action are similar between treatments, and in ~7% of appraisals, this may have reduced uncertainty and enabled conclusions of clinical similarity.
Clinical expert feedback	Clinical expert feedback was mentioned by companies in ~24% of appraisals, either based on clinician experience with the treatments or anticipated differences, or clinician review of ITC results to determine whether differences were clinically meaningful or not.	EAGs mentioned clinical expert feedback in ~34% of appraisals, including expert feedback that there are no differences between treatments based on their experience, that no differences were anticipated based on their knowledge of the treatments and/or support with interpretation of results, such as thresholds for clinically meaningful differences.	Committee discussions did not routinely include statements about clinical expert opinion. However, where EAGs raised uncertainty with ITC results, this was cited as a reason that the committee was ultimately able to conclude clinical similarity in ~17% of all included appraisals.
Evidence of a class effect from meta-analyses	Some companies highlighted previously published meta-analyses of the intervention and comparators against a common comparator (e.g. placebo) as supportive evidence of a class effect by some companies.	In two cases where the company suggested this, it was rejected by the EAG as a robust source of evidence, although it appeared to be of some use to the EAG in the third appraisal.	This was mentioned in the committee discussion of one of these appraisals, but there was no indication of whether it was considered useful to the committee.
Previous ITC results or NICE appraisals	Existing ITCs and/or NICE appraisals were cited in four appraisals, with results said to be similar to the current appraisal or decisions made based on a similar level of uncertainty to a previous appraisal.	These were mentioned by EAGs in a similar number of appraisals, including references to similar results being obtained or a similar level of uncertainty within ITC results that had not precluded a conclusion of clinical similarity.	These were generally not mentioned in documents summarising committee discussions.
Real-world data	One company had SACT data available, which was used as supportive evidence alongside ITC results.	The EAG also made use of these data when making its conclusions.	These data were not mentioned in the committee discussion of the evidence.
Other EAG sources	NA	Naïve comparative evidence (for example, a visual comparison of KM curves from each study or statistical testing of differences) was used by some EAGs.	This was mentioned in some committee discussions, suggesting it was of some use, but was not mentioned in others.

Abbreviations: EAG, External Assessment Group; ITC, indirect treatment comparison; KM, Kaplan-Meier; NICE, National Institute for Health and Care Excellence; SACT, Systemic Anti-Cancer Therapy.

For peer review only

Statistical Framework

A recent CCE, TA1019,[21] assessing crovalimab in patients with PNH, was used as a case study to illustrate the described novel framework. The SLR performed by the company for TA1019 identified five trials reporting data on transfusion avoidance in people with PNH, which formed a star-shaped network (Figure 5, Online Supplementary File 3). Two trials compared ravulizumab to eculizumab, two trials compared crovalimab to eculizumab, and one trial compared eculizumab to standard of care (Table 1, Online Supplementary File 3).

The results of the RE NMAs from TA1019 and the case study re-analysis are presented in Figure 3. Within the CS,[21] the company noted that the results of this NMA indicated that there was no significant evidence for a difference between crovalimab and both eculizumab and ravulizumab as the 95% CrIs overlapped 0 for both comparisons. However, the 95% CrIs for the comparison of crovalimab to ravulizumab overlapped the NIM of 0.20 (i.e., 20%), while the 95% CrIs for the comparison of crovalimab to eculizumab did not. Accordingly, crovalimab would be considered to be non-inferior to eculizumab but non-inferiority was not demonstrated compared to ravulizumab, although this was not explicitly stated in the company's submission. However, the company stated in text, that there was a 99% and 93% probability of crovalimab being non-inferior to eculizumab and ravulizumab, respectively, when considering the NIM of 0.20.[21] For the case study reanalysis, the point estimates for each of the comparisons to crovalimab align with those from the analysis in the company's submission. However, the 95% CrIs intervals associated with each point are much wider than those in the original analysis. As previously noted, this is expected to be due to the use of informative priors in the company's original analysis.

Results of the RE NMAs from the case study reanalysis were additionally analysed using the new statistical framework with point-and-density plots shown for the comparisons of crovalimab to eculizumab and crovalimab to ravulizumab (Figure 4). As shown by the point-

For peer review only

and-density plots, there was a relatively high probability of crovalimab being non-inferior compared to both ravulizumab (86.27%) and eculizumab (95.87%). Additionally, through the use of ECDFs it was possible to determine that a hypothetical threshold of 0.31 would be required in order for there to be a 95% probability that the point estimate, for the comparison of crovalimab to ravulizumab, was below this hypothetical threshold.

Within the re-analysis, the non-inferiority of crovalimab relative to both eculizumab and ravulizumab was not statistically demonstrated. The point-and-density figures illustrated that there was a high probability that the difference in treatment effects would be considered non-inferior for comparisons of crovalimab to both eculizumab and ravulizumab. Accordingly, the results of the re-analysis for the overall population support the use of a cost-comparison for TA1019.

DISCUSSION

Systematic Literature Review

The key finding from the SLR was inconsistency with regard to how statistically non-significant differences from ITCs were interpreted within CCEs; companies frequently interpreted these as sufficient evidence for clinical similarity, while EAGs generally seem to be aware of the importance of not misinterpreting the absence of a statistically significant difference as evidence of clinical similarity. Methods emerging from the current appraisals to further explore these ITC results include specific tests of non-inferiority utilising a threshold such as a clinically derived NIM or MCID. However, only a minority of CCEs in this SLR made use of such methods. Furthermore, additional detail within committee discussions would improve the transparency of decision-making in these appraisals, given it was often limited, and clarity regarding how many comparators need to be covered from the NICE final scope would be beneficial for companies and EAGs.

For peer review only

For peer review only - http://bmjopen.bmj.com/site/about/guidelines.xhtml

Findings from the SLR are largely in line with a recent publication by PenTAG, an EAG working on NICE appraisals, which also raised concerns about relying on statistically non-significant ITC results in CCEs and made recommendations on methods that may improve transparency in decision-making.[19] However, another EAG recently concluded that a lack of statistically significant differences or overlapping 95% confidence intervals may be sufficient to demonstrate clinical similarity in CCEs.[25] Therefore, the lack of guidance in this area may also be contributing to differences in perspectives across EAGs, and work on methods that can support the interpretation of statistically non-significant ITC results may improve the consistency and transparency of decision-making within CCEs.

Strengths of this SLR include the focus on CCE appraisals relying on non-significant ITC results; given there is more uncertainty associated with this type of evidence, this has allowed the review to focus specifically on methods being used in situations where there is the highest uncertainty in conclusions and the greatest need to identify methods to address this. Furthermore, a thorough review of company, EAG and committee perspectives has been performed, allowing the identification of similarities and inconsistencies in approaches across appraisals and also between companies, EAGs and committees. A potential limitation of this SLR is that keywords were used to search the NICE website rather than reviewing the full list of NICE technology appraisals, but on review of appraisals included in the work by PenTAG (which used the latter approach), the researchers are reassured that this approach did not lead to many relevant appraisals being missed.[19] Furthermore, inclusion of appraisals that did not use a CCE approach from the start but introduced them later in the process was not possible due to limited resources; it is possible that these types of appraisals may have provided useful additional information but the researchers consider it unlikely that the findings of this SLR would change considerably if these appraisals had been considered.

For peer review only

Statistical Framework

There is currently uncertainty, from pharmaceutical companies, regarding how NICE assesses equivalence, or non-inferiority, within NMAs and other forms of ITCs.[19] As such, the novel framework presented here provides a clear outline for how non-inferiority may be assessed within CCEs that utilise NMAs. Accordingly, a key strength of the newly developed framework is that it addresses a known challenge that NICE committees face when evaluating CCEs, namely, how to assess clinical similarity when the results of NMAs indicate that there is no evidence for a statistically significant difference. As shown by the results of the SLR, there are no methods or frameworks that are consistently applied in CCEs, that utilise NMAs, to assess clinical similarity.

Within this newly presented framework, specifically through the point-and-density plots, an approach has been provided that is readily interpretable and provides information that was previously unavailable, to allow NICE committees to draw robust conclusions as to whether a new treatment is non-inferior to an existing comparator. Crucially, when non-inferiority, or inferiority, has not been demonstrated in a direct comparison within a randomised controlled trial, the framework presents the probability that the new treatment is non-inferior to an existing comparator. As such, this allows NICE committees to make informed decisions on the likelihood of a new treatment being clinically similar to an existing comparator, even when non-inferiority has not been statistically demonstrated.

Furthermore, a recent review of CCEs and methodological approaches that are used to assess non-inferiority reported that visualisations to specifically assess non-inferiority were scarce and that where such visualisations were provided, the most common approach was to present forest plots with an overlaid NIM.[19] As such, NIMs may be viewed as being “shoehorned” into existing visualisations with little consideration of the most appropriate manner of displaying such information.[19] Accordingly, the new framework proposed here

For peer review only

helps to overcome this limitation through the development of point-and-density plots that specifically seek to display the results of NIMs in a dedicated, intuitive manner.

As has been previously discussed, the majority of CCEs do not provide a NIM, MCID, or another threshold to allow for a statistically robust assessment of non-inferiority. As a consequence, clinical similarity has typically been erroneously assessed through inappropriate statistical approaches.[25] However, there is disagreement between EAGs regarding what constitutes an appropriate method to assess clinical similarity within CCEs.[19, 25]

Where a NIM or MCID is not available, the new framework presented here allows for a 'hypothetical threshold' to be calculated which refers to the required value of a NIM in order for an arbitrary probability (e.g., 95%) that a treatment is non-inferior to a comparator. Such an approach is a strength of the new framework and provides further context to EAGs and committees with regards to the likelihood of a new treatment being non-inferior, relative to an existing comparator, when such information would not otherwise be available. Although it is possible to calculate a 'hypothetical threshold' where a NIM, MCID, or other threshold has not been provided, it is strongly recommended that this approach should not be considered a substitute for the provision of a clinically validated NIM or MCID. Indeed, it is recommended that a company should provide a clinically validated NIM or MCID for all primary, or co-primary, outcomes in order for an appraisal to be considered for the CCE pathway. The absence of a clinically validated NIM or MCID severely limits the ability of a committee, or EAG, to reach definitive conclusions as to whether a new treatment is non-inferior, or clinically similar, to a previously approved comparator.

Finally, within this framework, a 95% threshold for assessing non-inferiority has been recommended. The researchers acknowledge that this threshold represents a "high bar" for assessing non-inferiority and has been arbitrarily chosen to align with traditional statistical

For peer review only

significance testing. As such, if this framework is implemented in CCEs, committees and EAGs may wish to explore an alternative threshold for assessing non-inferiority.

Recommendations

A number of recommendations, that cover all aspects of the approach to assess clinical similarity within CCEs, resulting from both the SLR (Recommendations 1 to 6) and the novel framework (Recommendations 7 to 10) have been suggested. As such, NICE CCEs would be methodologically, and clinically, strengthened if all of these recommendations were to be adopted.

1. Use of MCIDs or NIMs should be considered to support the interpretation of statistically non-significant results from ITCs. Where these are not available or cannot be estimated robustly, clinical expert advice could be sought to estimate pragmatic thresholds for the purpose of the appraisal.
2. The incorporation of thresholds (e.g., NIMs or MCIDs) into Bayesian analyses to obtain probabilities of the new treatment being no worse than one or more established comparators with existing NICE guidance would be a robust method of using such thresholds. When multiple comparators exist, presentation as pairwise comparisons against each comparator may be useful given that committees evaluate the evidence to support a CCE against each comparator separately. Where a suitable threshold has not been identified, even with clinical expert input, probabilities of being the best treatment within a Bayesian network or being better than each comparator may be useful for decision-making purposes. When a Bayesian analysis is not possible, other methods of using MCIDs or NIMs to support the interpretation of results should be considered;

For peer review only

3. Guidance related to the uncertainties of statistically non-significant ITC results within the NICE methods manual or user guides for submissions may be useful to improve the case put forward in company submissions and provide support for EAGs in terms of what information or methods might be reasonable to request. This may also improve consistency across EAGs in terms of how statistically non-significant ITC results are interpreted, given the different positions noted in recent publications by EAGs in this area.[19, 25] This guidance need not be prescriptive but could simply highlight the limitations of relying on statistically non-significant differences from ITCs and that additional methods may be useful to support conclusions of clinical similarity;

4. Supportive information such as clinical expert feedback, comments on the similarity of mechanisms of action and consideration of naïve comparative evidence should not be considered sufficient to resolve uncertainty associated with statistically non-significant ITC results. Statistically robust approaches should be explored to improve confidence in conclusions of clinical similarity based on ITC results, with supportive information used to supplement these conclusions;

5. Additional detail regarding committees' interpretation of statistically non-significant ITC results within published NICE guidance for each appraisal may improve transparency within the decision-making process, particularly where there is discordance between overall conclusions made by the EAG and the committee;

6. Clarity on whether or not a CCE needs to make a case against multiple or only one relevant comparator within the NICE CCE process may be useful. Explanation of when a single comparator may be deemed appropriate within the NICE process and whether companies should still explore all or multiple comparators in their original submission might address this uncertainty;

7. CCEs should implement a consistent framework to support the interpretation of statistically non-significant results from NMAs. Through utilisation of point-and-density plots, committees and EAGs will be presented with results in a consistent format, while all stakeholders will have clarity regarding how clinical similarity is assessed.

8. When an established NIM or MCID is used, it is recommended that a probability of 95% is used to determine if a new treatment can be considered to be ‘clinically similar’ to a comparator. While this is an arbitrary value, it aligns with the typical threshold for statistical significance in traditional hypothesis testing and represents an appropriately high bar to assume clinical similarity between treatments;

9. When a NIM or MCID is unavailable, it is recommended that a value representing no difference is used to assess clinical similarity, given that this represents a conservative estimation of non-inferiority;

10. In exceptional circumstances, a committee may want to use an estimated hypothetical threshold that is required for a treatment to be considered “clinically similar” to a comparator. In doing so, this approach would allow the committee to qualitatively determine how different the estimated results are from this theoretical threshold.

For peer review only

CONCLUSIONS

NICE requires that CCEs demonstrate that new interventions are clinically similar to relevant comparators already approved by NICE. Here, the SLR sought to provide an overview of the current approaches that are used within CCEs with statistically non-significant ITC results to conclude clinical similarity. Furthermore, the newly developed Bayesian framework sought to provide EAGs and NICE committees with additional insights regarding clinical similarity.

Overall, this research provides vital information that may result in better-informed decision-making when assessing CCEs.

The SLR determined that assessments of clinical similarity were not consistent in the identified CCEs. Companies frequently interpreted statistically non-significant differences as evidence for clinical similarity, while EAGs often correctly refuted these assertions. Importantly, an absence of a statistically significant difference between two treatments is not evidence of clinical similarity. Instead, clinical similarity should be assessed through specific tests of non-inferiority that utilise a clinically relevant threshold (e.g., NIMs or MCIDs). The SLR indicated that 75% of CCEs did not use such approaches. Furthermore, there are some uncertainties regarding how NICE committees determine whether a new treatment is considered clinically similar to a comparator. For instance, it is unclear what methods NICE considers acceptable when relying on statistically non-significant differences from ITCs and whether clinical similarity is required to be demonstrated for an intervention relative to one, or all, relevant comparators.

The novel framework was developed to overcome several limitations of the current approach to assessing clinical similarity in the CCE pathway. Specifically, the framework represents a consistent and rigorous approach to assessing clinical similarity where the results of a Bayesian NMA are statistically non-significant. If this framework were to be widely implemented within CCEs, it would eliminate the current methodological inconsistencies in how clinical similarity is assessed, while also providing clarity to all stakeholders involved in the process. Accordingly, point-and-density plots are easily understandable through the combination of components from multiple commonly used figure types. Following the completion of the SLR and development of the novel framework, the researchers have made a number of recommendations which, if all were to be implemented, would enhance the robustness and transparency of the NICE CCE pathway.

For peer review only

Word Count: 5443 words

Data availability statement

Additional information on the methods and results of this research has been provided in the online supplementary files.

Funding statement

This research was commissioned by the National Institute for Health and Care Research (NIHR) Technology Assessment Reviews programme (NIHR 176061).

Acknowledgements

The authors thank members of the NICE team for responding to requests for information about completed and ongoing cost-comparison evaluations.

Competing interests

No competing interests were declared which affect the impartiality of this report. BMJ Technology Assessment Group (BMJ-TAG) and the editorial team of BMJ Group work independently to one another. The views and opinions expressed in this report are those of the BMJ-TAG.

For peer review only

Author contributions

SE was responsible for the conception of the research project and provision of research framework, provided guidance and validation of the work of the research group at all stages including all versions of the protocol, report and publication.

BB led on the development of the Bayesian framework for a formal assessment of clinical similarity, tested the developed framework using examples identified through the SLR and drafted sections of the report and publication related to the Bayesian framework.

ND led the development of the protocol for the SLR phase of this research, was the lead reviewer on the SLR, including development of search strategy, running searches, sifting of results, data extraction and narrative synthesis of results, and drafted sections of the report and publication related to the SLR.

SI supported during the Bayesian framework development stage by validating code and analyses.

AW was the second reviewer on the SLR phase of this research and was involved in the validation of the screening stage and a proportion of the extractions.

CD contributed to the development of the project and was available to provide feedback to resolve any outstanding conflict between the first two reviewers on the SLR.

For peer review only

Figure captions

Figure 1. Illustration of traditional statistical significance testing (A) and non-inferiority testing (B).

Footnote: Points denote effect sizes, while lines denote 95% credible intervals. Dashed lines correspond to the thresholds of the null and alternative hypotheses. For traditional significance testing the dashed line corresponds to either 0 or 1 (depending on the given effect size). For non-inferiority testing the dashed line corresponds to the non-inferiority margin. Areas without shading correspond to the alternative hypothesis, areas with red shading correspond to the null hypothesis. Red diamonds denote effect sizes where the null hypothesis was accepted.

Black circles denote effect sizes where the null hypothesis was rejected. Grey squares denote effect sizes where there is insufficient evidence to reject the null hypothesis.

Figure 2. Point-and-density plot for the comparison of an example treatment and comparator.

Footnote: The threshold for traditional hypothesis testing is shown by the solid line, while the dashed line indicates the NIM. The area that falls under (above) the NIM is shown in blue (orange). The probability that the effect size falls under, or above, the NIM is shown in the upper left corner of the plot. The pooled estimate is shown by the black circle, with the error bars corresponding to 95% CrIs.

Figure 3. Results of the random-effects network meta-analyses, for the percentage of patients achieving transfusion avoidance in the overall population, presented by the company[21] and as obtained from the case study re-analysis.

Footnote: Effect size estimates with black circles and red diamonds are from the analyses presented in TA1019 and the case study reanalysis, respectively.

Figure 4. Point-and-density plots for the comparisons of eculizumab to crovalimab and ravulizumab to crovalimab for the percentage of patients achieving transfusion avoidance in the overall population.

References

1. National Institute for Health and Care Excellence (NICE). NICE health technology evaluations: the manual, 2023. Available from: https://www.nice.org.uk/process/pmg36. Date accessed: Apr 25.
2. Thurgar E. The Evolution of Cost Comparison at NICE: What Can We Learn from Fast-Track Appraisals? 2022. p. https://www.ispor.org/docs/default-source/euro2022/thurgar-e-1-pdf.pdf?sfvrsn=494aa432_0. ISPOR Europe; 6-9 November Vienna, Austria
3. Armand J, Fontrier AM, Sawyer L. Cost-comparison, an easier route to NICE recommendations? 2023. p. https://www.ispor.org/docs/default-source/euro2023/isporeurope23armandhta357poster132726-pdf.pdf?sfvrsn=12a50a55_0. ISPOR Europe; 12-15 November; Copenhagen, Denmark.
4. Astill J, Roberts M, Cooper C. Overview of NICE Cost-Comparison Technology Appraisals: Key Takeaways Regarding Comparator Selection and Methods Used to Demonstrate Comparable Efficacy. 2024. p. https://www.ispor.org/docs/default-source/euro2024/isporeu24-hta194-astill143867-pdf.pdf?sfvrsn=c750ae89_0. ISPOR Europe; Barcelona, Spain.
5. Haycock M, Willows L, Wickstead R. Demonstrating Similar or Greater Health Benefits Based on Indirect Evidence: A Review of NICE Evaluations that Included a Cost-Comparison Approach. 2023. p. HTA34. ISPOR Europe; Copenhagen, Denmark.
6. National Institute for Health and Care Excellence (NICE). Technology appraisal methods and processes used before 2022, 2022. Available from: https://www.nice.org.uk/about/what-we-do/our-programmes/nice-guidance/nice-technology-appraisal-guidance/process. Date accessed: Apr 25.

For peer review only

7. National Institute for Health and Care Excellence (NICE). Taking a proportionate approach to technology appraisals, 2024. Available from: https://www.nice.org.uk/about/what-we-do/proportionate-approach-to-technology-appraisals. Date accessed: Apr 25.
8. Walker E, Nowacki AS. Understanding equivalence and noninferiority testing. *J Gen Intern Med* 2011; **26**: 192-6.
9. Thiam A, Noon K, Micallef J, Porteous A. HTA151 The NICE Cost Comparison Pathway - How Much Evidence Is Required to Meet the Criteria? *Value in Health* 2022; **25**: S326.
10. Gewandter JS, McDermott MP, Kitt RA, Chaudari J, Koch JG, Evans SR, et al. Interpretation of CIs in clinical trials with non-significant results: systematic review and recommendations. *BMJ Open* 2017; **7**: e017288.
11. Esterhuizen TM, Mbuagbaw L, Thabane L. Disparity between statistical and clinical significance in published randomised controlled trials indexed in PubMed: a protocol for a cross-sectional methodological survey. *BMJ Open* 2024; **14**: e084375.
12. Schumi J, Wittes JT. Through the looking glass: understanding non-inferiority. *Trials* 2011; **12**: 106.
13. Fleming TR, Odem-Davis K, Rothmann MD, Li Shen Y. Some essential considerations in the design and conduct of non-inferiority trials. *Clinical Trials* 2011; **8**: 432-9.
14. Hashim M, Vincken T, Kroi F, Gebregergish S, Spencer M, Wang J, et al. A systematic review of noninferiority margins in oncology clinical trials. *Journal of comparative effectiveness research* 2021; **10**: 443-55.
15. York Health Economics Consortium (YHEC) - NICE Decision Support Unit. Clinical Equivalence and Non-Inferiority - Draft Report, 2020. Available from: https://yhec.co.uk/wp-content/uploads/2021/01/Clinical-Equivalence-and-Non-Inferiority-Report-Final-21Feb2020-2.pdf. Date accessed: May 25.
16. University of Sheffield - NICE Decision Support Unit. Full list of technical support documents (TSDs), 2025. Available from: https://www.sheffield.ac.uk/nice-dsu/tsds/full-list. Date accessed: May 25.
17. National Institute for Health and Care Excellence (NICE). 2025. Available from: https://www.nice.org.uk/. Date accessed: Aug 25.
18. Lee D, Allen A, Lovell A, Abdelsabour A, Wilson ECF, Melendez-Torrez GJ. How similar is similar enough? Assessment of indirect treatment comparisons to support similarity for NICE's cost comparison route. 2024. p. https://www.ispor.org/docs/default-source/euro2024/how-similar-is-similar-enough-cost-comparison-poster-ispor142603-pdf.pdf?sfvrsn=21e7eb7b_0. ISPOR Europe; Barcelona, Spain.
19. Lee D, Allen A, Lovell A, Abdelsabour A, Wilson ECF, Melendez-Torres GJ. How similar is similar enough? Assessment of indirect treatment comparisons to support similarity for NICE's cost comparison route. *Value in Health* 2025.
20. Edwards S, Dadswell C, Downes N, Burgess B, Walters A, Ip S. Evaluating clinical similarity: a systematic review of NICE guidance and the development of a Bayesian framework for a formal assessment of clinical similarity. PROSPERO 2025 CRD420251034143., 2024. Available from: https://www.crd.york.ac.uk/PROSPERO/view/CRD420251034143. Date accessed: Aug 25.
21. National Institute for Health and Care Excellence (NICE). Crovalimab for treating paroxysmal nocturnal haemoglobinuria in people 12 years and over - Technology appraisal guidance [TA1019], 2024. Available from: https://www.nice.org.uk/guidance/ta1019. Date accessed: Apr 25.
22. National Institute for Health and Care Excellence (NICE). Crovalimab for treating paroxysmal nocturnal haemoglobinuria in people 12 years and over - Technology appraisal guidance [TA1019] - Final draft guidance, 2024. Available from: https://www.nice.org.uk/guidance/ta1019/history. Date accessed: Apr 25.
23. Röth A, He G, Tong H, Lin Z, Wang X, Chai-Adisaksopha C, et al. Phase 3 randomized COMMODORE 2 trial: Crovalimab versus eculizumab in patients with

For peer review only

paroxysmal nocturnal hemoglobinuria naive to complement inhibition. *Am J Hematol* 2024; **99**: 1768-77.
24. Scheinberg P, Clé DV, Kim JS, Nur E, Yenerel MN, Barcellini W, et al. Phase 3 randomized COMMODORE 1 trial: Crovalimab versus eculizumab in complement inhibitor-experienced patients with paroxysmal nocturnal hemoglobinuria. *Am J Hematol* 2024; **99**: 1757-67.
25. Poley MJ, Armstrong N, Yang H, Patel M, Stirk L, Al MJ, et al. Cost Comparisons in NICE Technology Appraisals: An External Assessment Group Perspective. *PharmacoEconomics* 2025.

For peer review only

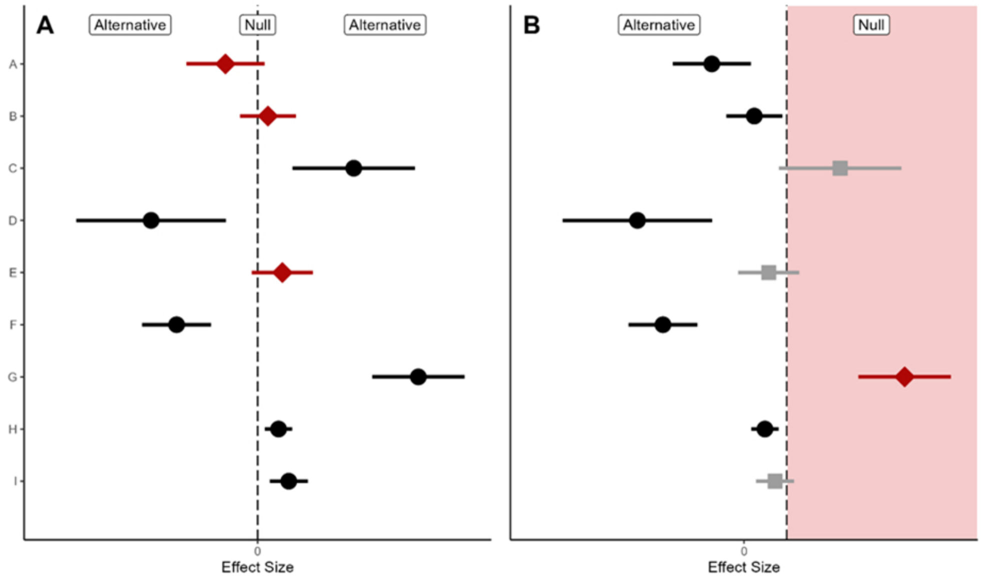

Caption: Figure 1. llustration of traditional statistical significance testing (A) and non-inferiority testing (B).

Footnote: Points denote effect sizes, while lines denote 95% credible intervals. Dashed lines correspond to the thresholds of the null and alternative hypotheses. For traditional significance testing the dashed line corresponds to either 0 or 1 (depending on the given effect size). For non-inferiority testing the dashed line corresponds to the non-inferiority margin. Areas without shading correspond to the alternative hypothesis, areas with red shading correspond to the null hypothesis. Red diamonds denote effect sizes where the null hypothesis was accepted. Black circles denote effect sizes where the null hypothesis was rejected. Grey squares denote effect sizes where there is insufficient evidence to reject the null hypothesis.

404x243mm (59 x 59 DPI)

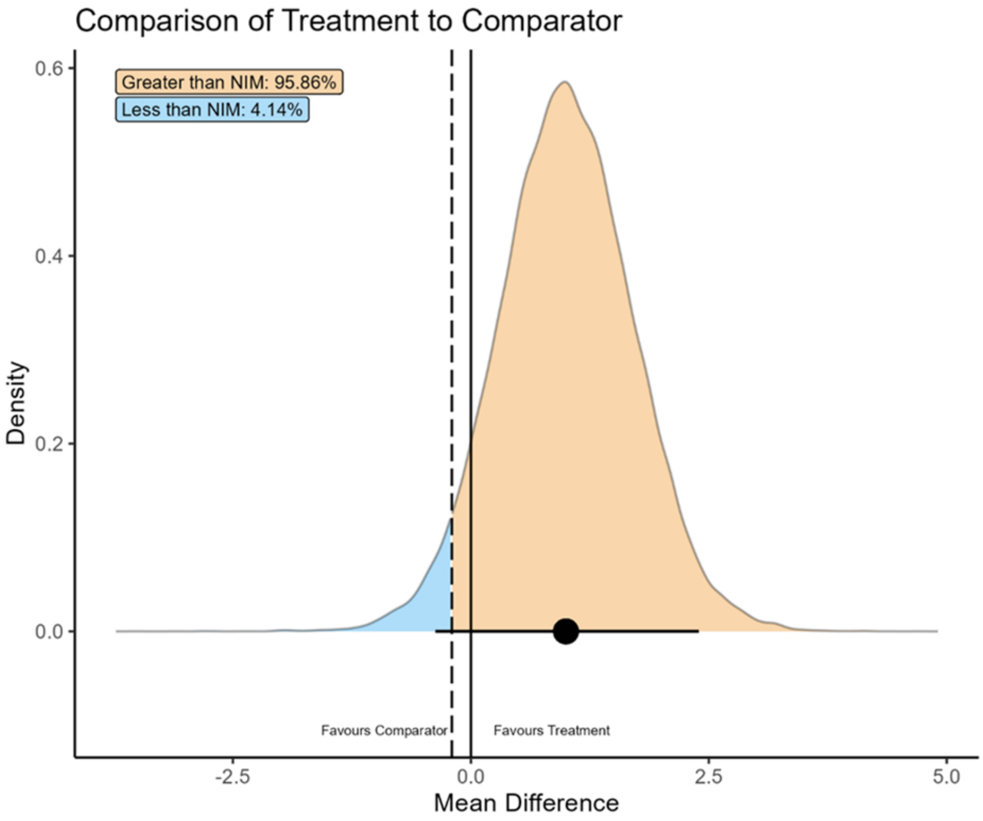

Caption: Figure 2. Point-and-density plot for the comparison of an example treatment and comparator.

Footnote: The threshold for traditional hypothesis testing is shown by the solid line, while the dashed line indicates the NIM. The area that falls under (above) the NIM is shown in blue (orange). The probability that the effect size falls under, or above, the NIM is shown in the upper left corner of the plot. The pooled estimate is shown by the black circle, with the error bars corresponding to 95% CrIs.

404x337mm (59 x 59 DPI)

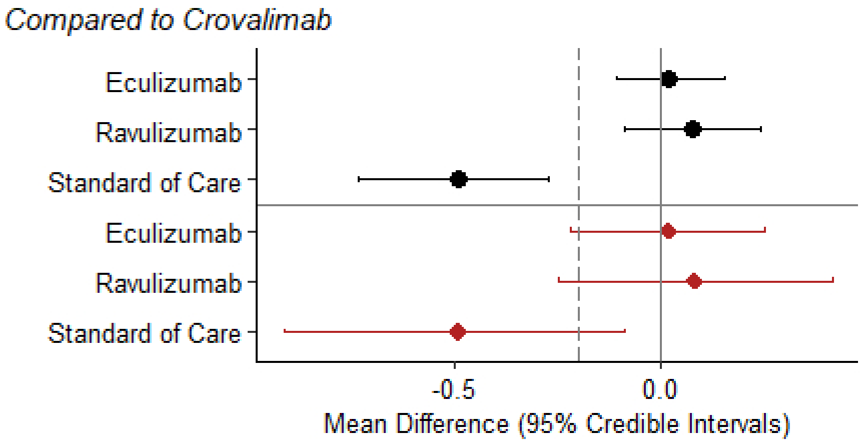

Caption: Figure 3. Results of the random-effects network meta-analyses, for the percentage of patients achieving transfusion avoidance in the overall population, presented by the company and as obtained from the case study re-analysis.

Footnote: Effect size estimates with black circles and red diamonds are from the analyses presented in TA1019 and the case study reanalysis, respectively.

295x154mm (38 x 38 DPI)

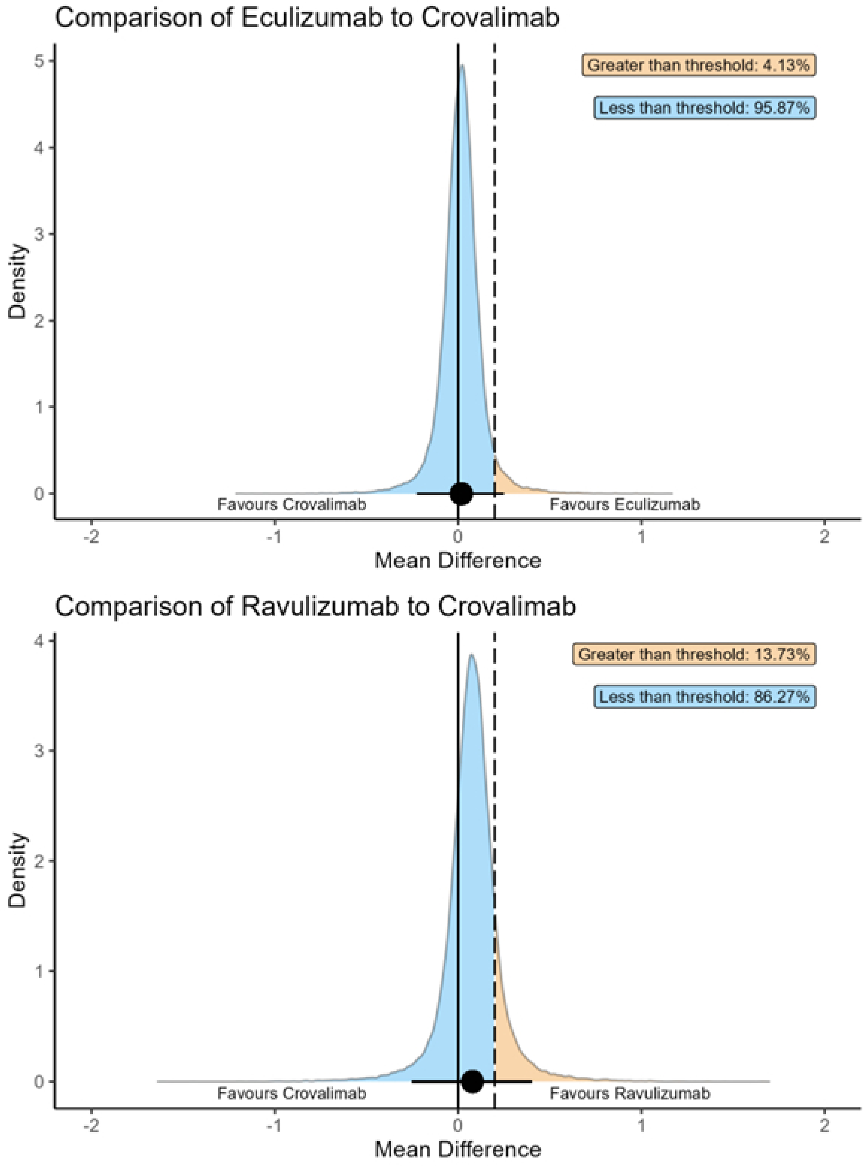

Caption: Point-and-density plots for the comparisons of eculizumab to crovalimab and ravulizumab to crovalimab for the percentage of patients achieving transfusion avoidance in the overall population.

402x536mm (38 x 38 DPI)

Online Supplementary File 1

Systematic Literature Review (SLR) methods

Inclusion and exclusion criteria

This review aimed to include National Institute for Health and Care Excellence (NICE) cost-comparison evaluations (CCEs) from 2017 onwards that followed the CCE approach from the outset, relied on results from indirect treatment comparisons (ITCs) that were not statistically significant for at least one comparator for the primary outcome(s) and had published final guidance before completion of the review. Appraisals that had direct randomised controlled trial (RCT) evidence or statistically significant ITC results for one comparator but relied on the non-significant results of an ITC for at least one other comparator were considered for inclusion providing there were clear statements from the External Assessment Group (EAG) and/or committee about where clinical similarity can be concluded for comparators relying on the latter.

Appraisals where the results from ITCs were all statistically significant were excluded given there is less uncertainty associated with these results; however, it is possible that some appraisals that were based solely on statistically significant ITC results have been included in the review, given it was sometimes difficult to decipher due to redaction of the results and discussion of statistical significance in the published NICE papers. Where it was clear that most emphasis was placed on specific outcomes for decision-making, these appraisals were excluded if the ITC results for these outcomes were statistically significant, even if there were some non-significant differences for other outcomes that were not key to decision-making. This was a pragmatic decision to try to identify appraisals where decisions were made based on the most uncertainty from ITCs, given that the presence of statistically significant differences for the key outcomes is likely to outweigh any more uncertain results for outcomes considered less important as part of the decision-making process. Only two appraisals were excluded for this reason.

In the protocol for this review, the researchers noted that appraisals that originated as a CCE, i.e. where the company's first submission was a CCE, would be prioritised for inclusion. It was also stated that appraisals that started with cost-utility models but later incorporated cost-comparison approaches would be considered for inclusion if it was clear that decision-making was solely based on the cost-comparison evidence but only if time permitted; given that 41 appraisals with a CCE approach as its first submission were

For peer review only

identified, there was insufficient resource available to consider inclusion of appraisals that adopted a CCE approach later in the process.

Definition of primary outcomes in this SLR

In line with the review protocol, reviewers only extracted information relating to outcomes that were considered to be primary outcomes for each appraisal. NICE papers did not often outline which outcomes were primary outcomes for decision-making purposes, but in the absence of this being explicitly stated, reviewers often based this decision on which were primary outcomes in the key trials of the intervention in each appraisal or which were noted in the submissions as being key for economic models of prior appraisals in the same area. While this may have led to some inconsistencies in terms of which outcomes were extracted as primary outcomes for different appraisals in the same or similar disease area, the researchers note that all general statements relating to how companies, EAGs and committees interpreted results and made conclusions on clinical similarity overall within the appraisal have been extracted, so key information relating to methods for concluding clinical similarity should not have been lost. Adverse events were not included as primary outcomes for any appraisals as in the researchers' experience there are usually very limited data on which to base conclusions about comparability of safety profiles between treatments within ITCs, given there are commonly few events and substantial uncertainty, and in many cases ITCs are not performed for safety outcomes for these reasons, with conclusions about these outcomes likely to be based more heavily on supplementary information such as feedback from clinical experts.

Data extraction

Identifying and summarising information about clinical similarity conclusions and the rationale or methods to make these conclusions was a key aim of this systematic literature review (SLR) and the following were extracted from included appraisals to allow approaches and patterns to be identified and discussed:

- Any mention of ways of interpreting ITC results such as specific minimal clinically important difference (MCID) thresholds or other thresholds and how these were identified;
- Results of the committee's preferred ITCs for relevant comparisons, including point estimates and measures of uncertainty (such as 95% confidence or credible intervals) of the effect estimates;

For peer review only

- Any committee or EAG comments on the uncertainty in the ITC results, including what they thought about the uncertainty in the ITC results and how it may have impacted their decision or conclusions;
- Narrative comments within company submissions, EAG reports or committee discussion documents describing how results of ITCs were ultimately interpreted and how clinical similarity was confirmed or refuted in the final decision-making process; clear statements from committee discussions were considered most useful, but were often not available, so often inferences from EAG reports about preferred analyses or interpretation were required.

Where multiple versions of an ITC were available across different documents (for example, an ITC performed within the company submission may have been adapted or amended by the EAG), methods and results relating to the committee's preferred analysis only were extracted where this was clear. In some cases, results of all analyses were discussed as a whole, in which case results of each analysis were extracted separately. Where the committee's preferred analysis was unclear, the EAG's preferred analysis was assumed to be the committee's preferred analysis unless there was evidence from supporting information that the committee dismissed this analysis in favour of the company's original analysis or that it considered the results of multiple analyses when making decisions.

Details related to interpretation and decision making were captured using quotation marks in extraction tables to ensure that detail was not lost during this process. Guidance documents and committee papers available on the NICE website were prioritised for review during data extraction, with public committee slides also checked where available to identify any additional information. For appraisals where the first committee meeting included a CCE that was then rejected, only NICE papers related to the first committee meeting were reviewed, as the papers relating to the second committee meeting were not likely to provide any further information on the conclusions of the CCE stage. Review of other documents such as NICE final scopes was rarely required.

For peer review only

SLR results

Figure 1. PRISMA flow diagram of records included in the SLR

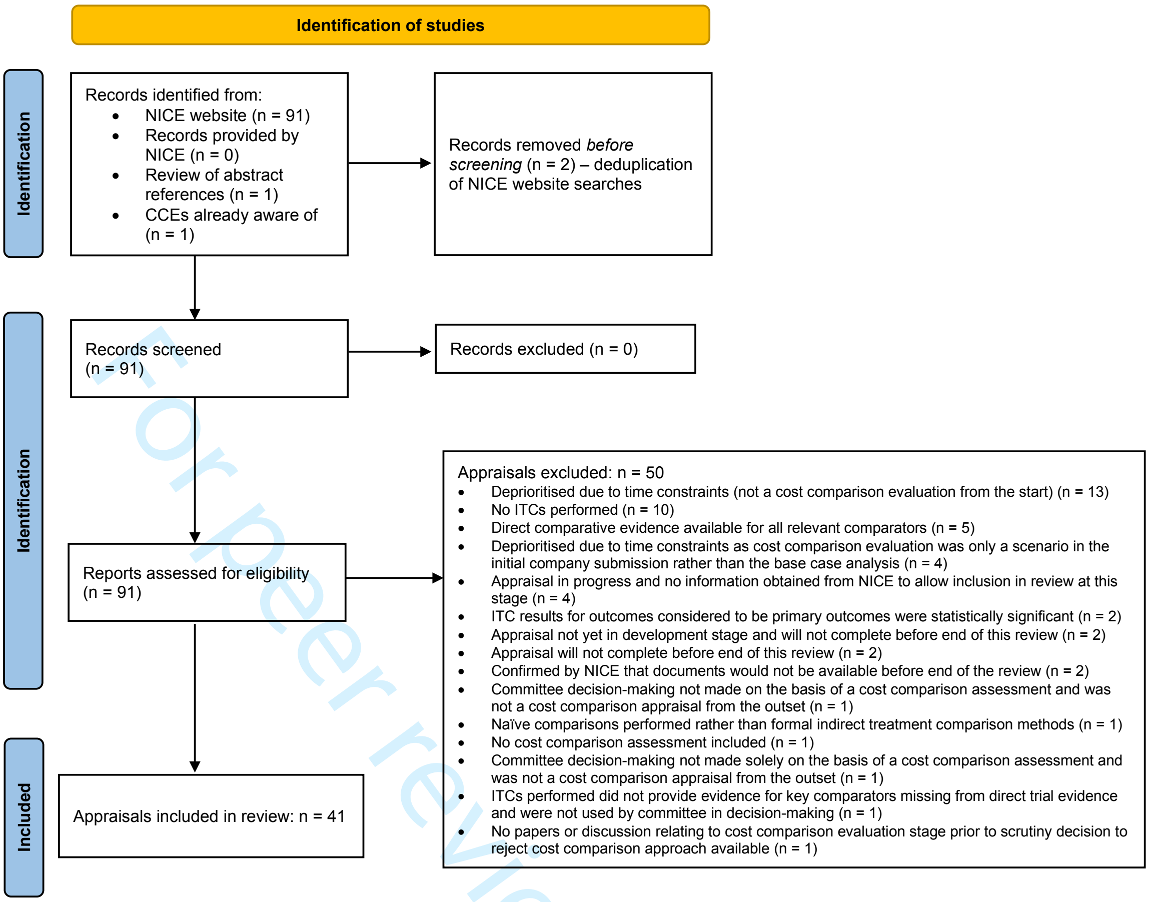

Abbreviations: CCE, cost-comparison evaluation; ITC, indirect treatment comparison; NICE, National Institute for Health and Care Excellence; PRISMA, Preferred Reporting Items for Systematic Reviews and Meta-Analyses; SLR, systematic literature review.

For peer review only

Table 1. Summary of NICE technology appraisals included in the SLR

Appraisal name (ID)	Disease area	Date published	ITC outcomes considered primary in this review	Other ITC outcomes	Comparators with only ITC evidence	Statistically significant ITC results for primary outcomes?	Comparators with direct evidence (superiority or non-inferiority)	Type of ITCs
Aflibercept for treating choroidal neovascularisation (TA486)[1]	Choroidal neovascularisation	November 2017	Gain in BCVA from baseline	None	Ranibizumab	None	None	NMAs
Golimumab for treating non-radiographic axial spondyloarthritis (TA497)[2]	Axial spondyloarthritis	January 2018	ASAS20; ASAS40; BASDAI50	CFB in BASFI; CFB in BASDAI; CFB in BASMI; AEs; serious AEs; infections	Adalimumab; etanercept; certolizumab pegol	None	None	NMAs
Guselkumab for treating moderate to severe plaque psoriasis (TA521)[3]	Plaque psoriasis	June 2018	PASI 75 response	PASI 50 response; PASI 90 response; PASI 100 response; PGA/IGA; HRQoL; AEs; serious AEs; withdrawal due to AEs	Secukinumab; ixekizumab; etanercept; infliximab<br><br>(first two considered key by committee and included here)	Statistically significant for comparison against 300 mg secukinumab 300 mg but not against 80 mg ixekizumab	Adalimumab (2 superiority trials); ustekinumab (1 superiority trial)	NMAs
Ertugliflozin as monotherapy or with metformin for treating type 2 diabetes (TA572)[4]	Type 2 diabetes	March 2019	% CFB in HbA1c	% CFB in weight; % CFB in SBP; HbA1c <7.0%; AEs; UTIs	Canagliflozin; dapagliflozin; empagliflozin	Some doses of ertugliflozin reported to be superior to specific comparators, but most non-significant	None	NMAs

For peer review only

Ertugliflozin with metformin and a dipeptidyl peptidase-4 inhibitor for treating type 2 diabetes (TA583)[5]	Type 2 diabetes	June 2019	% CFB in HbA1c	% CFB in weight; % CFB in SBP; HbA1c <7.0%; AEs; UTIs	Canagliflozin; dapagliflozin; empagliflozin	Some significant differences noted vs dapagliflozin when one trial considered but not in other analyses	None	NMAs
Risankizumab for treating moderate to severe plaque psoriasis (TA596)[6]	Plaque psoriasis	August 2019	PASI 90 response; PASI 75 response	PASI 50 response; PASI 100 response; DLQI 0/1; AEs; serious AEs; withdrawal due to AEs	Guselkumab	Likely all non-significant but unclear	Adalimumab (1 superiority trial); ustekinumab (2 superiority trials)	NMAs
Brolucizumab for treating wet age-related macular degeneration (TA672)[7]	Age-related macular degeneration	February 2021	Mean CFB in BCVA	CFB in injection frequency; CFB in CRT; gain ≥15 ETDRS letters; loss ≥15 ETDRS letters; AEs; treatment discontinuation	Ranibizumab	None	Aflibercept (2 non-inferiority trials)	NMAs
Acalabrutinib for treating chronic lymphocytic leukaemia (TA689)[8]	Chronic lymphocytic leukaemia	April 2021	OS; PFS	AEs	Ibrutinib	None	None	MAICs
Bimekizumab for treating moderate to severe plaque psoriasis (TA723)[9]	Plaque psoriasis	September 2021	PASI 90 response; PASI 75 response	PASI 50 response; PASI 100 response; AEs; serious AEs; AEs leading to discontinuation	Brodalumab; Risankizumab; ixekizumab	Possibly some statistically significant differences but unclear	Adalimumab (1 superiority trial); secukinumab (1 superiority trial); Ustekinumab (1 superiority trial)	NMAs
Secukinumab for treating moderate to severe plaque	Plaque psoriasis	October 2021	PASI 75 response	PASI 50 response; PASI 90 response;	Ustekinumab (adalimumab could not be	None	Etanercept (2 superiority trials)	NMAs

For peer review only

psoriasis in children and young people (TA734)[10]				PASI 100 response; mean CFB in CDLQI	included in the ITC)			
Tofacitinib for treating juvenile idiopathic arthritis (TA735)[11]	Juvenile idiopathic arthritis	October 2021	Disease flares; ACR Pedi responses (various thresholds)	URTI	Adalimumab; tocilizumab (etanercept could not be included in the ITC)	None	None	Bucher ITCs
Empagliflozin for treating chronic heart failure with reduced ejection fraction (TA773)[12]	Heart failure	March 2022	CV death or HHF composite	First HHF; total HHF; KCCQ; worsening renal function; CV death; all-cause mortality	Dapagliflozin	None	None	Bucher ITCs
Faricimab for treating diabetic macular oedema (TA799)[13]	Diabetic macular oedema	June 2022	Mean CFB in BCVA	Mean number of administration injections; CFB in CST; gain of ≥10/15 ETDRS letters; loss of ≥10/15 ETDRS letters; all-cause discontinuation; ocular AEs	Ranibizumab	Unclear – possibly statistically significant in overall population but not in ≥400 μm subgroup relevant to assessment	Aflibercept (4 non-inferiority trials)	NMAs
Faricimab for treating wet age-related macular degeneration (TA800)[14]	Age-related macular degeneration	June 2022	Mean CFB in BCVA	Mean number of injections; mean number of administration injections; mean CFB in CST; gain of ≥10/15 BCVA letters; ETDRS	Ranibizumab	Unclear	Aflibercept (4 non-inferiority trials)	NMAs

				letters categories; AEs; discontinuation				
Risankizumab for treating active psoriatic arthritis after inadequate response to DMARDs (TA803)[15]	Psoriatic arthritis	July 2022	ACR 20; PASI 75 response	PsARC response; ACR 50; ACR 70; PASI 50 response; PASI 90 response; PASI 100 response; CFB in HAQ-DI; AEs, serious AEs; AEs leading to discontinuation	Guselkumab	None	None	NMAs
Brolucizumab for treating diabetic macular oedema (TA820)[16]	Diabetic macular oedema	August 2022	CFB in BCVA	BCVA categorical analyses; CFB in DRSS; CFB in retinal thickness; study discontinuation; serious ocular AEs; serious non-ocular AEs	Ranibizumab	None when EAG-preferred ITCs considered (statistically significant for company's preferred analysis)	Aflibercept	NMAs
Upadacitinib for treating active ankylosing spondylitis (TA829)[17]	Ankylosing spondylitis	September 2022	ASAS 40; BASDAI 50	CFB in BASDAI; CFB in BASFI; ASAS 20; ASAS PR; CFB in total back pain score	Ixekizumab; secukinumab	None	None	NMAs
Palbociclib with fulvestrant for treating hormone receptor-positive, HER2-negative advanced breast cancer after endocrine therapy (TA836)[18]	Breast cancer	October 2022	OS; PFS	None	Abemaciclib + fulvestrant; ribociclib + fulvestrant	Unclear but likely non-significant	None	MAICs and Bucher ITCs also presented

For peer review only

Cabozantinib for previously treated advanced hepatocellular carcinoma (TA849)[19]	Hepatocellular carcinoma	December 2022	OS; PFS	Treatment-emergent AEs with a grade 3/4 occurring in ≥5% of patients in either arm	Regorafenib	None	None	Bucher ITCs and MAICs presented
Upadacitinib for treating active non-radiographic axial spondyloarthritis (TA861)[20]	Axial spondyloarthritis	February 2023	ASAS 40	BASDAI 50; CFB in BASDAI50; CFB in BASFI; ASAS 20; ASAS PR; patient assessment of CFB in total back pain	Secukinumab; ixekizumab	None	None	NMAs
Upadacitinib for previously treated moderately to severely active Crohn's disease (TA905)[21]	Crohn's disease	June 2023	CDAI clinical remission; CDAI clinical response	Serious AEs; discontinuation due to AEs	Ustekinumab; vedolizumab	Possibly statistically significant results for clinical remission outcomes but not clinical response outcomes, but unclear	None	NMAs
Bimekizumab for treating axial spondyloarthritis (TA918)[22]	Axial spondyloarthritis	October 2023	ASAS 40; BASDAI 50	ASAS 20; ASAS S5/6; ASAS PR; ASDAS <2.1; ASDAS-CII; ASDAS ID; ASDAS MI; BASDAI 50; ASDAS-CRP; ASQoL; BASDAI; BASFI; BASMI; fatigue NRS; MASES; NSP; PhGADA; SF-36 MCS; SF-36 PCS; all-cause	Secukinumab; ixekizumab	None	None	NMAs

For peer review only

				discontinuation; discontinuation due to AEs; serious AEs				
Tofacitinib for treating active ankylosing spondylitis (TA920)[23]	Ankylosing spondylitis	October 2023	ASAS 20	ASAS 40; BASDAI 50; BASDAI; BASFI; ASDAS; ASQoL; SF-36 PCS; AE-related discontinuation	Secukinumab; ixekizumab	None	None	NMAs
Mirikizumab for treating moderately to severely active ulcerative colitis (TA925)[24]	Ulcerative colitis	October 2023	Clinical response; clinical remission	Mucosal healing; all-cause discontinuation; serious AEs	Vedolizumab; ustekinumab	Possibly some statistically significant results for certain populations but most likely non-significant, unclear	None	NMAs
Empagliflozin for treating chronic heart failure with preserved or mildly reduced ejection fraction (TA929)[25]	Heart failure	November 2023	CV mortality or HHF composite	HHF; CV mortality; all-cause mortality	Dapagliflozin	None	None	Bucher ITCs
Zanubrutinib for treating chronic lymphocytic leukaemia (TA931)[26]	Chronic lymphocytic leukaemia	November 2023	OS; PFS	None	Acalabrutinib; VenR (ibrutinib was naïve comparison)	None	None	MAIC (acalabrutinib) and NMA (VenR, performed by EAG)
Empagliflozin for treating chronic kidney disease (TA942)[27]	Chronic kidney disease	December 2023	Composite renal outcome (using various thresholds)	Progression to ESKD/ESRD; HHF; CV death; HHF or CV death	Dapagliflozin	None	None	NMAs and MAICs

For peer review only

				composite; 3P-MACE+; all-cause mortality; all-cause hospitalisations				
Fluocinolone acetonide intravitreal implant for treating chronic diabetic macular oedema (TA953)[28]	Chronic diabetic macular oedema	March 2024	≥15-letter BCVA improvement	Mean CFB in BCVA letter score; mean CFB in CRT; serious ocular AEs; intraocular pressure-related AEs; cataract-related AEs	Dexamethasone intravitreal implant	None	None	Bucher ITCs and MAICs
Etrasimod for treating moderately to severely active ulcerative colitis in people aged 16 and over (TA956)[29]	Ulcerative colitis	March 2024	Clinical response; clinical remission	Serious infections during induction phase of treatment	Adalimumab, infliximab; vedolizumab (others listed but not focused on)	Statistically significant for comparison against adalimumab but not infliximab or vedolizumab	None	NMAs
Linzagolix for treating moderate to severe symptoms of uterine fibroids (TA996)[30]	Uterine fibroids	August 2024	Response (MBL ≤80 ml and ≥50% reduction from baseline)	% change in MBL; pain improvement; % change in primary fibroid volume; % change in haemoglobin; CFB in HRQoL	Relugolix CT	None	None	NMAs
Risankizumab for treating moderately to severely active ulcerative colitis (TA998)[31]	Ulcerative colitis	August 2024	Clinical response; clinical remission	Endoscopic improvement; serious infections; serious AEs	Ustekinumab	None	None	NMAs
Vibegron for treating symptoms	Overactive bladder syndrome	September 2024	Average number of daily	Total incidence of incontinence	Mirabegron	One statistically significant result	None	NMAs and Bucher ITCs

For peer review only

of overactive bladder syndrome (TA999)[32]			micturitions; number of UUI episodes	episodes; volume of urine per micturition; any AEs; serious AEs; AEs leading to study discontinuation; specific AEs (headache, hypertension, UTI, dry mouth and constipation)		for one comparison/time-point for number of UUI episodes but all others non-significant		
Faricimab for treating visual impairment caused by macular oedema after retinal vein occlusion (TA1004)[33]	Visual impairment caused by macular oedema after retinal vein occlusion	September 2024	Mean CFB in BCVA	Mean CFB in CST; categorical vision changes; serious ocular AEs; all-cause discontinuation	Ranibizumab	None	None	NMAs
Rucaparib for maintenance treatment of relapsed platinum-sensitive ovarian, fallopian tube or peritoneal cancer (TA1007)[34]	Ovarian, fallopian tube and peritoneal cancer	September 2024	PFS; OS	PFS for second event; time to start of subsequent therapy; time to treatment discontinuation	Niraparib; olaparib	Unclear, likely non-significant	None	NMAs and MAICs
Crovalimab for treating paroxysmal nocturnal haemoglobinuria in people 12 years and over (TA1019)[35]	Paroxysmal nocturnal haemoglobinuria	November 2024	Transfusion avoidance; breakthrough haemolysis	Haemoglobin stabilisation; number of packed red blood cell transfusions; FACIT fatigue score; AEs	Ravulizumab	None	Eculizumab (1 non-inferiority, 1 safety/tolerability study)	NMAs

For peer review only

Eplontersen for treating hereditary transthyretin-related amyloidosis (TA1020)[36]	Hereditary transthyretin-related amyloidosis	November 2024	Serum TTR % CFB; CFB in mNIS+7; CFB in Norfolk QoL-DN	Serum TTR absolute value and absolute CFB; response analyses for mNIS+7; responder analyses for Norfolk QoL-DN; CFB in mBMI; CFB in 10-MWT; CFB in R-ODS; serious AEs; severe AEs; treatment discontinuation	Vutrisiran	None	None	MAICs
Crizotinib for treating ROS1-positive advanced non-small-cell lung cancer (TA1021)[37]	Non-small-cell lung cancer	December 2024	OS; PFS; ORR	Duration of response	Entrectinib	Unclear but likely mostly non-significant	None	STCs and MAICs
Bevacizumab gamma for treating wet age-related macular degeneration (TA1022)[38]	Age-related macular degeneration	December 2024	Mean CFB in BCVA	Gain ≥15 BCVA letters; loss ≥15 BCVA letters; ocular AEs	Aflibercept; faricimab	None	Ranibizumab (2 superiority trials)	NMAs and MAICs
Ublituximab for treating relapsing multiple sclerosis (TA1025)[39]	Multiple sclerosis	December 2024	ARR	CDP-12; CDP-24; all-cause discontinuation	Ocrelizumab; ofatumumab	None	Teriflunomide (2 superiority trials)	NMAs
Olaparib for treating BRCA mutation-positive HER2-negative advanced breast	Breast cancer	February 2025	PFS; OS	ORR; any AEs; any serious AEs; any treatment-related serious AEs;	Talazoparib	None	None	NMAs

For peer review only

cancer after chemotherapy (TA1040)[40]				specific types of AEs				
Durvalumab with etoposide and either carboplatin or cisplatin for untreated extensive-stage small-cell lung cancer (TA1041)[41]	Small-cell lung cancer	February 2025	OS; PFS	Any AEs; specific AE types/severities	Atezolizumab	None	None	Bucher ITCs

Abbreviations: 3P-MACE, 3-point major adverse cardiovascular outcome; 10-MWT, 10-metre walk test; ACR, American College of Rheumatology; ACR Pedi, American College of Rheumatology Pediatric response criteria; AE, adverse event; ARR, annualised relapse rate; ASAS, Assessment of SpondyloArthritis international Society; ASAS-PR, Assessment of SpondyloArthritis International Society criteria partial remission; ASDAS, Ankylosing Spondylitis Disease Activity Score; ASDAS-CII, Ankylosing Spondylitis Disease Activity Score Clinically Important Improvement; ASDAS-CRP, Ankylosing Spondylitis Disease Activity Score with C-reactive protein; ASDAS-ID, Ankylosing Spondylitis Disease Activity Score Inactive Disease; ASDAS-MI, Ankylosing Spondylitis Disease Activity Score Major Improvement; ASQoL, Ankylosing Spondylitis Quality of Life; BASDAI, Bath Ankylosing Spondylitis Disease Activity Index; BASFI, Bath Ankylosing Spondylitis Functional Index; BASMI, Bath Ankylosing Spondylitis Metrology Index; BCVA, best corrected visual acuity; CFB, change from baseline; CDAI, Clinical Disease Activity Index; CDLQI, Children's Dermatology Life Quality Index; CDP, confirmed disability progression; CRT, central retinal thickness; CST, central subfield thickness; CV, cardiovascular; DLQI, Dermatology Life Quality Index; DRSS, Diabetic Retinopathy Severity Scale; EAG, External Assessment Group; ESKD/ESRD, end-stage kidney/renal disease; ETDRS, Early Treatment Diabetic Retinopathy Study; FACIT, Functional Assessment of Chronic Illness Therapy; HAQ-DI, Health Assessment Questionnaire Disability Index; HbA1c, haemoglobin A1C; HHF, hospitalisation for heart failure; HRQoL, health-related quality of life; ITC, indirect treatment comparison; KCCQ, Kansas City Cardiomyopathy Questionnaire; MAIC, matching-adjusted indirect comparison; MASES, Maastricht Ankylosing Spondylitis Enthesitis; MBL, menstrual blood loss; mBMI, modified body mass index; mNIS+7, Modified Neuropathy Impairment Score +7; NICE, National Institute for Health and Care Excellence; NMA, network-meta-analysis; NRS, numerical rating scale; Norfolk QoL-DN, Norfolk Quality of Life-Diabetic Neuropathy; NSP, nocturnal spine pain; ORR, overall response rate; OS, overall survival; PASI, Psoriasis Area and Severity Index; PCS, physical component summary; PFS, progression-free survival; PGA/IGA, Physician's Global Assessment Scale/Investigator's Global Assessment Scale; PhGADA, Physician's Global Assessment of Disease Activity; PsARC, Psoriatic Arthritis Response Criteria; R-ODS, Rasch-built Overall Disability Scale; SBP, systolic blood pressure; SF-36, Short Form 36-Item Health Survey; SLR, systematic literature review; STC, simulated treatment comparison; TTR, transthyretin; URTI, upper respiratory tract infection; UTI urinary tract infection; VenR, Venetoclax-Rituximab; UUI, urgency urinary incontinence.

Table 2. Appraisals excluded from the SLR

Appraisal (ID)	Reason for exclusion
Guidance on the use of capecitabine and tegafur with uracil for metastatic colorectal cancer (TA61)[42]	No ITCs performed
Frequency of application of topical corticosteroids for atopic eczema (TA81)[43]	No ITCs performed
Topotecan for the treatment of relapsed small-cell lung cancer (TA184)[44]	No ITCs performed
Capecitabine for the treatment of advanced gastric cancer (TA191)[45]	No ITCs performed
Dexamethasone intravitreal implant for the treatment of macular oedema secondary to retinal vein occlusion (TA229)[46]	Deprioritised due to time constraints (not a cost-comparison evaluation from the start)
Colistimethate sodium and tobramycin dry powders for inhalation for treating pseudomonas lung infection in cystic fibrosis (TA276)[47]	ITCs performed did not provide evidence for key comparators missing from direct trial evidence and were not used by committee in decision-making
Ciclosporin for treating dry eye disease that has not improved despite treatment with artificial tears (TA369)[48]	No ITCs performed
Dasatinib, nilotinib and high-dose imatinib for treating imatinib-resistant or intolerant chronic myeloid leukaemia (TA425)[49]	No ITCs performed
Dasatinib, nilotinib and imatinib for untreated chronic myeloid leukaemia (TA426)[50]	Deprioritised due to time constraints (not a cost-comparison evaluation from the start)
Everolimus for advanced renal cell carcinoma after previous treatment (TA432)[51]	No ITCs performed
Ustekinumab for moderately to severely active Crohn's disease after previous treatment (TA456)[52]	Deprioritised due to time constraints as cost-comparison evaluation was only a scenario in the initial company submission rather than the base case analysis
Atezolizumab for treating locally advanced or metastatic non-small-cell lung cancer after chemotherapy (TA520)[53]	Deprioritised due to time constraints (not a cost-comparison evaluation from the start)
Pembrolizumab for treating relapsed or refractory classical Hodgkin lymphoma (TA540)[54]	No cost-comparison assessment included
Venetoclax with rituximab for previously treated chronic lymphocytic leukaemia (TA561)[55]	Deprioritised due to time constraints (not a cost-comparison evaluation from the start)
Encorafenib with binimetinib for unresectable or metastatic BRAF V600 mutation-positive melanoma (TA562)[56]	Committee decision-making not made solely on the basis of a cost-comparison assessment and was not a cost-comparison appraisal from the outset
Abemaciclib with an aromatase inhibitor for previously untreated, hormone receptor-positive, HER2-negative, locally advanced or metastatic breast cancer (TA563)[57]	Deprioritised due to time constraints (not a cost-comparison evaluation from the start)
Benralizumab for treating severe eosinophilic asthma (TA565)[58]	Deprioritised due to time constraints (not a cost-comparison evaluation from the start)

Ustekinumab for treating moderately to severely active ulcerative colitis (TA633)[59]	Deprioritised due to time constraints (not a cost-comparison evaluation from the start)
Brigatinib for ALK-positive advanced non-small-cell lung cancer that has not been previously treated with an ALK inhibitor (TA670)[60]	Deprioritised due to time constraints as cost-comparison evaluation was only a scenario in the initial company submission rather than the base case analysis
Mepolizumab for treating severe eosinophilic asthma (TA671)[61]	ITC results for outcomes considered to be primary outcomes were statistically significant
Anakinra for treating Still's disease (TA685)[62]	Deprioritised due to time constraints (not a cost-comparison evaluation from the start)
Selective internal radiation therapies for treating hepatocellular carcinoma (TA688)[63]	No ITCs performed
Atezolizumab monotherapy for untreated advanced non-small-cell lung cancer (TA705)[64]	Deprioritised due to time constraints as cost-comparison evaluation was only a scenario in the initial company submission rather than the base case analysis
Diroximel fumarate for treating relapsing–remitting multiple sclerosis (TA794)[65]	Direct comparative evidence available for all relevant comparators
Somatrogon for treating growth disturbance in children and young people aged 3 years and over (TA863)[66]	Direct comparative evidence available for all relevant comparators
Vutrisiran for treating hereditary transthyretin-related amyloidosis (TA868)[67]	Direct comparative evidence available for all relevant comparators
Eptinezumab for preventing migraine (TA871)[68]	Deprioritised due to time constraints as cost-comparison evaluation was only a scenario in the initial company submission rather than the base case analysis
Risankizumab for previously treated moderately to severely active Crohn's disease (TA888)[69]	Deprioritised due to time constraints (not a cost-comparison evaluation from the start)
Pegunigalsidase alfa for treating Fabry disease (TA915)[70]	No ITCs performed
Bimekizumab for treating active psoriatic arthritis (TA916)[71]	ITC results for outcomes considered to be primary outcomes were statistically significant
Momelotinib for treating myelofibrosis-related splenomegaly or symptoms (TA957)[72]	Direct comparative evidence available for all relevant comparators
Atogepant for preventing migraine (TA973)[73]	No papers or discussion relating to CCE stage prior to scrutiny decision to reject CCE available
Selective internal radiation therapy with QuiremSpheres for treating unresectable advanced hepatocellular carcinoma (TA985)[74]	Naïve comparisons performed rather than formal indirect treatment comparison methods
Tenecteplase for treating acute ischaemic stroke (TA990)[75]	Direct comparative evidence available for all relevant comparators
Trastuzumab deruxtecan for treating HER2-low metastatic or unresectable breast cancer after chemotherapy (TA992)[76]	No ITCs performed
Pembrolizumab with platinum- and fluoropyrimidine-based chemotherapy for untreated advanced HER2-negative gastric or gastro-oesophageal junction adenocarcinoma (TA997)[77]	Deprioritised due to time constraints (not a cost-comparison evaluation from the start)

Evinacumab for treating homozygous familial hypercholesterolaemia in people 12 years and over (TA1002)[78]	Deprioritised due to time constraints (not a cost-comparison evaluation from the start)
Futibatinib for previously treated advanced cholangiocarcinoma with FGFR2 fusion or rearrangement (TA1005)[79]	Committee decision-making not made on the basis of a cost-comparison assessment and was not a cost-comparison appraisal from the outset
Latanoprost–netarsudil for previously treated primary open-angle glaucoma or ocular hypertension (TA1009)[80]	Deprioritised due to time constraints (not a cost-comparison evaluation from the start)
Fenfluramine for treating seizures associated with Lennox–Gastaut syndrome in people 2 years and over (TA1050)[81]	Deprioritised due to time constraints (not a cost-comparison evaluation from the start)
Tislelizumab for treating unresectable advanced oesophageal squamous cell cancer after platinum-based chemotherapy (ID4070)[82]	Appraisal in progress and no information obtained from NICE to allow inclusion in review at this stage
Somapacitan for treating growth hormone deficiency in children (ID6178)[83]	Appraisal in progress and no information obtained from NICE to allow inclusion in review at this stage
Guselkumab for treating moderately to severely active ulcerative colitis (ID6237)[84]	Appraisal will not complete before end of this review
Guselkumab for previously treated moderately to severely active Crohn's disease (ID6238)[85]	Appraisal in progress and no information obtained from NICE to allow inclusion in review at this stage
Mirikizumab for treating moderately to severely active Crohn's disease (ID6244)[86]	Appraisal in progress and no information obtained from NICE to allow inclusion in review at this stage
Nivolumab as neoadjuvant (with chemotherapy) and adjuvant (as monotherapy) treatment for resectable non-small-cell lung cancer (ID6310)[87]	Confirmed by NICE that documents would not be available before end of the review
Vanzacaftor–tezacaftor–deutivacaftor for treating cystic fibrosis with 1 or more F508del mutations in the CFTR gene in people aged 6 years and over (ID6372)[88]	Appraisal will not complete before end of this review
Abiraterone (originator and generics) for treating newly diagnosed high-risk hormone-sensitive metastatic prostate cancer (review of TA721) (ID6378)[89]	Appraisal not yet in development stage and will not complete before end of this review
Rilzabrutinib for treating persistent or chronic immune thrombocytopenia in people aged 12 and over (ID6395)[90]	Appraisal not yet in development stage and will not complete before end of this review
Darolutamide with androgen deprivation therapy for treating hormone-sensitive metastatic prostate cancer (ID6452)[91]	Confirmed by NICE that documents would not be available before end of the review
Abbreviations: CCE, cost-comparison evaluation; ITC, indirect treatment comparison; NICE, National Institute for Health and Care Excellence; SLR, systematic literature review.	

Table 3. Summary of methods used by companies to demonstrate clinical similarity

Method	Details	Appraisals
**Interpreting ITC results**		
Lack of statistically significant differences only	Relies solely on a lack of statistically significant differences only to interpret ITC results and conclude similarity.	**Three:** TA735, TA829 and TA1040.[11, 17, 40]
Lack of statistically significant differences and some discussion of point estimate direction and/or size	Relies mostly on a lack of statistically significant differences and direction and/or size of point estimates to interpret ITC results and conclude similarity.<br><br>Point estimates favouring the intervention over comparators are often noted, with those discussing point estimate size commenting on the difference being small and close to 0.0 or 1.0 (line of null effect).<br><br>Some refer to the fact that point estimates vary in direction for different outcomes, suggesting no consistent trend for a benefit of one treatment or the other as evidence of no difference.	**Fourteen:** TA689, TA773, TA849, TA861, TA929, TA931, TA942, TA953, TA998, TA1007, TA1020, TA1021, TA1025, TA1041[8, 19, 20, 25-28, 31, 34, 36, 37, 39, 41, 92]
Some statistically significant differences but none favouring the comparators	Some analyses with statistically significant differences are noted but none of these favour the comparator(s).	**Eight:** TA521, TA572, TA583, TA672, TA799, TA836, TA918, TA925[3-5, 7, 13, 18, 22, 24]
Size and direction of point estimates mentioned with no mention of lack of statistically significant differences	Size and direction of point estimates used to make conclusions of clinical similarity, with no emphasis placed on the lack of statistically significant differences.	**One:** TA803[15]
Some use of probabilities calculated from NMAs to interpret results	NMAs used to calculate probabilities of being best treatment, among best treatments, clinically equivalent or non-inferior, for example based on SUCRA values or incorporating equivalence or non-inferiority margins.<br><br>Often used alongside other methods of interpreting results such as highlighting a lack of statistically significant differences, point estimates close to 1.0 or 0.0 or point estimates favouring the intervention over the comparator(s).<br><br>In some cases they are mentioned but not focused on or are dismissed if not favouring the intervention.	**Six:** TA734, TA803, TA820, TA996, TA1019, TA1022[10, 15, 16, 30, 35, 38]

For peer review only

MCIDs or other thresholds used to support with interpretation of results	MCIDs or other thresholds used by the company in some way to interpret ITC results and conclude clinical similarity.<br><br>Often used alongside other methods of interpreting results such as highlighting a lack of statistically significant differences, point estimates close to 1.0 or 0.0 or point estimates favouring the intervention over the comparator(s).	**Four:** TA486, TA803, TA999, TA1019[1, 15, 32, 35]
Unclear what focus of decision-making was	Rationale for concluding similarity was unclear either because results and discussion sections were heavily redacted or because the rationale for conclusions of clinical similarity were not described.	**Ten:** TA497, TA596, TA723, TA734, TA800, TA820, TA905, TA920, TA956, TA1004[2, 6, 9, 10, 14, 16, 21, 23, 29, 33]
**Supplementary evidence**		
Similar mechanisms of action or pharmacodynamic profiles noted	Similarity in terms of mechanisms of action or pharmacodynamic profiles noted by the company as supportive evidence that the intervention is expected to be similar to the comparator(s).	**Six:** TA486, TA572, TA689, TA773, TA953, TA998[1, 4, 8, 28, 31, 92]
Clinical expert feedback	Clinical expert feedback stating that the treatments are expected to or have similar efficacy in their experience or clinical expert interpretation of ITC results as likely similar.	**Ten:** TA689, TA773, TA803, TA849, TA861, TA931, TA942, TA996, TA1040, TA1041[8, 15, 19, 20, 26, 27, 30, 40, 41, 92]
Reference to meta-analyses of intervention and comparator trials vs a common comparator as evidence of a class effect	Meta-analyses of comparator and intervention trials against a common comparator cited as evidence that there is a class effect, suggesting clinical similarity should be concluded.	**Three:** TA773, TA836, TA942[18, 27, 92]
Reference to previously published ITC results or NICE appraisals	Reference to other ITCs of the intervention and comparator(s) relevant to the appraisal within the literature or previous NICE appraisals as supportive evidence of clinical similarity given similar results were obtained.	**Four:** TA803, TA836, TA849, TA861[15, 18-20]
Reference to real-world data as supportive evidence	Reference to real-world data used as supportive evidence to ITCs evidence	**One:** TA1007[34]
Abbreviations: ITC, indirect treatment comparison; MCID, minimal clinically important difference; NICE, National Institute for Health and Care Excellence; NMA, network meta-analyses; SUCRA, Surface Under the Cumulative RAnking curve.		

For peer review only

Table 4. Summary of methods used or comments made by EAGs in assessing clinical similarity

Method/comment	Details	Number of appraisals
**Interpretation of lack of statistically significant differences**		
Lack of statistically significant differences with some point estimates favouring the intervention used to make conclusions	Discussion of results and conclusions of clinical similarity focuses on the lack of statistically significant differences as well as at least some point estimates that favour the new intervention.	**Three:** TA829, TA953, TA1004[17, 28, 33]
Lack of statistically significant differences not completely dismissed but other methods of interpreting ITC results considered in making conclusions	Does not reject the use of lack of statistically significant differences as a method of making conclusions completely but incorporates other evidence in the decision-making, such as visual comparisons of adjusted KM curves, clinical expert feedback, similar mechanisms of action, decisions made in previous appraisals or additional data or analyses requested from the company or performed by the EAG itself.<br><br>While lack of statistically significant differences not rejected outright, EAGs sometimes highlight that it should be interpreted as no evidence of a difference between treatments rather than clinical similarity or equivalence conclusively.	**Twelve:** TA486, TA497, TA583, TA596, TA689, TA735, TA918, TA929, TA942, TA1007, TA1019, TA1041[1, 2, 5, 6, 8, 11, 22, 25, 27, 34, 35, 41]
Dismisses a lack of statistically significant differences as evidence for clinical similarity or equivalence	EAGs explicitly dismissed the idea that a lack of statistically significant differences is sufficient to conclude clinical similarity or equivalence, or it was otherwise suggested based on uncertainties discussed or requests made to the company.<br>In many cases this contributed to conclusions made by the EAG that there was insufficient evidence to support a CCE, but in some cases these uncertainties were addressed by consideration of other evidence and clinical similarity was concluded overall. For some appraisals, rejection of a CCE was likely not solely based on the lack of statistically significant differences and other factors such as concerns about trials included in the ITC or methodology may have contributed to this conclusion.	**Nineteen:** TA521, TA773, TA803, TA820, TA836, TA849, TA861, TA905, TA920, TA925, TA931, TA956, TA996, TA998, TA999, TA1020, TA1021, TA1025, TA1040[3, 15, 16, 18-21, 23, 24, 26, 29-32, 36, 37, 39, 40, 92]
Main rationale for conclusions of clinical similarity and position on lack of statistically significant differences unclear	EAG concludes that clinical similarity is likely to be a reasonable conclusion but the main factors for decision-making are unclear and there is no clear statement on the EAG's position regarding lack of statistically significant differences.	**Five:** TA572, TA672, TA723, TA800, TA1022[4, 7, 9, 14, 38]
**Formal methods of interpreting ITC results**		

Some use of probabilities calculated from NMAs to interpret results	NMAs used to calculate probabilities of being the best treatment, among the best treatments, clinically equivalent or non-inferior; for example, based on SUCRA values or incorporating equivalence or non-inferiority margins.<br><br>Reference to these data provided by companies was made to aid with the EAG's interpretation of results, there was a suggestion that these would have been useful if not provided or there was no criticism of the company's use of these data	**Nine:** TA497, TA734, TA803, TA820, TA996, TA1019, TA1022, TA1025, TA1040[2, 10, 15, 16, 30, 35, 38-40]
MCIDs or other thresholds used to support with interpretation of results	MCIDs or other thresholds used by the EAG in some way to interpret ITC results and conclude clinical similarity or mentioned as being potentially useful.<br><br>Based on the EAG making use of these put forward by the company or adapted versions, the EAG putting forward this approach itself or no criticism of the method having been used by the company	**Nine:** TA486, TA497, TA803, TA931, TA996, TA999, TA1019, TA1020, TA1040 [1, 2, 15, 26, 30, 32, 35, 36, 40]
**Supplementary evidence**		
Similar mechanisms of action or pharmacodynamic profiles noted	Similarity in terms of mechanisms of action or pharmacodynamic profiles noted by the EAG as supportive evidence that the intervention is expected to be similar to the comparator(s).	**Fourteen:** TA486, TA497, TA572, TA596, TA836, TA918, TA929, TA996, TA998, TA999, TA1007, TA1022, TA1025, TA1040[1, 2, 4, 6, 18, 22, 25, 30-32, 34, 38-40]
Clinical expert feedback	Clinical expert feedback stating that the treatments are expected to or have similar efficacy in their experience or clinical expert input to support with interpretation of ITC results, such as identification of MCIDs	**Fourteen:** TA497, TA689, TA723, TA836, TA929, TA931, TA956, TA998, TA999, TA1007, TA1020, TA1022, TA1025, TA1040[2, 8, 9, 18, 26, 29, 31, 32, 34, 36, 38-40, 93]
Other sources noted	Other sources of evidence used as supportive evidence of clinical similarity, including results of ITCs from previous NICE appraisals, statistical significance testing requested from companies, results from amended analyses to better align trials, visual review of adjusted KM curves from ITCs and naïve comparison of data.<br><br>Typically rejected meta-analyses of interventions and comparators to demonstrate class effect as supportive of clinical similarity when put forward by the company.	**Fourteen:** TA486, TA497, TA572, TA583, TA723, TA735, TA773, TA836, TA918, TA929, TA942, TA1007, TA1021, TA1041[1, 2, 4, 5, 9, 11, 18, 22, 25, 27, 34, 37, 41, 92]

For peer review only

Abbreviations: EAG, External Assessment Group; CCE, cost-comparison evaluation; ITC, indirect treatment comparison; KM, Kaplan-Meier; MCID, minimal clinically important difference; NICE, National Institute for Health and Care Excellence; NMA, network meta-analyses; SUCRA, Surface Under the Cumulative RAnking curve.		

Table 5. Summary of comments made by committees when making conclusions of clinical similarity.

Comment	Details	Number of appraisals
Discussion very limited, simply makes conclusions of clinical similarity	Only brief statement that results from ITCs were considered to indicate that intervention is likely to be similar to comparator(s), with no rationale or comments on uncertainty mentioned.	**Twenty-four:** TA572, TA583, TA596, TA672, TA723, TA734, TA735, TA800, TA836, TA905, TA918, TA925, TA942, TA953, TA998, TA999, TA1007, TA1019, TA1020, TA1021, TA1022, TA1025, TA1040, TA1041[4-7, 9-11, 14, 18, 21, 22, 24, 27, 28, 31, 32, 34-41]
Concludes similarity with uncertainty mentioned but no rationale as to how this was resolved	Notes uncertainties in ITC results but concludes clinical similarity likely overall with no discussion of how this uncertainty was addressed, why it was not considered a major issue or mention of other information that may have reduced uncertainty.	**Four:** TA521, TA799, TA920, TA929[3, 13, 23, 25]
Notes uncertainty in ITC results with clinical expert feedback considered before concluding clinical similarity	Uncertainty in ITC results noted but clinical expert feedback appears to have reduced concerns about this uncertainty to allow a conclusion of clinical similarity to be made.	**Seven:** TA486, TA689, TA820, TA829, TA849, TA861, TA905[1, 8, 16, 17, 19-21]
Notes uncertainty in ITC results with similarities in mechanisms of action considered before concluding clinical similarity	Uncertainty in ITC results noted but similarities in mechanisms of action may have reduced concerns about this uncertainty to allow a conclusion of clinical similarity to be made.	**Three:** TA773, TA803, TA956[9, 15, 29]
Notes uncertainty in ITC results but robustness of results to alternative analyses considered before concluding clinical similarity	Uncertainty in ITC results noted but the fact that results were robust to alternative analyses seems to have reduced some of the concern to allow a conclusion of clinical similarity to be made.	**One:** TA497[2]

For peer review only

Notes uncertainty in ITC results but point estimates being close to 1.0 or 0.0 considered in decision-making	Uncertainty in ITC results noted but the fact that point estimates were close to 1.0 or 0.0 seems to have reduced some of the concern to allow a conclusion of clinical similarity to be made.	**One:** TA803[15]
Committee concluded that results are too uncertain and cost-utility analyses required, or cost-utility analyses used despite conclusions of similarity from ITC results	Conclusion that there was too much uncertainty in the ITC results and cost-utility analyses were required for decision-making, or a conclusion of clinical similarity was made based on ITC results, but cost-utility analyses still appear to have been used for decision-making.	**Three:** TA849, TA931, TA996[19, 26, 30]
Cost-utility results available alongside cost-comparison analyses which may have contributed to decision-making	Conclusion of clinical similarity made but some cost-utility analyses were available alongside cost-comparison analyses, which may have reduced uncertainty in decision-making.	**One:** TA773[92]
Abbreviations: ITC, indirect treatment comparison.		

For peer review only

Table 6. Additional findings from the SLR

Finding	Details
Additional detail regarding the committee's interpretation of statistically non-significant ITC results and consideration of supportive evidence may improve the transparency of decision-making.	In some appraisals, EAGs rejected the CCE given the uncertainty in the conclusions of clinical similarity. In some cases, the committee subsequently determined that there was sufficient evidence of clinical similarity.<br>In these cases, it was clear from most committee discussions that supportive evidence, such as clinical expert feedback, may have supported this decision, but there was rarely an explicit discussion of why these were considered to sufficiently alleviate the uncertainties raised by the EAG.
Guidance on whether or not a CCE within the NICE process needs to present a case against multiple or only one relevant comparator would be helpful, as there is clearly some uncertainty about this	EAG and committee discussions in some appraisals in this SLR suggest that demonstrating clinical similarity compared to one relevant comparator may be sufficient, including statements made by the committee in NICE TA1022 and by the EAG in NICE TA1007.[34, 38] In CCEs with more than one comparator, EAGs and committees have generally discussed the evidence for clinical similarity for all comparators separately rather than focusing on one with the strongest evidence.<br>A clear statement supporting the requirement for a case against only one comparator was not identified in the current NICE methods manual,[94] and an article by Costello Medical has recently expressed uncertainty with regards to this.[95] Therefore, clarity surrounding this requirement within NICE CCEs in the form of updated guidance may be useful for companies and EAGs.
Abbreviations:	

For peer review only

References

1. National Institute for Health and Care Excellence (NICE). Aflibercept for treating choroidal neovascularisation - Technology appraisal guidance [TA486], 2017. Available from: https://www.nice.org.uk/guidance/ta486. Date accessed: May 25.

2. National Institute for Health and Care Excellence (NICE). Golimumab for treating non-radiographic axial spondyloarthritis - Technology appraisal guidance [TA497] 2018. Available from: https://www.nice.org.uk/guidance/ta497. Date accessed: May 25.

3. National Institute for Health and Care Excellence (NICE). Guselkumab for treating moderate to severe plaque psoriasis - Technology appraisal guidance [TA521], 2018. Available from: https://www.nice.org.uk/guidance/ta521. Date accessed: Apr 25.

4. National Institute for Health and Care Excellence (NICE). Ertugliflozin as monotherapy or with metformin for treating type 2 diabetes - Technology appraisal guidance [TA572], 2019. Available from: https://www.nice.org.uk/guidance/ta572. Date accessed: May 25.

5. National Institute for Health and Care Excellence (NICE). Ertugliflozin with metformin and a dipeptidyl peptidase-4 inhibitor for treating type 2 diabetes - Technology appraisal guidance [TA583], 2019. Available from: https://www.nice.org.uk/guidance/ta583. Date accessed: May 25.

6. National Institute for Health and Care Excellence (NICE). Risankizumab for treating moderate to severe plaque psoriasis - Technology appraisal guidance [TA596], 2019. Available from: https://www.nice.org.uk/guidance/ta596. Date accessed: Apr 25.

7. National Institute for Health and Care Excellence (NICE). Brolucizumab for treating wet age-related macular degeneration - Technology appraisal guidance [TA672], 2021. Available from: https://www.nice.org.uk/guidance/ta672. Date accessed: Apr 25.

8. National Institute for Health and Care Excellence (NICE). Acalabrutinib for treating chronic lymphocytic leukaemia - Technology appraisal guidance [TA689], 2021. Available from: https://www.nice.org.uk/guidance/ta689. Date accessed: Apr 25.

9. National Institute for Health and Care Excellence (NICE). Bimekizumab for treating moderate to severe plaque psoriasis - Technology appraisal guidance [TA723], 2021. Available from: https://www.nice.org.uk/guidance/ta723. Date accessed: Apr 25.

10. National Institute for Health and Care Excellence (NICE). Secukinumab for treating moderate to severe plaque psoriasis in children and young people - Technology appraisal guidance [TA734], 2021. Available from: https://www.nice.org.uk/guidance/ta734. Date accessed: Apr 25.

11. National Institute for Health and Care Excellence (NICE). Tofacitinib for treating juvenile idiopathic arthritis - Technology appraisal guidance [TA735], 2021. Available from: https://www.nice.org.uk/guidance/ta735. Date accessed: Apr 25.

12. National Institute for Health and Care Excellence (NICE). Empagliflozin for treating chronic heart failure with reduced ejection fraction - Technology appraisal guidance [TA773] - Committee papers, 2022. Available from: https://www.nice.org.uk/guidance/ta773/history. Date accessed: May 25.

For peer review only

13. National Institute for Health and Care Excellence (NICE). Faricimab for treating diabetic macular oedema - Technology appraisal guidance [TA799], 2022. Available from: https://www.nice.org.uk/guidance/ta799. Date accessed: Apr 25.

14. National Institute for Health and Care Excellence (NICE). Faricimab for treating wet age-related macular degeneration - Technology appraisal guidance [TA800], 2022. Available from: https://www.nice.org.uk/guidance/ta800. Date accessed: Apr 25.

15. National Institute for Health and Care Excellence (NICE). Risankizumab for treating active psoriatic arthritis after inadequate response to DMARDs - Technology appraisal guidance [TA803], 2022. Available from: https://www.nice.org.uk/guidance/ta803. Date accessed: Apr 25.

16. National Institute for Health and Care Excellence (NICE). Brolucizumab for treating diabetic macular oedema - Technology appraisal guidance [TA820], 2022. Available from: https://www.nice.org.uk/guidance/ta820. Date accessed: Apr 25.

17. National Institute for Health and Care Excellence (NICE). Upadacitinib for treating active ankylosing spondylitis - Technology appraisal guidance [TA829], 2022. Available from: https://www.nice.org.uk/guidance/ta829. Date accessed: Apr 25.

18. National Institute for Health and Care Excellence (NICE). Palbociclib with fulvestrant for treating hormone receptor-positive, HER2-negative advanced breast cancer after endocrine therapy - Technology appraisal guidance [TA836], 2022. Available from: https://www.nice.org.uk/guidance/ta836. Date accessed: Apr 25.

19. National Institute for Health and Care Excellence (NICE). Cabozantinib for previously treated advanced hepatocellular carcinoma - Technology appraisal giudance [TA849], 2022. Available from: https://www.nice.org.uk/guidance/ta849. Date accessed: May 25.

20. National Institute for Health and Care Excellence (NICE). Upadacitinib for treating active non-radiographic axial spondyloarthritis - Technology appraisal guidance [TA861], 2023. Available from: https://www.nice.org.uk/guidance/ta861. Date accessed: Apr 25.

21. National Institute for Health and Care Excellence (NICE). Upadacitinib for previously treated moderately to severely active Crohn's disease - Technology appraisal guidance [TA905], 2023. Available from: https://www.nice.org.uk/guidance/ta905. Date accessed: Apr 25.

22. National Institute for Health and Care Excellence (NICE). Bimekizumab for treating axial spondyloarthritis - Technology appraisal guidance [TA918], 2023. Available from: https://www.nice.org.uk/guidance/ta918. Date accessed: Apr 25.

23. National Institute for Health and Care Excellence (NICE). Tofacitinib for treating active ankylosing spondylitis - Technology appraisal guidance [TA920], 2023. Available from: https://www.nice.org.uk/guidance/ta920. Date accessed: Apr 25.

24. National Institute for Health and Care Excellence (NICE). Mirikizumab for treating moderately to severely active ulcerative colitis - Technology appraisal guidance [TA925], 2023. Available from: https://www.nice.org.uk/guidance/ta925. Date accessed: Apr 25.

25. National Institute for Health and Care Excellence (NICE). Empagliflozin for treating chronic heart failure with preserved or mildly reduced ejection fraction - Technology appraisal guidance [TA929], 2023. Available from: https://www.nice.org.uk/guidance/ta929. Date accessed: Apr 25.

For peer review only

26. National Institute for Health and Care Excellence (NICE). Zanubrutinib for treating chronic lymphocytic leukaemia - Technology appraisal guidance [TA931], 2023. Available from: https://www.nice.org.uk/guidance/ta931. Date accessed: May 25.

27. National Institute for Health and Care Excellence (NICE). Empagliflozin for treating chronic kidney disease - Technology appraisal guidance [TA942], 2023. Available from: https://www.nice.org.uk/guidance/ta942. Date accessed: Apr 25.

28. National Institute for Health and Care Excellence (NICE). Fluocinolone acetonide intravitreal implant for treating chronic diabetic macular oedema - Technology appraisal guidance [TA953], 2024. Available from: https://www.nice.org.uk/guidance/ta953. Date accessed: Apr 25.

29. National Institute for Health and Care Excellence (NICE). Etrasimod for treating moderately to severely active ulcerative colitis in people aged 16 and over - Technology appraisal guidance [TA956], 2024. Available from: https://www.nice.org.uk/guidance/ta956. Date accessed: Apr 25.

30. National Institute for Health and Care Excellence (NICE). Linzagolix for treating moderate to severe symptoms of uterine fibroids - Technology appraisal guidance [TA996], 2024. Available from: https://www.nice.org.uk/guidance/ta996. Date accessed: Apr 25.

31. National Institute for Health and Care Excellence (NICE). Risankizumab for treating moderately to severely active ulcerative colitis - Technology appraisal guidance [TA998], 2024. Available from: https://www.nice.org.uk/guidance/ta998. Date accessed: Apr 25.

32. National Institute for Health and Care Excellence (NICE). Vibegron for treating symptoms of overactive bladder syndrome - Technology appraisal guidance [TA999], 2024. Available from: https://www.nice.org.uk/guidance/ta999. Date accessed: Apr 25.

33. National Institute for Health and Care Excellence (NICE). Faricimab for treating visual impairment caused by macular oedema after retinal vein occlusion - Technology appraisal guidance [TA1004], 2024. Available from: https://www.nice.org.uk/guidance/ta1004. Date accessed: Apr 25.

34. National Institute for Health and Care Excellence (NICE). Rucaparib for maintenance treatment of relapsed platinum-sensitive ovarian, fallopian tube or peritoneal cancer - Technology appraisal guidance [TA1007], 2024. Available from: https://www.nice.org.uk/guidance/ta1007. Date accessed: Apr 25.

35. National Institute for Health and Care Excellence (NICE). Crovalimab for treating paroxysmal nocturnal haemoglobinuria in people 12 years and over - Technology appraisal guidance [TA1019], 2024. Available from: https://www.nice.org.uk/guidance/ta1019. Date accessed: Apr 25.

36. National Institute for Health and Care Excellence (NICE). Eplontersen for treating hereditary transthyretin-related amyloidosis - Technology appraisal guidance [TA1020], 2024. Available from: https://www.nice.org.uk/guidance/ta1020. Date accessed: Apr 25.

37. National Institute for Health and Care Excellence (NICE). Crizotinib for treating ROS1-positive advanced non-small-cell lung cancer - Technology appraisal guidance [TA1021], 2024. Available from: https://www.nice.org.uk/guidance/ta1021. Date accessed: Apr 25.

For peer review only

38. National Institute for Health and Care Excellence (NICE). Bevacizumab gamma for treating wet age-related macular degeneration - Technology appraisal guidance [TA1022], 2024. Available from: https://www.nice.org.uk/guidance/ta1022. Date accessed: Apr 25.

39. National Institute for Health and Care Excellence (NICE). Ublituximab for treating relapsing multiple sclerosis - Technology appraisal guidance [TA1025], 2024. Available from: https://www.nice.org.uk/guidance/ta1025. Date accessed: Apr 25.

40. National Institute for Health and Care Excellence (NICE). Olaparib for treating BRCA mutation-positive HER2-negative advanced breast cancer after chemotherapy - Technology appraisal guidance [TA1040], 2025. Available from: https://www.nice.org.uk/guidance/ta1040. Date accessed: Apr 25.

41. National Institute for Health and Care Excellence (NICE). Durvalumab with etoposide and either carboplatin or cisplatin for untreated extensive-stage small-cell lung cancer - Technology appraisal guidance [TA1041], 2025. Available from: https://www.nice.org.uk/guidance/ta1041. Date accessed: Apr 25.

42. National Institute for Health and Care Excellence (NICE). Guidance on the use of capecitabine and tegafur with uracil for metastatic colorectal cancer - Technology appraisal guidance [TA61], 2003. Available from: https://www.nice.org.uk/guidance/ta61. Date accessed: May 25.

43. National Institute for Health and Care Excellence (NICE). Frequency of application of topical corticosteroids for atopic eczema - Technology appraisal guidance [TA81], 2004. Available from: https://www.nice.org.uk/guidance/ta81. Date accessed: May 25.

44. National Institute for Health and Care Excellence (NICE). Topotecan for the treatment of relapsed small-cell lung cancer - Technology appraisal guidance [TA184], 2009. Available from: https://www.nice.org.uk/guidance/ta184. Date accessed: May 25.

45. National Institute for Health and Care Excellence (NICE). Capecitabine for the treatment of advanced gastric cancer - Technology appraisal guidance [TA191], 2010. Available from: https://www.nice.org.uk/guidance/ta191. Date accessed: May 25.

46. National Institute for Health and Care Excellence (NICE). Dexamethasone intravitreal implant for the treatment of macular oedema secondary to retinal vein occlusion - Technology appraisal guidance [TA229], 2011. Available from: https://www.nice.org.uk/guidance/ta229. Date accessed: May 25.

47. National Institute for Health and Care Excellence (NICE). Colistimethate sodium and tobramycin dry powders for inhalation for treating pseudomonas lung infection in cystic fibrosis - Technology appraisal guidance [TA276], 2013. Available from: https://www.nice.org.uk/guidance/ta276. Date accessed: May 25.

48. National Institute for Health and Care Excellence (NICE). Ciclosporin for treating dry eye disease that has not improved despite treatment with artificial tears - Technology appraisal guidance [TA369], 2015. Available from: https://www.nice.org.uk/guidance/ta369. Date accessed: May 25.

49. National Institute for Health and Care Excellence (NICE). Dasatinib, nilotinib and high-dose imatinib for treating imatinib-resistant or intolerant chronic myeloid leukaemia - Technology appraisal guidance [TA425], 2016. Available from: https://www.nice.org.uk/guidance/ta425. Date accessed: May 25.

For peer review only

50. National Institute for Health and Care Excellence (NICE). Dasatinib, nilotinib and imatinib for untreated chronic myeloid leukaemia - Technology appraisal guidance [TA426], 2016. Available from: https://www.nice.org.uk/guidance/ta426. Date accessed: May 25.

51. National Institute for Health and Care Excellence (NICE). Everolimus for advanced renal cell carcinoma after previous treatment - Technology appraisal guidance [TA432], 2017. Available from: https://www.nice.org.uk/guidance/ta432. Date accessed: May 25.

52. National Institute for Health and Care Excellence (NICE). Ustekinumab for moderately to severely active Crohn's disease after previous treatment - Technology appraisal guidance [TA456], 2017. Available from: https://www.nice.org.uk/guidance/ta456. Date accessed: May 25.

53. National Institute for Health and Care Excellence (NICE). Atezolizumab for treating locally advanced or metastatic non-small-cell lung cancer after chemotherapy - Technology appraisal guidance [TA520], 2018. Available from: https://www.nice.org.uk/guidance/ta520. Date accessed: May 25.

54. National Institute for Health and Care Excellence (NICE). Pembrolizumab for treating relapsed or refractory classical Hodgkin lymphoma - Technology appraisal guidance [TA540], 2018. Available from: https://www.nice.org.uk/guidance/ta540. Date accessed: May 25.

55. National Institute for Health and Care Excellence (NICE). Venetoclax with rituximab for previously treated chronic lymphocytic leukaemia - Technology appraisal guidance [TA565], 2019. Available from: https://www.nice.org.uk/guidance/ta561. Date accessed: May 25.

56. National Institute for Health and Care Excellence (NICE). Encorafenib with binimetinib for unresectable or metastatic BRAF V600 mutation-positive melanoma - Technology appraisal guidance [TA562], 2019. Available from: https://www.nice.org.uk/guidance/ta562. Date accessed: May 25.

57. National Institute for Health and Care Excellence (NICE). Abemaciclib with an aromatase inhibitor for previously untreated, hormone receptor-positive, HER2-negative, locally advanced or metastatic breast cancer - Technology appraisal guidance [TA563], 2019. Available from: https://www.nice.org.uk/guidance/ta563. Date accessed: May 25.

58. National Institute for Health and Care Excellence (NICE). Benralizumab for treating severe eosinophilic asthma - Technology appraisal guidance [TA565], 2019. Available from: https://www.nice.org.uk/guidance/ta565. Date accessed: May 25.

59. National Institute for Health and Care Excellence (NICE). Ustekinumab for treating moderately to severely active ulcerative colitis - Technology appraisal guidance [TA633], 2020. Available from: https://www.nice.org.uk/guidance/ta633. Date accessed: May 25.

60. National Institute for Health and Care Excellence (NICE). Brigatinib for ALK-positive advanced non-small-cell lung cancer that has not been previously treated with an ALK inhibitor - Technology appraisal guidance [TA670], 2021. Available from: https://www.nice.org.uk/guidance/ta670. Date accessed: May 25.

61. National Institute for Health and Care Excellence (NICE). Mepolizumab for treating severe eosinophilic asthma - Technology appraisal guidance [TA671], 2021. Available from: https://www.nice.org.uk/guidance/ta671. Date accessed: May 25.

For peer review only

62. National Institute for Health and Care Excellence (NICE). Anakinra for treating Still's disease - Technology appraisal guidance [TA685], 2021. Available from: https://www.nice.org.uk/guidance/ta685. Date accessed: May 25.

63. National Institute for Health and Care Excellence (NICE). Selective internal radiation therapies for treating hepatocellular carcinoma - Technology appraisal guidance [TA688], 2024. Available from: https://www.nice.org.uk/guidance/ta688. Date accessed: May 25.

64. National Institute for Health and Care Excellence (NICE). Atezolizumab monotherapy for untreated advanced non-small-cell lung cancer - Technology appraisal guidance [TA705], 2021. Available from: https://www.nice.org.uk/guidance/ta705. Date accessed: May 25.

65. National Institute for Health and Care Excellence (NICE). Diroximel fumarate for treating relapsing–remitting multiple sclerosis - Technology appraisal guidance [TA794], 2022. Available from: https://www.nice.org.uk/guidance/ta794. Date accessed: May 25.

66. National Institute for Health and Care Excellence (NICE). Somatrogon for treating growth disturbance in children and young people aged 3 years and over - Technology appraisal guidance [TA863], 2023. Available from: https://www.nice.org.uk/guidance/ta863. Date accessed: May 25.

67. National Institute For Health and Care Excellence (NICE). Vutrisiran for treating hereditary transthyretin-related amyloidosis - Technology appraisal guidance [TA868], 2023. Available from: https://www.nice.org.uk/guidance/ta868. Date accessed: Mar 24.

68. National Institute for Health and Care Excellence (NICE). Eptinezumab for preventing migraine - Technology appraisal guidance [TA871], 2023. Available from: https://www.nice.org.uk/guidance/ta871. Date accessed: May 25.

69. National Institute for Health and Care Excellence (NICE). Risankizumab for previously treated moderately to severely active Crohn's disease - Technology appraisal guidance [TA888], 2023. Available from: https://www.nice.org.uk/guidance/ta888. Date accessed: May 25.

70. National Institute for Health and Care Excellence (NICE). Pegunigalsidase alfa for treating Fabry disease - Technology appraisal guidance [TA915], 2023. Available from: https://www.nice.org.uk/guidance/ta915. Date accessed: May 25.

71. National Institute for Health and Care Excellence (NICE). Bimekizumab for treating active psoriatic arthritis - Technology appraisal guidance [TA916], 2023. Available from: https://www.nice.org.uk/guidance/ta916. Date accessed: May 25.

72. National Institute for Health and Care Excellence (NICE). Momelotinib for treating myelofibrosis-related splenomegaly or symptoms - Technology appraisal guidance [TA957], 2024. Available from: https://www.nice.org.uk/guidance/ta957. Date accessed: May 25.

73. National Institute for Health and Care Excellence (NICE). Atogepant for preventing migraine - Technology appraisal guidance [TA973], 2024. Available from: https://www.nice.org.uk/guidance/ta973. Date accessed: May 25.

74. National Institute for Health and Care Excellence (NICE). Selective internal radiation therapy with QuiremSpheres for treating unresectable advanced hepatocellular carcinoma - Technology appraisal guidance [TA985], 2024. Available from: https://www.nice.org.uk/guidance/ta985. Date accessed: May 25.

75. National Institute for Health and Care Excellence (NICE). Tenecteplase for treating acute ischaemic stroke - Technology appraisal guidance [TA990], 2024. Available from: https://www.nice.org.uk/guidance/ta990. Date accessed: May 25.

76. National Institute for Health and Care Excellence (NICE). Trastuzumab deruxtecan for treating HER2-low metastatic or unresectable breast cancer after chemotherapy - Technology appraisal guidance [TA992], 2024. Available from: https://www.nice.org.uk/guidance/ta992. Date accessed: May 25.

77. National Institute for Health and Care Excellence (NICE). Pembrolizumab with platinum- and fluoropyrimidine-based chemotherapy for untreated advanced HER2-negative gastric or gastro-oesophageal junction adenocarcinoma - Technology appraisal guidance [TA997], 2024. Available from: https://www.nice.org.uk/guidance/ta997. Date accessed: May 25.

78. National Institute for Health and Care Excellence (NICE). Evinacumab for treating homozygous familial hypercholesterolaemia in people 12 years and over - Technology appraisal guidance [TA1002], 2024. Available from: https://www.nice.org.uk/guidance/ta1002. Date accessed: May 25.

79. National Institute for Health and Care Excellence (NICE). Futibatinib for previously treated advanced cholangiocarcinoma with FGFR2 fusion or rearrangement - Technology appraisal guidance [TA1005], 2024. Available from: https://www.nice.org.uk/guidance/ta1005. Date accessed: May 25.

80. National Institute for Health and Care Excellence (NICE). Latanoprost–netarsudil for previously treated primary open-angle glaucoma or ocular hypertension Technology appraisal guidance [TA1009], 2024. Available from: https://www.nice.org.uk/guidance/ta1009. Date accessed: May 25.

81. National Institute for Health and Care Excellence (NICE). Fenfluramine for treating seizures associated with Lennox–Gastaut syndrome in people 2 years and over - Technology appraisal guidance [TA1050], 2025. Available from: https://www.nice.org.uk/guidance/ta1050. Date accessed: May 25.

82. National Institute for Health and Care Excellence (NICE). Tislelizumab for treating unresectable advanced oesophageal squamous cell cancer after platinum-based chemotherapy [ID4070], 2025. Available from: https://www.nice.org.uk/guidance/indevelopment/gid-ta11042. Date accessed: May 25.

83. National Institute for Health and Care Excellence (NICE). Somapacitan for treating growth hormone deficiency in people 3 to 17 years [ID6178], 2025. Available from: https://www.nice.org.uk/guidance/indevelopment/gid-ta11153. Date accessed: May 25.

84. National Institute for Health and Care Excellence (NICE). Guselkumab for treating moderately to severely active ulcerative colitis [ID6237], 2025. Available from: https://www.nice.org.uk/guidance/indevelopment/gid-ta11247. Date accessed: May 25.

85. National Institute for Health and Care Excellence (NICE). Guselkumab for previously treated moderately to severely active Crohn's disease [ID6238], 2025. Available from: https://www.nice.org.uk/guidance/indevelopment/gid-ta11245. Date accessed: May 25.

86. National Institute for Health and Care Excellence (NICE). Mirikizumab for treating moderately to severely active Crohn's disease [ID6244], 2025. Available from: https://www.nice.org.uk/guidance/indevelopment/gid-ta11267. Date accessed: May 25.

For peer review only

87. National Institute for Health and Care Excellence (NICE). Nivolumab as neoadjuvant (with chemotherapy) and adjuvant (as monotherapy) treatment for resectable non-small-cell lung cancer [ID6310], 2025. Available from: https://www.nice.org.uk/guidance/indevelopment/gid-ta11504. Date accessed: May 25.

88. National Institute for Health and Care Excellence (NICE). Vanzacaftor–tezacaftor–deutivacaftor for treating cystic fibrosis with 1 or more F508del mutations in the CFTR gene in people aged 6 years and over [ID6372], 2025. Available from: https://www.nice.org.uk/guidance/indevelopment/gid-ta11430. Date accessed: May 25.

89. National Institute for Health and Care Excellence (NICE). Abiraterone (originator and generics) for treating newly diagnosed high-risk hormone-sensitive metastatic prostate cancer (review of TA721) [ID6378], 2025. Available from: https://www.nice.org.uk/guidance/indevelopment/gid-ta11730. Date accessed: May 25.

90. National Institute for Health and Care Excellence (NICE). Rilzabrutinib for treating persistent or chronic immune thrombocytopenia in people aged 12 and over [ID6395], 2025. Available from: https://www.nice.org.uk/guidance/awaiting-development/gid-ta11491. Date accessed: May 25.

91. National Institute for Health and Care Excellence (NICE). Darolutamide with androgen deprivation therapy for treating hormone-sensitive metastatic prostate cancer ID6452, 2025. Available from: https://www.nice.org.uk/guidance/indevelopment/gid-ta11557. Date accessed: May 25.

92. National Institute for Health and Care Excellence (NICE). Empagliflozin for treating chronic heart failure with reduced ejection fraction - Technology appraisal guidance [TA773], 2022. Available from: https://www.nice.org.uk/guidance/ta773. Date accessed: May 25.

93. National Institute for Health and Care Excellence (NICE). Empagliflozin for treating chronic heart failure with preserved or mildly reduced ejection fraction - Technology appraisal guidance [TA929] - Final draft guidance. Available from: https://www.nice.org.uk/guidance/ta929/history. Date accessed: Apr 25.

94. National Institute for Health and Care Excellence (NICE). NICE health technology evaluations: the manual, 2023. Available from: https://www.nice.org.uk/process/pmg36. Date accessed: Apr 25.

95. Costello Medical. NICE's 'Proportionate Approach to Technology Appraisals (PATT)': The Story So Far, 2024. Available from: https://www.costellomedical.com/what-we-do/value-and-access/hta/nices-proportionate-approach-to-technology-appraisals/. Date accessed: May 25.

For peer review only

BMJ TAG

Evaluating clinical similarity: a systematic review of NICE guidance and the development of a Bayesian framework for a formal assessment of clinical similarity

Protocol

April 2025

For peer review only

1 Title of the project

Evaluating clinical similarity: a systematic review of NICE guidance and the development of a Bayesian framework for a formal assessment of clinical similarity.

2 Name of TAR team and project 'lead'

BMJ Technology Assessment Group (BMJ-TAG)

Lead: Steve Edwards

Director of Health Technology Assessment

BMJ Group

5th Floor, 90 Whitfield Street, London W1T 4EZ

For peer review only

For peer review only - http://bmjopen.bmj.com/site/about/guidelines.xhtml

3 Plain English Summary

Cost-comparison evaluations (CCEs) are one type of technology appraisal being used by the National Institute for Health and Care Excellence (NICE) to assess the clinical and cost effectiveness of new treatments. It allows new treatments to be compared against a comparator treatment in the same indication that has already been recommended by NICE and is likely to be "clinically similar", with no requirement for complex health economic modelling and a process that aims to be faster than assessments requiring more complex modelling.

Deciding whether two treatments are "clinically similar" can be difficult if there are no trials directly comparing the two treatments and an indirect treatment comparison (ITC) has been required. ITCs often report no statistically significant difference, which cannot itself be interpreted as evidence of two treatments being clinically similar. There is thought to be variation in terms of how clinical similarity is currently assessed in these cases within NICE CCEs. The availability of thresholds such as minimally clinically important differences (MCIDs) may help with the interpretation of clinical similarity, but these are not always available and it is unclear how often these or other methods are used to support decisions about clinical similarity within NICE CCEs.

This research aims to review all CCEs performed as part of the NICE technology appraisal programme since 2017 to identify and summarise any variations in the methods used to support decisions about clinical similarity when ITCs have been required. Subsequent to this, a novel Bayesian framework will be developed with the aim of supporting the interpretation of non-significant results from Bayesian network meta-analyses (NMAs). Worked examples demonstrating the use of the new tool will also be provided.

For peer review only

For peer review only - http://bmjopen.bmj.com/site/about/guidelines.xhtml

4 Research question

4.1 Purpose

Technology appraisals, such as those performed in England as part of the National Institute for Health and Care Excellence (NICE) technology appraisal programme, involve the assessment of the clinical and cost-effectiveness of specific drugs, technologies or other interventions. Most appraisals require complex health economic models, such as cost-utility analyses, to assess whether the intervention of interest is likely to be a good use of NHS resources. However, since 2017, cost comparison evaluations (CCEs) have been accepted within the NICE technology appraisal programme under specific circumstances. CCEs involve a simpler comparison of costs and resources used with one drug compared to another, without the need for a complex model such as those used in cost-utility analyses, but they require a strong assumption that the drugs being compared are similar in terms of clinical effectiveness.

CCEs were first introduced as one option under the fast-track appraisal (FTA) process in 2017; while the FTA process itself was replaced in 2022, its replacement was a process focusing only on CCEs and methods relating to the CCE itself did not notably change. For an appraisal to be suitable for the cost comparison route, Section 4.2.13 of the 2022 NICE health technology evaluations manual states that the new interventions should be, "likely to provide similar or greater health benefits at similar or lower cost than the relevant comparator(s)"; relevant comparators are considered to be, "those recommended in published NICE guidance for the same population",[1] which is consistent with requirements described for the CCE process prior to 2022.[2] The aim of CCEs is to provide an assessment of clinical and cost-effectiveness for suitable drugs that can be used to decide whether the they should be recommended but in a shorter time frame and using fewer resources compared to technology appraisals using cost-utility methods, as part of NICE's commitment to taking a proportionate approach to technology appraisals.[3]

While the clinical similarity of two interventions can be robustly demonstrated through non-inferiority randomised controlled trials (RCTs) using prespecified non-inferiority thresholds or clinical equivalence RCTs,[4] many CCEs submitted to NICE do not involve such trials. Instead, they rely on the results of indirect treatment comparisons (ITCs) such as network meta-analyses (NMAs), matching-adjusted indirect comparisons (MAICs) or simple Bucher ITCs. These analyses often result in non-

For peer review only - http://bmjopen.bmj.com/site/about/guidelines.xhtml

significant results based on confidence intervals or credible intervals that cross the line of null effect. A lack of a statistically significant difference should not be used to determine that two treatments are clinically similar; these analyses are often based on a small number of studies with small sample sizes, meaning there is considerable uncertainty in the effect estimate that should not be interpreted as an absence of a difference. Therefore, the interpretation of these results in terms of whether interventions can be considered clinically similar can be challenging. The use of minimally clinically important differences (MCIDs) established in the literature or estimated through other methods may aid the interpretation of results and the transparency of this process;[5] if the point estimates and uncertainty interval obtained from the ITC were less than (or mostly less than) the MCID, it may increase the confidence of decision-makers that the treatments are most likely to be clinically similar, as any difference that is present is unlikely to be clinically meaningful. Alternatively, if the point estimate and uncertainty interval are greater than the MCID and in favour of the new intervention, there would be increased confidence that the new treatment may be better than the previously recommended intervention. However, established MCIDs are not available for all outcomes and it is unclear how often these methods are used currently to assess clinical similarity in NICE CCEs.

Through a systematic literature review (SLR), this research aims to review and summarise the basis for decision-makers assuming clinical similarity based on non-significant results obtained from ITCs as part of NICE CCEs, such as how often MCIDs or other approaches are used for interpretation and decision-making, and which thresholds have been used so far. The results of the SLR will support the development of a novel Bayesian framework that can integrate MCIDs or other thresholds to support the interpretation of non-significant results from Bayesian NMAs. Worked examples demonstrating the use of the tool, taken from the CCEs identified in the SLR, will be provided as part of this research. The SLR will highlight whether inconsistencies exist in current decisions or whether thresholds for clinical similarity have emerged organically from decision-makers, while the proposed framework should allow for consistent, robust, and reproducible decision-making for future CCEs.

For peer review only - http://bmjopen.bmj.com/site/about/guidelines.xhtml

4.2 Content of the systematic literature review

The SLR will aim to identify and include all NICE technology appraisals that:

- have followed the CCE approach for decision-making from the outset;
- rely on non-significant results from ITCs to assess clinical similarity for at least one comparator for the primary outcome(s);
- have published final guidance at the time of searching or where this can be obtained via NICE for appraisals due to publish final guidance imminently (i.e. before completion of the SLR).

Appraisals that only involve a non-inferiority or equivalence RCT (i.e. there are no comparators for which an ITC has been required) will not be included given these trials are considered to be robust methods of assessing clinical similarity and are not subject to the same uncertainties as results from ITCs. Similarly, appraisals that only include ITCs with statistically significant results will not be included, as there is less uncertainty associated with these results. Appraisals that include direct RCT evidence or statistically significant ITC results for one comparator but rely on the non-significant results of an ITC for another comparator will only be included if there are clear statements about the committee or external assessment group (EAG)'s conclusion of clinical similarity for the comparison where the ITC with non-significant results was performed; this is because clinical similarity only has to be demonstrated against one comparator in the NICE CCE process, meaning if multiple comparators are deemed appropriate, the decision-making is likely to have been based mostly on the one with the strongest evidence (in this case the one with direct RCT evidence or statistically significant ITC results). During the sifting stage, details about which appraisals involved decisions based on statistically significant results from ITCs or direct comparisons between treatments (and whether these were superiority, non-inferiority or equivalence trials) will be recorded, to allow brief comments to be made on this despite their exclusion from the main review.

Appraisals that have followed the CCE process from the start of the appraisal (i.e. where the company's first submission is a CCE) will be prioritised for inclusion. It is possible that some Single Technology Appraisals (STAs) start with cost-utility models but incorporate cost

For peer review only - http://bmjopen.bmj.com/site/about/guidelines.xhtml

comparison approaches following requests from the EAG or committee. If time permits, these will be considered for inclusion but only if it is clear that decision-making was solely based on the cost comparison assessment given the exact contribution of the CCE to decision-making would otherwise be difficult to disentangle. An appraisal that has been a CCE from the start will usually be evident from the introductory pages of the NICE committee papers or the decision problem table submitted by the company, but other sections of the appraisal documents will be explored where this is unclear. Conclusions about whether decisions were based solely on cost comparison analyses for STAs may be available from committee discussion sections included in final guidance documents, but where this is unclear the appraisal will be excluded.

The review will aim to cover all CCEs meeting the above criteria from 2017 onwards; however, upon identification of relevant appraisals, prioritisation of those from 2022 onwards (given these represent the most recent examples of the NICE CCE process) may be required if the workload associated with this outweighs the resources available for this project. Due to the time constraints of this review, only the primary outcome(s) in each appraisal will be assessed with regards to how the decision of clinical similarity was made. It is anticipated that the documenting of which outcome is the primary outcome may be unclear in some appraisals; in this review, if not otherwise stated, the outcome that is discussed first, is most heavily discussed in the report or NICE committee meeting slides and/or is key in terms of any economic assessment presented will be considered to be the primary outcome. In cases where it appears there is more than one primary outcome, these will be considered separately. A clear rationale for why specific outcomes in each appraisal have been interpreted as the primary outcome will be included in the extraction tables.

4.3 Development of a Bayesian framework for a formal assessment of clinical similarity

The results of the SLR described in Section 4.2 will be used to support the development of a novel framework that utilises Bayesian NMAs to allow non-significant results to be interpreted more robustly. The framework will allow the inclusion of MCIDs or other thresholds when available to support with the interpretation of non-significant results. It is anticipated that this will involve building on existing, commonly used approaches such as cumulative density functions, point estimates, and credible intervals. Outputs will be designed such that they are clear and easily

For peer review only - http://bmjopen.bmj.com/site/about/guidelines.xhtml

interpretable by stakeholders regardless of their statistical background. Worked examples demonstrating the use of the tool, taken from the CCEs identified in the SLR, will also be provided as part of this research. The methods for this phase of the research are not described further in this protocol but will be written up in the final report.

5 Methods for the systematic literature review

Methods described here are specifically for the systematic literature review (SLR) phase of this research, as outlined in Section 4.2. More detailed methods associated with the development of the Bayesian framework outlined in Section 4.3 are not included here but will be covered in the final report.

The SLR will be performed and reported according to the PRISMA statement. A flow diagram illustrating the number of records identified, included and excluded throughout the SLR will be presented according to the PRISMA reporting guidelines.[6]

5.1 Search strategy

Multiple free-text searches of the National Institute for Health and Care Excellence (NICE) website will be performed by one reviewer. The terms "cost comparison", "cost minimisation" and "cost minimization" (including the quotation marks) will be searched using the search bar at the top of the main NICE webpage (https://www.nice.org.uk/). This will pick up any records including the exact terms "cost comparison" or "cost-comparison" while avoiding records that mention cost and comparison but not adjacently (or equivalent for cost minimisation and cost minimization). This is considered to be a reasonable approach given the term "cost comparison" is a formal and consistent term used by NICE in these appraisals and is likely to be mentioned somewhere in all appraisals that have followed the cost comparison evaluation (CCE) route. The terms "cost minimisation" and "cost minimization" will be included to identify any records that may have used this terminology instead, which can also be used to refer to the cost comparison approach in health economic assessment.

The results of the search will be further refined to show only technology appraisal guidance; the option to filter by guidance programme on the left-hand side of the search results page will be used

For peer review only - http://bmjopen.bmj.com/site/about/guidelines.xhtml

to achieve this by selecting the “technology appraisal guidance” box. All records remaining after applying this filter will be screened for inclusion against the criteria outlined in Section 4.2.

For records that have not had final guidance published at the time of screening and that are due to publish before completion of the SLR, one reviewer will contact NICE to determine whether information on final guidance can be obtained to allow inclusion in this assessment. During the screening process, the contact person for appraisals that have not yet published final guidance will be captured to facilitate this. NICE will also be contacted with a full list of CCEs identified from the search of the NICE website, to ask whether it is aware of any other CCEs not captured by the search.

5.2 Review process

Given it is not possible to export records from the NICE website, records obtained from the search described in Section 5.1 will be manually added to a Microsoft Excel® spreadsheet for tracking and screening purposes. Records will then be screened for inclusion against the criteria outlined in Section 4.2 by a single reviewer. This will involve reviewing any information and documents published on the NICE website (or provided in communication with NICE) for each record in a single screening step; information within guidance documents and committee papers will be prioritised, but other documents such as final scopes may be reviewed if information remains unclear or if other documents with key information are identified. Validation of this process will be performed by a second reviewer. Any disagreement will be resolved through discussion and involvement of a third reviewer if required to resolve outstanding conflicts.

5.3 Data extraction

Data for each included record will be extracted by a single reviewer into standardised data extraction forms using Microsoft Word®. Validation of a representative 30% sample of extractions will be performed by a second reviewer, with discussion of any discrepancies and involvement of a third reviewer if required. A draft data extraction form is included in Appendix 8.1 but is subject to change; a pilot of five extractions will be performed before validation by a second reviewer, after which the data extraction form will be finalised and used for the remaining extractions.

General information about each record such as project name, project ID and disease area will be extracted, as well as information about primary outcomes, comparators that the ITC was required

For peer review only - http://bmjopen.bmj.com/site/about/guidelines.xhtml

for and type of ITC performed, date of final guidance publication and whether or not the treatment was recommended. Information about clinical similarity and reasons that decisions were made are likely to be reported in various ways across projects, but reviewers will be exhaustive and as a minimum will capture any information relating to:

- Any mention of ways of interpreting ITC results such as specific MCIDs or other thresholds and how these were identified;
- Results of the committee's preferred ITCs for relevant comparisons, including point estimates and measures of uncertainty (such as 95% confidence or credible intervals) of the effect estimates;
- Any committee or EAG comments on the uncertainty in the ITC, including what they thought about the uncertainty in the ITC and how it may have impacted their decision or conclusions;
- Brief comments on the availability of input data for the ITCs from NICE papers, to allow an assessment of how feasible it would be to replicate the ITCs from this information (and whether identification of data from individual publications would instead be required) for the worked Bayesian framework examples that will be provided as part of the second phase of this project;
- Narrative comments within company submissions, EAG reports or committee discussion documents describing how results of ITCs were ultimately interpreted and how clinical similarity was confirmed or refuted in the final decision-making process; clear statements from committee discussions will be most useful, but in the absence of this inferences from EAG reports about preferred analyses or interpretation may be required.

Extractions will include details on the location that each piece of information was identified to facilitate validation. Where multiple versions of an ITC are available across different documents (for example, an ITC performed within the company submission may have been adapted or amended by the EAG), methods and results relating to the committee's preferred analysis only will be extracted, which should be evident from the guidance documents. Where this information is uncertain, the EAG's preferred analysis will be preferentially extracted unless there is evidence from supporting information that the committee dismissed this analysis in favour of the company's original analysis.

For peer review only - http://bmjopen.bmj.com/site/about/guidelines.xhtml

Details related to interpretation and decision making will be captured using quotation marks in extraction tables to ensure that detail is not lost during this process. Guidance documents and committee papers available on the NICE website will be prioritised for review during data extraction but review of other documents such as final scopes may be required if information remains unclear or if other documents with key information are identified. Any additional information provided by NICE will be considered where applicable.

5.4 Quality assessment

Given that the records to be included in this review are NICE technology appraisals rather than clinical trials such as randomised controlled trials (RCTs), non-randomised studies or qualitative studies, a quality assessment process will not be incorporated as part of this review.

5.5 Synthesis

Information extracted from included records will be summarised narratively and using summary tables, with similarities and differences across the identified CCEs noted. The narrative synthesis will focus mostly on the ways in which clinical similarity was confirmed or refuted, including:

- similarities and differences across appraisals with regards to methods used, such as specific thresholds that have been used and how they were derived (e.g. elicitation, arbitrary or other) or other methods of assessing clinical similarity;
- the rationale for the methods used;
- how common each method of assessing clinical similarity is;
- whether there are any differences in how similar methods or thresholds have been interpreted across appraisals.

Any other key aspects identified throughout the review may also be covered. Specific CCEs may be highlighted as examples of different methods used to assess clinical similarity, such as use of MCIDs or other thresholds. Key strengths, limitations and uncertainties will be highlighted and summarised where appropriate.

For peer review only - http://bmjopen.bmj.com/site/about/guidelines.xhtml

6 Contribution of the research group:

Steve Edwards	Director of Health Technology Assessment, BMJ-TAG, London. Conception of the research project and provision of research framework. Guidance and validation of the work of the research group at all stages; provision of feedback on all versions of the protocol and the report. Guarantor of the report.
Clare Dadswell	Clinical Evidence Manager, BMJ-TAG, London. Contribution to the development of the project. Providing feedback to resolve any outstanding conflict between the first two reviewers on the systematic literature review.
Nicole Downes	Senior Clinical Evidence Analyst, BMJ-TAG, London. Lead on the development of this protocol. Lead reviewer on the systematic literature review of NICE guidance, including development of search strategy, running searches, sifting of results, data extraction and narrative synthesis of results. Drafting sections of the final report related to the systematic literature review.
Ben Burgess	Senior Clinical Evidence Analyst, BMJ-TAG, London. Leading on the development of a Bayesian framework for a formal assessment of clinical similarity. Testing the developed framework using examples identified through the systematic literature review. Drafting sections of the final report related to the Bayesian framework.
Archie Walters	Health Economist, BMJ-TAG, London. Second reviewer on the systematic literature review of NICE guidance, involved in validation of the screening stage and validation of a proportion of the extractions.
Sophie Ip	Senior Health Economist, BMJ-TAG, London. Supporting during the Bayesian framework development stage by validating code and analyses.

For peer review only - http://bmjopen.bmj.com/site/about/guidelines.xhtml

7 Timetable/Milestones

Key dates for this project are outlined in Table 1 below and are mostly related to the systematic literature review (SLR) phase of this research.

Table 1 Draft milestones table

Milestone	Start date	End date
Project start date	07/04/2025	07/04/2025
Draft protocol development	07/04/2025	11/04/2025
SLR searching, sifting, extraction and validation	14/04/2025	02/05/2025
SLR synthesis and report writing	06/05/2025	06/06/2025
Work on Bayesian framework including report writing	07/04/2025	18/06/2025
Project submission	19/06/2025	19/06/2025

8 Appendices

8.1 Draft data extraction form

Appraisal	Appraisal name (ID) {reference}
Appraisal details	**Comparator(s) included:**<br>• **ITC required for:**<br><br>**Source:**
	**Outcomes included in the ITCs:**<br><br>**Outcome(s) considered to be primary outcomes by reviewer:**<br>•<br>•<br>•<br><br>(defined as primary outcomes…)<br><br>**Outcome scales and thresholds:**<br>•<br>•<br>•<br><br>**Source:**
	**Final appraisal decision:**

For peer review only - http://bmjopen.bmj.com/site/about/guidelines.xhtml

	**Source:**
Details of committee's preferred ITCs	**Preferred ITCs:**<br><br>**Source:**
ITC results – committee's preferred analyses	***Comparison 1 -***<br>***Outcome 1***<br>• **Effect estimate from ITC:** (e.g. RR and 95% CI)<br>• **Statistical significance from ITC:**<br>• **Source:**<br>• **Availability of ITC input data for replication:**<br><br>***Outcome 2***<br>• **Effect estimate from ITC:**<br>• **Statistical significance from ITC:**<br>• **Source:**<br>• **Availability of ITC input data for replication:**<br><br>***Outcome 3***<br>• **Effect estimate from ITC:**<br>• **Statistical significance from ITC:**<br>• **Source:**<br>• **Availability of ITC input data for replication:**
	Comparison 2<br>***Outcome 1***<br>• **Effect estimate from ITC:**<br>• **Statistical significance from ITC:**<br>• **Source:**<br>• **Availability of ITC input data for replication:**
	Comparison 3<br>***Outcome 1***<br>• **Effect estimate from ITC:**<br>• **Statistical significance from ITC:**<br>• **Source:**<br>• **Availability of ITC input data for replication:**

For peer review only - http://bmjopen.bmj.com/site/about/guidelines.xhtml

<table>
<tr><td rowspan="3">Methods used to assess clinical similarity and conclusions</td><td>Company approach
Method used and rationale:
Details of thresholds used:
Conclusions for each outcome:
Uncertainty in the ITC results and impact on decision-making:
Comments:
Source:</td></tr>
<tr><td>EAG approach
Method used and rationale:
Details of thresholds used:
Conclusions for each outcome:
Uncertainty in the ITC results and impact on decision-making:
Comments:
Source:</td></tr>
<tr><td>Committee approach
Method used and rationale:
Details of thresholds used:
Conclusions for each outcome:
Uncertainty in the ITC results and impact on decision-making:
Comments:
Source:</td></tr>
<tr><td>Comments</td><td></td></tr>
<tr><td colspan="2">Abbreviations:</td></tr>
</table>

9 References

1. National Institute for Health and Care Excellence (NICE). NICE health technology evaluations: the manual, 2023. Available from: https://www.nice.org.uk/process/pmg36. Date accessed: Apr 25.
2. National Institute for Health and Care Excellence (NICE). Technology appraisal methods and processes used before 2022, 2022. Available from: https://www.nice.org.uk/about/what-we-do/our-programmes/nice-guidance/nice-technology-appraisal-guidance/process. Date accessed: Apr 25.
3. National Institute for Health and Care Excellence (NICE). Taking a proportionate approach to technology appraisals, 2024. Available from: https://www.nice.org.uk/about/what-we-do/proportionate-approach-to-technology-appraisals. Date accessed: Apr 25.
4. Walker E, Nowacki AS. Understanding equivalence and noninferiority testing. *J Gen Intern Med* 2011; **26**: 192-6.
5. Klukowska AM, Vandertop WP, Schröder ML, Staartjes VE. Calculation of the minimum clinically important difference (MCID) using different methodologies: case study and practical guide. *Eur Spine J* 2024; **33**: 3388-400.
6. Page MJ, McKenzie JE, Bossuyt PM, Boutron I, Hoffmann TC, Mulrow CD, et al. The PRISMA 2020 statement: an updated guideline for reporting systematic reviews. *BMJ* 2021; **372**: n71

For peer review only - http://bmjopen.bmj.com/site/about/guidelines.xhtml

Online Supplementary File 3

Point-and-density plot methods

Development of point-and-density plots

The framework (i.e., point-and-density plots) described in this research does not seek to replace any current methods with regards to performing Bayesian network meta-analyses (NMAs). Indeed, it is recommended that all National Institute for Care and Health Excellence (NICE) Decision Support Unit (DSU) guidelines[1] with regards to indirect treatment comparisons continue to be followed. Instead, the proposed framework should be viewed as an add-on to existing approaches for NMAs, with all relevant procedures and outputs continuing to be provided (e.g., forest plots, trace plots, and Gelman-Rubin diagrams). As such, the following detailed methodology may be considered an 'add-on' analysis to be specifically utilised for NICE Cost Comparison Evaluations (CCEs) and other Technology Appraisals (TA) where an assessment of clinical similarity, or non-inferiority, is required.

The statistics underpinning the generation of point-and-density plots are provided below.

When performing a Bayesian NMA, density plots are generated for each comparison in the network, an example of which is shown in Figure 1, which is an example of the default output of the *gemtc* R package.[2] Such a density plot is generated using the values of iterations from across all chains used within a Bayesian analysis and can be produced for each treatment comparison.

For peer review only

Figure 1. Default density plot from the *gemtc* R package for the mean difference between an example treatment and comparator.

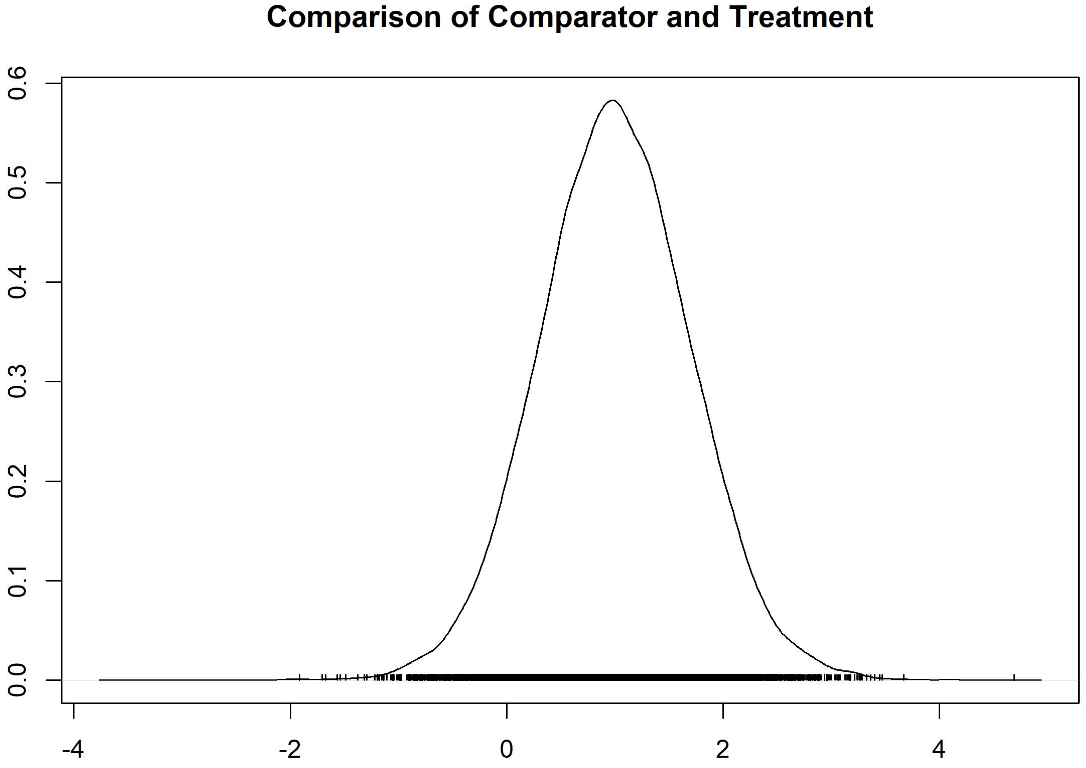

The default density plots produced by the *gemtc* R package[2] have minimal labels. Within Figure 1, the x-axis denotes the mean difference of the comparison of the example comparator to the example treatment, while the y-axis denotes the corresponding density.

For each treatment comparison (e.g., the example comparator to the example treatment in Figure 1) it is possible to generate an empirical cumulative density function (ECDF) from the density plot. An example of such ECDF is shown in Figure 2. In contrast to a regular density plot, an ECDF shows the cumulative density across all values (e.g., mean difference in Figure 2). Within the example from Figure 2, the ECDF will have a cumulative density of 0 at the minimum mean difference reported for the comparison of the example comparator to the example treatment. Likewise, within the example from Figure 2, the ECDF will have a cumulative density of 1 at the maximum mean difference reported for the comparison of the example comparator to the example treatment. Within the example from Figure 2, the ECDF can be used to determine the cumulative density of any point between the minimum and maximum mean difference reported for the comparison of the example comparator to the example treatment. Accordingly, by specifying a value that corresponds to a non-inferiority margin (NIM), minimal clinically important difference (MCID), or other threshold, it is possible to determine the cumulative density of points that fall below, and above, this threshold. Figure 2 shows an example of the application of a NIM to an ECDF, where a NIM of -0.20 is specified. As illustrated in Figure 3, the NIM of -0.20 would correspond to a cumulative density of 0.041.

Figure 2. Empirical cumulative density function for the comparison of an example treatment and comparator.

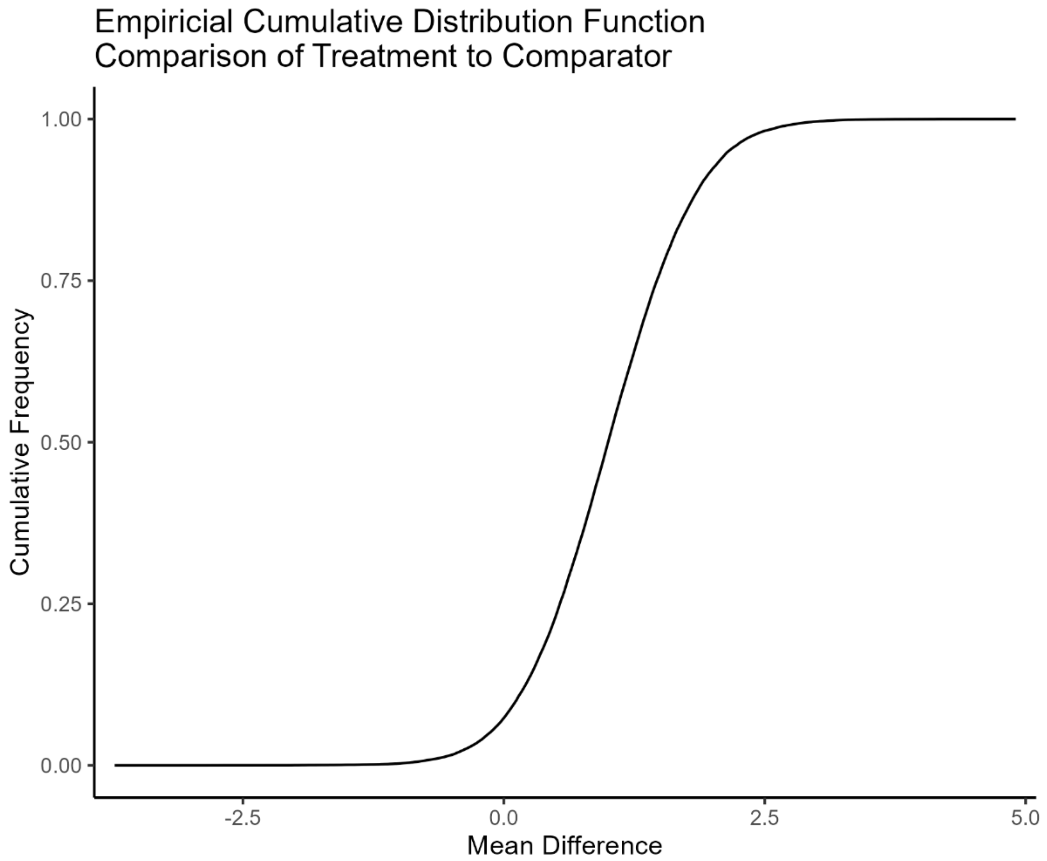

For peer review only

Figure 3. Empirical cumulative density function, with a non-inferiority margin, for the comparison of an example treatment and comparator.

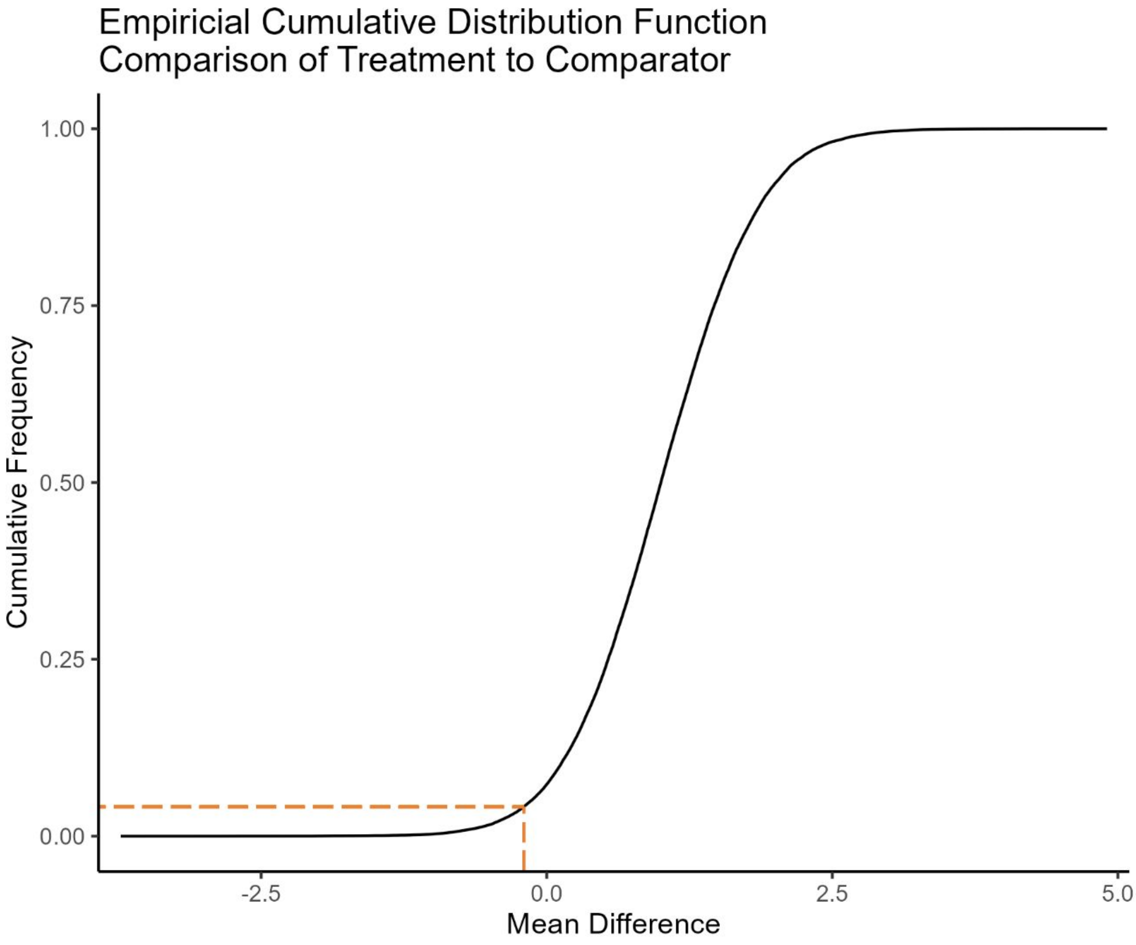

Dashed orange line corresponds to a non-inferiority margin of -0.20 and a corresponding cumulative density of 0.041.

Given the nature of Bayesian analyses, the ECDF for a given comparison can be used to determine the proportion of iterations from the Bayesian analysis that fall below a given threshold (e.g., NIM). Accordingly, within the example shown in Figure 3, the cumulative density represents the probability that the mean difference of the comparison of the example comparator to the example treatment falls below the given threshold. As such, the probability that the mean difference of the comparison falls above the given threshold can be calculated. Therefore, by combining the results of the ECDF with those of a traditional density plot and forest plot, it is possible to convey the results of all three separate analyses within a single readily interpretable figure (i.e., a point-and-density plot). An example of a point-and-density plot is shown in Figure 4, again using the comparison of the example comparator to the example treatment as above.

Figure 4. Point-and-density plot for the comparison of an example treatment and comparator.

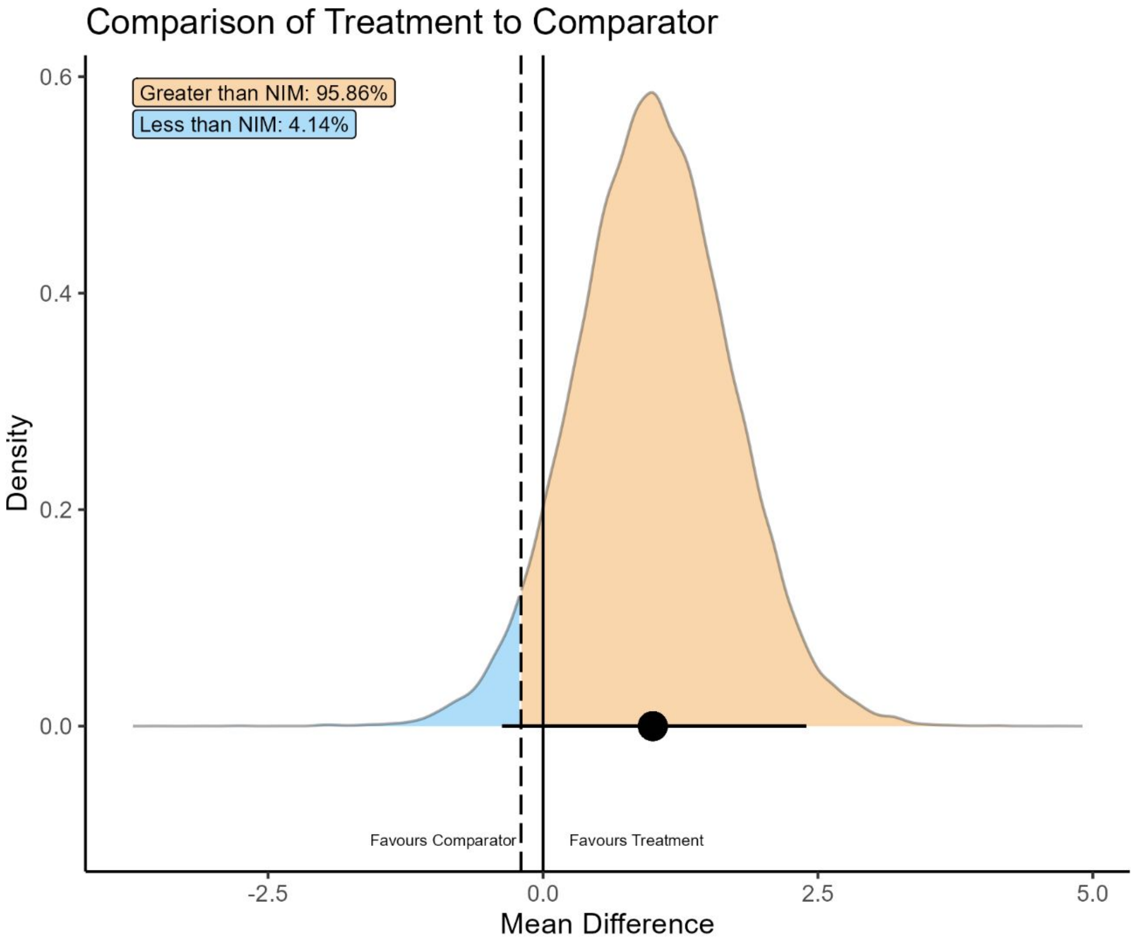

The threshold for traditional hypothesis testing is shown by the solid line, while the dashed line indicates the NIM. The area that falls under (above) the NIM is shown in blue (orange). The probability that the effect size falls under, or above, the NIM is shown in the upper left corner of the plot. The pooled estimate is shown by the black circle, with the error bars corresponding to 95% credible intervals.

Point-and-density plots have been designed to be readily interpretable and showcase data not previously reported from Bayesian NMAs, in addition to data from existing figures (e.g., forest plots). Within Figure 4, the thick black line denotes the threshold for traditional hypothesis testing (i.e., either 0 or 1 depending on the effect estimate), while the dashed black line denotes the specified NIM, MCID, or another designated threshold. The point estimate, denoted by the black circle, indicates the pooled estimate for the comparison, with the error bars indicating 95% credible intervals. The point estimate shown within the point-and-density plot will be the same as that shown within a typical forest plot. As shown by Figure 4, the density curve aligns with that shown from the typical output of the *gemtc* R package[2] (Figure 1). The area under the density curve that falls above and below the NIM is shown in blue and orange, respectively. Likewise, the probability that the effect estimate for the comparison falls above or below the NIM (dashed black line) is shown in the top-left corner, with these values being derived using the corresponding ECDF. Furthermore, within

the point-and-density figure, labels have been added to denote whether a given effect size favours the treatment or the comparator.

As shown by Figure 4, the pooled estimate for the comparison of the example comparator to the example treatment is 1.00, with 95% credible intervals of -0.34 to 2.40. Accordingly, based solely on traditional statistical testing, there was no statistically significant difference in treatment effects between the example comparator and the example treatment. In addition, the 95% credible intervals overlap the specified NIM of -0.20, indicating that non-inferiority of the example treatment compared to the example comparator is not demonstrated. However, through the use of the ECDF, the point-and-density plot (Figure 4) depicts that there was a 95.87% probability of the example treatment being non-inferior to the example comparator. It is suggested that committees initially focus on whether the 95% credible intervals overlap the NIM to determine whether a treatment can be considered to be non-inferior. However, as in the worked example, the use of probabilities of non-inferiority may provide valuable information, particularly where non-inferiority has not been demonstrated. Additionally, the plots of ECDF functions do not necessarily need to be presented within CCE submissions; instead, it is recommended that committees primarily focus on the results of point-and-density figures.

The above example of the comparison of the example comparator to the example treatment uses a NIM of -0.20. However, for some comparisons a NIM, MCID, or other designated threshold may not be available. Accordingly, when clinically validated thresholds are not available, it is recommended that the corresponding threshold be set to either 0 (for effect estimates such as mean difference) or 1 (for effect estimates such as hazard ratios or odds ratios). By using this approach it is possible to determine the probability that the effect size for the comparison of two treatments will fall above, or below, the corresponding threshold. However, it should be noted that this approach will result in more conservative estimations of non-inferiority compared to using a clinically validated NIM, MCID, or another relevant threshold. Furthermore, in situations where a NIM, or similar threshold, is not reported, by using the corresponding ECDF it is possible to determine a hypothetical threshold. Such a hypothetical threshold can be used to determine the required threshold for a given, arbitrary, probability that a treatment is non-inferior to a comparator. For instance, using the above example, comparing the example comparator to the example treatment, the ECDF reveals that a hypothetical threshold of -0.65 would be required for there to be 99% probability that the example treatment was non-inferior to the example comparator. However, it is important to note that such a hypothetical threshold should not be considered to be clinically appropriate or meaningful. However, in the absence of NIM, MCID, or other designated threshold, a hypothetical threshold can help inform a committee by indicating whether a

For peer review only

relatively large, or small, hypothetical threshold would be required for there to be a certain, arbitrary, probability that a treatment is non-inferior to a comparator. Ultimately, when deriving a hypothetical threshold, the selection of the desired probability of non-inferiority may be arbitrarily selected. However, the authors suggest that a 95% probability of non-inferiority may be selected for a hypothetical threshold. In doing so, a 95% probability of non-inferiority aligns with the typical 95% threshold used for statistical significance testing. Importantly, the selection of a 95% threshold represents a 'high bar' for any assessments of non-inferiority in the absence of a clinically validated NIM or MCID.

Code

The code to perform the analyses described above has been developed in R v4.2.0[3] and utilises the *gemtc*[2]*, data.table*[4]*, igraph*[5]*, tidyverse*[6]*, magrittr*[7]*, and ggplot2*[8] *packages.* The code was developed by one researcher and subsequently independently reviewed and tested by a second researcher on two separate occasions. The R code was tested using synthetic datasets to ensure that it worked as expected for a variety of link functions (e.g., *cloglog*, *identity*, *logit*, and *log*) and data types (e.g., mean and standard deviations or the overall number of patients and the number of responders).

The results of the code were also compared to the *gemtcPlus* package[9] which allows users to determine the probability of that an effect size falls above, or below, a threshold of 0 or 1. While users of the *gemtcPlus* package[9] cannot specify an alternative threshold, comparisons of the results obtained using the *gemtcPlus* package[9] and the approach detailed here aligns.

R code and NMA output to generate example point-and-density plots from the case study for crovalimab for Paroxysmal Nocturnal Haemoglobinuria (TA1019)[10] are provided as supplementary materials.

Crovalimab for Paroxysmal Nocturnal Haemoglobinuria (TA1019)

Network of evidence

Within TA1019[10], the company identified a connected evidence network (Figure 5) that comprised crovalimab and the comparators of eculizumab, ravulizumab, and standard of care for the endpoint of transfusion avoidance.

For peer review only

Figure 5. Network of evidence for studies included within network meta-analyses for the percentage of patients achieving transfusion avoidance for the overall population.

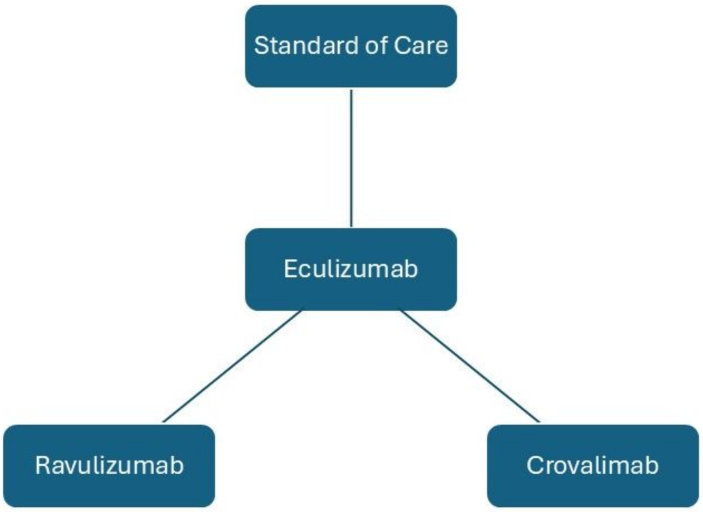

Raw data

The raw data used to perform the original analyses was reported in TA1019[10], as such these data were used to perform the re-analyses for the overall population. Data were extracted from TA1019[10] by one researcher and independently verified by a second researcher. Overall, the raw data used in the original analysis and re-analysis are shown in Table 1.

Table 1. Data used in the analyses and re-analyses of the network meta-analyses for the percentage of patients achieving transfusion avoidance (taken from page 62 of the company submission).

Study	Population	Treatment	Mean	Standard Error
Study 301[11]				
	Naïve	Ravulizumab	0.736	0.039
	Naïve	Eculizumab	0.661	0.043
TRIUMPH[12]				
	Naïve	Eculizumab	0.51	0.078
	Naïve	Standard of Care	0	0.025
COMMODORE 2[13]				
	Naïve	Crovalimab	0.664	0.042
	Naïve	Eculizumab	0.681	0.058
Study 302[14]				
	Experienced	Ravulizumab	0.876	0.033
	Experienced	Eculizumab	0.827	0.038
COMMODORE 1[15]				
	Experienced	Crovalimab	0.795	0.069
	Experienced	Eculizumab	0.811	0.069

For peer review only - http://bmjopen.bmj.com/site/about/guidelines.xhtml

Network meta-analyses

As detailed above, analyses were performed in R v4.2.0[3] and utilised the *gemtc*[2], *data.table*[4], *igraph*[5], *tidyverse*[6], *magrittr*[7], *and ggplot2*[8] R packages. Network meta-analyses were performed using a random-effects model, with 5 chains, a burn in period of 5000 iterations, a total of 100,000 iterations, and a thinning margin of 10. As the outcome of transfusion avoidance was analysed using mean differences, an identity link function was specified.

References

1. University of Sheffield - NICE Decision Support Unit. Full list of technical support documents (TSDs), 2025. Available from: https://www.sheffield.ac.uk/nice-dsu/tsds/full-list. Date accessed: May 25.
2. van Valkenhoef G, Kuiper J. gemtc: Network Meta-Analysis Using Bayesian Methods v1.0.2. 2023.
3. Computing TRFfS. R version 4.2.0. 2022.
4. Barrett T, Dowle M, Srinivasan A, Gorecki J, Chirico M, Hocking T, et al. data.table: Extension of `data.frame` v1.17.0. 2025.
5. Csardi G, Nepusz T. The igraph software package for complex network research v2.0.3. 2006.
6. Wickham H, Averick M, Bryan J, Chang W, McGowan LDA, François R, et al. Welcome to the Tidyverse. *Journal of Open Source Software* 2019; **4**: 1686.
7. Bache S, Wickham H. magrittr: A Forward-Pipe Operator for R v2.0.3. 2022.
8. Wickham H, Sievert C. *ggplot2: Elegant Graphics for Data Analysis*: Springer New York; 2009.
9. Gsteiger S, Howlett N, Ashlee B. gemtcPlus: Provides a suite of extension functions for NMA using the `gemtc` package v1.0.0. 2025.
10. National Institute for Health and Care Excellence (NICE). Crovalimab for treating paroxysmal nocturnal haemoglobinuria in people 12 years and over - Technology appraisal guidance [TA1019], 2024. Available from: https://www.nice.org.uk/guidance/ta1019. Date accessed: Apr 25.
11. Lee JW, Sicre de Fontbrune F, Wong Lee Lee L, Pessoa V, Gualandro S, Füreder W, et al. Ravulizumab (ALXN1210) vs eculizumab in adult patients with PNH naive to complement inhibitors: the 301 study. *Blood* 2019; **133**: 530-9.
12. Hillmen P, Young NS, Schubert J, Brodsky RA, Socié G, Muus P, et al. The complement inhibitor eculizumab in paroxysmal nocturnal hemoglobinuria. *The New England journal of medicine* 2006; **355**: 1233-43.
13. Röth A, He G, Tong H, Lin Z, Wang X, Chai-Adisaksopha C, et al. Phase 3 randomized COMMODORE 2 trial: Crovalimab versus eculizumab in patients with paroxysmal nocturnal hemoglobinuria naive to complement inhibition. *Am J Hematol* 2024; **99**: 1768-77.
14. Kulasekararaj AG, Hill A, Rottinghaus ST, Langemeijer S, Wells R, Gonzalez-Fernandez FA, et al. Ravulizumab (ALXN1210) vs eculizumab in C5-inhibitor-experienced adult patients with PNH: the 302 study. *Blood* 2019; **133**: 540-9.
15. Scheinberg P, Clé DV, Kim JS, Nur E, Yenerel MN, Barcellini W, et al. Phase 3 randomized COMMODORE 1 trial: Crovalimab versus eculizumab in complement inhibitor-experienced patients with paroxysmal nocturnal hemoglobinuria. *Am J Hematol* 2024; **99**: 1757-67.

For peer review only

BMJ Open

Clinical similarity in cost-comparison evaluations: a systematic review of current methods in NICE appraisals and the development of a framework for the formal assessment of clinical similarity

Journal:	*BMJ Open*
Manuscript ID	bmjopen-2025-112164.R1
Article Type:	Original research
Date Submitted by the Author:	26-May-2026

Complete List of Authors:	Edwards, Steve; BMJ Technology Assessment Group, BMJ-TAG, BMJ Group<br>Burgess, Benjamin; BMJ Technology Assessment Group, BMJ-TAG, BMJ Group<br>Downes, Nicole; BMJ Technology Assessment Group, BMJ-TAG, BMJ Group<br>Ip, Sophie; BMJ Technology Assessment Group, BMJ-TAG, BMJ Group<br>Walters, Archie; BMJ Technology Assessment Group, BMJ-TAG, BMJ Group<br>Dadswell, Clare; BMJ Technology Assessment Group, BMJ-TAG, BMJ Group

**Primary Subject Heading**:	Research methods
Secondary Subject Heading:	Evidence based practice, Health policy
Keywords:	Systematic Review, QUALITATIVE RESEARCH, STATISTICS & RESEARCH METHODS, Network Meta-Analysis

Note: The following files were submitted by the author for peer review, but cannot be converted to PDF. You must view these files (e.g. movies) online.

Online Supplementary File 4_Publication_Analysis_Code v1.1.R
Online Supplementary File 5_Publication_Analysis_Data.RData

SCHOLARONE MANUSCRIPTS

I, the Submitting Author has the right to grant and does grant on behalf of all authors of the Work (as defined in the below author licence), an exclusive licence and/or a non-exclusive licence for contributions from authors who are: i) UK Crown employees; ii) where BMJ has agreed a CC-BY licence shall apply, and/or iii) in accordance with the terms applicable for US Federal Government officers or employees acting as part of their official duties; on a worldwide, perpetual, irrevocable, royalty-free basis to BMJ Publishing Group Ltd ("BMJ") its licensees and where the relevant Journal is co-owned by BMJ to the co-owners of the Journal, to publish the Work in this journal and any other BMJ products and to exploit all rights, as set out in our licence.

The Submitting Author accepts and understands that any supply made under these terms is made by BMJ to the Submitting Author unless you are acting as an employee on behalf of your employer or a postgraduate student of an affiliated institution which is paying any applicable article publishing charge ("APC") for Open Access articles. Where the Submitting Author wishes to make the Work available on an Open Access basis (and intends to pay the relevant APC), the terms of reuse of such Open Access shall be governed by a Creative Commons licence – details of these licences and which Creative Commons licence will apply to this Work are set out in our licence referred to above.

Other than as permitted in any relevant BMJ Author's Self Archiving Policies, I confirm this Work has not been accepted for publication elsewhere, is not being considered for publication elsewhere and does not duplicate material already published. I confirm all authors consent to publication of this Work and authorise the granting of this licence.

For peer review only

Article Type

Original research

Title

Clinical similarity in cost-comparison evaluations: a systematic review of current methods in NICE appraisals and the development of a framework for the formal assessment of clinical similarity

Steven J. Edwards[1], Benjamin J. Burgess[1], Nicole Downes[1], Sophie Ip[1], Archie Walters[1], Clare Dadswell[1]

Correspondence to Dr Steven J. Edwards; sedwards@bmj.com

[1]BMJ Technology Assessment Group (BMJ-TAG), BMJ Group, London, UK.

Abstract

Objectives

To review how statistically non-significant indirect treatment comparison (ITC) results are interpreted within National Institute for Health and Care Excellence (NICE) cost-comparison evaluations (CCEs) and develop a framework to support interpretations of these results from Bayesian network meta-analyses (NMAs).

Design

A systematic review of CCEs between 2017 (first introduced) and April 2025. A framework (point-and-density plots) was developed to better interpret statistically non-significant NMA results for CCEs.

Data sources

For peer review only

CCEs were identified through NICE website searches, references of similar reviews, and communications with NICE.

Eligibility criteria

NICE technology appraisals (from 2017) that followed a CCE approach *ab initio*, had final guidance available and used non-statistically significant ITC results were included.

Data extraction and synthesis

A single reviewer performed screening and data extraction, with validation by a second reviewer. Narrative syntheses were performed separately for company, External Assessment Group (EAG) and committee perspectives. Point-and-density plots combine elements of forest plots and density plots alongside reporting the probability that a treatment is non-inferior relative to a comparator. These were applied to a recent CCE (TA1019) for crovalimab for patients with Paroxysmal Nocturnal Haemoglobinuria.

Results

Among 41 CCEs, EAGs raised concerns about statistically non-significant ITC results while companies relied heavily on them. Only ~32% of CCEs applied formal methods to explore ITC result uncertainty.

For the example framework analysis, comparisons of crovalimab to eculizumab (mean difference [MD]: 0.018; 95% confidence intervals [CIs]: -0.22 to 0.25) and ravulizumab (MD: 0.079; 95% CIs: -0.25 to 0.41) were statistically non-significant, with non-inferiority not demonstrated. However, point-and-density plots indicated a 95.9% and 86.3% probability of non-inferiority of crovalimab versus eculizumab and ravulizumab.

Conclusions

For peer review only

Interpretations of statistically non-significant ITC results are inconsistent within individual CCEs and across appraisals. Implementation of the presented recommendations and framework would improve the consistency and robustness of CCEs.

PROSPERO registration number

CRD420251034143.

Article Summary

Strengths and limitations of this study

The SLR provides insight into company, EAG and committee perspectives on evidence used to support conclusions of clinical similarity within NICE CCEs, with a focus on CCEs associated with the greatest amount of uncertainty.

The inclusion of appraisals that did not use a CCE approach from the start, but introduced these later, may have provided additional insights not currently captured.

The framework details how non-inferiority may be clearly, and robustly, assessed in NICE CCEs, addressing a known limitation with this pathway.

Implementation of the framework becomes more challenging when non-inferiority margins (or other thresholds) are not available.

A series of recommendations for NICE CCEs, where the results of ITCs are statistically non-significant are provided that, if fully implemented, would substantially improve the robustness and clarity of assessments of clinical similarity within this pathway.

For peer review only

INTRODUCTION

Background

NICE cost-comparison evaluation process

Technology appraisals, such as those performed in England as part of the National Institute for Health and Care Excellence (NICE) technology appraisal programme, involve the assessment of the clinical and cost-effectiveness of specific drugs, technologies or other interventions. As part of the NICE technology appraisal process, independent academic groups, called External Assessment Groups (EAGs), critique company submissions on behalf of NICE. This critique is considered alongside the company submission in the NICE committees' decision-making process. Complex health economic models are often required to assess whether the intervention of interest is likely to be a good use of National Health Service (NHS) resources, with decision-making informed by whether incremental cost-effectiveness ratios (ICERs) resulting from these models are within the range considered an acceptable and effective use of NHS resources and other considerations.[1] However, since 2017, cost-comparison evaluations (CCEs) have been accepted within the NICE technology appraisal programme; CCEs may be considered suitable where a new intervention is "likely to provide similar or greater health benefits at similar or lower cost than the relevant comparator(s)",[1-5] with relevant comparators considered to be, "those recommended in published NICE guidance for the same population".[1] CCEs involve a simpler comparison of costs and resources used between interventions, without the need for a complex health economic model such as those used in cost-utility analyses, but they require a strong assumption that the interventions being compared have similar clinical effectiveness and safety.[1, 3]

For peer review only

CCEs were first introduced as one of three routes under the Fast-Track Appraisal (FTA) process in 2017. In 2022, other routes via the FTA process, including where a base case ICER was <£10,000 per quality-adjusted life year (QALY) or where the most plausible ICER was <£20,000 per QALY and highly unlikely to be <£30,000, were removed and the new process focused solely on CCEs.[1-4, 6, 7] CCEs are an alternative to the standard NICE Single Technology Appraisal (STA) where a single new intervention is being assessed. They would not typically be used as an alternative to other appraisal types, such as Multiple Technology Appraisals (MTAs). The aim of CCEs is to provide an assessment of clinical and cost-effectiveness for suitable interventions that can be used for decision-making but in a shorter time frame and using fewer resources compared to the STA process, as part of NICE's commitment to taking a proportionate approach to technology appraisals.[8]

Challenges within cost-comparison evaluations

While the CCE process aims to improve the efficiency of the NICE technology appraisal process for appraisals that are considered to be a lower risk in terms of cost-effectiveness and whether they are an effective and acceptable use of NHS resources, making conclusions regarding whether there is sufficient evidence to conclude that a new treatment has "similar" efficacy and safety to a NICE-recommended comparator treatment can be challenging.

Decisions about clinical similarity are often more straightforward within CCEs that include direct evidence from randomised controlled trials (RCTs) for the new treatment against at least one of the NICE-recommended comparators; evidence from non-inferiority, equivalence and superiority RCTs can provide robust evidence to support conclusions of clinical similarity or that the new treatment is considerably better than a relevant comparator treatment.[9] However, NICE CCEs often do not have any direct comparative evidence for any relevant comparators and instead rely on the results of indirect treatment comparisons

For peer review only

(ITCs) such as network meta-analyses (NMAs), matching-adjusted indirect comparisons (MAICs), simulated treatment comparisons (STCs) or simple Bucher ITCs.[4, 5, 10]

Conclusions about the clinical similarity of treatments based on ITC results can be challenging given these analyses often generate results that are not statistically significant and there is not currently any guidance from NICE on how such results should be interpreted in order to prove clinical similarity. While non-significant differences indicate that there is insufficient evidence to conclude that there are statistically significant differences between treatments, it does not prove similarity, non-inferiority or equivalence. Therefore, a lack of statistically significant differences alone should not be used as conclusive evidence that two treatments are similar in terms of clinical outcomes.[11] Furthermore, it is possible that a difference in efficacy between two treatments may be clinically important even where the results are statistically non-significant.[12] Therefore, the interpretation of results from ITCs that indicate no statistically significant differences between treatments is challenging in terms of whether the treatments can be considered clinically similar.

As traditional statistical significance testing is not recommended for assessing clinical similarity, non-inferiority testing is of particular importance for CCEs, with such tests seeking to establish whether a new treatment is 'not unacceptably' worse than a comparator. For non-inferiority testing, an alternative threshold is specified, which is termed a non-inferiority margin (NIM) and represents the maximum reduction in clinical effectiveness that is considered acceptable while still considering the treatments to be equal.[9, 13-15] A pictorial comparison of traditional significance and non-inferiority testing is provided in Figure 1.

Existing guidance and research

The NICE Health Technology Evaluations Manual does not provide any guidance on how clinical similarity can be concluded in situations where ITC results are not statistically

significant and it only provides broad guidance on the type of evidence that can be considered or that is preferred; a preference for non-inferiority or equivalence RCTs with appropriate non-inferiority margins and including relevant comparators is expressed where possible, with meta-analysis and ITCs mentioned as alternatives if these sources of data are not available.[1] It also mentions that sufficient certainty is required in conclusions of clinical similarity to appropriate comparators in order for committees to recommend a technology, although no further detail on what constitutes uncertainty is described.[1]

There also appears to be a lack of guidance on this elsewhere; while some discussion of clinical equivalence and non-inferiority is available in a NICE Decision Support Unit document produced by York Health Economics Consortium (YHEC),[16] this is limited to the interpretation of results from equivalence and non-inferiority trials and is not applied specifically to ITCs in this document. Furthermore, on review of all Technical Support Documents developed by the NICE Decision Support Unit, no guidance to support with interpretation of non-significant results from ITCs was identified.[17] This lack of guidance exacerbates the challenges associated with concluding whether treatments can be considered to have similar clinical outcomes based on statistically non-significant ITC results given there is a risk of inconsistent decision-making.

Aims of this research

This research was commissioned by the National Institute for Health and Care Research (NIHR) Evidence Synthesis Programme (NIHR 176061). The project involved a systematic literature review (SLR) stage and a subsequent stage to develop a Bayesian framework to aid decision making.

The purpose of the SLR was to review and summarise the basis for decision-makers assuming clinical similarity based on statistically non-significant results obtained from ITCs within NICE CCEs, such as how often minimal clinically important difference (MCIDs) or

For peer review only

other approaches are used for interpretation and decision-making. The aim was to gain insight into whether inconsistencies exist in current decisions or whether thresholds for clinical similarity have emerged organically from decision-makers.

After this, the project aimed to develop a Bayesian framework to support the interpretation of statistically non-significant results from Bayesian NMAs, with the framework able to integrate MCIDs or other thresholds where available (including any pragmatic thresholds considered appropriate by committees during their deliberations in the absence of established thresholds). Ideally, the goal was for the proposed framework to allow for consistent, robust, and reproducible decision-making for future CCEs.

METHODS

Systematic Literature Review

The protocol for the SLR was registered on PROSPERO (CRD420251034143; see Online Supplementary File 1); there were no major deviations from the protocol herein.[18] The SLR was reported in accordance with Preferred Reporting Items for Systematic Reviews and Meta-Analyses (PRISMA). For the SLR, searches of the main NICE website using “cost comparison”, “cost minimisation” and “cost minimization” terms (including quotation marks) were performed by a single reviewer in April 2025.[19] Within the “Guidance programme” filter, the “technology appraisal guidance” option was selected to refine results. Results from each search were added to a Microsoft® Excel spreadsheet and duplicate records were removed before screening. In addition, NICE were contacted regarding ongoing CCEs that might be published before completion of the SLR analysis and similar reviews of NICE CCEs were reviewed to identify any additional appraisals not identified through the searches.[2, 3, 20, 21]

For peer review only

197 The SLR aimed to identify and include all NICE technology appraisals from 2017 onwards
198 that followed the CCE approach from the outset, relied on results from ITCs that were not
199 statistically significant for at least one comparator for the primary outcome(s) and had
200 published final guidance before completion of the SLR. A date of 2017 onwards was applied
201 given this is the year that CCEs were first introduced as an option by NICE.[22] Further details
202 regarding the inclusion and exclusion process, including pragmatic approaches that were
203 required, are provided in Online Supplementary File 2. All NICE appraisals identified from
204 the searches were screened against these criteria by a single reviewer. As validation, a
205 second reviewer reviewed all decisions to determine whether they agreed or disagreed with
206 the decision to include or exclude each appraisal. A third reviewer was available to resolve
207 any disagreements. Given the time constraints of this review, the protocol outlined that the
208 SLR would focus on the interpretation of any ITC results for primary outcomes used for
209 decision-making within the CCE, given ITCs can be performed for numerous outcomes.
210 Where no explicit mention of which outcomes were considered primary outcomes for
211 decision-making in a specific CCE was made, outcomes that were defined as primary
212 outcomes in the key clinical trials providing evidence for that appraisal, or that were noted as
213 being key components of economic models of earlier NICE technology appraisals in the
214 same indication, were considered to be the primary outcomes. Primary outcomes were not
215 prespecified, given they were to be specific to each included appraisal, dependent on the
216 disease area.
217
218 A single reviewer performed data extraction for each CCE using a standardised data
219 extraction form in Microsoft® Word, with a second reviewer validating a representative 30%
220 sample of extractions. Key information extracted included project name and ID, disease
221 area, primary outcomes used for decision-making, comparators requiring ITCs, type of ITC
222 performed, results of committee-preferred ITCs and whether or not the treatment was
223 recommended as a result of the CCE. Details on the methods used to support decision-
224 making and the rationale for these were also extracted, and any comments made by either

For peer review only

the company, EAG or committee relating to the ITC results and interpretation of clinical similarity were extracted from the documents in full for use in narrative syntheses. Further information on the data extraction process is outlined in Online Supplementary File 2.

As the SLR includes NICE CCEs, rather than clinical trials for which validated quality assessment tools exist, a risk of bias assessment was not performed within this SLR. A narrative synthesis of results was performed separately for company, EAG and committee perspectives, and an overall comparison between these groups was also considered. Tables were used to record new methods or themes identified from the extracted data and to collate all CCEs that mentioned each method or theme. Summary tables outlining the key findings have been presented alongside a narrative discussion of findings.

Statistical Framework

The aim of the new framework is to present the results of non-inferiority tests to allow NICE committees to make informed decisions regarding the clinical similarity of treatments, with a particular emphasis on CCEs. Importantly, the framework that has been developed can be viewed as an 'add-on' to existing methods for performing Bayesian NMAs. Although formal non-inferiority testing should remain the primary focus of any assessments of clinical similarity, it is anticipated that this new framework will be of particular use where there is no evidence of a significant difference between a treatment and a comparator.

The statistical framework uses empirical cumulative density functions (ECDFs) to determine the probability that an effect size falls below a given threshold (e.g., NIM). Accordingly, by combining the results of ECDFs with those of traditional density and forest plots, it is possible to convey all three separate analyses within a single readily interpretable figure, which addresses the lack of a specific visualisation for non-inferiority analyses. An example of this figure, termed a point-and-density plot, is shown in Figure 2. In this example, the 95%

For peer review only

credible intervals (CrIs) overlap both 0 and the NIM; as such, there is no evidence for a statistically significant difference between the treatment and comparator, while the non-inferiority of the treatment, relative to the comparator, is not demonstrated. However, there is a 95.86% probability that the treatment was non-inferior to the comparator. It is suggested that committees initially focus on whether the 95% CrIs overlap the NIM to determine whether a treatment is non-inferior. However, the use of probabilities of non-inferiority may provide valuable information, particularly where non-inferiority has not been demonstrated.

For some analyses, a NIM (or other threshold) may not be available. In such a situation, it is recommended that the corresponding threshold is set to either 0 or 1 (depending on the considered effect size), given that this represents a conservative threshold by which to assess non-inferiority. By using ECDFs, it is possible to determine a hypothetical, non-clinically validated, threshold. Such a hypothetical threshold can be used to determine the required threshold for a given, arbitrary, probability that a treatment is non-inferior to a comparator. It is suggested that an arbitrary 95% probability of non-inferiority may be selected for a hypothetical threshold. In doing so, a 95% probability of non-inferiority represents a “high bar” for any assessments of non-inferiority in the absence of a clinically validated NIM.

Full details of the statistical framework are provided in Online Supplementary File 3, with Supplementary Figures 2 to 5 providing a visual representation of the processes applied within the framework. Furthermore, R code (and associated data) to generate point-and-density figures for the below case study are provided as Online Supplementary Files 4 and 5.

Case study

To showcase how this framework may be used within CCEs, a case study is presented where the framework has been applied to data presented in a recent CCE. NICE TA1019

For peer review only

assessed crovalimab for the treatment of paroxysmal nocturnal haemoglobinuria (PNH) in people 12 years and over,[23] with crovalimab subsequently being approved for use by NICE in line with its marketing authorisation. TA1019 compared crovalimab to the two treatments currently used to treat PNH, namely eculizumab and ravulizumab. Within the final draft guidance, NICE stated that the clinical trial evidence indicated that crovalimab works as well as eculizumab and is likely to be as effective as ravulizumab.[24]

Within TA1019, the company presented Bayesian NMAs for the primary co-endpoint from the COMMODORE 1 and 2 trials of transfusion avoidance (but not the primary co-endpoint of haemolysis control),[25, 26] which was assessed using the endpoint of mean difference.[23] Furthermore, the company stated that a NIM of 0.20 was considered based on data from a previous clinical trial.[23] The company provided the results for random-effects (RE) Bayesian NMAs for the overall population, patients who had previously received complement inhibitors (experienced subgroup), and patients who had not previously received complement inhibitors (naive subgroup).[23]

For the case study, the Bayesian RE NMA for the overall population was re-analysed using data provided in the company's submission for TA1019. As such, the results of the company's submission are able to be compared with those from the re-analysis. Within the company's submission, the company stated that meaningfully informative priors were used to estimate between-study heterogeneity; however, the company did not provide these informative priors in the submission.[23] As such, any differences in the results of the original analysis and the equivalent analysis presented here are likely to be attributed, at least in part, to a difference in priors with uninformative priors used in the re-analysis.

Patient and public involvement

There was no patient or public involvement in this research.

For peer review only

RESULTS

Systematic Literature Review

Search and screening results

A total of 91 individual records of NICE technology appraisals were identified and screened for inclusion in this SLR, with 41 appraisals ultimately included. A full PRISMA diagram is presented in Supplementary Figure 1. A range of disease areas were covered by the included appraisals. Multiple CCEs included in this SLR had been performed for certain conditions, such as plaque psoriasis and ulcerative colitis, while other conditions were only represented in one of the included CCEs (see Supplementary Table 1). In terms of ITCs, over 60% relied solely on NMAs, with smaller percentages using other ITC methods. While all appraisals relied on ITC evidence for at least one comparator, ~25% had direct trial evidence for another comparator relevant to the appraisal. Details of all included appraisals and a list of excluded appraisals are provided in Supplementary Tables 1 and 2, respectively.

Themes identified

Narrative syntheses of company, EAG and committee approaches to the interpretation of statistically non-significant ITC results and additional evidence sources were performed. Supplementary Tables 3 to 5 include an overview of the different methods used by companies, EAGs and committees to interpret the ITC results as well as additional methods used to support conclusions about clinical similarity, such as use of clinical expert feedback or reference to real-world data. An overall synthesis of these findings across these three groups is discussed narratively below, comparing and contrasting the approaches used across companies, EAGs and committees, with summaries relating to the interpretation of

For peer review only

ITC results and use of supplementary evidence across these groups presented in Tables 1 and 2, respectively.

The key finding was a lack of consistency in terms of methods used to interpret statistically non-significant ITC results and whether these were considered sufficient evidence of clinical similarity. While inconsistency existed across appraisals, EAGs tended to raise concerns about relying solely on these results as evidence of clinical similarity and companies usually placed a strong emphasis on the lack of statistically significant differences. EAGs sometimes concluded that there was too much uncertainty within ITCs for a CCE to be appropriate, while others considered supplementary information such as similar mechanisms of action, clinical expert feedback, previously published ITC results or NICE appraisals and/or naive comparative data to alleviate these concerns.

More formal methods of exploring uncertainty were sought by some EAGs and/or companies, including the application of MCIDs or NIMs and/or presentation of probabilities based on Surface Under the Cumulative RAnking curves (SUCRAs) from NMAs. However, one or more of these approaches were only explored in a minority (~32.0%) of appraisals. Of note, no insight into committee preferences regarding which methods are most useful in terms of interpreting these results was identified from the appraisals, but committees frequently made use of clinical expert feedback and information on mechanisms of action where uncertainty was noted. To better illustrate these differences, the approaches used and conclusions made by companies, EAGs and committees in two of the included CCEs are summarised in Table 3. These examples show how companies often relied on the lack of statistically significant differences and that EAGs often looked to expand on this by using additional information to inform their conclusions about clinical similarity. These examples also show the differences between EAGs in terms of non-statistically significant differences; one strongly dismisses their use in judging clinical similarity, while another does not dismiss them but uses other data to add to these observations. Furthermore, one EAG used clinically

For peer review only

meaningful thresholds to facilitate interpretation of the ITC results, sought via clinical expert feedback, while another referred to other sources of data to support conclusions from ITCs. The researchers consider that these differences in approach are primarily due to the lack of guidance for companies and EAGs on how to approach the interpretation of non-statistically significant ITC results, including suitable additional methods that may improve decision-making, within NICE CCEs.

Two more general themes relating to CCEs were also identified, including improving the transparency of the decision-making process within committee discussions and clarifying whether only one relevant comparator outlined in the NICE final scope needs to be covered. These are summarised in Supplementary Table 6; they are not addressed by the framework described in this publication but were considered to be useful findings that may be addressed through updated guidance or reporting of committee discussions by NICE.

For peer review only

Table 1. Overview of methods used to interpret statistically non-significant ITC results

Method	Company	EAG	Committee
No statistically significant differences	Some relied solely on a lack of statistically significant differences but most also discussed the direction/size of the point estimate. Companies generally did not acknowledge the uncertainty of statistically non-significant ITC results.	EAGs often considered statistically non-significant differences with or without consideration of point estimate size/direction to be insufficient. Limitations associated with statistically non-significant results from ITCs were often noted.	No clear position on whether statistically non-significant results from ITCs were sufficient for decision-making was identified.
Point estimate direction and/or size	Companies often heavily relied on point estimate direction/size. Some suggested that point estimates favouring the intervention for some analyses and the comparator for others was evidence of no consistent treatment difference. Some also suggested that clinical similarity was a conservative assumption where point estimates favoured the intervention.	Limitations of non-significant differences from ITCs were often highlighted, often noting that the inferiority of the new treatment could not be ruled out. However, many did mention them as supportive evidence and were reassured by review of other evidence or the robustness of the results to alternative analyses.	No clear position on the interpretation of point estimate direction and/or size was identified in most appraisals but point estimates close to the line of null effect in one appraisal reduced the committees concerns about uncertainty raised by the EAG.
Some statistically significant benefits of the new intervention	In some appraisals, statistically significant differences were identified from ITCs for some but not all analyses. Companies tended to consider this evidence that clinical similarity was conservative.	No strong EAG conclusions regarding these were noted. Some statistically significant benefits may have increased EAG confidence of clinical similarity, but concerns may have remained.	No explicit opinion from committees on this was identified within this SLR, but it may have provided some reassurance.
Probabilities calculated from NMAs	Probabilities from NMAs were mentioned in ~15% of company submissions. However, not all used them to support their conclusions. Most involved SUCRA information from NMAs but two combined this with a NIM or clinical equivalence threshold, either from the outset or following a request from the EAG.	EAGs requested these methods from companies, suggested adaptations to company approaches or noted that they would have reduced uncertainty in ~17% of appraisals. Simpler probability ranks and calculating probabilities of falling within a clinical equivalence range were noted as options.	No explicit opinion from committees on the application of this method for interpreting ITC results, and whether it would increase committee confidence for decision-making, was identified within this SLR.
MCIDs or other thresholds	Companies in ~10% appraisals incorporated MCIDs or NIMs. Thresholds were used either to assess whether point estimates represented a meaningful difference, or incorporated into an NMA to calculate probabilities of clinical equivalence.	EAGs (~17% of appraisals) used or adapted company threshold-based approaches or indicated that they would have been useful. Application of thresholds to assess whether ITC results were clinically meaningful or within NMAs to calculate probabilities were mentioned.	No explicit opinion on the application of this method for interpreting ITC results, and whether it would increase committee confidence for decision-making, was identified within this SLR.

Abbreviations: EAG, External Assessment Group; ITC, indirect treatment comparison; MCID, minimal clinically important difference; NIM, non-inferiority margin; NMA, network meta-analyses; SLR, systematic literature review; SUCRA, Surface Under the Cumulative RAnking curve.

For peer review only

Table 2. Overview of sources of supplementary evidence used to support conclusions of clinical similarity

Method	Company	EAG	Committee
Similar mechanisms of action	Similar mechanisms of action were mentioned in ~15% of appraisals as supportive evidence that outcomes of the treatments were likely to be similar.	While not always explicitly used as supportive evidence for conclusions, ~34% of EAGs commented on the similarity of mechanisms of action between the intervention and comparators.	Committee discussions often mentioned whether mechanisms of action are similar between treatments, and in ~7% of appraisals, this may have reduced uncertainty and enabled conclusions of clinical similarity.
Clinical expert feedback	Clinical expert feedback was mentioned by companies in ~24% of appraisals, either based on clinician experience with the treatments or anticipated differences, or clinician review of ITC results to determine whether differences were clinically meaningful or not.	EAGs mentioned clinical expert feedback in ~34% of appraisals, including expert feedback that there are no differences between treatments based on their experience, that no differences were anticipated based on their knowledge of the treatments and/or support with interpretation of results, such as thresholds for clinically meaningful differences.	Committee discussions did not routinely include statements about clinical expert opinion. However, where EAGs raised uncertainty with ITC results, this was cited as a reason that the committee was ultimately able to conclude clinical similarity in ~17% of all included appraisals.
Evidence of a class effect from meta-analyses	Some companies highlighted previously published meta-analyses of the intervention and comparators against a common comparator (e.g. placebo) as supportive evidence of a class effect by some companies.	In two cases where the company suggested this, it was rejected by the EAG as a robust source of evidence, although it appeared to be of some use to the EAG in the third appraisal.	This was mentioned in the committee discussion of one of these appraisals, but there was no indication of whether it was considered useful to the committee.
Previous ITC results or NICE appraisals	Existing ITCs and/or NICE appraisals were cited in four appraisals, with results said to be similar to the current appraisal or decisions made based on a similar level of uncertainty to a previous appraisal.	These were mentioned by EAGs in a similar number of appraisals, including references to similar results being obtained or a similar level of uncertainty within ITC results that had not precluded a conclusion of clinical similarity.	These were generally not mentioned in documents summarising committee discussions.
Real-world data	One company had SACT data available, which was used as supportive evidence alongside ITC results.	The EAG also made use of these data when making its conclusions.	These data were not mentioned in the committee discussion of the evidence.
Other EAG sources	NA	Naïve comparative evidence (for example, a visual comparison of KM curves from each study or statistical testing of differences) was used by some EAGs.	This was mentioned in some committee discussions, suggesting it was of some use, but was not mentioned in others.

Abbreviations: EAG, External Assessment Group; ITC, indirect treatment comparison; KM, Kaplan-Meier; NICE, National Institute for Health and Care Excellence; SACT, Systemic Anti-Cancer Therapy.

For peer review only

370 Table 3. Comparison of methods used across two CCE examples

Study information	Acalabrutinib for treating chronic lymphocytic leukaemia (TA689)[27]	Zanubrutinib for treating chronic lymphocytic leukaemia (TA931)[28]
Primary outcome(s)	• OS<br>• PFS	• OS<br>• PFS
Company approach	• Focus on lack of statistically significant differences and trend towards point estimates favouring the intervention as evidence of clinical similarity<br>• Similar mechanisms of action and clinical expert feedback also noted as supplementary evidence	• Focus on lack of statistically significant differences and some point estimates favouring the intervention as evidence of clinical similarity<br>• Supplementary discussion of clinical expert feedback that treatments likely similar
EAG approach	• Does not reject the company's conclusions relating to statistically significant differences outright<br>• Also considered similarity of adjusted KM curves, clinical expert feedback, the limitations of the ITCs performed in terms of power available to detect statistically significant differences and variability across sensitivity analyses<br>• Concluded that clinical similarity was a reasonable conclusion for one of the two included populations	• Dismisses lack of statistically significant differences and point estimates favouring the intervention as evidence of clinical similarity<br>• Notes uncertainty and does not agree with CCE approach<br>• Incorporates clinically meaningful differences based on clinical expert feedback into its interpretation of the results (upper CIs crossed these)
Committee considerations	• Acknowledges uncertainties relating to the conclusions but concludes that clinical similarity is a reasonable conclusion<br>• Considered clinical expert feedback alongside ITC results	• CCE approach was rejected given too much uncertainty in the ITC results

Abbreviations: CCE, cost-comparison evaluation; CI, confidence interval; EAG, External Assessment Group; ITC, indirect treatment comparison; KM, Kaplan-Meier; OS, overall survival; PFS, progression-free survival; TA, technology appraisal.

For peer review only

Statistical Framework

A recent CCE, TA1019,[23] assessing crovalimab in patients with PNH, was used as a case study to illustrate the described framework. The SLR performed by the company for TA1019 identified five trials reporting data on transfusion avoidance in people with PNH, which formed a star-shaped network (Supplementary Figure 6). Two trials compared ravulizumab to eculizumab, two trials compared crovalimab to eculizumab, and one trial compared eculizumab to standard of care, with data for each of the included trials provided in Supplementary Table 7.

The results of the RE NMAs from TA1019 and the case study re-analysis are presented in Figure 3. Within the CS,[23] the company noted that the results of this NMA indicated that there was no statistically significant evidence for a difference between crovalimab and both eculizumab (MD: 0.017; 95% CrIs: -0.11 to 0.15) and ravulizumab (MD: 0.077; 95% CrIs: -0.09 to 0.24) as the 95% CrIs overlapped 0 for both comparisons. However, the 95% CrIs for the comparison of crovalimab to ravulizumab overlapped the NIM of 0.20 (i.e., 20%), while the 95% CrIs for the comparison of crovalimab to eculizumab did not. Accordingly, crovalimab would be considered to be non-inferior to eculizumab but non-inferiority was not demonstrated compared to ravulizumab, although this was not explicitly stated in the company's submission. However, the company stated in text, that there was a 99% and 93% probability of crovalimab being non-inferior to eculizumab and ravulizumab, respectively, when considering the NIM of 0.20.[23] For the case study reanalysis, the point estimates for each of the comparisons of crovalimab to eculizumab (MD: 0.018; 95% CrIs: -0.22 to 0.25) and ravulizumab (MD: 0.079; 95% CrIs: -0.25 to 0.41) align with those from the analysis in the company's submission. However, the 95% CrIs intervals associated with each point are much wider than those in the original analysis. As previously noted, this is expected to be due to the use of informative priors in the company's original analysis.

For peer review only

398 Results of the RE NMAs from the case study reanalysis were additionally analysed using the new statistical framework with point-and-density plots shown for the comparisons of crovalimab to eculizumab and crovalimab to ravulizumab (Figure 4). As shown by the point-and-density plots, there was a relatively high probability of crovalimab being non-inferior compared to both ravulizumab (86.27%) and eculizumab (95.87%). Additionally, through the use of ECDFs it was possible to determine that a hypothetical threshold of 0.31 would be required in order for there to be a 95% probability that the point estimate, for the comparison of crovalimab to ravulizumab, was below this hypothetical threshold.

406 Within the re-analysis, the non-inferiority of crovalimab relative to both eculizumab and ravulizumab was not statistically demonstrated. The point-and-density figures illustrated that there was a high probability that the difference in treatment effects would be considered non-inferior for comparisons of crovalimab to both eculizumab and ravulizumab. Accordingly, the results of the re-analysis for the overall population support the use of a cost-comparison for TA1019.

DISCUSSION

Systematic Literature Review

414 The key finding from the SLR was inconsistency with regard to how statistically non-significant differences from ITCs were interpreted within CCEs; companies frequently interpreted these as sufficient evidence for clinical similarity, while EAGs generally seem to be aware of the importance of not misinterpreting the absence of a statistically significant difference as evidence of clinical similarity. Methods emerging from the current appraisals to further explore these ITC results include specific tests of non-inferiority utilising a threshold such as a clinically derived NIM or MCID. However, only a minority of CCEs in this SLR made use of such methods. Furthermore, additional detail within committee discussions

422 would improve the transparency of decision-making in these appraisals, given it was often
423 limited, and clarity regarding how many comparators need to be covered from the NICE final
424 scope would be beneficial for companies and EAGs.

425 Findings from the SLR are largely in line with a recent publication by PenTAG, an EAG
426 working on NICE appraisals, which also raised concerns about relying on statistically non-
427 significant ITC results in CCEs and made recommendations on methods that may improve
428 transparency in decision-making.[21] However, another EAG recently concluded that a lack of
429 statistically significant differences or overlapping 95% confidence intervals may be sufficient
430 to demonstrate clinical similarity in CCEs.[29] Therefore, the current lack of guidance in this
431 area of NICE CCEs may also be contributing to differences in perspectives across EAGs,
432 and work on methods that can support the interpretation of statistically non-significant ITC
433 results may improve the consistency and transparency of decision-making within CCEs.
434

435 Strengths of this SLR include the focus on CCE appraisals relying on non-significant ITC
436 results; given there is more uncertainty associated with this type of evidence, this has
437 allowed the review to focus specifically on methods being used in situations where there is
438 the highest uncertainty in conclusions and the greatest need to identify methods to address
439 this. Furthermore, a thorough review of company, EAG and committee perspectives has
440 been performed, allowing the identification of similarities and inconsistencies in approaches
441 across appraisals and also between companies, EAGs and committees. A potential limitation
442 of this SLR is that keywords were used to search the NICE website rather than reviewing the
443 full list of NICE technology appraisals, but on review of appraisals included in the work by
444 PenTAG (which used the latter approach), the researchers are reassured that this approach
445 did not lead to many relevant appraisals being missed.[21] Furthermore, inclusion of appraisals
446 that did not use a CCE approach from the start but introduced them later in the process was
447 not possible due to limited resources; it is possible that these types of appraisals may have
448 provided useful additional information but the researchers consider it unlikely that the

For peer review only

findings of this SLR would change considerably if these appraisals had been considered. Finally, it was not possible within the time constraints of this research to perform searching, screening and data extraction completely in duplicate using two independent reviewers, which is a further limitation of the SLR in this research; however, approaches to validate each of these stages using input from a second reviewer were implemented to minimise the risk of any errors during these stages.

Statistical Framework

There is currently uncertainty, from pharmaceutical companies, regarding how NICE assesses equivalence, or non-inferiority, within NMAs and other forms of ITCs.[21] As such, the framework presented here provides a clear outline for how non-inferiority may be assessed within CCEs that utilise NMAs. Accordingly, a key strength of the newly developed framework is that it addresses a known challenge that NICE committees face when evaluating CCEs, namely, how to assess clinical similarity when the results of NMAs indicate that there is no evidence for a statistically significant difference. As shown by the results of the SLR, there are no methods or frameworks that are consistently applied in CCEs, that utilise NMAs, to assess clinical similarity.

Within this newly presented framework, specifically through the point-and-density plots, an approach has been provided that is readily interpretable and provides information that was previously unavailable, to allow NICE committees to draw robust conclusions as to whether a new treatment is non-inferior to an existing comparator. Crucially, when non-inferiority, or inferiority, has not been demonstrated in a direct comparison within a randomised controlled trial, the framework presents the probability that the new treatment is non-inferior to an existing comparator. As such, this allows NICE committees to make informed decisions on the likelihood of a new treatment being clinically similar to an existing comparator, even

For peer review only

when non-inferiority has not been statistically demonstrated. Additionally, the proposed framework has been implemented within an EAG's review of a recent CCE for resectable non-small-cell lung cancer.[30]

Furthermore, a recent review of CCEs and methodological approaches that are used to assess non-inferiority reported that visualisations to specifically assess non-inferiority were scarce and that where such visualisations were provided, the most common approach was to present forest plots with an overlaid NIM.[21] As such, NIMs may be viewed as being "shoehorned" into existing visualisations with little consideration of the most appropriate manner of displaying such information.[21] Accordingly, the new framework proposed here helps to overcome this limitation through the development of point-and-density plots that specifically seek to display the results of NIMs in a dedicated, intuitive manner.

As has been previously discussed, the majority of CCEs do not provide a NIM, MCID, or another threshold to allow for a statistically robust assessment of non-inferiority. As a consequence, clinical similarity has typically been erroneously assessed through inappropriate statistical approaches.[29] However, there is disagreement between EAGs regarding what constitutes an appropriate method to assess clinical similarity within CCEs.[21, 29]

Where a NIM or MCID is not available, the new framework presented here allows for a 'hypothetical threshold' to be calculated which refers to the required value of a NIM in order for an arbitrary probability (e.g., 95%) that a treatment is non-inferior to a comparator. Such an approach is a strength of the new framework and provides further context to EAGs and committees with regards to the likelihood of a new treatment being non-inferior, relative to an existing comparator, when such information would not otherwise be available. Although it is possible to calculate a 'hypothetical threshold' where a NIM, MCID, or other threshold has not been provided, it is strongly recommended that this approach should not be considered a

For peer review only

substitute for the provision of a clinically validated NIM or MCID. Indeed, it is recommended that a company should provide a clinically validated NIM or MCID for all primary, or co-primary, outcomes in order for an appraisal to be considered for the CCE pathway. The absence of a clinically validated NIM or MCID severely limits the ability of a committee, or EAG, to reach definitive conclusions as to whether a new treatment is non-inferior, or clinically similar, to a previously approved comparator. Accordingly, when a ‘hypothetical threshold’ is implemented is it imperative that is labelled as such, in order to prevent EAGs and committees from erroneously assuming that the selected threshold was clinically validated.

Within this framework, a 95% threshold for assessing non-inferiority has been recommended. The researchers acknowledge that this threshold represents a “high bar” for assessing non-inferiority. As such, if this framework is implemented in CCEs, committees and EAGs may wish to explore alternative thresholds for assessing non-inferiority (e.g., a 80% or 90% probability of being non-inferior), in doing so assessing how sensitive assessments of non-inferiority are to the choice of hypothetical threshold.

The proposed framework is only able to be implemented with Bayesian, and not frequentist, NMAs. For frequentist NMAs, fixed parameters represent population characteristics, therefore meaning that these analyses can only help researchers decide whether to accept, or reject, a hypothesis based on statistical significance.[31, 32] In contrast, Bayesian NMAs use a probability distribution of model parameters which, through Markov Chain Monte Carlo simulations, generate a posterior probability distribution.[32] Within the framework, the posterior probability is used to determine the probability of non-inferiority. Although studies have suggested that there are no important differences in the performance of these approaches, it has been reported that Bayesian NMAs are more frequently used compared to frequentist NMAs.[32, 33] Accordingly, given the predominance of Bayesian NMAs, and the availability of software to perform Bayesian analyses,[32] the sole applicability of the

For peer review only

framework to Bayesian NMAs is not considered to be a limitation of the approach. Alongside the choice of Bayesian or frequentist methods, NMAs are dependent on numerous factors that should considered *a priori* (e.g., the choice of a fixed-effect or random-effects model) based on clinical or statistical rationale and not selected to allow the presented framework to be applied. However, it should be noted that the presented framework can be used for both fixed-effect and random-effects Bayesian NMAs. Additionally, although the presented case study centred on the effect size of mean difference, the framework is applicable to multiple other effect sizes (e.g., odds ratios, hazard ratios, or standardised mean differences). Finally, we note that NMAs are the most commonly implemented approach for ITCs of fully connected networks. However, multi-level network meta-regression (ML-NMR) is a recent Bayesian ITC method that is becoming more widely adopted.[34] Accordingly, future research may seek to allow the presented framework to be applied to both Bayesian NMAs and ML-NMRs.

The presented framework has been recently implemented within an EAG's review of a recent CCE.[30] Although the framework addresses issues with how NICE assesses non-inferiority in CCEs, the adoption of this method by EAGs, NICE committees, and pharmaceutical companies, may take some time. Conversely, alternative, statistically rigorous, frameworks may be developed and subsequently implemented. Accordingly, it is suggested that a future update to the SLR is performed, hence allowing the uptake of the proposed framework to be comprehensively assessed.

Recommendations

A number of recommendations, that cover all aspects of the approach to assess clinical similarity within CCEs, resulting from both the SLR (Recommendations 1 to 6) and the framework (Recommendations 7 to 10) have been suggested. As such, NICE CCEs would

For peer review only

be methodologically, and clinically, strengthened if all of these recommendations were to be adopted.

1. Use of MCIDs or NIMs should be considered to support the interpretation of statistically non-significant results from ITCs. Where these are not available or cannot be estimated robustly, clinical expert advice could be sought to estimate pragmatic thresholds for the purpose of the appraisal;

2. The incorporation of thresholds (e.g., NIMs or MCIDs) into Bayesian analyses to obtain probabilities of the new treatment being no worse than one or more established comparators with existing NICE guidance would be a robust method of using such thresholds. When multiple comparators exist, presentation as pairwise comparisons against each comparator may be useful given that committees evaluate the evidence to support a CCE against each comparator separately. Where a suitable threshold has not been identified, even with clinical expert input, probabilities of being the best treatment within a Bayesian network or being better than each comparator may be useful for decision-making purposes. When a Bayesian analysis is not possible, other methods of using MCIDs or NIMs to support the interpretation of results should be considered;

3. Guidance related to the uncertainties of statistically non-significant ITC results within the NICE methods manual or user guides for submissions may be useful to improve the case put forward in company submissions and provide support for EAGs in terms of what information or methods might be reasonable to request. This may also improve consistency across EAGs in terms of how statistically non-significant ITC results are interpreted, given the different positions noted in recent publications by EAGs in this area.[21, 29] This guidance need not be prescriptive but could simply highlight the limitations of relying on statistically non-significant differences from ITCs

For peer review only

and that additional methods may be useful to support conclusions of clinical similarity;

4. Supportive information such as clinical expert feedback, comments on the similarity of mechanisms of action and consideration of naïve comparative evidence should not be considered sufficient to resolve uncertainty associated with statistically non-significant ITC results. Statistically robust approaches should be explored to improve confidence in conclusions of clinical similarity based on ITC results, with supportive information used to supplement these conclusions;

5. Additional detail regarding committees' interpretation of statistically non-significant ITC results within published NICE guidance for each appraisal may improve transparency within the decision-making process, particularly where there is discordance between overall conclusions made by the EAG and the committee;

6. Clarity on whether or not a CCE needs to make a case against multiple or only one relevant comparator within the NICE CCE process may be useful. Explanation of when a single comparator may be deemed appropriate within the NICE process and whether companies should still explore all or multiple comparators in their original submission might address this uncertainty;

7. CCEs should implement a consistent framework to support the interpretation of statistically non-significant results from NMAs. Through utilisation of point-and-density plots, committees and EAGs will be presented with results in a consistent format, while all stakeholders will have clarity regarding how clinical similarity is assessed;

8. When an established NIM or MCID is used, it is recommended that a probability of 95% is used to determine if a new treatment can be considered to be 'clinically similar' to a comparator;

For peer review only

9. When a NIM or MCID is unavailable, it is recommended that a value representing no difference is used to assess clinical similarity, given that this represents a conservative estimation of non-inferiority;

10. In exceptional circumstances, a committee may want to use an estimated hypothetical threshold that is required for a treatment to be considered "clinically similar" to a comparator. In doing so, this approach would allow the committee to qualitatively determine how different the estimated results are from this theoretical threshold.

CONCLUSIONS

NICE requires that CCEs demonstrate that new interventions are clinically similar to relevant comparators already approved by NICE. Here, the SLR sought to provide an overview of the current approaches that are used within CCEs with statistically non-significant ITC results to conclude clinical similarity. Furthermore, the newly developed Bayesian framework sought to provide EAGs and NICE committees with additional insights regarding clinical similarity. Overall, this research provides vital information that may result in better-informed decision-making when assessing CCEs.

The SLR determined that assessments of clinical similarity were not consistent in the identified CCEs. Companies frequently interpreted statistically non-significant differences as evidence for clinical similarity, while EAGs often correctly refuted these assertions. Importantly, an absence of a statistically significant difference between two treatments is not evidence of clinical similarity. Instead, clinical similarity should be assessed through specific tests of non-inferiority that utilise a clinically relevant threshold (e.g., NIMs or MCIDs). The SLR indicated that 75% of CCEs did not use such approaches. Furthermore, there are some uncertainties regarding how NICE committees determine whether a new treatment is

For peer review only

considered clinically similar to a comparator. For instance, it is unclear what methods NICE considers acceptable when relying on statistically non-significant differences from ITCs and whether clinical similarity is required to be demonstrated for an intervention relative to one, or all, relevant comparators.

The framework was developed to overcome several limitations of the current approach to assessing clinical similarity in the CCE pathway. Specifically, the framework represents a consistent and rigorous approach to assessing clinical similarity where the results of a Bayesian NMA are statistically non-significant. If this framework were to be widely implemented within CCEs, it would eliminate the current methodological inconsistencies in how clinical similarity is assessed, while also providing clarity to all stakeholders involved in the process. Accordingly, point-and-density plots are easily understandable through the combination of components from multiple commonly used figure types, and represent a stepwise improvement over previously used visualisations, given that point-and-density plots have been designed specifically for CCEs. Following the completion of the SLR and development of the framework, the researchers have made a number of recommendations which, if all were to be implemented, would enhance the robustness and transparency of the NICE CCE pathway.

Word Count: 6521 words

Data availability statement

Additional information on the methods and results of this research has been provided in the online supplementary files.

For peer review only

Funding statement

This research was commissioned by the National Institute for Health and Care Research (NIHR) Technology Assessment Reviews programme (NIHR 176061).

Acknowledgements

The authors thank members of the NICE team for responding to requests for information about completed and ongoing cost-comparison evaluations.

Competing interests

No competing interests were declared which affect the impartiality of this report. BMJ Technology Assessment Group (BMJ-TAG) and the editorial team of BMJ Group work independently to one another. The views and opinions expressed in this report are those of the BMJ-TAG.

Ethical statement

This research did not require ethical approval or patient consent for publication given it was a systematic review of NICE technology appraisals and the development of a statistical framework. It does not include information that could identify individuals and did not involve enrolment of human or animal subjects.

Author contributions

SE was responsible for the conception of the research project and provision of research framework, provided guidance and validation of the work of the research group at all stages including all versions of the protocol, report and publication. Guarantor of this manuscript.

For peer review only

BB led on the development of the Bayesian framework for a formal assessment of clinical similarity, tested the developed framework using examples identified through the SLR and drafted sections of the report and publication related to the Bayesian framework.

ND led the development of the protocol for the SLR phase of this research, was the lead reviewer on the SLR, including development of search strategy, running searches, sifting of results, data extraction and narrative synthesis of results, and drafted sections of the report and publication related to the SLR.

SI supported during the Bayesian framework development stage by validating code and analyses.

AW was the second reviewer on the SLR phase of this research and was involved in the validation of the screening stage and a proportion of the extractions.

CD contributed to the development of the project and was available to provide feedback to resolve any outstanding conflict between the first two reviewers on the SLR.

Figure captions

Figure 1. Illustration of traditional statistical significance testing (A) and non-inferiority testing (B).

Footnote: Points denote effect sizes, while lines denote 95% credible intervals. Dashed lines correspond to the thresholds of the null and alternative hypotheses. For traditional significance testing the dashed line corresponds to either 0 or 1 (depending on the given effect size). For non-inferiority testing the dashed line corresponds to the non-inferiority margin. Areas without shading correspond to the alternative hypothesis, areas with red shading correspond to the null hypothesis. Red diamonds denote effect sizes where the null hypothesis was accepted. Black circles denote effect sizes where the null hypothesis was rejected. Grey squares denote effect sizes where there is insufficient evidence to reject the null hypothesis.

Figure 2. Point-and-density plot for the comparison of an example treatment and comparator.

Footnote: The threshold for traditional hypothesis testing is shown by the solid line, while the dashed line indicates the NIM. The area that falls under (above) the NIM is shown in blue (orange). The probability that the effect size falls under, or above, the NIM is shown in the upper left corner of the plot. The pooled estimate is shown by the black circle, with the error bars corresponding to 95% CrIs.

For peer review only

Figure 3. Results of the random-effects network meta-analyses, for the percentage of patients achieving transfusion avoidance in the overall population, presented by the company[23] and as obtained from the case study re-analysis.

Footnote: Effect size estimates with black circles and red diamonds are from the analyses presented in TA1019 and the case study reanalysis, respectively.

Figure 4. Point-and-density plots for the comparisons of eculizumab to crovalimab and ravulizumab to crovalimab for the percentage of patients achieving transfusion avoidance in the overall population.

References

1. National Institute for Health and Care Excellence (NICE). NICE health technology evaluations: the manual, 2023. Available from: https://www.nice.org.uk/process/pmg36. Date accessed: Apr 25.
2. Thurgar E. The Evolution of Cost Comparison at NICE: What Can We Learn from Fast-Track Appraisals? 2022. p. https://www.ispor.org/docs/default-source/euro2022/thurgar-e-1-pdf.pdf?sfvrsn=494aa432_0. ISPOR Europe; 6-9 November Vienna, Austria
3. Armand J, Fontrier AM, Sawyer L. Cost-comparison, an easier route to NICE recommendations? 2023. p. https://www.ispor.org/docs/default-source/euro2023/isporeurope23armandhta357poster132726-pdf.pdf?sfvrsn=12a50a55_0. ISPOR Europe; 12-15 November; Copenhagen, Denmark.
4. Astill J, Roberts M, Cooper C. Overview of NICE Cost-Comparison Technology Appraisals: Key Takeaways Regarding Comparator Selection and Methods Used to Demonstrate Comparable Efficacy. 2024. p. https://www.ispor.org/docs/default-source/euro2024/isporeu24-hta194-astill143867-pdf.pdf?sfvrsn=c750ae89_0. ISPOR Europe; Barcelona, Spain.
5. Haycock M, Willows L, Wickstead R. Demonstrating Similar or Greater Health Benefits Based on Indirect Evidence: A Review of NICE Evaluations that Included a Cost-Comparison Approach. 2023. p. HTA34. ISPOR Europe; Copenhagen, Denmark.
6. National Institute for Health and Care Excellence (NICE). Technology appraisal methods and processes used before 2022, 2022. Available from: https://www.nice.org.uk/about/what-we-do/our-programmes/nice-guidance/nice-technology-appraisal-guidance/process. Date accessed: Apr 25.
7. National Institute for Health and Care Excellence (NICE). Guide to the processes of technology appraisal, 2018. Available from: https://www.nice.org.uk/Media/Default/About/what-we-do/NICE-guidance/NICE-technology-appraisals/technology-appraisal-processes-guide-apr-2018.pdf. Date accessed: May 26.
8. National Institute for Health and Care Excellence (NICE). Taking a proportionate approach to technology appraisals, 2024. Available from: https://www.nice.org.uk/about/what-we-do/proportionate-approach-to-technology-appraisals. Date accessed: Apr 25.
9. Walker E, Nowacki AS. Understanding equivalence and noninferiority testing. *J Gen Intern Med* 2011; **26**: 192-6.
10. Thiam A, Noon K, Micallef J, Porteous A. HTA151 The NICE Cost Comparison Pathway - How Much Evidence Is Required to Meet the Criteria? *Value in Health* 2022; **25**: S326.

11. Gewandter JS, McDermott MP, Kitt RA, Chaudari J, Koch JG, Evans SR, et al. Interpretation of CIs in clinical trials with non-significant results: systematic review and recommendations. *BMJ Open* 2017; **7**: e017288.

12. Esterhuizen TM, Mbuagbaw L, Thabane L. Disparity between statistical and clinical significance in published randomised controlled trials indexed in PubMed: a protocol for a cross-sectional methodological survey. *BMJ Open* 2024; **14**: e084375.

13. Schumi J, Wittes JT. Through the looking glass: understanding non-inferiority. *Trials* 2011; **12**: 106.

14. Fleming TR, Odem-Davis K, Rothmann MD, Li Shen Y. Some essential considerations in the design and conduct of non-inferiority trials. *Clinical Trials* 2011; **8**: 432-9.

15. Hashim M, Vincken T, Kroi F, Gebregergish S, Spencer M, Wang J, et al. A systematic review of noninferiority margins in oncology clinical trials. *Journal of comparative effectiveness research* 2021; **10**: 443-55.

16. York Health Economics Consortium (YHEC) - NICE Decision Support Unit. Clinical Equivalence and Non-Inferiority - Draft Report, 2020. Available from: https://yhec.co.uk/wp-content/uploads/2021/01/Clinical-Equivalence-and-Non-Inferiority-Report-Final-21Feb2020-2.pdf. Date accessed: May 25.

17. University of Sheffield - NICE Decision Support Unit. Full list of technical support documents (TSDs), 2025. Available from: https://www.sheffield.ac.uk/nice-dsu/tsds/full-list. Date accessed: May 25.

18. Edwards S, Dadswell C, Downes N, Burgess B, Walters A, Ip S. Evaluating clinical similarity: a systematic review of NICE guidance and the development of a Bayesian framework for a formal assessment of clinical similarity. PROSPERO 2025 CRD420251034143., 2024. Available from: https://www.crd.york.ac.uk/PROSPERO/view/CRD420251034143. Date accessed: Aug 25.

19. National Institute for Health and Care Excellence (NICE). 2025. Available from: https://www.nice.org.uk/. Date accessed: Aug 25.

20. Lee D, Allen A, Lovell A, Abdelsabour A, Wilson ECF, Melendez-Torrez GJ. How similar is similar enough? Assessment of indirect treatment comparisons to support similarity for NICE's cost comparison route. 2024. p. https://www.ispor.org/docs/default-source/euro2024/how-similar-is-similar-enough-cost-comparison-poster-ispor142603-pdf.pdf?sfvrsn=21e7eb7b_0. ISPOR Europe; Barcelona, Spain.

21. Lee D, Allen A, Lovell A, Abdelsabour A, Wilson ECF, Melendez-Torres GJ. How similar is similar enough? Assessment of indirect treatment comparisons to support similarity for NICE's cost comparison route. *Value in Health* 2025.

22. Brown A, Curry A, Ralston S, Cork D. PHP279 - ASSESSMENT OF THE NICE FAST-TRACK APPRAISAL (FTA) PROCESS. *Value in Health* 2018; **21**: S197.

23. National Institute for Health and Care Excellence (NICE). Crovalimab for treating paroxysmal nocturnal haemoglobinuria in people 12 years and over - Technology appraisal guidance [TA1019], 2024. Available from: https://www.nice.org.uk/guidance/ta1019. Date accessed: Apr 25.

24. National Institute for Health and Care Excellence (NICE). Crovalimab for treating paroxysmal nocturnal haemoglobinuria in people 12 years and over - Technology appraisal guidance [TA1019] - Final draft guidance, 2024. Available from: https://www.nice.org.uk/guidance/ta1019/history. Date accessed: Apr 25.

25. Röth A, He G, Tong H, Lin Z, Wang X, Chai-Adisaksopha C, et al. Phase 3 randomized COMMODORE 2 trial: Crovalimab versus eculizumab in patients with paroxysmal nocturnal hemoglobinuria naive to complement inhibition. *Am J Hematol* 2024; **99**: 1768-77.

26. Scheinberg P, Clé DV, Kim JS, Nur E, Yenerel MN, Barcellini W, et al. Phase 3 randomized COMMODORE 1 trial: Crovalimab versus eculizumab in complement inhibitor-experienced patients with paroxysmal nocturnal hemoglobinuria. *Am J Hematol* 2024; **99**: 1757-67.

For peer review only - http://bmjopen.bmj.com/site/about/guidelines.xhtml

27. National Institute for Health and Care Excellence (NICE). Acalabrutinib for treating chronic lymphocytic leukaemia - Technology appraisal guidance [TA689], 2021. Available from: https://www.nice.org.uk/guidance/ta689. Date accessed: Apr 25.

28. National Institute for Health and Care Excellence (NICE). Zanubrutinib for treating chronic lymphocytic leukaemia - Technology appraisal guidance [TA931], 2023. Available from: https://www.nice.org.uk/guidance/ta931. Date accessed: May 25.

29. Poley MJ, Armstrong N, Yang H, Patel M, Stirk L, Al MJ, et al. Cost Comparisons in NICE Technology Appraisals: An External Assessment Group Perspective. *PharmacoEconomics* 2025.

30. National Institute for Health and Care Excellence. Nivolumab with chemotherapy for neoadjuvant treatment then alone for adjuvant treatment of resectable non-small-cell lung cancer [TA1127] - Committee papers, 2026. Available from: https://www.nice.org.uk/guidance/TA1127. Date accessed: May 26.

31. Bland JM, Altman DG. Bayesians and frequentists. *BMJ* 1998; **317**: 1151-60.

32. Sadeghirad B, Foroutan F, Zoratti MJ, Busse JW, Brignardello-Petersen R, Guyatt G, et al. Theory and practice of Bayesian and frequentist frameworks for network meta-analysis. *BMJ Evidence-Based Medicine* 2023; **28**: 204-9.

33. Sehmbi H, Retter S, Shah UJ, Nguyen D, Martin J, Uppal V. Epidemiological, methodological, and statistical characteristics of network meta-analysis in anaesthesia: a systematic review. *British Journal of Anaesthesia* 2023; **130**: 272-86.

34. Phillippo DM, Dias S, Ades AE, Belger M, Brnabic A, Schacht A, et al. Multilevel Network Meta-Regression for Population-Adjusted Treatment Comparisons. *Journal of the Royal Statistical Society Series A: Statistics in Society* 2020; **183**: 1189-210.

For peer review only

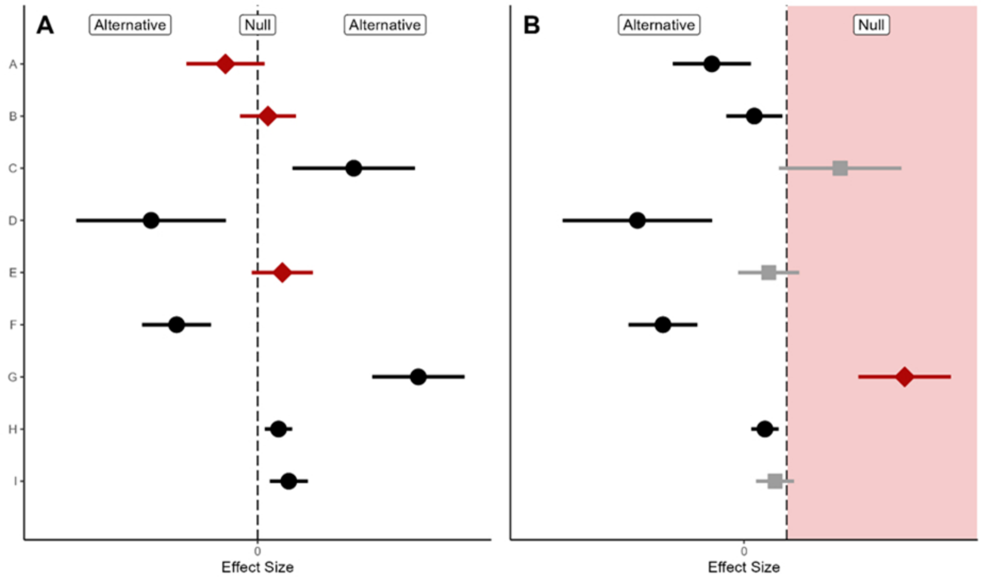

Caption: Figure 1. llustration of traditional statistical significance testing (A) and non-inferiority testing (B).

Footnote: Points denote effect sizes, while lines denote 95% credible intervals. Dashed lines correspond to the thresholds of the null and alternative hypotheses. For traditional significance testing the dashed line corresponds to either 0 or 1 (depending on the given effect size). For non-inferiority testing the dashed line corresponds to the non-inferiority margin. Areas without shading correspond to the alternative hypothesis, areas with red shading correspond to the null hypothesis. Red diamonds denote effect sizes where the null hypothesis was accepted. Black circles denote effect sizes where the null hypothesis was rejected. Grey squares denote effect sizes where there is insufficient evidence to reject the null hypothesis.

404x243mm (59 x 59 DPI)

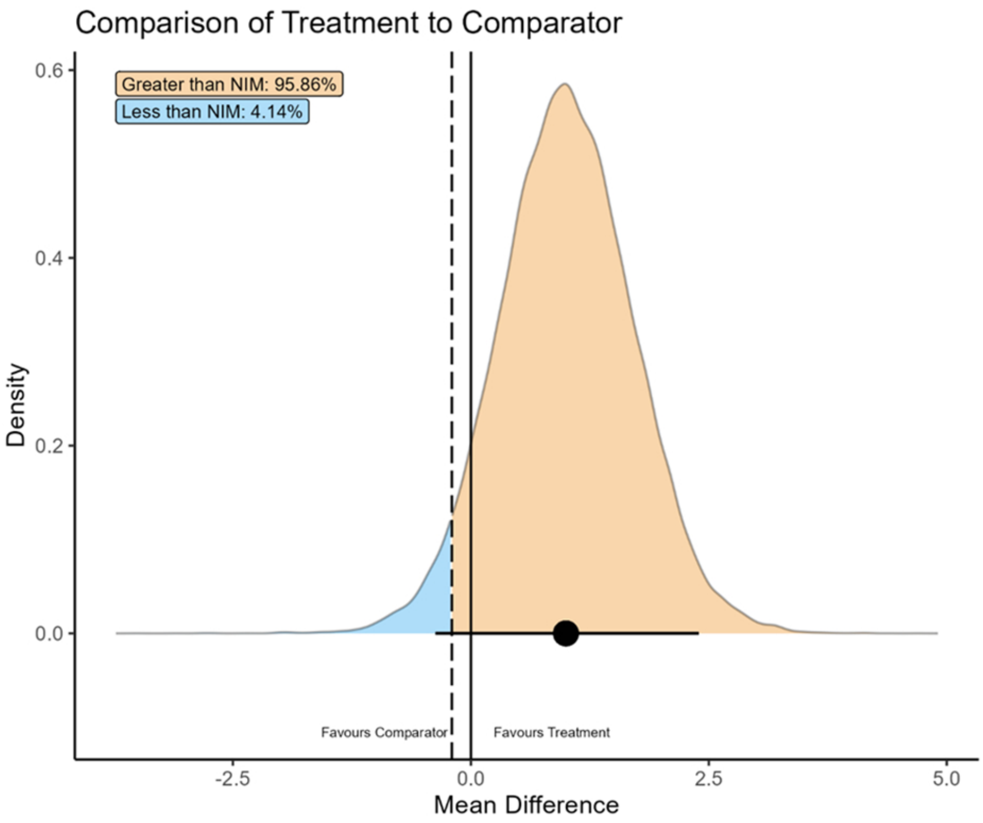

Caption: Figure 2. Point-and-density plot for the comparison of an example treatment and comparator.

Footnote: The threshold for traditional hypothesis testing is shown by the solid line, while the dashed line indicates the NIM. The area that falls under (above) the NIM is shown in blue (orange). The probability that the effect size falls under, or above, the NIM is shown in the upper left corner of the plot. The pooled estimate is shown by the black circle, with the error bars corresponding to 95% CrIs.

404x337mm (59 x 59 DPI)

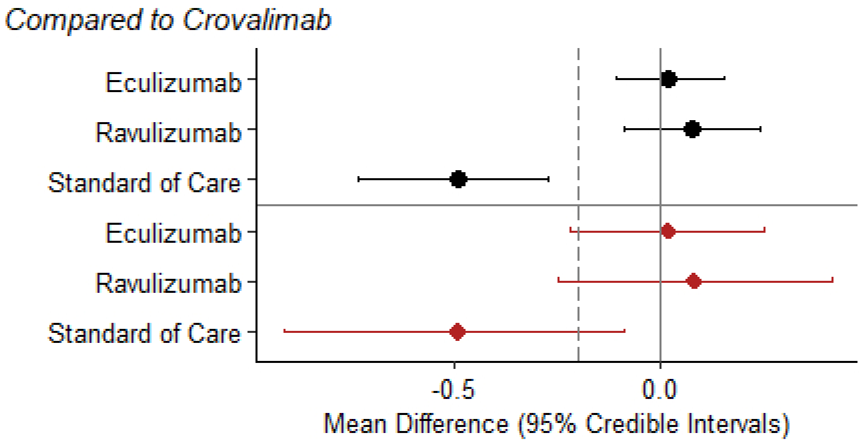

Caption: Figure 3. Results of the random-effects network meta-analyses, for the percentage of patients achieving transfusion avoidance in the overall population, presented by the company and as obtained from the case study re-analysis.

Footnote: Effect size estimates with black circles and red diamonds are from the analyses presented in TA1019 and the case study reanalysis, respectively.

295x154mm (38 x 38 DPI)

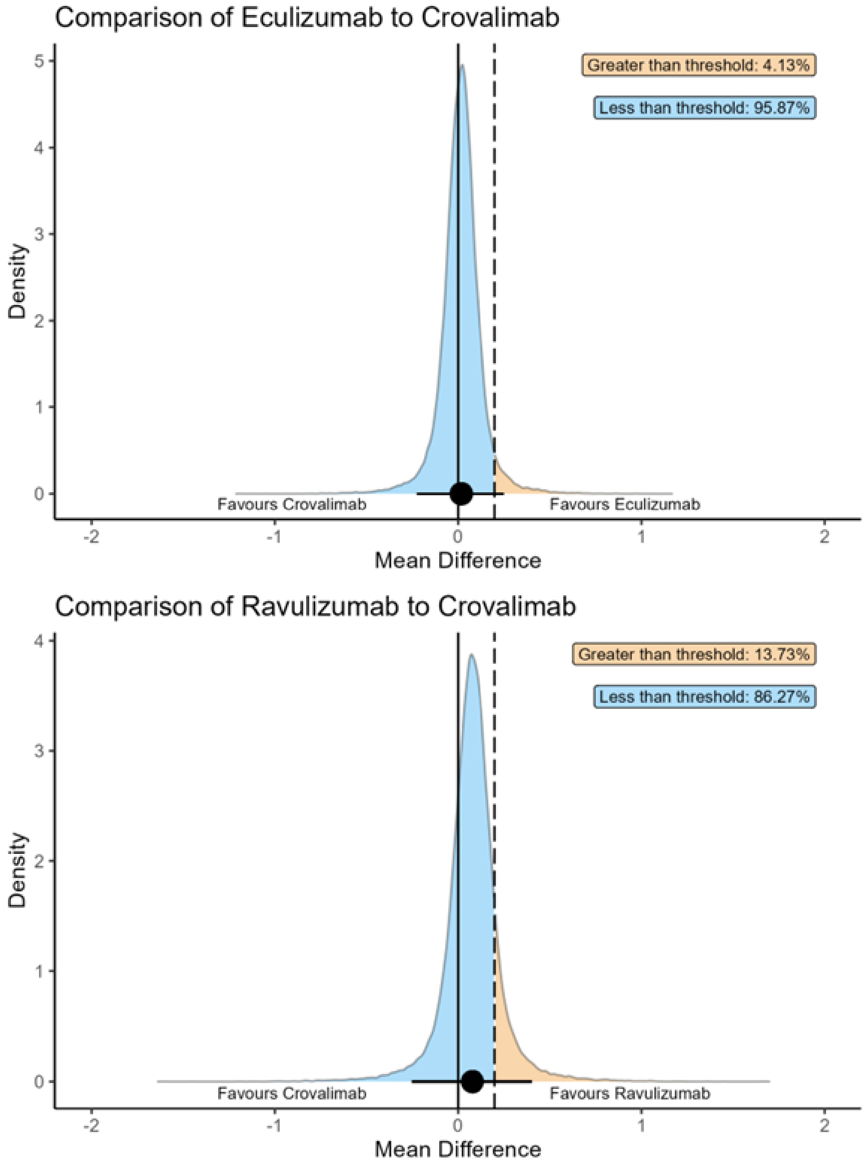

Caption: Point-and-density plots for the comparisons of eculizumab to crovalimab and ravulizumab to crovalimab for the percentage of patients achieving transfusion avoidance in the overall population.

402x536mm (38 x 38 DPI)

BMJ TAG

Evaluating clinical similarity: a systematic review of NICE guidance and the development of a Bayesian framework for a formal assessment of clinical similarity

Protocol

April 2025

For peer review only

1 Title of the project

Evaluating clinical similarity: a systematic review of NICE guidance and the development of a Bayesian framework for a formal assessment of clinical similarity.

2 Name of TAR team and project 'lead'

BMJ Technology Assessment Group (BMJ-TAG)

Lead: Steve Edwards

Director of Health Technology Assessment

BMJ Group

5th Floor, 90 Whitfield Street, London W1T 4EZ

For peer review only

For peer review only - http://bmjopen.bmj.com/site/about/guidelines.xhtml

3 Plain English Summary

Cost-comparison evaluations (CCEs) are one type of technology appraisal being used by the National Institute for Health and Care Excellence (NICE) to assess the clinical and cost effectiveness of new treatments. It allows new treatments to be compared against a comparator treatment in the same indication that has already been recommended by NICE and is likely to be "clinically similar", with no requirement for complex health economic modelling and a process that aims to be faster than assessments requiring more complex modelling.

Deciding whether two treatments are "clinically similar" can be difficult if there are no trials directly comparing the two treatments and an indirect treatment comparison (ITC) has been required. ITCs often report no statistically significant difference, which cannot itself be interpreted as evidence of two treatments being clinically similar. There is thought to be variation in terms of how clinical similarity is currently assessed in these cases within NICE CCEs. The availability of thresholds such as minimally clinically important differences (MCIDs) may help with the interpretation of clinical similarity, but these are not always available and it is unclear how often these or other methods are used to support decisions about clinical similarity within NICE CCEs.

This research aims to review all CCEs performed as part of the NICE technology appraisal programme since 2017 to identify and summarise any variations in the methods used to support decisions about clinical similarity when ITCs have been required. Subsequent to this, a novel Bayesian framework will be developed with the aim of supporting the interpretation of non-significant results from Bayesian network meta-analyses (NMAs). Worked examples demonstrating the use of the new tool will also be provided.

For peer review only - http://bmjopen.bmj.com/site/about/guidelines.xhtml

4 Research question

4.1 Purpose

Technology appraisals, such as those performed in England as part of the National Institute for Health and Care Excellence (NICE) technology appraisal programme, involve the assessment of the clinical and cost-effectiveness of specific drugs, technologies or other interventions. Most appraisals require complex health economic models, such as cost-utility analyses, to assess whether the intervention of interest is likely to be a good use of NHS resources. However, since 2017, cost comparison evaluations (CCEs) have been accepted within the NICE technology appraisal programme under specific circumstances. CCEs involve a simpler comparison of costs and resources used with one drug compared to another, without the need for a complex model such as those used in cost-utility analyses, but they require a strong assumption that the drugs being compared are similar in terms of clinical effectiveness.

CCEs were first introduced as one option under the fast-track appraisal (FTA) process in 2017; while the FTA process itself was replaced in 2022, its replacement was a process focusing only on CCEs and methods relating to the CCE itself did not notably change. For an appraisal to be suitable for the cost comparison route, Section 4.2.13 of the 2022 NICE health technology evaluations manual states that the new interventions should be, “likely to provide similar or greater health benefits at similar or lower cost than the relevant comparator(s)”; relevant comparators are considered to be, “those recommended in published NICE guidance for the same population”,[1] which is consistent with requirements described for the CCE process prior to 2022.[2] The aim of CCEs is to provide an assessment of clinical and cost-effectiveness for suitable drugs that can be used to decide whether the they should be recommended but in a shorter time frame and using fewer resources compared to technology appraisals using cost-utility methods, as part of NICE’s commitment to taking a proportionate approach to technology appraisals.[3]

While the clinical similarity of two interventions can be robustly demonstrated through non-inferiority randomised controlled trials (RCTs) using prespecified non-inferiority thresholds or clinical equivalence RCTs,[4] many CCEs submitted to NICE do not involve such trials. Instead, they rely on the results of indirect treatment comparisons (ITCs) such as network meta-analyses (NMAs), matching-adjusted indirect comparisons (MAICs) or simple Bucher ITCs. These analyses often result in non-

For peer review only - http://bmjopen.bmj.com/site/about/guidelines.xhtml

significant results based on confidence intervals or credible intervals that cross the line of null effect. A lack of a statistically significant difference should not be used to determine that two treatments are clinically similar; these analyses are often based on a small number of studies with small sample sizes, meaning there is considerable uncertainty in the effect estimate that should not be interpreted as an absence of a difference. Therefore, the interpretation of these results in terms of whether interventions can be considered clinically similar can be challenging. The use of minimally clinically important differences (MCIDs) established in the literature or estimated through other methods may aid the interpretation of results and the transparency of this process;[5] if the point estimates and uncertainty interval obtained from the ITC were less than (or mostly less than) the MCID, it may increase the confidence of decision-makers that the treatments are most likely to be clinically similar, as any difference that is present is unlikely to be clinically meaningful. Alternatively, if the point estimate and uncertainty interval are greater than the MCID and in favour of the new intervention, there would be increased confidence that the new treatment may be better than the previously recommended intervention. However, established MCIDs are not available for all outcomes and it is unclear how often these methods are used currently to assess clinical similarity in NICE CCEs.

Through a systematic literature review (SLR), this research aims to review and summarise the basis for decision-makers assuming clinical similarity based on non-significant results obtained from ITCs as part of NICE CCEs, such as how often MCIDs or other approaches are used for interpretation and decision-making, and which thresholds have been used so far. The results of the SLR will support the development of a novel Bayesian framework that can integrate MCIDs or other thresholds to support the interpretation of non-significant results from Bayesian NMAs. Worked examples demonstrating the use of the tool, taken from the CCEs identified in the SLR, will be provided as part of this research. The SLR will highlight whether inconsistencies exist in current decisions or whether thresholds for clinical similarity have emerged organically from decision-makers, while the proposed framework should allow for consistent, robust, and reproducible decision-making for future CCEs.

For peer review only - http://bmjopen.bmj.com/site/about/guidelines.xhtml

4.2 Content of the systematic literature review

The SLR will aim to identify and include all NICE technology appraisals that:

- have followed the CCE approach for decision-making from the outset;
- rely on non-significant results from ITCs to assess clinical similarity for at least one comparator for the primary outcome(s);
- have published final guidance at the time of searching or where this can be obtained via NICE for appraisals due to publish final guidance imminently (i.e. before completion of the SLR).

Appraisals that only involve a non-inferiority or equivalence RCT (i.e. there are no comparators for which an ITC has been required) will not be included given these trials are considered to be robust methods of assessing clinical similarity and are not subject to the same uncertainties as results from ITCs. Similarly, appraisals that only include ITCs with statistically significant results will not be included, as there is less uncertainty associated with these results. Appraisals that include direct RCT evidence or statistically significant ITC results for one comparator but rely on the non-significant results of an ITC for another comparator will only be included if there are clear statements about the committee or external assessment group (EAG)'s conclusion of clinical similarity for the comparison where the ITC with non-significant results was performed; this is because clinical similarity only has to be demonstrated against one comparator in the NICE CCE process, meaning if multiple comparators are deemed appropriate, the decision-making is likely to have been based mostly on the one with the strongest evidence (in this case the one with direct RCT evidence or statistically significant ITC results). During the sifting stage, details about which appraisals involved decisions based on statistically significant results from ITCs or direct comparisons between treatments (and whether these were superiority, non-inferiority or equivalence trials) will be recorded, to allow brief comments to be made on this despite their exclusion from the main review.

Appraisals that have followed the CCE process from the start of the appraisal (i.e. where the company's first submission is a CCE) will be prioritised for inclusion. It is possible that some Single Technology Appraisals (STAs) start with cost-utility models but incorporate cost

For peer review only - http://bmjopen.bmj.com/site/about/guidelines.xhtml

comparison approaches following requests from the EAG or committee. If time permits, these will be considered for inclusion but only if it is clear that decision-making was solely based on the cost comparison assessment given the exact contribution of the CCE to decision-making would otherwise be difficult to disentangle. An appraisal that has been a CCE from the start will usually be evident from the introductory pages of the NICE committee papers or the decision problem table submitted by the company, but other sections of the appraisal documents will be explored where this is unclear. Conclusions about whether decisions were based solely on cost comparison analyses for STAs may be available from committee discussion sections included in final guidance documents, but where this is unclear the appraisal will be excluded.

The review will aim to cover all CCEs meeting the above criteria from 2017 onwards; however, upon identification of relevant appraisals, prioritisation of those from 2022 onwards (given these represent the most recent examples of the NICE CCE process) may be required if the workload associated with this outweighs the resources available for this project. Due to the time constraints of this review, only the primary outcome(s) in each appraisal will be assessed with regards to how the decision of clinical similarity was made. It is anticipated that the documenting of which outcome is the primary outcome may be unclear in some appraisals; in this review, if not otherwise stated, the outcome that is discussed first, is most heavily discussed in the report or NICE committee meeting slides and/or is key in terms of any economic assessment presented will be considered to be the primary outcome. In cases where it appears there is more than one primary outcome, these will be considered separately. A clear rationale for why specific outcomes in each appraisal have been interpreted as the primary outcome will be included in the extraction tables.

4.3 Development of a Bayesian framework for a formal assessment of clinical similarity

The results of the SLR described in Section 4.2 will be used to support the development of a novel framework that utilises Bayesian NMAs to allow non-significant results to be interpreted more robustly. The framework will allow the inclusion of MCIDs or other thresholds when available to support with the interpretation of non-significant results. It is anticipated that this will involve building on existing, commonly used approaches such as cumulative density functions, point estimates, and credible intervals. Outputs will be designed such that they are clear and easily

For peer review only - http://bmjopen.bmj.com/site/about/guidelines.xhtml

interpretable by stakeholders regardless of their statistical background. Worked examples demonstrating the use of the tool, taken from the CCEs identified in the SLR, will also be provided as part of this research. The methods for this phase of the research are not described further in this protocol but will be written up in the final report.

5 Methods for the systematic literature review

Methods described here are specifically for the systematic literature review (SLR) phase of this research, as outlined in Section 4.2. More detailed methods associated with the development of the Bayesian framework outlined in Section 4.3 are not included here but will be covered in the final report.

The SLR will be performed and reported according to the PRISMA statement. A flow diagram illustrating the number of records identified, included and excluded throughout the SLR will be presented according to the PRISMA reporting guidelines.[6]

5.1 Search strategy

Multiple free-text searches of the National Institute for Health and Care Excellence (NICE) website will be performed by one reviewer. The terms "cost comparison", "cost minimisation" and "cost minimization" (including the quotation marks) will be searched using the search bar at the top of the main NICE webpage (https://www.nice.org.uk/). This will pick up any records including the exact terms "cost comparison" or "cost-comparison" while avoiding records that mention cost and comparison but not adjacently (or equivalent for cost minimisation and cost minimization). This is considered to be a reasonable approach given the term "cost comparison" is a formal and consistent term used by NICE in these appraisals and is likely to be mentioned somewhere in all appraisals that have followed the cost comparison evaluation (CCE) route. The terms "cost minimisation" and "cost minimization" will be included to identify any records that may have used this terminology instead, which can also be used to refer to the cost comparison approach in health economic assessment.

The results of the search will be further refined to show only technology appraisal guidance; the option to filter by guidance programme on the left-hand side of the search results page will be used

For peer review only - http://bmjopen.bmj.com/site/about/guidelines.xhtml

to achieve this by selecting the “technology appraisal guidance” box. All records remaining after applying this filter will be screened for inclusion against the criteria outlined in Section 4.2.

For records that have not had final guidance published at the time of screening and that are due to publish before completion of the SLR, one reviewer will contact NICE to determine whether information on final guidance can be obtained to allow inclusion in this assessment. During the screening process, the contact person for appraisals that have not yet published final guidance will be captured to facilitate this. NICE will also be contacted with a full list of CCEs identified from the search of the NICE website, to ask whether it is aware of any other CCEs not captured by the search.

5.2 Review process

Given it is not possible to export records from the NICE website, records obtained from the search described in Section 5.1 will be manually added to a Microsoft Excel® spreadsheet for tracking and screening purposes. Records will then be screened for inclusion against the criteria outlined in Section 4.2 by a single reviewer. This will involve reviewing any information and documents published on the NICE website (or provided in communication with NICE) for each record in a single screening step; information within guidance documents and committee papers will be prioritised, but other documents such as final scopes may be reviewed if information remains unclear or if other documents with key information are identified. Validation of this process will be performed by a second reviewer. Any disagreement will be resolved through discussion and involvement of a third reviewer if required to resolve outstanding conflicts.

5.3 Data extraction

Data for each included record will be extracted by a single reviewer into standardised data extraction forms using Microsoft Word®. Validation of a representative 30% sample of extractions will be performed by a second reviewer, with discussion of any discrepancies and involvement of a third reviewer if required. A draft data extraction form is included in Appendix 8.1 but is subject to change; a pilot of five extractions will be performed before validation by a second reviewer, after which the data extraction form will be finalised and used for the remaining extractions.

General information about each record such as project name, project ID and disease area will be extracted, as well as information about primary outcomes, comparators that the ITC was required

For peer review only - http://bmjopen.bmj.com/site/about/guidelines.xhtml

for and type of ITC performed, date of final guidance publication and whether or not the treatment was recommended. Information about clinical similarity and reasons that decisions were made are likely to be reported in various ways across projects, but reviewers will be exhaustive and as a minimum will capture any information relating to:

- Any mention of ways of interpreting ITC results such as specific MCIDs or other thresholds and how these were identified;
- Results of the committee's preferred ITCs for relevant comparisons, including point estimates and measures of uncertainty (such as 95% confidence or credible intervals) of the effect estimates;
- Any committee or EAG comments on the uncertainty in the ITC, including what they thought about the uncertainty in the ITC and how it may have impacted their decision or conclusions;
- Brief comments on the availability of input data for the ITCs from NICE papers, to allow an assessment of how feasible it would be to replicate the ITCs from this information (and whether identification of data from individual publications would instead be required) for the worked Bayesian framework examples that will be provided as part of the second phase of this project;
- Narrative comments within company submissions, EAG reports or committee discussion documents describing how results of ITCs were ultimately interpreted and how clinical similarity was confirmed or refuted in the final decision-making process; clear statements from committee discussions will be most useful, but in the absence of this inferences from EAG reports about preferred analyses or interpretation may be required.

Extractions will include details on the location that each piece of information was identified to facilitate validation. Where multiple versions of an ITC are available across different documents (for example, an ITC performed within the company submission may have been adapted or amended by the EAG), methods and results relating to the committee's preferred analysis only will be extracted, which should be evident from the guidance documents. Where this information is uncertain, the EAG's preferred analysis will be preferentially extracted unless there is evidence from supporting information that the committee dismissed this analysis in favour of the company's original analysis.

For peer review only - http://bmjopen.bmj.com/site/about/guidelines.xhtml

Details related to interpretation and decision making will be captured using quotation marks in extraction tables to ensure that detail is not lost during this process. Guidance documents and committee papers available on the NICE website will be prioritised for review during data extraction but review of other documents such as final scopes may be required if information remains unclear or if other documents with key information are identified. Any additional information provided by NICE will be considered where applicable.

5.4 Quality assessment

Given that the records to be included in this review are NICE technology appraisals rather than clinical trials such as randomised controlled trials (RCTs), non-randomised studies or qualitative studies, a quality assessment process will not be incorporated as part of this review.

5.5 Synthesis

Information extracted from included records will be summarised narratively and using summary tables, with similarities and differences across the identified CCEs noted. The narrative synthesis will focus mostly on the ways in which clinical similarity was confirmed or refuted, including:

- similarities and differences across appraisals with regards to methods used, such as specific thresholds that have been used and how they were derived (e.g. elicitation, arbitrary or other) or other methods of assessing clinical similarity;
- the rationale for the methods used;
- how common each method of assessing clinical similarity is;
- whether there are any differences in how similar methods or thresholds have been interpreted across appraisals.

Any other key aspects identified throughout the review may also be covered. Specific CCEs may be highlighted as examples of different methods used to assess clinical similarity, such as use of MCIDs or other thresholds. Key strengths, limitations and uncertainties will be highlighted and summarised where appropriate.

For peer review only - http://bmjopen.bmj.com/site/about/guidelines.xhtml

6 Contribution of the research group:

Steve Edwards	Director of Health Technology Assessment, BMJ-TAG, London. Conception of the research project and provision of research framework. Guidance and validation of the work of the research group at all stages; provision of feedback on all versions of the protocol and the report. Guarantor of the report.
Clare Dadswell	Clinical Evidence Manager, BMJ-TAG, London. Contribution to the development of the project. Providing feedback to resolve any outstanding conflict between the first two reviewers on the systematic literature review.
Nicole Downes	Senior Clinical Evidence Analyst, BMJ-TAG, London. Lead on the development of this protocol. Lead reviewer on the systematic literature review of NICE guidance, including development of search strategy, running searches, sifting of results, data extraction and narrative synthesis of results. Drafting sections of the final report related to the systematic literature review.
Ben Burgess	Senior Clinical Evidence Analyst, BMJ-TAG, London. Leading on the development of a Bayesian framework for a formal assessment of clinical similarity. Testing the developed framework using examples identified through the systematic literature review. Drafting sections of the final report related to the Bayesian framework.
Archie Walters	Health Economist, BMJ-TAG, London. Second reviewer on the systematic literature review of NICE guidance, involved in validation of the screening stage and validation of a proportion of the extractions.
Sophie Ip	Senior Health Economist, BMJ-TAG, London. Supporting during the Bayesian framework development stage by validating code and analyses.

For peer review only

For peer review only - http://bmjopen.bmj.com/site/about/guidelines.xhtml

7 Timetable/Milestones

Key dates for this project are outlined in Table 1 below and are mostly related to the systematic literature review (SLR) phase of this research.

Table 1 Draft milestones table

Milestone	Start date	End date
Project start date	07/04/2025	07/04/2025
Draft protocol development	07/04/2025	11/04/2025
SLR searching, sifting, extraction and validation	14/04/2025	02/05/2025
SLR synthesis and report writing	06/05/2025	06/06/2025
Work on Bayesian framework including report writing	07/04/2025	18/06/2025
Project submission	19/06/2025	19/06/2025

8 Appendices

8.1 Draft data extraction form

Appraisal	Appraisal name (ID) {reference}
Appraisal details	**Comparator(s) included:**<br>• **ITC required for:**<br><br>**Source:**
	**Outcomes included in the ITCs:**<br><br>**Outcome(s) considered to be primary outcomes by reviewer:**<br>•<br>•<br>•<br><br>(defined as primary outcomes…)<br><br>**Outcome scales and thresholds:**<br>•<br>•<br>•<br><br>**Source:**
	**Final appraisal decision:**

For peer review only - http://bmjopen.bmj.com/site/about/guidelines.xhtml

	**Source:**
Details of committee's preferred ITCs	**Preferred ITCs:**<br><br>**Source:**
ITC results – committee's preferred analyses	***Comparison 1 -***<br>***Outcome 1***<br>• **Effect estimate from ITC:** (e.g. RR and 95% CI)<br>• **Statistical significance from ITC:**<br>• **Source:**<br>• **Availability of ITC input data for replication:**<br><br>***Outcome 2***<br>• **Effect estimate from ITC:**<br>• **Statistical significance from ITC:**<br>• **Source:**<br>• **Availability of ITC input data for replication:**<br><br>***Outcome 3***<br>• **Effect estimate from ITC:**<br>• **Statistical significance from ITC:**<br>• **Source:**<br>• **Availability of ITC input data for replication:**
	Comparison 2<br>***Outcome 1***<br>• **Effect estimate from ITC:**<br>• **Statistical significance from ITC:**<br>• **Source:**<br>• **Availability of ITC input data for replication:**
	Comparison 3<br>***Outcome 1***<br>• **Effect estimate from ITC:**<br>• **Statistical significance from ITC:**<br>• **Source:**<br>• **Availability of ITC input data for replication:**

<table>
<tr><td rowspan="3">Methods used to assess clinical similarity and conclusions</td><td>Company approach
Method used and rationale:
Details of thresholds used:
Conclusions for each outcome:
Uncertainty in the ITC results and impact on decision-making:
Comments:
Source:</td></tr>
<tr><td>EAG approach
Method used and rationale:
Details of thresholds used:
Conclusions for each outcome:
Uncertainty in the ITC results and impact on decision-making:
Comments:
Source:</td></tr>
<tr><td>Committee approach
Method used and rationale:
Details of thresholds used:
Conclusions for each outcome:
Uncertainty in the ITC results and impact on decision-making:
Comments:
Source:</td></tr>
<tr><td>Comments</td><td></td></tr>
<tr><td colspan="2">Abbreviations:</td></tr>
</table>

9 References

1. National Institute for Health and Care Excellence (NICE). NICE health technology evaluations: the manual, 2023. Available from: https://www.nice.org.uk/process/pmg36. Date accessed: Apr 25.

2. National Institute for Health and Care Excellence (NICE). Technology appraisal methods and processes used before 2022, 2022. Available from: https://www.nice.org.uk/about/what-we-do/our-programmes/nice-guidance/nice-technology-appraisal-guidance/process. Date accessed: Apr 25.

3. National Institute for Health and Care Excellence (NICE). Taking a proportionate approach to technology appraisals, 2024. Available from: https://www.nice.org.uk/about/what-we-do/proportionate-approach-to-technology-appraisals. Date accessed: Apr 25.

4. Walker E, Nowacki AS. Understanding equivalence and noninferiority testing. *J Gen Intern Med* 2011; **26**: 192-6.

5. Klukowska AM, Vandertop WP, Schröder ML, Staartjes VE. Calculation of the minimum clinically important difference (MCID) using different methodologies: case study and practical guide. *Eur Spine J* 2024; **33**: 3388-400.

6. Page MJ, McKenzie JE, Bossuyt PM, Boutron I, Hoffmann TC, Mulrow CD, et al. The PRISMA 2020 statement: an updated guideline for reporting systematic reviews. *BMJ* 2021; **372**: n71

For peer review only - http://bmjopen.bmj.com/site/about/guidelines.xhtml

Online Supplementary File 2

Systematic Literature Review (SLR) methods

Inclusion and exclusion criteria

This review aimed to include National Institute for Health and Care Excellence (NICE) cost-comparison evaluations (CCEs) from 2017 onwards that followed the CCE approach from the outset, relied on results from indirect treatment comparisons (ITCs) that were not statistically significant for at least one comparator for the primary outcome(s) and had published final guidance before completion of the review. Appraisals that had direct randomised controlled trial (RCT) evidence or statistically significant ITC results for one comparator but relied on the non-significant results of an ITC for at least one other comparator were considered for inclusion providing there were clear statements from the External Assessment Group (EAG) and/or committee about where clinical similarity can be concluded for comparators relying on the latter.

Appraisals where the results from ITCs were all statistically significant were excluded given there is less uncertainty associated with these results; however, it is possible that some appraisals that were based solely on statistically significant ITC results have been included in the review, given it was sometimes difficult to decipher due to redaction of the results and discussion of statistical significance in the published NICE papers. Where it was clear that most emphasis was placed on specific outcomes for decision-making, these appraisals were excluded if the ITC results for these outcomes were statistically significant, even if there were some non-significant differences for other outcomes that were not key to decision-making. This was a pragmatic decision to try to identify appraisals where decisions were made based on the most uncertainty from ITCs, given that the presence of statistically significant differences for the key outcomes is likely to outweigh any more uncertain results for outcomes considered less important as part of the decision-making process. Only two appraisals were excluded for this reason.

In the protocol for this review, the researchers noted that appraisals that originated as a CCE, i.e. where the company's first submission was a CCE, would be prioritised for inclusion. It was also stated that appraisals that started with cost-utility models but later incorporated cost-comparison approaches would be considered for inclusion if it was clear that decision-making was solely based on the cost-comparison evidence but only if time permitted; given that 41 appraisals with a CCE approach as its first submission were

For peer review only - http://bmjopen.bmj.com/site/about/guidelines.xhtml

identified, there was insufficient resource available to consider inclusion of appraisals that adopted a CCE approach later in the process.

Definition of primary outcomes in this SLR

In line with the review protocol, reviewers only extracted information relating to outcomes that were considered to be primary outcomes for each appraisal. NICE papers did not often outline which outcomes were primary outcomes for decision-making purposes, but in the absence of this being explicitly stated, reviewers often based this decision on which were primary outcomes in the key trials of the intervention in each appraisal or which were noted in the submissions as being key for economic models of prior appraisals in the same area. While this may have led to some inconsistencies in terms of which outcomes were extracted as primary outcomes for different appraisals in the same or similar disease area, the researchers note that all general statements relating to how companies, EAGs and committees interpreted results and made conclusions on clinical similarity overall within the appraisal have been extracted, so key information relating to methods for concluding clinical similarity should not have been lost. Adverse events were not included as primary outcomes for any appraisals as in the researchers' experience there are usually very limited data on which to base conclusions about comparability of safety profiles between treatments within ITCs, given there are commonly few events and substantial uncertainty, and in many cases ITCs are not performed for safety outcomes for these reasons, with conclusions about these outcomes likely to be based more heavily on supplementary information such as feedback from clinical experts.

For peer review only

Data extraction

Identifying and summarising information about clinical similarity conclusions and the rationale or methods to make these conclusions was a key aim of this systematic literature review (SLR) and the following were extracted from included appraisals to allow approaches and patterns to be identified and discussed:

- Any mention of ways of interpreting ITC results such as specific minimal clinically important difference (MCID) thresholds or other thresholds and how these were identified;
- Results of the committee's preferred ITCs for relevant comparisons, including point estimates and measures of uncertainty (such as 95% confidence or credible intervals) of the effect estimates;

- Any committee or EAG comments on the uncertainty in the ITC results, including what they thought about the uncertainty in the ITC results and how it may have impacted their decision or conclusions;
- Narrative comments within company submissions, EAG reports or committee discussion documents describing how results of ITCs were ultimately interpreted and how clinical similarity was confirmed or refuted in the final decision-making process; clear statements from committee discussions were considered most useful, but were often not available, so often inferences from EAG reports about preferred analyses or interpretation were required.

Where multiple versions of an ITC were available across different documents (for example, an ITC performed within the company submission may have been adapted or amended by the EAG), methods and results relating to the committee's preferred analysis only were extracted where this was clear. In some cases, results of all analyses were discussed as a whole, in which case results of each analysis were extracted separately. Where the committee's preferred analysis was unclear, the EAG's preferred analysis was assumed to be the committee's preferred analysis unless there was evidence from supporting information that the committee dismissed this analysis in favour of the company's original analysis or that it considered the results of multiple analyses when making decisions.

Details related to interpretation and decision making were captured using quotation marks in extraction tables to ensure that detail was not lost during this process. Guidance documents and committee papers available on the NICE website were prioritised for review during data extraction, with public committee slides also checked where available to identify any additional information. For appraisals where the first committee meeting included a CCE that was then rejected, only NICE papers related to the first committee meeting were reviewed, as the papers relating to the second committee meeting were not likely to provide any further information on the conclusions of the CCE stage. Review of other documents such as NICE final scopes was rarely required.

For peer review only

SLR results

Supplementary Figure 1. PRISMA flow diagram of records included in the SLR

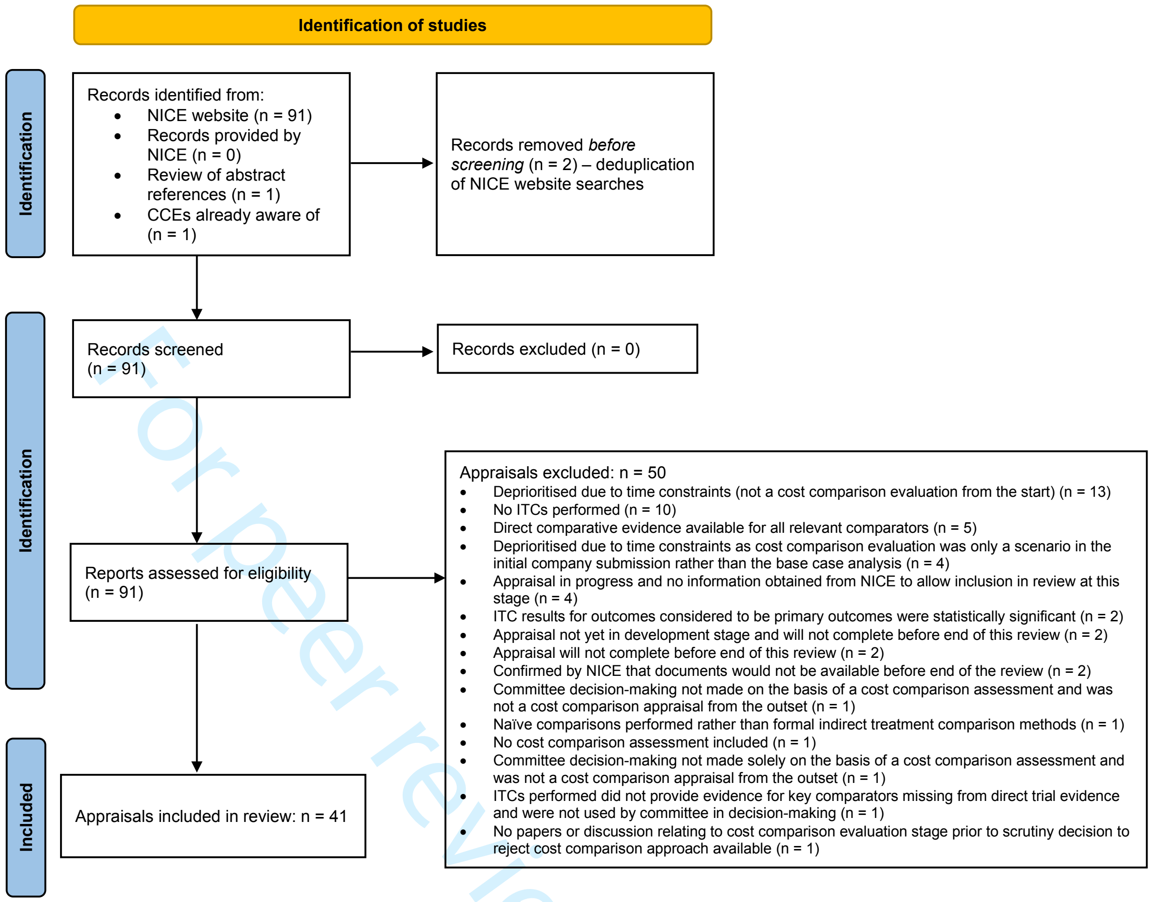

Abbreviations: CCE, cost-comparison evaluation; ITC, indirect treatment comparison; NICE, National Institute for Health and Care Excellence; PRISMA, Preferred Reporting Items for Systematic Reviews and Meta-Analyses; SLR, systematic literature review.

For peer review only

Supplementary Table 1. Summary of NICE technology appraisals included in the SLR

Appraisal name (ID)	Disease area	Date published	ITC outcomes considered primary in this review	Other ITC outcomes	Comparators with only ITC evidence	Statistically significant ITC results for primary outcomes?	Comparators with direct evidence (superiority or non-inferiority)	Type of ITCs
Aflibercept for treating choroidal neovascularisation (TA486)[1]	Choroidal neovascularisation	November 2017	Gain in BCVA from baseline	None	Ranibizumab	None	None	NMAs
Golimumab for treating non-radiographic axial spondyloarthritis (TA497)[2]	Axial spondyloarthritis	January 2018	ASAS20; ASAS40; BASDAI50	CFB in BASFI; CFB in BASDAI; CFB in BASMI; AEs; serious AEs; infections	Adalimumab; etanercept; certolizumab pegol	None	None	NMAs
Guselkumab for treating moderate to severe plaque psoriasis (TA521)[3]	Plaque psoriasis	June 2018	PASI 75 response	PASI 50 response; PASI 90 response; PASI 100 response; PGA/IGA; HRQoL; AEs; serious AEs; withdrawal due to AEs	Secukinumab; ixekizumab; etanercept; infliximab<br><br>(first two considered key by committee and included here)	Statistically significant for comparison against 300 mg secukinumab 300 mg but not against 80 mg ixekizumab	Adalimumab (2 superiority trials); ustekinumab (1 superiority trial)	NMAs
Ertugliflozin as monotherapy or with metformin for treating type 2 diabetes (TA572)[4]	Type 2 diabetes	March 2019	% CFB in HbA1c	% CFB in weight; % CFB in SBP; HbA1c <7.0%; AEs; UTIs	Canagliflozin; dapagliflozin; empagliflozin	Some doses of ertugliflozin reported to be superior to specific comparators, but most non-significant	None	NMAs

For peer review only

Ertugliflozin with metformin and a dipeptidyl peptidase-4 inhibitor for treating type 2 diabetes (TA583)[5]	Type 2 diabetes	June 2019	% CFB in HbA1c	% CFB in weight; % CFB in SBP; HbA1c <7.0%; AEs; UTIs	Canagliflozin; dapagliflozin; empagliflozin	Some significant differences noted vs dapagliflozin when one trial considered but not in other analyses	None	NMAs
Risankizumab for treating moderate to severe plaque psoriasis (TA596)[6]	Plaque psoriasis	August 2019	PASI 90 response; PASI 75 response	PASI 50 response; PASI 100 response; DLQI 0/1; AEs; serious AEs; withdrawal due to AEs	Guselkumab	Likely all non-significant but unclear	Adalimumab (1 superiority trial); ustekinumab (2 superiority trials)	NMAs
Brolucizumab for treating wet age-related macular degeneration (TA672)[7]	Age-related macular degeneration	February 2021	Mean CFB in BCVA	CFB in injection frequency; CFB in CRT; gain ≥15 ETDRS letters; loss ≥15 ETDRS letters; AEs; treatment discontinuation	Ranibizumab	None	Aflibercept (2 non-inferiority trials)	NMAs
Acalabrutinib for treating chronic lymphocytic leukaemia (TA689)[8]	Chronic lymphocytic leukaemia	April 2021	OS; PFS	AEs	Ibrutinib	None	None	MAICs
Bimekizumab for treating moderate to severe plaque psoriasis (TA723)[9]	Plaque psoriasis	September 2021	PASI 90 response; PASI 75 response	PASI 50 response; PASI 100 response; AEs; serious AEs; AEs leading to discontinuation	Brodalumab; Risankizumab; ixekizumab	Possibly some statistically significant differences but unclear	Adalimumab (1 superiority trial); secukinumab (1 superiority trial); Ustekinumab (1 superiority trial)	NMAs
Secukinumab for treating moderate to severe plaque	Plaque psoriasis	October 2021	PASI 75 response	PASI 50 response; PASI 90 response;	Ustekinumab (adalimumab could not be	None	Etanercept (2 superiority trials)	NMAs

For peer review only

psoriasis in children and young people (TA734)[10]				PASI 100 response; mean CFB in CDLQI	included in the ITC)			
Tofacitinib for treating juvenile idiopathic arthritis (TA735)[11]	Juvenile idiopathic arthritis	October 2021	Disease flares; ACR Pedi responses (various thresholds)	URTI	Adalimumab; tocilizumab (etanercept could not be included in the ITC)	None	None	Bucher ITCs
Empagliflozin for treating chronic heart failure with reduced ejection fraction (TA773)[12]	Heart failure	March 2022	CV death or HHF composite	First HHF; total HHF; KCCQ; worsening renal function; CV death; all-cause mortality	Dapagliflozin	None	None	Bucher ITCs
Faricimab for treating diabetic macular oedema (TA799)[13]	Diabetic macular oedema	June 2022	Mean CFB in BCVA	Mean number of administration injections; CFB in CST; gain of ≥10/15 ETDRS letters; loss of ≥10/15 ETDRS letters; all-cause discontinuation; ocular AEs	Ranibizumab	Unclear – possibly statistically significant in overall population but not in ≥400 μm subgroup relevant to assessment	Aflibercept (4 non-inferiority trials)	NMAs
Faricimab for treating wet age-related macular degeneration (TA800)[14]	Age-related macular degeneration	June 2022	Mean CFB in BCVA	Mean number of injections; mean number of administration injections; mean CFB in CST; gain of ≥10/15 BCVA letters; ETDRS	Ranibizumab	Unclear	Aflibercept (4 non-inferiority trials)	NMAs

For peer review only

				letters categories; AEs; discontinuation				
Risankizumab for treating active psoriatic arthritis after inadequate response to DMARDs (TA803)[15]	Psoriatic arthritis	July 2022	ACR 20; PASI 75 response	PsARC response; ACR 50; ACR 70; PASI 50 response; PASI 90 response; PASI 100 response; CFB in HAQ-DI; AEs, serious AEs; AEs leading to discontinuation	Guselkumab	None	None	NMAs
Brolucizumab for treating diabetic macular oedema (TA820)[16]	Diabetic macular oedema	August 2022	CFB in BCVA	BCVA categorical analyses; CFB in DRSS; CFB in retinal thickness; study discontinuation; serious ocular AEs; serious non-ocular AEs	Ranibizumab	None when EAG-preferred ITCs considered (statistically significant for company's preferred analysis)	Aflibercept	NMAs
Upadacitinib for treating active ankylosing spondylitis (TA829)[17]	Ankylosing spondylitis	September 2022	ASAS 40; BASDAI 50	CFB in BASDAI; CFB in BASFI; ASAS 20; ASAS PR; CFB in total back pain score	Ixekizumab; secukinumab	None	None	NMAs
Palbociclib with fulvestrant for treating hormone receptor-positive, HER2-negative advanced breast cancer after endocrine therapy (TA836)[18]	Breast cancer	October 2022	OS; PFS	None	Abemaciclib + fulvestrant; ribociclib + fulvestrant	Unclear but likely non-significant	None	MAICs and Bucher ITCs also presented

For peer review only

Cabozantinib for previously treated advanced hepatocellular carcinoma (TA849)[19]	Hepatocellular carcinoma	December 2022	OS; PFS	Treatment-emergent AEs with a grade 3/4 occurring in ≥5% of patients in either arm	Regorafenib	None	None	Bucher ITCs and MAICs presented
Upadacitinib for treating active non-radiographic axial spondyloarthritis (TA861)[20]	Axial spondyloarthritis	February 2023	ASAS 40	BASDAI 50; CFB in BASDAI50; CFB in BASFI; ASAS 20; ASAS PR; patient assessment of CFB in total back pain	Secukinumab; ixekizumab	None	None	NMAs
Upadacitinib for previously treated moderately to severely active Crohn's disease (TA905)[21]	Crohn's disease	June 2023	CDAI clinical remission; CDAI clinical response	Serious AEs; discontinuation due to AEs	Ustekinumab; vedolizumab	Possibly statistically significant results for clinical remission outcomes but not clinical response outcomes, but unclear	None	NMAs
Bimekizumab for treating axial spondyloarthritis (TA918)[22]	Axial spondyloarthritis	October 2023	ASAS 40; BASDAI 50	ASAS 20; ASAS S5/6; ASAS PR; ASDAS <2.1; ASDAS-CII; ASDAS ID; ASDAS MI; BASDAI 50; ASDAS-CRP; ASQoL; BASDAI; BASFI; BASMI; fatigue NRS; MASES; NSP; PhGADA; SF-36 MCS; SF-36 PCS; all-cause	Secukinumab; ixekizumab	None	None	NMAs

For peer review only

				discontinuation; discontinuation due to AEs; serious AEs				
Tofacitinib for treating active ankylosing spondylitis (TA920)[23]	Ankylosing spondylitis	October 2023	ASAS 20	ASAS 40; BASDAI 50; BASDAI; BASFI; ASDAS; ASQoL; SF-36 PCS; AE-related discontinuation	Secukinumab; ixekizumab	None	None	NMAs
Mirikizumab for treating moderately to severely active ulcerative colitis (TA925)[24]	Ulcerative colitis	October 2023	Clinical response; clinical remission	Mucosal healing; all-cause discontinuation; serious AEs	Vedolizumab; ustekinumab	Possibly some statistically significant results for certain populations but most likely non-significant, unclear	None	NMAs
Empagliflozin for treating chronic heart failure with preserved or mildly reduced ejection fraction (TA929)[25]	Heart failure	November 2023	CV mortality or HHF composite	HHF; CV mortality; all-cause mortality	Dapagliflozin	None	None	Bucher ITCs
Zanubrutinib for treating chronic lymphocytic leukaemia (TA931)[26]	Chronic lymphocytic leukaemia	November 2023	OS; PFS	None	Acalabrutinib; VenR (ibrutinib was naïve comparison)	None	None	MAIC (acalabrutinib) and NMA (VenR, performed by EAG)
Empagliflozin for treating chronic kidney disease (TA942)[27]	Chronic kidney disease	December 2023	Composite renal outcome (using various thresholds)	Progression to ESKD/ESRD; HHF; CV death; HHF or CV death	Dapagliflozin	None	None	NMAs and MAICs

For peer review only

				composite; 3P-MACE+; all-cause mortality; all-cause hospitalisations				
Fluocinolone acetonide intravitreal implant for treating chronic diabetic macular oedema (TA953)[28]	Chronic diabetic macular oedema	March 2024	≥15-letter BCVA improvement	Mean CFB in BCVA letter score; mean CFB in CRT; serious ocular AEs; intraocular pressure-related AEs; cataract-related AEs	Dexamethasone intravitreal implant	None	None	Bucher ITCs and MAICs
Etrasimod for treating moderately to severely active ulcerative colitis in people aged 16 and over (TA956)[29]	Ulcerative colitis	March 2024	Clinical response; clinical remission	Serious infections during induction phase of treatment	Adalimumab, infliximab; vedolizumab (others listed but not focused on)	Statistically significant for comparison against adalimumab but not infliximab or vedolizumab	None	NMAs
Linzagolix for treating moderate to severe symptoms of uterine fibroids (TA996)[30]	Uterine fibroids	August 2024	Response (MBL ≤80 ml and ≥50% reduction from baseline)	% change in MBL; pain improvement; % change in primary fibroid volume; % change in haemoglobin; CFB in HRQoL	Relugolix CT	None	None	NMAs
Risankizumab for treating moderately to severely active ulcerative colitis (TA998)[31]	Ulcerative colitis	August 2024	Clinical response; clinical remission	Endoscopic improvement; serious infections; serious AEs	Ustekinumab	None	None	NMAs
Vibegron for treating symptoms	Overactive bladder syndrome	September 2024	Average number of daily	Total incidence of incontinence	Mirabegron	One statistically significant result	None	NMAs and Bucher ITCs

For peer review only

of overactive bladder syndrome (TA999)[32]			micturitions; number of UUI episodes	episodes; volume of urine per micturition; any AEs; serious AEs; AEs leading to study discontinuation; specific AEs (headache, hypertension, UTI, dry mouth and constipation)		for one comparison/time-point for number of UUI episodes but all others non-significant		
Faricimab for treating visual impairment caused by macular oedema after retinal vein occlusion (TA1004)[33]	Visual impairment caused by macular oedema after retinal vein occlusion	September 2024	Mean CFB in BCVA	Mean CFB in CST; categorical vision changes; serious ocular AEs; all-cause discontinuation	Ranibizumab	None	None	NMAs
Rucaparib for maintenance treatment of relapsed platinum-sensitive ovarian, fallopian tube or peritoneal cancer (TA1007)[34]	Ovarian, fallopian tube and peritoneal cancer	September 2024	PFS; OS	PFS for second event; time to start of subsequent therapy; time to treatment discontinuation	Niraparib; olaparib	Unclear, likely non-significant	None	NMAs and MAICs
Crovalimab for treating paroxysmal nocturnal haemoglobinuria in people 12 years and over (TA1019)[35]	Paroxysmal nocturnal haemoglobinuria	November 2024	Transfusion avoidance; breakthrough haemolysis	Haemoglobin stabilisation; number of packed red blood cell transfusions; FACIT fatigue score; AEs	Ravulizumab	None	Eculizumab (1 non-inferiority, 1 safety/tolerability study)	NMAs

For peer review only

Eplontersen for treating hereditary transthyretin-related amyloidosis (TA1020)[36]	Hereditary transthyretin-related amyloidosis	November 2024	Serum TTR % CFB; CFB in mNIS+7; CFB in Norfolk QoL-DN	Serum TTR absolute value and absolute CFB; response analyses for mNIS+7; responder analyses for Norfolk QoL-DN; CFB in mBMI; CFB in 10-MWT; CFB in R-ODS; serious AEs; severe AEs; treatment discontinuation	Vutrisiran	None	None	MAICs
Crizotinib for treating ROS1-positive advanced non-small-cell lung cancer (TA1021)[37]	Non-small-cell lung cancer	December 2024	OS; PFS; ORR	Duration of response	Entrectinib	Unclear but likely mostly non-significant	None	STCs and MAICs
Bevacizumab gamma for treating wet age-related macular degeneration (TA1022)[38]	Age-related macular degeneration	December 2024	Mean CFB in BCVA	Gain ≥15 BCVA letters; loss ≥15 BCVA letters; ocular AEs	Aflibercept; faricimab	None	Ranibizumab (2 superiority trials)	NMAs and MAICs
Ublituximab for treating relapsing multiple sclerosis (TA1025)[39]	Multiple sclerosis	December 2024	ARR	CDP-12; CDP-24; all-cause discontinuation	Ocrelizumab; ofatumumab	None	Teriflunomide (2 superiority trials)	NMAs
Olaparib for treating BRCA mutation-positive HER2-negative advanced breast	Breast cancer	February 2025	PFS; OS	ORR; any AEs; any serious AEs; any treatment-related serious AEs;	Talazoparib	None	None	NMAs

For peer review only

cancer after chemotherapy (TA1040)[40]				specific types of AEs				
Durvalumab with etoposide and either carboplatin or cisplatin for untreated extensive-stage small-cell lung cancer (TA1041)[41]	Small-cell lung cancer	February 2025	OS; PFS	Any AEs; specific AE types/severities	Atezolizumab	None	None	Bucher ITCs

Abbreviations: 3P-MACE, 3-point major adverse cardiovascular outcome; 10-MWT, 10-metre walk test; ACR, American College of Rheumatology; ACR Pedi, American College of Rheumatology Pediatric response criteria; AE, adverse event; ARR, annualised relapse rate; ASAS, Assessment of SpondyloArthritis international Society; ASAS-PR, Assessment of SpondyloArthritis International Society criteria partial remission; ASDAS, Ankylosing Spondylitis Disease Activity Score; ASDAS-CII, Ankylosing Spondylitis Disease Activity Score Clinically Important Improvement; ASDAS-CRP, Ankylosing Spondylitis Disease Activity Score with C-reactive protein; ASDAS-ID, Ankylosing Spondylitis Disease Activity Score Inactive Disease; ASDAS-MI, Ankylosing Spondylitis Disease Activity Score Major Improvement; ASQoL, Ankylosing Spondylitis Quality of Life; BASDAI, Bath Ankylosing Spondylitis Disease Activity Index; BASFI, Bath Ankylosing Spondylitis Functional Index; BASMI, Bath Ankylosing Spondylitis Metrology Index; BCVA, best corrected visual acuity; CFB, change from baseline; CDAI, Clinical Disease Activity Index; CDLQI, Children's Dermatology Life Quality Index; CDP, confirmed disability progression; CRT, central retinal thickness; CST, central subfield thickness; CV, cardiovascular; DLQI, Dermatology Life Quality Index; DRSS, Diabetic Retinopathy Severity Scale; EAG, External Assessment Group; ESKD/ESRD, end-stage kidney/renal disease; ETDRS, Early Treatment Diabetic Retinopathy Study; FACIT, Functional Assessment of Chronic Illness Therapy; HAQ-DI, Health Assessment Questionnaire Disability Index; HbA1c, haemoglobin A1C; HHF, hospitalisation for heart failure; HRQoL, health-related quality of life; ITC, indirect treatment comparison; KCCQ, Kansas City Cardiomyopathy Questionnaire; MAIC, matching-adjusted indirect comparison; MASES, Maastricht Ankylosing Spondylitis Enthesitis; MBL, menstrual blood loss; mBMI, modified body mass index; mNIS+7, Modified Neuropathy Impairment Score +7; NICE, National Institute for Health and Care Excellence; NMA, network-meta-analysis; NRS, numerical rating scale; Norfolk QoL-DN, Norfolk Quality of Life-Diabetic Neuropathy; NSP, nocturnal spine pain; ORR, overall response rate; OS, overall survival; PASI, Psoriasis Area and Severity Index; PCS, physical component summary; PFS, progression-free survival; PGA/IGA, Physician's Global Assessment Scale/Investigator's Global Assessment Scale; PhGADA, Physician's Global Assessment of Disease Activity; PsARC, Psoriatic Arthritis Response Criteria; R-ODS, Rasch-built Overall Disability Scale; SBP, systolic blood pressure; SF-36, Short Form 36-Item Health Survey; SLR, systematic literature review; STC, simulated treatment comparison; TTR, transthyretin; URTI, upper respiratory tract infection; UTI urinary tract infection; VenR, Venetoclax-Rituximab; UUI, urgency urinary incontinence.

Supplementary Table 2. Appraisals excluded from the SLR

Appraisal (ID)	Reason for exclusion
Guidance on the use of capecitabine and tegafur with uracil for metastatic colorectal cancer (TA61)[42]	No ITCs performed
Frequency of application of topical corticosteroids for atopic eczema (TA81)[43]	No ITCs performed
Topotecan for the treatment of relapsed small-cell lung cancer (TA184)[44]	No ITCs performed
Capecitabine for the treatment of advanced gastric cancer (TA191)[45]	No ITCs performed
Dexamethasone intravitreal implant for the treatment of macular oedema secondary to retinal vein occlusion (TA229)[46]	Deprioritised due to time constraints (not a cost-comparison evaluation from the start)
Colistimethate sodium and tobramycin dry powders for inhalation for treating pseudomonas lung infection in cystic fibrosis (TA276)[47]	ITCs performed did not provide evidence for key comparators missing from direct trial evidence and were not used by committee in decision-making
Ciclosporin for treating dry eye disease that has not improved despite treatment with artificial tears (TA369)[48]	No ITCs performed
Dasatinib, nilotinib and high-dose imatinib for treating imatinib-resistant or intolerant chronic myeloid leukaemia (TA425)[49]	No ITCs performed
Dasatinib, nilotinib and imatinib for untreated chronic myeloid leukaemia (TA426)[50]	Deprioritised due to time constraints (not a cost-comparison evaluation from the start)
Everolimus for advanced renal cell carcinoma after previous treatment (TA432)[51]	No ITCs performed
Ustekinumab for moderately to severely active Crohn's disease after previous treatment (TA456)[52]	Deprioritised due to time constraints as cost-comparison evaluation was only a scenario in the initial company submission rather than the base case analysis
Atezolizumab for treating locally advanced or metastatic non-small-cell lung cancer after chemotherapy (TA520)[53]	Deprioritised due to time constraints (not a cost-comparison evaluation from the start)
Pembrolizumab for treating relapsed or refractory classical Hodgkin lymphoma (TA540)[54]	No cost-comparison assessment included
Venetoclax with rituximab for previously treated chronic lymphocytic leukaemia (TA561)[55]	Deprioritised due to time constraints (not a cost-comparison evaluation from the start)
Encorafenib with binimetinib for unresectable or metastatic BRAF V600 mutation-positive melanoma (TA562)[56]	Committee decision-making not made solely on the basis of a cost-comparison assessment and was not a cost-comparison appraisal from the outset
Abemaciclib with an aromatase inhibitor for previously untreated, hormone receptor-positive, HER2-negative, locally advanced or metastatic breast cancer (TA563)[57]	Deprioritised due to time constraints (not a cost-comparison evaluation from the start)
Benralizumab for treating severe eosinophilic asthma (TA565)[58]	Deprioritised due to time constraints (not a cost-comparison evaluation from the start)

For peer review only - http://bmjopen.bmj.com/site/about/guidelines.xhtml

Ustekinumab for treating moderately to severely active ulcerative colitis (TA633)[59]	Deprioritised due to time constraints (not a cost-comparison evaluation from the start)
Brigatinib for ALK-positive advanced non-small-cell lung cancer that has not been previously treated with an ALK inhibitor (TA670)[60]	Deprioritised due to time constraints as cost-comparison evaluation was only a scenario in the initial company submission rather than the base case analysis
Mepolizumab for treating severe eosinophilic asthma (TA671)[61]	ITC results for outcomes considered to be primary outcomes were statistically significant
Anakinra for treating Still's disease (TA685)[62]	Deprioritised due to time constraints (not a cost-comparison evaluation from the start)
Selective internal radiation therapies for treating hepatocellular carcinoma (TA688)[63]	No ITCs performed
Atezolizumab monotherapy for untreated advanced non-small-cell lung cancer (TA705)[64]	Deprioritised due to time constraints as cost-comparison evaluation was only a scenario in the initial company submission rather than the base case analysis
Diroximel fumarate for treating relapsing–remitting multiple sclerosis (TA794)[65]	Direct comparative evidence available for all relevant comparators
Somatrogon for treating growth disturbance in children and young people aged 3 years and over (TA863)[66]	Direct comparative evidence available for all relevant comparators
Vutrisiran for treating hereditary transthyretin-related amyloidosis (TA868)[67]	Direct comparative evidence available for all relevant comparators
Eptinezumab for preventing migraine (TA871)[68]	Deprioritised due to time constraints as cost-comparison evaluation was only a scenario in the initial company submission rather than the base case analysis
Risankizumab for previously treated moderately to severely active Crohn's disease (TA888)[69]	Deprioritised due to time constraints (not a cost-comparison evaluation from the start)
Pegunigalsidase alfa for treating Fabry disease (TA915)[70]	No ITCs performed
Bimekizumab for treating active psoriatic arthritis (TA916)[71]	ITC results for outcomes considered to be primary outcomes were statistically significant
Momelotinib for treating myelofibrosis-related splenomegaly or symptoms (TA957)[72]	Direct comparative evidence available for all relevant comparators
Atogepant for preventing migraine (TA973)[73]	No papers or discussion relating to CCE stage prior to scrutiny decision to reject CCE available
Selective internal radiation therapy with QuiremSpheres for treating unresectable advanced hepatocellular carcinoma (TA985)[74]	Naïve comparisons performed rather than formal indirect treatment comparison methods
Tenecteplase for treating acute ischaemic stroke (TA990)[75]	Direct comparative evidence available for all relevant comparators
Trastuzumab deruxtecan for treating HER2-low metastatic or unresectable breast cancer after chemotherapy (TA992)[76]	No ITCs performed
Pembrolizumab with platinum- and fluoropyrimidine-based chemotherapy for untreated advanced HER2-negative gastric or gastro-oesophageal junction adenocarcinoma (TA997)[77]	Deprioritised due to time constraints (not a cost-comparison evaluation from the start)

Evinacumab for treating homozygous familial hypercholesterolaemia in people 12 years and over (TA1002)[78]	Deprioritised due to time constraints (not a cost-comparison evaluation from the start)
Futibatinib for previously treated advanced cholangiocarcinoma with FGFR2 fusion or rearrangement (TA1005)[79]	Committee decision-making not made on the basis of a cost-comparison assessment and was not a cost-comparison appraisal from the outset
Latanoprost–netarsudil for previously treated primary open-angle glaucoma or ocular hypertension (TA1009)[80]	Deprioritised due to time constraints (not a cost-comparison evaluation from the start)
Fenfluramine for treating seizures associated with Lennox–Gastaut syndrome in people 2 years and over (TA1050)[81]	Deprioritised due to time constraints (not a cost-comparison evaluation from the start)
Tislelizumab for treating unresectable advanced oesophageal squamous cell cancer after platinum-based chemotherapy (ID4070)[82]	Appraisal in progress and no information obtained from NICE to allow inclusion in review at this stage
Somapacitan for treating growth hormone deficiency in children (ID6178)[83]	Appraisal in progress and no information obtained from NICE to allow inclusion in review at this stage
Guselkumab for treating moderately to severely active ulcerative colitis (ID6237)[84]	Appraisal will not complete before end of this review
Guselkumab for previously treated moderately to severely active Crohn's disease (ID6238)[85]	Appraisal in progress and no information obtained from NICE to allow inclusion in review at this stage
Mirikizumab for treating moderately to severely active Crohn's disease (ID6244)[86]	Appraisal in progress and no information obtained from NICE to allow inclusion in review at this stage
Nivolumab as neoadjuvant (with chemotherapy) and adjuvant (as monotherapy) treatment for resectable non-small-cell lung cancer (ID6310)[87]	Confirmed by NICE that documents would not be available before end of the review
Vanzacaftor–tezacaftor–deutivacaftor for treating cystic fibrosis with 1 or more F508del mutations in the CFTR gene in people aged 6 years and over (ID6372)[88]	Appraisal will not complete before end of this review
Abiraterone (originator and generics) for treating newly diagnosed high-risk hormone-sensitive metastatic prostate cancer (review of TA721) (ID6378)[89]	Appraisal not yet in development stage and will not complete before end of this review
Rilzabrutinib for treating persistent or chronic immune thrombocytopenia in people aged 12 and over (ID6395)[90]	Appraisal not yet in development stage and will not complete before end of this review
Darolutamide with androgen deprivation therapy for treating hormone-sensitive metastatic prostate cancer (ID6452)[91]	Confirmed by NICE that documents would not be available before end of the review
Abbreviations: CCE, cost-comparison evaluation; ITC, indirect treatment comparison; NICE, National Institute for Health and Care Excellence; SLR, systematic literature review.	

Supplementary Table 3. Summary of methods used by companies to demonstrate clinical similarity

Method	Details	Appraisals
**Interpreting ITC results**		
Lack of statistically significant differences only	Relies solely on a lack of statistically significant differences only to interpret ITC results and conclude similarity.	**Three:** TA735, TA829 and TA1040.[11, 17, 40]
Lack of statistically significant differences and some discussion of point estimate direction and/or size	Relies mostly on a lack of statistically significant differences and direction and/or size of point estimates to interpret ITC results and conclude similarity.<br><br>Point estimates favouring the intervention over comparators are often noted, with those discussing point estimate size commenting on the difference being small and close to 0.0 or 1.0 (line of null effect).<br><br>Some refer to the fact that point estimates vary in direction for different outcomes, suggesting no consistent trend for a benefit of one treatment or the other as evidence of no difference.	**Fourteen:** TA689, TA773, TA849, TA861, TA929, TA931, TA942, TA953, TA998, TA1007, TA1020, TA1021, TA1025, TA1041[8, 19, 20, 25-28, 31, 34, 36, 37, 39, 41, 92]
Some statistically significant differences but none favouring the comparators	Some analyses with statistically significant differences are noted but none of these favour the comparator(s).	**Eight:** TA521, TA572, TA583, TA672, TA799, TA836, TA918, TA925[3-5, 7, 13, 18, 22, 24]
Size and direction of point estimates mentioned with no mention of lack of statistically significant differences	Size and direction of point estimates used to make conclusions of clinical similarity, with no emphasis placed on the lack of statistically significant differences.	**One:** TA803[15]
Some use of probabilities calculated from NMAs to interpret results	NMAs used to calculate probabilities of being best treatment, among best treatments, clinically equivalent or non-inferior, for example based on SUCRA values or incorporating equivalence or non-inferiority margins.<br><br>Often used alongside other methods of interpreting results such as highlighting a lack of statistically significant differences, point estimates close to 1.0 or 0.0 or point estimates favouring the intervention over the comparator(s).<br><br>In some cases they are mentioned but not focused on or are dismissed if not favouring the intervention.	**Six:** TA734, TA803, TA820, TA996, TA1019, TA1022[10, 15, 16, 30, 35, 38]

For peer review only

MCIDs or other thresholds used to support with interpretation of results	MCIDs or other thresholds used by the company in some way to interpret ITC results and conclude clinical similarity.<br><br>Often used alongside other methods of interpreting results such as highlighting a lack of statistically significant differences, point estimates close to 1.0 or 0.0 or point estimates favouring the intervention over the comparator(s).	**Four:** TA486, TA803, TA999, TA1019[1, 15, 32, 35]
Unclear what focus of decision-making was	Rationale for concluding similarity was unclear either because results and discussion sections were heavily redacted or because the rationale for conclusions of clinical similarity were not described.	**Ten:** TA497, TA596, TA723, TA734, TA800, TA820, TA905, TA920, TA956, TA1004[2, 6, 9, 10, 14, 16, 21, 23, 29, 33]
**Supplementary evidence**		
Similar mechanisms of action or pharmacodynamic profiles noted	Similarity in terms of mechanisms of action or pharmacodynamic profiles noted by the company as supportive evidence that the intervention is expected to be similar to the comparator(s).	**Six:** TA486, TA572, TA689, TA773, TA953, TA998[1, 4, 8, 28, 31, 92]
Clinical expert feedback	Clinical expert feedback stating that the treatments are expected to or have similar efficacy in their experience or clinical expert interpretation of ITC results as likely similar.	**Ten:** TA689, TA773, TA803, TA849, TA861, TA931, TA942, TA996, TA1040, TA1041[8, 15, 19, 20, 26, 27, 30, 40, 41, 92]
Reference to meta-analyses of intervention and comparator trials vs a common comparator as evidence of a class effect	Meta-analyses of comparator and intervention trials against a common comparator cited as evidence that there is a class effect, suggesting clinical similarity should be concluded.	**Three:** TA773, TA836, TA942[18, 27, 92]
Reference to previously published ITC results or NICE appraisals	Reference to other ITCs of the intervention and comparator(s) relevant to the appraisal within the literature or previous NICE appraisals as supportive evidence of clinical similarity given similar results were obtained.	**Four:** TA803, TA836, TA849, TA861[15, 18-20]
Reference to real-world data as supportive evidence	Reference to real-world data used as supportive evidence to ITCs evidence	**One:** TA1007[34]
Abbreviations: ITC, indirect treatment comparison; MCID, minimal clinically important difference; NICE, National Institute for Health and Care Excellence; NMA, network meta-analyses; SUCRA, Surface Under the Cumulative RAnking curve.		

113

For peer review only

Supplementary Table 4. Summary of methods used or comments made by EAGs in assessing clinical similarity

Method/comment	Details	Number of appraisals
**Interpretation of lack of statistically significant differences**		
Lack of statistically significant differences with some point estimates favouring the intervention used to make conclusions	Discussion of results and conclusions of clinical similarity focuses on the lack of statistically significant differences as well as at least some point estimates that favour the new intervention.	**Three:** TA829, TA953, TA1004[17, 28, 33]
Lack of statistically significant differences not completely dismissed but other methods of interpreting ITC results considered in making conclusions	Does not reject the use of lack of statistically significant differences as a method of making conclusions completely but incorporates other evidence in the decision-making, such as visual comparisons of adjusted KM curves, clinical expert feedback, similar mechanisms of action, decisions made in previous appraisals or additional data or analyses requested from the company or performed by the EAG itself.<br><br>While lack of statistically significant differences not rejected outright, EAGs sometimes highlight that it should be interpreted as no evidence of a difference between treatments rather than clinical similarity or equivalence conclusively.	**Twelve:** TA486, TA497, TA583, TA596, TA689, TA735, TA918, TA929, TA942, TA1007, TA1019, TA1041[1, 2, 5, 6, 8, 11, 22, 25, 27, 34, 35, 41]
Dismisses a lack of statistically significant differences as evidence for clinical similarity or equivalence	EAGs explicitly dismissed the idea that a lack of statistically significant differences is sufficient to conclude clinical similarity or equivalence, or it was otherwise suggested based on uncertainties discussed or requests made to the company.<br>In many cases this contributed to conclusions made by the EAG that there was insufficient evidence to support a CCE, but in some cases these uncertainties were addressed by consideration of other evidence and clinical similarity was concluded overall. For some appraisals, rejection of a CCE was likely not solely based on the lack of statistically significant differences and other factors such as concerns about trials included in the ITC or methodology may have contributed to this conclusion.	**Nineteen:** TA521, TA773, TA803, TA820, TA836, TA849, TA861, TA905, TA920, TA925, TA931, TA956, TA996, TA998, TA999, TA1020, TA1021, TA1025, TA1040[3, 15, 16, 18-21, 23, 24, 26, 29-32, 36, 37, 39, 40, 92]
Main rationale for conclusions of clinical similarity and position on lack of statistically significant differences unclear	EAG concludes that clinical similarity is likely to be a reasonable conclusion but the main factors for decision-making are unclear and there is no clear statement on the EAG's position regarding lack of statistically significant differences.	**Five:** TA572, TA672, TA723, TA800, TA1022[4, 7, 9, 14, 38]
**Formal methods of interpreting ITC results**		

For peer review only

Some use of probabilities calculated from NMAs to interpret results	NMAs used to calculate probabilities of being the best treatment, among the best treatments, clinically equivalent or non-inferior; for example, based on SUCRA values or incorporating equivalence or non-inferiority margins.<br><br>Reference to these data provided by companies was made to aid with the EAG's interpretation of results, there was a suggestion that these would have been useful if not provided or there was no criticism of the company's use of these data	**Nine:** TA497, TA734, TA803, TA820, TA996, TA1019, TA1022, TA1025, TA1040[2, 10, 15, 16, 30, 35, 38-40]
MCIDs or other thresholds used to support with interpretation of results	MCIDs or other thresholds used by the EAG in some way to interpret ITC results and conclude clinical similarity or mentioned as being potentially useful.<br><br>Based on the EAG making use of these put forward by the company or adapted versions, the EAG putting forward this approach itself or no criticism of the method having been used by the company	**Nine:** TA486, TA497, TA803, TA931, TA996, TA999, TA1019, TA1020, TA1040 [1, 2, 15, 26, 30, 32, 35, 36, 40]
**Supplementary evidence**		
Similar mechanisms of action or pharmacodynamic profiles noted	Similarity in terms of mechanisms of action or pharmacodynamic profiles noted by the EAG as supportive evidence that the intervention is expected to be similar to the comparator(s).	**Fourteen:** TA486, TA497, TA572, TA596, TA836, TA918, TA929, TA996, TA998, TA999, TA1007, TA1022, TA1025, TA1040[1, 2, 4, 6, 18, 22, 25, 30-32, 34, 38-40]
Clinical expert feedback	Clinical expert feedback stating that the treatments are expected to or have similar efficacy in their experience or clinical expert input to support with interpretation of ITC results, such as identification of MCIDs	**Fourteen:** TA497, TA689, TA723, TA836, TA929, TA931, TA956, TA998, TA999, TA1007, TA1020, TA1022, TA1025, TA1040[2, 8, 9, 18, 26, 29, 31, 32, 34, 36, 38-40, 93]
Other sources noted	Other sources of evidence used as supportive evidence of clinical similarity, including results of ITCs from previous NICE appraisals, statistical significance testing requested from companies, results from amended analyses to better align trials, visual review of adjusted KM curves from ITCs and naïve comparison of data.<br><br>Typically rejected meta-analyses of interventions and comparators to demonstrate class effect as supportive of clinical similarity when put forward by the company.	**Fourteen:** TA486, TA497, TA572, TA583, TA723, TA735, TA773, TA836, TA918, TA929, TA942, TA1007, TA1021, TA1041[1, 2, 4, 5, 9, 11, 18, 22, 25, 27, 34, 37, 41, 92]

For peer review only

Abbreviations: CCE, cost-comparison evaluation; EAG, External Assessment Group; ITC, indirect treatment comparison; KM, Kaplan-Meier; MCID, minimal clinically important difference; NICE, National Institute for Health and Care Excellence; NMA, network meta-analyses; SUCRA, Surface Under the Cumulative RAnking curve.		

Supplementary Table 5. Summary of comments made by committees when making conclusions of clinical similarity.

Comment	Details	Number of appraisals
Discussion very limited, simply makes conclusions of clinical similarity	Only brief statement that results from ITCs were considered to indicate that intervention is likely to be similar to comparator(s), with no rationale or comments on uncertainty mentioned.	**Twenty-four:** TA572, TA583, TA596, TA672, TA723, TA734, TA735, TA800, TA836, TA905, TA918, TA925, TA942, TA953, TA998, TA999, TA1007, TA1019, TA1020, TA1021, TA1022, TA1025, TA1040, TA1041[4-7, 9-11, 14, 18, 21, 22, 24, 27, 28, 31, 32, 34-41]
Concludes similarity with uncertainty mentioned but no rationale as to how this was resolved	Notes uncertainties in ITC results but concludes clinical similarity likely overall with no discussion of how this uncertainty was addressed, why it was not considered a major issue or mention of other information that may have reduced uncertainty.	**Four:** TA521, TA799, TA920, TA929[3, 13, 23, 25]
Notes uncertainty in ITC results with clinical expert feedback considered before concluding clinical similarity	Uncertainty in ITC results noted but clinical expert feedback appears to have reduced concerns about this uncertainty to allow a conclusion of clinical similarity to be made.	**Seven:** TA486, TA689, TA820, TA829, TA849, TA861, TA905[1, 8, 16, 17, 19-21]
Notes uncertainty in ITC results with similarities in mechanisms of action considered before concluding clinical similarity	Uncertainty in ITC results noted but similarities in mechanisms of action may have reduced concerns about this uncertainty to allow a conclusion of clinical similarity to be made.	**Three:** TA773, TA803, TA956[9, 15, 29]
Notes uncertainty in ITC results but robustness of results to alternative analyses considered before concluding clinical similarity	Uncertainty in ITC results noted but the fact that results were robust to alternative analyses seems to have reduced some of the concern to allow a conclusion of clinical similarity to be made.	**One:** TA497[2]

For peer review only

Notes uncertainty in ITC results but point estimates being close to 1.0 or 0.0 considered in decision-making	Uncertainty in ITC results noted but the fact that point estimates were close to 1.0 or 0.0 seems to have reduced some of the concern to allow a conclusion of clinical similarity to be made.	**One:** TA803[15]
Committee concluded that results are too uncertain and cost-utility analyses required, or cost-utility analyses used despite conclusions of similarity from ITC results	Conclusion that there was too much uncertainty in the ITC results and cost-utility analyses were required for decision-making, or a conclusion of clinical similarity was made based on ITC results, but cost-utility analyses still appear to have been used for decision-making.	**Three:** TA849, TA931, TA996[19, 26, 30]
Cost-utility results available alongside cost-comparison analyses which may have contributed to decision-making	Conclusion of clinical similarity made but some cost-utility analyses were available alongside cost-comparison analyses, which may have reduced uncertainty in decision-making.	**One:** TA773[92]
Abbreviations: ITC, indirect treatment comparison.		

For peer review only

Supplementary Table 6. Additional findings from the SLR

Finding	Details
Additional detail regarding the committee's interpretation of statistically non-significant ITC results and consideration of supportive evidence may improve the transparency of decision-making.	In some appraisals, EAGs rejected the CCE given the uncertainty in the conclusions of clinical similarity. In some cases, the committee subsequently determined that there was sufficient evidence of clinical similarity.<br>In these cases, it was clear from most committee discussions that supportive evidence, such as clinical expert feedback, may have supported this decision, but there was rarely an explicit discussion of why these were considered to sufficiently alleviate the uncertainties raised by the EAG.
Guidance on whether or not a CCE within the NICE process needs to present a case against multiple or only one relevant comparator would be helpful, as there is clearly some uncertainty about this	EAG and committee discussions in some appraisals in this SLR suggest that demonstrating clinical similarity compared to one relevant comparator may be sufficient, including statements made by the committee in NICE TA1022 and by the EAG in NICE TA1007.[34, 38] In CCEs with more than one comparator, EAGs and committees have generally discussed the evidence for clinical similarity for all comparators separately rather than focusing on one with the strongest evidence.<br>A clear statement supporting the requirement for a case against only one comparator was not identified in the current NICE methods manual,[94] and an article by Costello Medical has recently expressed uncertainty with regards to this.[95] Therefore, clarity surrounding this requirement within NICE CCEs in the form of updated guidance may be useful for companies and EAGs.
Abbreviations: CCE, cost-comparison evaluation; EAG, External Assessment Group; ITC, indirect treatment comparison; NICE, National Institute for Health and Care Excellence; SLR, systematic literature review.	

References

For peer review only

1. National Institute for Health and Care Excellence (NICE). Aflibercept for treating choroidal neovascularisation - Technology appraisal guidance [TA486], 2017. Available from: https://www.nice.org.uk/guidance/ta486. Date accessed: May 25.

2. National Institute for Health and Care Excellence (NICE). Golimumab for treating non-radiographic axial spondyloarthritis - Technology appraisal guidance [TA497] 2018. Available from: https://www.nice.org.uk/guidance/ta497. Date accessed: May 25.

3. National Institute for Health and Care Excellence (NICE). Guselkumab for treating moderate to severe plaque psoriasis - Technology appraisal guidance [TA521], 2018. Available from: https://www.nice.org.uk/guidance/ta521. Date accessed: Apr 25.

4. National Institute for Health and Care Excellence (NICE). Ertugliflozin as monotherapy or with metformin for treating type 2 diabetes - Technology appraisal guidance [TA572], 2019. Available from: https://www.nice.org.uk/guidance/ta572. Date accessed: May 25.

5. National Institute for Health and Care Excellence (NICE). Ertugliflozin with metformin and a dipeptidyl peptidase-4 inhibitor for treating type 2 diabetes - Technology appraisal guidance [TA583], 2019. Available from: https://www.nice.org.uk/guidance/ta583. Date accessed: May 25.

6. National Institute for Health and Care Excellence (NICE). Risankizumab for treating moderate to severe plaque psoriasis - Technology appraisal guidance [TA596], 2019. Available from: https://www.nice.org.uk/guidance/ta596. Date accessed: Apr 25.

7. National Institute for Health and Care Excellence (NICE). Brolucizumab for treating wet age-related macular degeneration - Technology appraisal guidance [TA672], 2021. Available from: https://www.nice.org.uk/guidance/ta672. Date accessed: Apr 25.

8. National Institute for Health and Care Excellence (NICE). Acalabrutinib for treating chronic lymphocytic leukaemia - Technology appraisal guidance [TA689], 2021. Available from: https://www.nice.org.uk/guidance/ta689. Date accessed: Apr 25.

9. National Institute for Health and Care Excellence (NICE). Bimekizumab for treating moderate to severe plaque psoriasis - Technology appraisal guidance [TA723], 2021. Available from: https://www.nice.org.uk/guidance/ta723. Date accessed: Apr 25.

10. National Institute for Health and Care Excellence (NICE). Secukinumab for treating moderate to severe plaque psoriasis in children and young people - Technology appraisal guidance [TA734], 2021. Available from: https://www.nice.org.uk/guidance/ta734. Date accessed: Apr 25.

11. National Institute for Health and Care Excellence (NICE). Tofacitinib for treating juvenile idiopathic arthritis - Technology appraisal guidance [TA735], 2021. Available from: https://www.nice.org.uk/guidance/ta735. Date accessed: Apr 25.

12. National Institute for Health and Care Excellence (NICE). Empagliflozin for treating chronic heart failure with reduced ejection fraction - Technology appraisal guidance [TA773] - Committee papers, 2022. Available from: https://www.nice.org.uk/guidance/ta773/history. Date accessed: May 25.

13. National Institute for Health and Care Excellence (NICE). Faricimab for treating diabetic macular oedema - Technology appraisal guidance [TA799], 2022. Available from: https://www.nice.org.uk/guidance/ta799. Date accessed: Apr 25.

For peer review only

14. National Institute for Health and Care Excellence (NICE). Faricimab for treating wet age-related macular degeneration - Technology appraisal guidance [TA800], 2022. Available from: https://www.nice.org.uk/guidance/ta800. Date accessed: Apr 25.

15. National Institute for Health and Care Excellence (NICE). Risankizumab for treating active psoriatic arthritis after inadequate response to DMARDs - Technology appraisal guidance [TA803], 2022. Available from: https://www.nice.org.uk/guidance/ta803. Date accessed: Apr 25.

16. National Institute for Health and Care Excellence (NICE). Brolucizumab for treating diabetic macular oedema - Technology appraisal guidance [TA820], 2022. Available from: https://www.nice.org.uk/guidance/ta820. Date accessed: Apr 25.

17. National Institute for Health and Care Excellence (NICE). Upadacitinib for treating active ankylosing spondylitis - Technology appraisal guidance [TA829], 2022. Available from: https://www.nice.org.uk/guidance/ta829. Date accessed: Apr 25.

18. National Institute for Health and Care Excellence (NICE). Palbociclib with fulvestrant for treating hormone receptor-positive, HER2-negative advanced breast cancer after endocrine therapy - Technology appraisal guidance [TA836], 2022. Available from: https://www.nice.org.uk/guidance/ta836. Date accessed: Apr 25.

19. National Institute for Health and Care Excellence (NICE). Cabozantinib for previously treated advanced hepatocellular carcinoma - Technology appraisal giudance [TA849], 2022. Available from: https://www.nice.org.uk/guidance/ta849. Date accessed: May 25.

20. National Institute for Health and Care Excellence (NICE). Upadacitinib for treating active non-radiographic axial spondyloarthritis - Technology appraisal guidance [TA861], 2023. Available from: https://www.nice.org.uk/guidance/ta861. Date accessed: Apr 25.

21. National Institute for Health and Care Excellence (NICE). Upadacitinib for previously treated moderately to severely active Crohn's disease - Technology appraisal guidance [TA905], 2023. Available from: https://www.nice.org.uk/guidance/ta905. Date accessed: Apr 25.

22. National Institute for Health and Care Excellence (NICE). Bimekizumab for treating axial spondyloarthritis - Technology appraisal guidance [TA918], 2023. Available from: https://www.nice.org.uk/guidance/ta918. Date accessed: Apr 25.

23. National Institute for Health and Care Excellence (NICE). Tofacitinib for treating active ankylosing spondylitis - Technology appraisal guidance [TA920], 2023. Available from: https://www.nice.org.uk/guidance/ta920. Date accessed: Apr 25.

24. National Institute for Health and Care Excellence (NICE). Mirikizumab for treating moderately to severely active ulcerative colitis - Technology appraisal guidance [TA925], 2023. Available from: https://www.nice.org.uk/guidance/ta925. Date accessed: Apr 25.

25. National Institute for Health and Care Excellence (NICE). Empagliflozin for treating chronic heart failure with preserved or mildly reduced ejection fraction - Technology appraisal guidance [TA929], 2023. Available from: https://www.nice.org.uk/guidance/ta929. Date accessed: Apr 25.

26. National Institute for Health and Care Excellence (NICE). Zanubrutinib for treating chronic lymphocytic leukaemia - Technology appraisal guidance [TA931], 2023. Available from: https://www.nice.org.uk/guidance/ta931. Date accessed: May 25.

For peer review only

27. National Institute for Health and Care Excellence (NICE). Empagliflozin for treating chronic kidney disease - Technology appraisal guidance [TA942], 2023. Available from: https://www.nice.org.uk/guidance/ta942. Date accessed: Apr 25.

28. National Institute for Health and Care Excellence (NICE). Fluocinolone acetonide intravitreal implant for treating chronic diabetic macular oedema - Technology appraisal guidance [TA953], 2024. Available from: https://www.nice.org.uk/guidance/ta953. Date accessed: Apr 25.

29. National Institute for Health and Care Excellence (NICE). Etrasimod for treating moderately to severely active ulcerative colitis in people aged 16 and over - Technology appraisal guidance [TA956], 2024. Available from: https://www.nice.org.uk/guidance/ta956. Date accessed: Apr 25.

30. National Institute for Health and Care Excellence (NICE). Linzagolix for treating moderate to severe symptoms of uterine fibroids - Technology appraisal guidance [TA996], 2024. Available from: https://www.nice.org.uk/guidance/ta996. Date accessed: Apr 25.

31. National Institute for Health and Care Excellence (NICE). Risankizumab for treating moderately to severely active ulcerative colitis - Technology appraisal guidance [TA998], 2024. Available from: https://www.nice.org.uk/guidance/ta998. Date accessed: Apr 25.

32. National Institute for Health and Care Excellence (NICE). Vibegron for treating symptoms of overactive bladder syndrome - Technology appraisal guidance [TA999], 2024. Available from: https://www.nice.org.uk/guidance/ta999. Date accessed: Apr 25.

33. National Institute for Health and Care Excellence (NICE). Faricimab for treating visual impairment caused by macular oedema after retinal vein occlusion - Technology appraisal guidance [TA1004], 2024. Available from: https://www.nice.org.uk/guidance/ta1004. Date accessed: Apr 25.

34. National Institute for Health and Care Excellence (NICE). Rucaparib for maintenance treatment of relapsed platinum-sensitive ovarian, fallopian tube or peritoneal cancer - Technology appraisal guidance [TA1007], 2024. Available from: https://www.nice.org.uk/guidance/ta1007. Date accessed: Apr 25.

35. National Institute for Health and Care Excellence (NICE). Crovalimab for treating paroxysmal nocturnal haemoglobinuria in people 12 years and over - Technology appraisal guidance [TA1019], 2024. Available from: https://www.nice.org.uk/guidance/ta1019. Date accessed: Apr 25.

36. National Institute for Health and Care Excellence (NICE). Eplontersen for treating hereditary transthyretin-related amyloidosis - Technology appraisal guidance [TA1020], 2024. Available from: https://www.nice.org.uk/guidance/ta1020. Date accessed: Apr 25.

37. National Institute for Health and Care Excellence (NICE). Crizotinib for treating ROS1-positive advanced non-small-cell lung cancer - Technology appraisal guidance [TA1021], 2024. Available from: https://www.nice.org.uk/guidance/ta1021. Date accessed: Apr 25.

38. National Institute for Health and Care Excellence (NICE). Bevacizumab gamma for treating wet age-related macular degeneration - Technology appraisal guidance [TA1022], 2024. Available from: https://www.nice.org.uk/guidance/ta1022. Date accessed: Apr 25.

For peer review only

39. National Institute for Health and Care Excellence (NICE). Ublituximab for treating relapsing multiple sclerosis - Technology appraisal guidance [TA1025], 2024. Available from: https://www.nice.org.uk/guidance/ta1025. Date accessed: Apr 25.

40. National Institute for Health and Care Excellence (NICE). Olaparib for treating BRCA mutation-positive HER2-negative advanced breast cancer after chemotherapy - Technology appraisal guidance [TA1040], 2025. Available from: https://www.nice.org.uk/guidance/ta1040. Date accessed: Apr 25.

41. National Institute for Health and Care Excellence (NICE). Durvalumab with etoposide and either carboplatin or cisplatin for untreated extensive-stage small-cell lung cancer - Technology appraisal guidance [TA1041], 2025. Available from: https://www.nice.org.uk/guidance/ta1041. Date accessed: Apr 25.

42. National Institute for Health and Care Excellence (NICE). Guidance on the use of capecitabine and tegafur with uracil for metastatic colorectal cancer - Technology appraisal guidance [TA61], 2003. Available from: https://www.nice.org.uk/guidance/ta61. Date accessed: May 25.

43. National Institute for Health and Care Excellence (NICE). Frequency of application of topical corticosteroids for atopic eczema - Technology appraisal guidance [TA81], 2004. Available from: https://www.nice.org.uk/guidance/ta81. Date accessed: May 25.

44. National Institute for Health and Care Excellence (NICE). Topotecan for the treatment of relapsed small-cell lung cancer - Technology appraisal guidance [TA184], 2009. Available from: https://www.nice.org.uk/guidance/ta184. Date accessed: May 25.

45. National Institute for Health and Care Excellence (NICE). Capecitabine for the treatment of advanced gastric cancer - Technology appraisal guidance [TA191], 2010. Available from: https://www.nice.org.uk/guidance/ta191. Date accessed: May 25.

46. National Institute for Health and Care Excellence (NICE). Dexamethasone intravitreal implant for the treatment of macular oedema secondary to retinal vein occlusion - Technology appraisal guidance [TA229], 2011. Available from: https://www.nice.org.uk/guidance/ta229. Date accessed: May 25.

47. National Institute for Health and Care Excellence (NICE). Colistimethate sodium and tobramycin dry powders for inhalation for treating pseudomonas lung infection in cystic fibrosis - Technology appraisal guidance [TA276], 2013. Available from: https://www.nice.org.uk/guidance/ta276. Date accessed: May 25.

48. National Institute for Health and Care Excellence (NICE). Ciclosporin for treating dry eye disease that has not improved despite treatment with artificial tears - Technology appraisal guidance [TA369], 2015. Available from: https://www.nice.org.uk/guidance/ta369. Date accessed: May 25.

49. National Institute for Health and Care Excellence (NICE). Dasatinib, nilotinib and high-dose imatinib for treating imatinib-resistant or intolerant chronic myeloid leukaemia - Technology appraisal guidance [TA425], 2016. Available from: https://www.nice.org.uk/guidance/ta425. Date accessed: May 25.

50. National Institute for Health and Care Excellence (NICE). Dasatinib, nilotinib and imatinib for untreated chronic myeloid leukaemia - Technology appraisal guidance [TA426], 2016. Available from: https://www.nice.org.uk/guidance/ta426. Date accessed: May 25.

For peer review only

51. National Institute for Health and Care Excellence (NICE). Everolimus for advanced renal cell carcinoma after previous treatment - Technology appraisal guidance [TA432], 2017. Available from: https://www.nice.org.uk/guidance/ta432. Date accessed: May 25.

52. National Institute for Health and Care Excellence (NICE). Ustekinumab for moderately to severely active Crohn's disease after previous treatment - Technology appraisal guidance [TA456], 2017. Available from: https://www.nice.org.uk/guidance/ta456. Date accessed: May 25.

53. National Institute for Health and Care Excellence (NICE). Atezolizumab for treating locally advanced or metastatic non-small-cell lung cancer after chemotherapy - Technology appraisal guidance [TA520], 2018. Available from: https://www.nice.org.uk/guidance/ta520. Date accessed: May 25.

54. National Institute for Health and Care Excellence (NICE). Pembrolizumab for treating relapsed or refractory classical Hodgkin lymphoma - Technology appraisal guidance [TA540], 2018. Available from: https://www.nice.org.uk/guidance/ta540. Date accessed: May 25.

55. National Institute for Health and Care Excellence (NICE). Venetoclax with rituximab for previously treated chronic lymphocytic leukaemia - Technology appraisal guidance [TA565], 2019. Available from: https://www.nice.org.uk/guidance/ta561. Date accessed: May 25.

56. National Institute for Health and Care Excellence (NICE). Encorafenib with binimetinib for unresectable or metastatic BRAF V600 mutation-positive melanoma - Technology appraisal guidance [TA562], 2019. Available from: https://www.nice.org.uk/guidance/ta562. Date accessed: May 25.

57. National Institute for Health and Care Excellence (NICE). Abemaciclib with an aromatase inhibitor for previously untreated, hormone receptor-positive, HER2-negative, locally advanced or metastatic breast cancer - Technology appraisal guidance [TA563], 2019. Available from: https://www.nice.org.uk/guidance/ta563. Date accessed: May 25.

58. National Institute for Health and Care Excellence (NICE). Benralizumab for treating severe eosinophilic asthma - Technology appraisal guidance [TA565], 2019. Available from: https://www.nice.org.uk/guidance/ta565. Date accessed: May 25.

59. National Institute for Health and Care Excellence (NICE). Ustekinumab for treating moderately to severely active ulcerative colitis - Technology appraisal guidance [TA633], 2020. Available from: https://www.nice.org.uk/guidance/ta633. Date accessed: May 25.

60. National Institute for Health and Care Excellence (NICE). Brigatinib for ALK-positive advanced non-small-cell lung cancer that has not been previously treated with an ALK inhibitor - Technology appraisal guidance [TA670], 2021. Available from: https://www.nice.org.uk/guidance/ta670. Date accessed: May 25.

61. National Institute for Health and Care Excellence (NICE). Mepolizumab for treating severe eosinophilic asthma - Technology appraisal guidance [TA671], 2021. Available from: https://www.nice.org.uk/guidance/ta671. Date accessed: May 25.

62. National Institute for Health and Care Excellence (NICE). Anakinra for treating Still's disease - Technology appraisal guidance [TA685], 2021. Available from: https://www.nice.org.uk/guidance/ta685. Date accessed: May 25.

For peer review only

63. National Institute for Health and Care Excellence (NICE). Selective internal radiation therapies for treating hepatocellular carcinoma - Technology appraisal guidance [TA688], 2024. Available from: https://www.nice.org.uk/guidance/ta688. Date accessed: May 25.

64. National Institute for Health and Care Excellence (NICE). Atezolizumab monotherapy for untreated advanced non-small-cell lung cancer - Technology appraisal guidance [TA705], 2021. Available from: https://www.nice.org.uk/guidance/ta705. Date accessed: May 25.

65. National Institute for Health and Care Excellence (NICE). Diroximel fumarate for treating relapsing–remitting multiple sclerosis - Technology appraisal guidance [TA794], 2022. Available from: https://www.nice.org.uk/guidance/ta794. Date accessed: May 25.

66. National Institute for Health and Care Excellence (NICE). Somatrogon for treating growth disturbance in children and young people aged 3 years and over - Technology appraisal guidance [TA863], 2023. Available from: https://www.nice.org.uk/guidance/ta863. Date accessed: May 25.

67. National Institute For Health and Care Excellence (NICE). Vutrisiran for treating hereditary transthyretin-related amyloidosis - Technology appraisal guidance [TA868], 2023. Available from: https://www.nice.org.uk/guidance/ta868. Date accessed: Mar 24.

68. National Institute for Health and Care Excellence (NICE). Eptinezumab for preventing migraine - Technology appraisal guidance [TA871], 2023. Available from: https://www.nice.org.uk/guidance/ta871. Date accessed: May 25.

69. National Institute for Health and Care Excellence (NICE). Risankizumab for previously treated moderately to severely active Crohn's disease - Technology appraisal guidance [TA888], 2023. Available from: https://www.nice.org.uk/guidance/ta888. Date accessed: May 25.

70. National Institute for Health and Care Excellence (NICE). Pegunigalsidase alfa for treating Fabry disease - Technology appraisal guidance [TA915], 2023. Available from: https://www.nice.org.uk/guidance/ta915. Date accessed: May 25.

71. National Institute for Health and Care Excellence (NICE). Bimekizumab for treating active psoriatic arthritis - Technology appraisal guidance [TA916], 2023. Available from: https://www.nice.org.uk/guidance/ta916. Date accessed: May 25.

72. National Institute for Health and Care Excellence (NICE). Momelotinib for treating myelofibrosis-related splenomegaly or symptoms - Technology appraisal guidance [TA957], 2024. Available from: https://www.nice.org.uk/guidance/ta957. Date accessed: May 25.

73. National Institute for Health and Care Excellence (NICE). Atogepant for preventing migraine - Technology appraisal guidance [TA973], 2024. Available from: https://www.nice.org.uk/guidance/ta973. Date accessed: May 25.

74. National Institute for Health and Care Excellence (NICE). Selective internal radiation therapy with QuiremSpheres for treating unresectable advanced hepatocellular carcinoma - Technology appraisal guidance [TA985], 2024. Available from: https://www.nice.org.uk/guidance/ta985. Date accessed: May 25.

75. National Institute for Health and Care Excellence (NICE). Tenecteplase for treating acute ischaemic stroke - Technology appraisal guidance [TA990], 2024. Available from: https://www.nice.org.uk/guidance/ta990. Date accessed: May 25.

For peer review only

76. National Institute for Health and Care Excellence (NICE). Trastuzumab deruxtecan for treating HER2-low metastatic or unresectable breast cancer after chemotherapy - Technology appraisal guidance [TA992], 2024. Available from: https://www.nice.org.uk/guidance/ta992. Date accessed: May 25.

77. National Institute for Health and Care Excellence (NICE). Pembrolizumab with platinum- and fluoropyrimidine-based chemotherapy for untreated advanced HER2-negative gastric or gastro-oesophageal junction adenocarcinoma - Technology appraisal guidance [TA997], 2024. Available from: https://www.nice.org.uk/guidance/ta997. Date accessed: May 25.

78. National Institute for Health and Care Excellence (NICE). Evinacumab for treating homozygous familial hypercholesterolaemia in people 12 years and over - Technology appraisal guidance [TA1002], 2024. Available from: https://www.nice.org.uk/guidance/ta1002. Date accessed: May 25.

79. National Institute for Health and Care Excellence (NICE). Futibatinib for previously treated advanced cholangiocarcinoma with FGFR2 fusion or rearrangement - Technology appraisal guidance [TA1005], 2024. Available from: https://www.nice.org.uk/guidance/ta1005. Date accessed: May 25.

80. National Institute for Health and Care Excellence (NICE). Latanoprost–netarsudil for previously treated primary open-angle glaucoma or ocular hypertension Technology appraisal guidance [TA1009], 2024. Available from: https://www.nice.org.uk/guidance/ta1009. Date accessed: May 25.

81. National Institute for Health and Care Excellence (NICE). Fenfluramine for treating seizures associated with Lennox–Gastaut syndrome in people 2 years and over - Technology appraisal guidance [TA1050], 2025. Available from: https://www.nice.org.uk/guidance/ta1050. Date accessed: May 25.

82. National Institute for Health and Care Excellence (NICE). Tislelizumab for treating unresectable advanced oesophageal squamous cell cancer after platinum-based chemotherapy [ID4070], 2025. Available from: https://www.nice.org.uk/guidance/indevelopment/gid-ta11042. Date accessed: May 25.

83. National Institute for Health and Care Excellence (NICE). Somapacitan for treating growth hormone deficiency in people 3 to 17 years [ID6178], 2025. Available from: https://www.nice.org.uk/guidance/indevelopment/gid-ta11153. Date accessed: May 25.

84. National Institute for Health and Care Excellence (NICE). Guselkumab for treating moderately to severely active ulcerative colitis [ID6237], 2025. Available from: https://www.nice.org.uk/guidance/indevelopment/gid-ta11247. Date accessed: May 25.

85. National Institute for Health and Care Excellence (NICE). Guselkumab for previously treated moderately to severely active Crohn's disease [ID6238], 2025. Available from: https://www.nice.org.uk/guidance/indevelopment/gid-ta11245. Date accessed: May 25.

86. National Institute for Health and Care Excellence (NICE). Mirikizumab for treating moderately to severely active Crohn's disease [ID6244], 2025. Available from: https://www.nice.org.uk/guidance/indevelopment/gid-ta11267. Date accessed: May 25.

87. National Institute for Health and Care Excellence (NICE). Nivolumab as neoadjuvant (with chemotherapy) and adjuvant (as monotherapy) treatment for resectable non-small-cell

For peer review only

lung cancer [ID6310], 2025. Available from: https://www.nice.org.uk/guidance/indevelopment/gid-ta11504. Date accessed: May 25.

88. National Institute for Health and Care Excellence (NICE). Vanzacaftor–tezacaftor–deutivacaftor for treating cystic fibrosis with 1 or more F508del mutations in the CFTR gene in people aged 6 years and over [ID6372], 2025. Available from: https://www.nice.org.uk/guidance/indevelopment/gid-ta11430. Date accessed: May 25.

89. National Institute for Health and Care Excellence (NICE). Abiraterone (originator and generics) for treating newly diagnosed high-risk hormone-sensitive metastatic prostate cancer (review of TA721) [ID6378], 2025. Available from: https://www.nice.org.uk/guidance/indevelopment/gid-ta11730. Date accessed: May 25.

90. National Institute for Health and Care Excellence (NICE). Rilzabrutinib for treating persistent or chronic immune thrombocytopenia in people aged 12 and over [ID6395], 2025. Available from: https://www.nice.org.uk/guidance/awaiting-development/gid-ta11491. Date accessed: May 25.

91. National Institute for Health and Care Excellence (NICE). Darolutamide with androgen deprivation therapy for treating hormone-sensitive metastatic prostate cancer ID6452, 2025. Available from: https://www.nice.org.uk/guidance/indevelopment/gid-ta11557. Date accessed: May 25.

92. National Institute for Health and Care Excellence (NICE). Empagliflozin for treating chronic heart failure with reduced ejection fraction - Technology appraisal guidance [TA773], 2022. Available from: https://www.nice.org.uk/guidance/ta773. Date accessed: May 25.

93. National Institute for Health and Care Excellence (NICE). Empagliflozin for treating chronic heart failure with preserved or mildly reduced ejection fraction - Technology appraisal guidance [TA929] - Final draft guidance. Available from: https://www.nice.org.uk/guidance/ta929/history. Date accessed: Apr 25.

94. National Institute for Health and Care Excellence (NICE). NICE health technology evaluations: the manual, 2023. Available from: https://www.nice.org.uk/process/pmg36. Date accessed: Apr 25.

95. Costello Medical. NICE's 'Proportionate Approach to Technology Appraisals (PATT)': The Story So Far, 2024. Available from: https://www.costellomedical.com/what-we-do/value-and-access/hta/nices-proportionate-approach-to-technology-appraisals/. Date accessed: May 25.

For peer review only

Online Supplementary File 3

Point-and-density plot methods

Development of point-and-density plots

The framework (i.e., point-and-density plots) described in this research does not seek to replace any current methods with regards to performing Bayesian network meta-analyses (NMAs). Indeed, it is recommended that all National Institute for Care and Health Excellence (NICE) Decision Support Unit (DSU) guidelines[1] with regards to indirect treatment comparisons continue to be followed. Instead, the proposed framework should be viewed as an add-on to existing approaches for NMAs, with all relevant procedures and outputs continuing to be provided (e.g., forest plots, trace plots, and Gelman-Rubin diagrams). As such, the following detailed methodology may be considered an 'add-on' analysis to be specifically utilised for NICE Cost Comparison Evaluations (CCEs) and other Technology Appraisals (TA) where an assessment of clinical similarity, or non-inferiority, is required.

The statistics underpinning the generation of point-and-density plots are provided below.

When performing a Bayesian NMA, density plots are generated for each comparison in the network, an example of which is shown in Supplementary Figure 2, which is an example of the default output of the *gemtc* R package.[2] Such a density plot is generated using the values of iterations from across all chains used within a Bayesian analysis and can be produced for each treatment comparison.

For peer review only

Supplementary Figure 2. Default density plot from the *gemtc* R package for the mean difference between an example treatment and comparator.

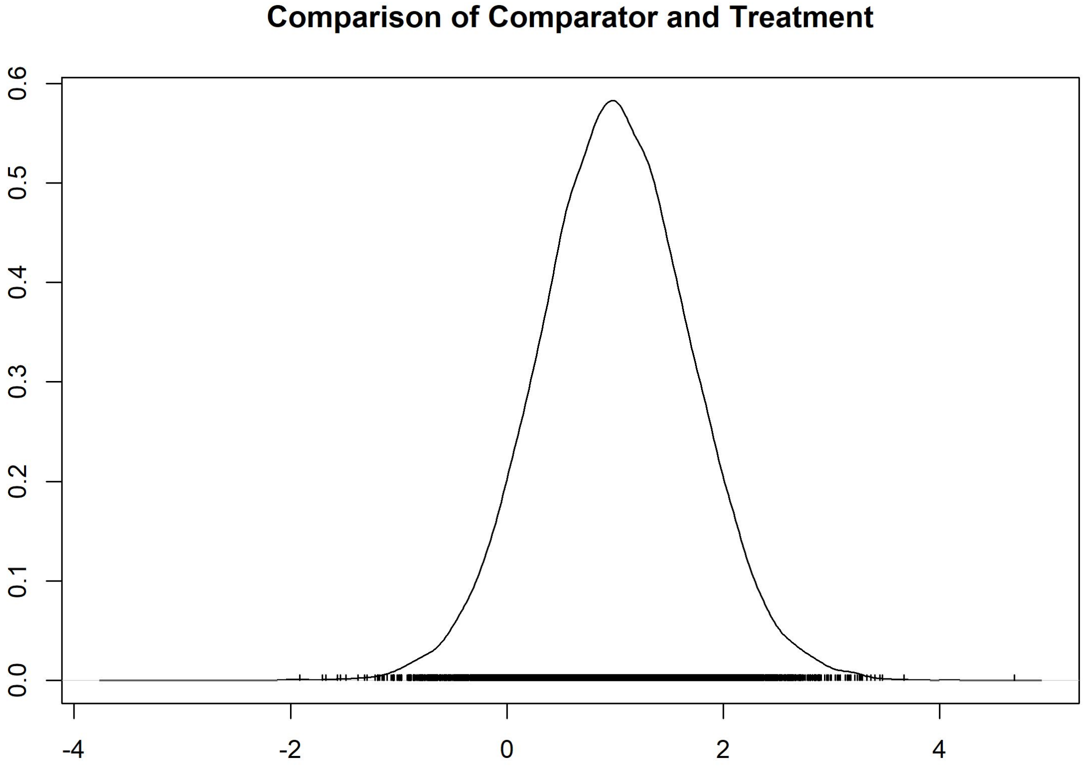

The default density plots produced by the *gemtc* R package[2] have minimal labels. Within Supplementary Figure 2, the x-axis denotes the mean difference of the comparison of the example comparator to the example treatment, while the y-axis denotes the corresponding density.

For each treatment comparison (e.g., the example comparator to the example treatment in Supplementary Figure 2) it is possible to generate an empirical cumulative density function (ECDF) from the density plot. An example of such ECDF is shown in Supplementary Figure 3. In contrast to a regular density plot, an ECDF shows the cumulative density across all values (e.g., mean difference in in Supplementary Figure 3). Within the example from in Supplementary Figure 3, the ECDF will have a cumulative density of 0 at the minimum mean difference reported for the comparison of the example comparator to the example treatment. Likewise, within the example from in Supplementary Figure 3, the ECDF will have a cumulative density of 1 at the maximum mean difference reported for the comparison of the example comparator to the example treatment. Within the example from in Supplementary Figure 3, the ECDF can be used to determine the cumulative density of any point between the minimum and maximum mean difference reported for the comparison of the example comparator to the example treatment. Accordingly, by specifying a value that corresponds to a non-inferiority margin (NIM), minimal clinically important difference (MCID), or other threshold, it is possible to determine the cumulative density of points that fall below, and above, this threshold. in Supplementary Figure 3shows an example of the application of a

For peer review only - http://bmjopen.bmj.com/site/about/guidelines.xhtml

NIM to an ECDF, where a NIM of -0.20 is specified. As illustrated in Supplementary Figure 4, the NIM of -0.20 would correspond to a cumulative density of 0.041.

Supplementary Figure 3. Empirical cumulative density function for the comparison of an example treatment and comparator.

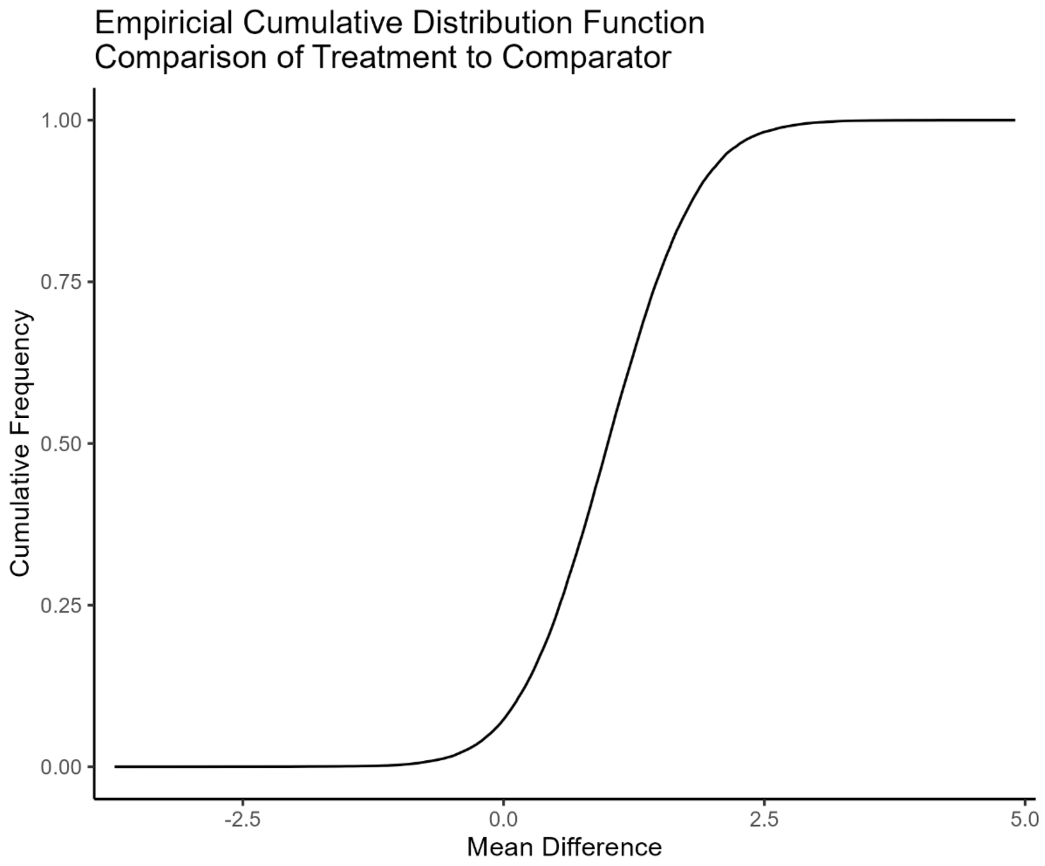

w only

Supplementary Figure 4. Empirical cumulative density function, with a non-inferiority margin, for the comparison of an example treatment and comparator.

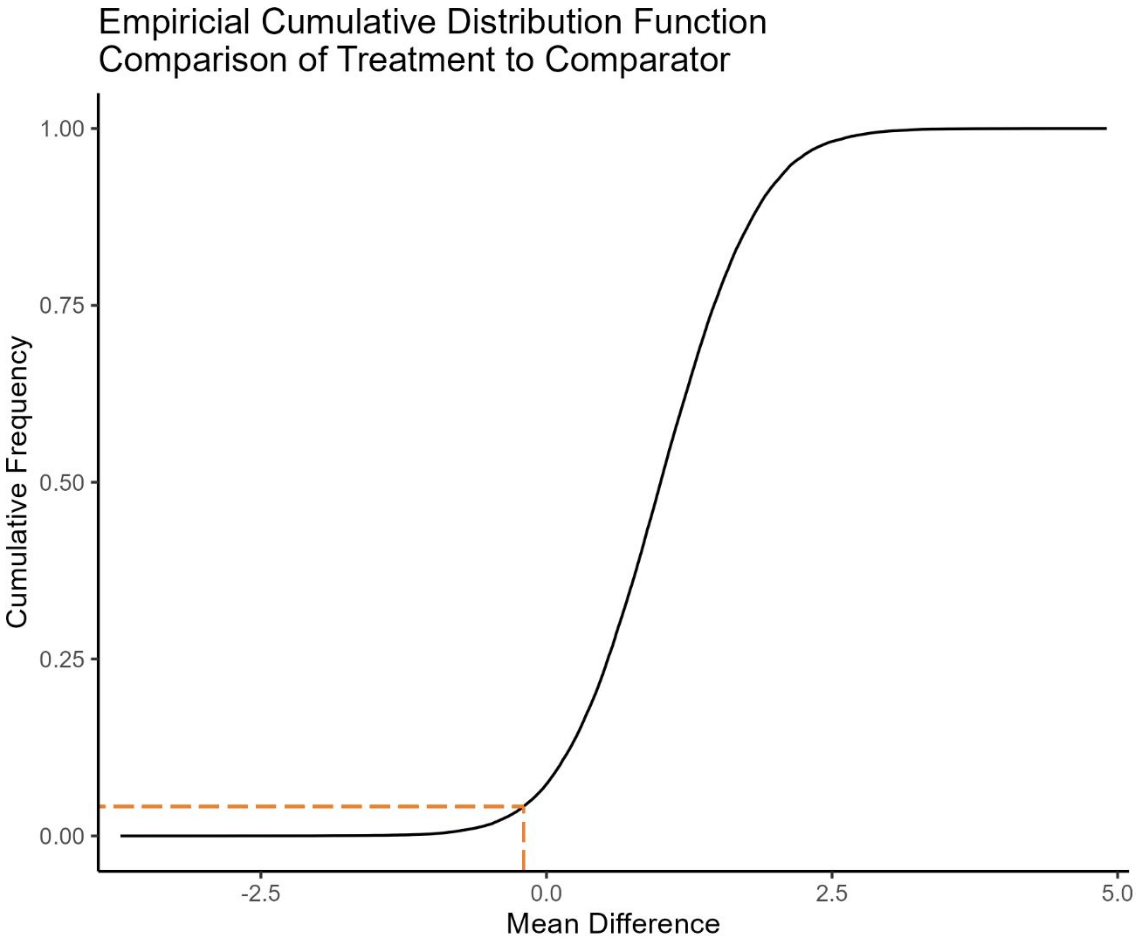

Dashed orange line corresponds to a non-inferiority margin of -0.20 and a corresponding cumulative density of 0.041.

Given the nature of Bayesian analyses, the ECDF for a given comparison can be used to determine the proportion of iterations from the Bayesian analysis that fall below a given threshold (e.g., NIM). Accordingly, within the example shown in Supplementary Figure 4, the cumulative density represents the probability that the mean difference of the comparison of the example comparator to the example treatment falls below the given threshold. As such, the probability that the mean difference of the comparison falls above the given threshold can be calculated. Therefore, by combining the results of the ECDF with those of a traditional density plot and forest plot, it is possible to convey the results of all three separate analyses within a single readily interpretable figure (i.e., a point-and-density plot). An example of a point-and-density plot is shown in Supplementary Figure 5, again using the comparison of the example comparator to the example treatment as above.

For peer review only - http://bmjopen.bmj.com/site/about/guidelines.xhtml

Supplementary Figure 5. Point-and-density plot for the comparison of an example treatment and comparator.

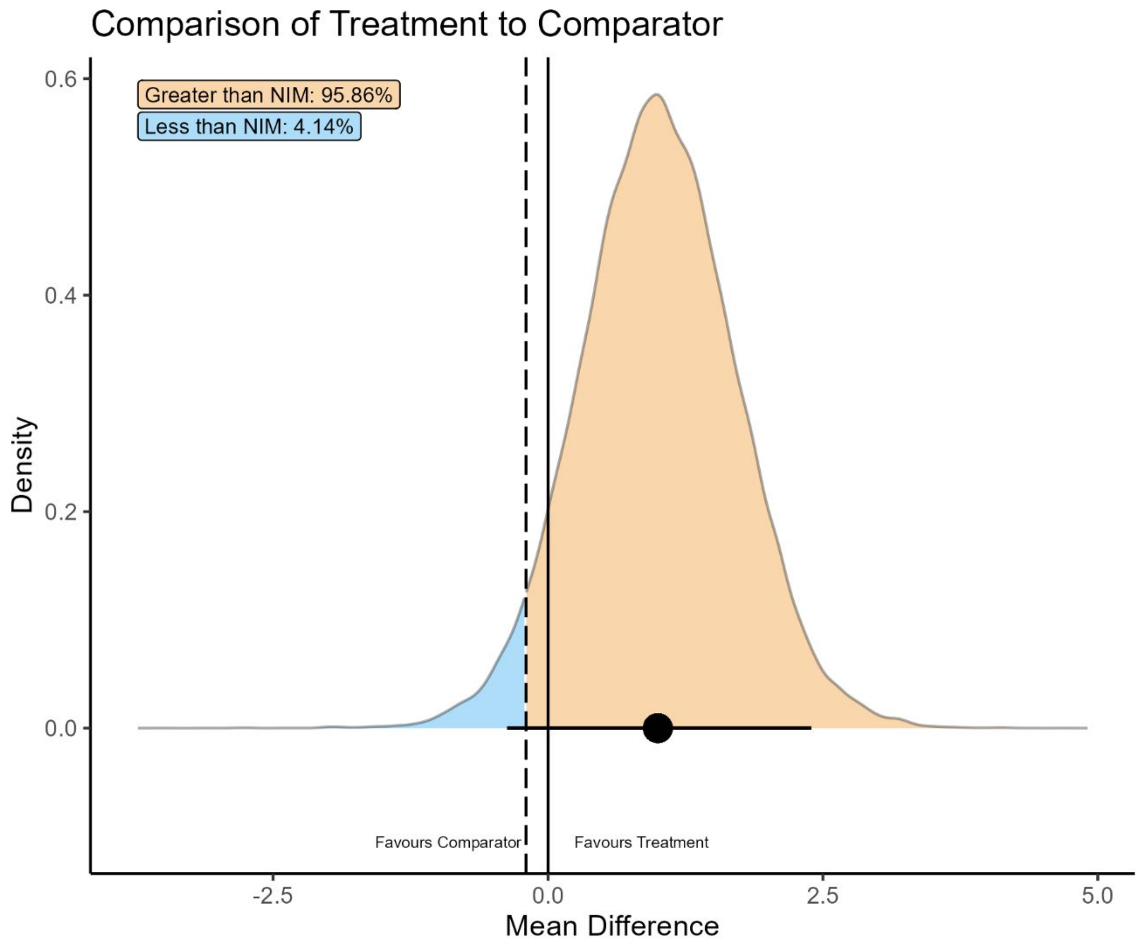

The threshold for traditional hypothesis testing is shown by the solid line, while the dashed line indicates the NIM. The area that falls under (above) the NIM is shown in blue (orange). The probability that the effect size falls under, or above, the NIM is shown in the upper left corner of the plot. The pooled estimate is shown by the black circle, with the error bars corresponding to 95% credible intervals.

Point-and-density plots have been designed to be readily interpretable and showcase data not previously reported from Bayesian NMAs, in addition to data from existing figures (e.g., forest plots). Within Supplementary Figure 5, the thick black line denotes the threshold for traditional hypothesis testing (i.e., either 0 or 1 depending on the effect estimate), while the dashed black line denotes the specified NIM, MCID, or another designated threshold. The point estimate, denoted by the black circle, indicates the pooled estimate for the comparison, with the error bars indicating 95% credible intervals. The point estimate shown within the point-and-density plot will be the same as that shown within a typical forest plot. As shown by Supplementary Figure 5, the density curve aligns with that shown from the typical output of the *gemtc* R package[2] (Supplementary Figure 2). The area under the density curve that falls above and below the NIM is shown in blue and orange, respectively. Likewise, the probability that the effect estimate for the comparison falls above or below the NIM (dashed black line) is shown in the top-left corner, with these values being derived using the

corresponding ECDF. Furthermore, within the point-and-density figure, labels have been added to denote whether a given effect size favours the treatment or the comparator.

As shown by Supplementary Figure 5, the pooled estimate for the comparison of the example comparator to the example treatment is 1.00, with 95% credible intervals of -0.34 to 2.40. Accordingly, based solely on traditional statistical testing, there was no statistically significant difference in treatment effects between the example comparator and the example treatment. In addition, the 95% credible intervals overlap the specified NIM of -0.20, indicating that non-inferiority of the example treatment compared to the example comparator is not demonstrated. However, through the use of the ECDF, the point-and-density plot (Supplementary Figure 5) depicts that there was a 95.87% probability of the example treatment being non-inferior to the example comparator. It is suggested that committees initially focus on whether the 95% credible intervals overlap the NIM to determine whether a treatment can be considered to be non-inferior. However, as in the worked example, the use of probabilities of non-inferiority may provide valuable information, particularly where non-inferiority has not been demonstrated. Additionally, the plots of ECDF functions do not necessarily need to be presented within CCE submissions; instead, it is recommended that committees primarily focus on the results of point-and-density figures.

The above example of the comparison of the example comparator to the example treatment uses a NIM of -0.20. However, for some comparisons a NIM, MCID, or other designated threshold may not be available. Accordingly, when clinically validated thresholds are not available, it is recommended that the corresponding threshold be set to either 0 (for effect estimates such as mean difference) or 1 (for effect estimates such as hazard ratios or odds ratios). By using this approach it is possible to determine the probability that the effect size for the comparison of two treatments will fall above, or below, the corresponding threshold. However, it should be noted that this approach will result in more conservative estimations of non-inferiority compared to using a clinically validated NIM, MCID, or another relevant threshold. Furthermore, in situations where a NIM, or similar threshold, is not reported, by using the corresponding ECDF it is possible to determine a hypothetical threshold. Such a hypothetical threshold can be used to determine the required threshold for a given, arbitrary, probability that a treatment is non-inferior to a comparator. For instance, using the above example, comparing the example comparator to the example treatment, the ECDF reveals that a hypothetical threshold of -0.65 would be required for there to be 99% probability that the example treatment was non-inferior to the example comparator. However, it is important to note that such a hypothetical threshold should not be considered to be clinically appropriate or meaningful. However, in the absence of NIM, MCID, or other designated threshold, a hypothetical threshold can help inform a committee by indicating whether a

For peer review only

118 relatively large, or small, hypothetical threshold would be required for there to be a certain, arbitrary, probability that a treatment is non-inferior to a comparator. Ultimately, when deriving a hypothetical threshold, the selection of the desired probability of non-inferiority may be arbitrarily selected. However, the authors suggest that a 95% probability of non-inferiority may be selected for a hypothetical threshold. Importantly, the selection of a 95% threshold represents a 'high bar' for any assessments of non-inferiority in the absence of a clinically validated NIM or MCID.

125 ***Code***

126 The code to perform the analyses described above has been developed in R v4.2.0[3] and utilises the *gemtc*[2], *data.table*[4], *igraph*[5], *tidyverse*[6], *magrittr*[7], *and ggplot2*[8] *packages.* The code was developed by one researcher and subsequently independently reviewed and tested by a second researcher on two separate occasions. The R code was tested using synthetic datasets to ensure that it worked as expected for a variety of link functions (e.g., *cloglog*, *identity*, *logit*, and *log*) and data types (e.g., mean and standard deviations or the overall number of patients and the number of responders).

133 The results of the code were also compared to the *gemtcPlus* package[9] which allows users to determine the probability of that an effect size falls above, or below, a threshold of 0 or 1. While users of the *gemtcPlus* package[9] cannot specify an alternative threshold, comparisons of the results obtained using the *gemtcPlus* package[9] and the approach detailed here aligns.

137 R code and NMA output to generate example point-and-density plots from the case study for crovalimab for Paroxysmal Nocturnal Haemoglobinuria (TA1019)[10] are provided as supplementary materials (Online Supplementary Files 4 and 5).

140

141 **Crovalimab for Paroxysmal Nocturnal Haemoglobinuria (TA1019)**

142 ***Network of evidence***

143 Within TA1019[10], the company identified a connected evidence network (Supplementary Figure 6) that comprised crovalimab and the comparators of eculizumab, ravulizumab, and standard of care for the endpoint of transfusion avoidance.

For peer review only

Supplementary Figure 6. Network of evidence for studies included within network meta-analyses for the percentage of patients achieving transfusion avoidance for the overall population.

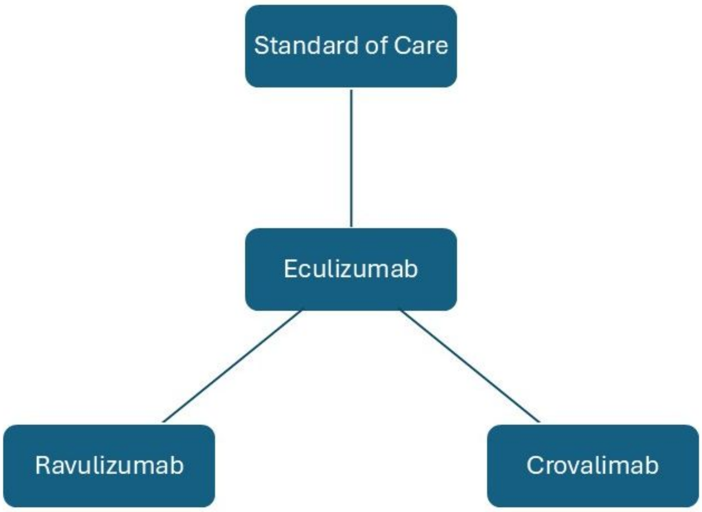

Raw data

The raw data used to perform the original analyses was reported in TA1019[10], as such these data were used to perform the re-analyses for the overall population. Data were extracted from TA1019[10] by one researcher and independently verified by a second researcher. Overall, the raw data used in the original analysis and re-analysis are shown in Supplementary Table 7.

Supplementary Table 7. Data used in the analyses and re-analyses of the network meta-analyses for the percentage of patients achieving transfusion avoidance (taken from page 62 of the company submission).

Study	Population	Treatment	Mean	Standard Error
Study 301[11]				
	Naïve	Ravulizumab	0.736	0.039
	Naïve	Eculizumab	0.661	0.043
TRIUMPH[12]				
	Naïve	Eculizumab	0.51	0.078
	Naïve	Standard of Care	0	0.025
COMMODORE 2[13]				
	Naïve	Crovalimab	0.664	0.042
	Naïve	Eculizumab	0.681	0.058
Study 302[14]				
	Experienced	Ravulizumab	0.876	0.033
	Experienced	Eculizumab	0.827	0.038
COMMODORE 1[15]				

For peer review only - http://bmjopen.bmj.com/site/about/guidelines.xhtml

	Experienced	Crovalimab	0.795	0.069
	Experienced	Eculizumab	0.811	0.069

Network meta-analyses

As detailed above, analyses were performed in R v4.2.0[3] and utilised the *gemtc*[2]*, data.table*[4]*, igraph*[5]*, tidyverse*[6]*, magrittr*[7]*, and ggplot2*[8] R packages. Network meta-analyses were performed using a random-effects model, with 5 chains, a burn in period of 5000 iterations, a total of 100,000 iterations, and a thinning margin of 10. As the outcome of transfusion avoidance was analysed using mean differences, an identity link function was specified.

References

1. University of Sheffield - NICE Decision Support Unit. Full list of technical support documents (TSDs), 2025. Available from: https://www.sheffield.ac.uk/nice-dsu/tsds/full-list. Date accessed: May 25.
2. van Valkenhoef G, Kuiper J. gemtc: Network Meta-Analysis Using Bayesian Methods v1.0.2. 2023.
3. Computing TRFfS. R version 4.2.0. 2022.
4. Barrett T, Dowle M, Srinivasan A, Gorecki J, Chirico M, Hocking T, et al. data.table: Extension of `data.frame` v1.17.0. 2025.
5. Csardi G, Nepusz T. The igraph software package for complex network research v2.0.3. 2006.
6. Wickham H, Averick M, Bryan J, Chang W, McGowan LDA, François R, et al. Welcome to the Tidyverse. *Journal of Open Source Software* 2019; **4**: 1686.
7. Bache S, Wickham H. magrittr: A Forward-Pipe Operator for R v2.0.3. 2022.
8. Wickham H, Sievert C. *ggplot2: Elegant Graphics for Data Analysis*: Springer New York; 2009.
9. Gsteiger S, Howlett N, Ashlee B. gemtcPlus: Provides a suite of extension functions for NMA using the `gemtc` package v1.0.0. 2025.
10. National Institute for Health and Care Excellence (NICE). Crovalimab for treating paroxysmal nocturnal haemoglobinuria in people 12 years and over - Technology appraisal guidance [TA1019], 2024. Available from: https://www.nice.org.uk/guidance/ta1019. Date accessed: Apr 25.
11. Lee JW, Sicre de Fontbrune F, Wong Lee Lee L, Pessoa V, Gualandro S, Füreder W, et al. Ravulizumab (ALXN1210) vs eculizumab in adult patients with PNH naive to complement inhibitors: the 301 study. *Blood* 2019; **133**: 530-9.
12. Hillmen P, Young NS, Schubert J, Brodsky RA, Socié G, Muus P, et al. The complement inhibitor eculizumab in paroxysmal nocturnal hemoglobinuria. *The New England journal of medicine* 2006; **355**: 1233-43.
13. Röth A, He G, Tong H, Lin Z, Wang X, Chai-Adisaksopha C, et al. Phase 3 randomized COMMODORE 2 trial: Crovalimab versus eculizumab in patients with paroxysmal nocturnal hemoglobinuria naive to complement inhibition. *Am J Hematol* 2024; **99**: 1768-77.
14. Kulasekararaj AG, Hill A, Rottinghaus ST, Langemeijer S, Wells R, Gonzalez-Fernandez FA, et al. Ravulizumab (ALXN1210) vs eculizumab in C5-inhibitor-experienced adult patients with PNH: the 302 study. *Blood* 2019; **133**: 540-9.
15. Scheinberg P, Clé DV, Kim JS, Nur E, Yenerel MN, Barcellini W, et al. Phase 3 randomized COMMODORE 1 trial: Crovalimab versus eculizumab in complement inhibitor-

For peer review only

experienced patients with paroxysmal nocturnal hemoglobinuria. *Am J Hematol* 2024; **99**: 1757-67.

For peer review only
